# Comorbidities in Juvenile-Onset Rheumatic Diseases: A Systematic Review and Meta-Analysis

**DOI:** 10.3390/children13080995

**Published:** 2026-07-28

**Authors:** Sab Siddiq, Shabnam Cheetham, Clare E. Pain, Eve M. D. Smith, Sizheng Steven Zhao, Liza J. McCann, David M. Hughes

**Affiliations:** 1 Department of Health Data Science, Institute of Population Health, University of Liverpool, Waterhouse Building, Block F, 1–5 Brownlow Street, Liverpool L69 3GL, UK; sab.siddiq@liverpool.ac.uk; 2 Department of Paediatric Rheumatology, Royal Manchester Children’s Hospital, Manchester University NHS Foundation Trust, Manchester M13 9WL, UK; shabnam.cheetham@nhs.net; 3 Department of Paediatric Rheumatology, Institute in the Park, Alder Hey Children’s NHS Foundation Trust, Liverpool L14 5AB, UK; clare.pain@alderhey.nhs.uk; 4 Department of Women and Children’s Health, Institute of Life Course and Medical Sciences, University of Liverpool, Liverpool L8 7SS, UK; 5 School of Infection and Immunity, University of Glasgow, Glasgow G12 8TA, UK; eve.smith@glasgow.ac.uk; 6 Centre for Musculoskeletal Research, Faculty of Biology, Medicine and Health, School of Biological Sciences, Division of Musculoskeletal & Dermatological Sciences, University of Manchester, Oxford Road, Manchester M13 9PL, UK; steven.zhao.25@gmail.com; 7 NIHR Manchester Biomedical Research Centre, Manchester University NHS Foundation Trust, Manchester M13 9WU, UK

**Keywords:** Juvenile Idiopathic Arthritis, Juvenile-onset Systemic Lupus Erythematosus, Juvenile Dermatomyositis, comorbidity, systematic review, adults, meta-analysis

## Abstract

**Highlights:**

**What are the main findings?**

- This systematic review reports patients with Juvenile Idiopathic Arthritis (JIA), Juvenile-onset Systemic Lupus Erythematosus (jSLE) and Juvenile Dermatomyositis (JDM) to be at an increased risk of a wide range of comorbidities in both childhood and adulthood.
- This highlights the additional disease burden and impact on quality of life across the life-course of patients extending beyond their primary disease.

**What is the implication of the main finding?**

- Findings suggest the need for a proactive, multidisciplinary approach to monitoring and managing comorbidities in patients with juvenile-onset rheumatic disease.
- This review exposes the limitations of current studies and identifies the need for more robust long-term follow up to better determine the risk of comorbidities in patients with childhood-onset rheumatic disease.

**Abstract:**

**Background/Objectives**: Patients with childhood-onset rheumatic diseases may be at additional risk of developing other health conditions. This systematic review aimed to (i) identify and describe the comorbidities associated with three significant childhood-onset rheumatic diseases—Juvenile Idiopathic Arthritis (JIA), Juvenile-onset Systemic Lupus Erythematosus (jSLE), and Juvenile Dermatomyositis (JDM); (ii) describe comorbidity prevalence and incidence reported as apparent in childhood or adulthood, and (iii) compare these comorbidity estimates with control groups. **Methods**: PubMed, Web of Science, and Scopus databases were systematically searched without restrictions, in accordance with PRISMA guidelines. Where three or more studies reported the same comorbidity, a meta-analysis was performed using random-effect models. The risk of bias and study quality were assessed using an adjusted version of the Newcastle–Ottawa Scale. **Results**: Comorbidities were reported in 136,072 patients, of which 115,062 (84.56%) presented in childhood. There was significant heterogeneity within the results. The comorbidities presenting in childhood were uveitis (13.95%, 95% CI 11.79–16.43) in JIA patients, chronic kidney disease (48.38%, 95% CI 0.67–99.24) in jSLE patients, and calcinosis (29.70%, 95% CI 25.91–33.81) in JDM patients. The comorbidities identified in adult populations with childhood-onset rheumatic disease were uveitis (14.46%, 95% CI9.76–20.90) in JIA, hypertension (18.30%, 95% CI 7.52–38.16) in jSLE, and calcinosis (40.37%, 19.02–66.11) in JDM patients. Almost all comorbidities that were compared to control groups were more common in patients with childhood-onset rheumatic disease if statistically significant. **Conclusions**: Uveitis, chronic kidney disease/hypertension and calcinosis were most commonly identified among JIA, jSLE and JDM patients, respectively. Patients with the three childhood-onset rheumatic diseases evaluated in this systematic review were often found to be at higher risk of comorbidities compared to controls. This finding supports the need for the proactive, multidisciplinary management of comorbidities by clinicians and highlights the breadth of disease burden for patients.

## 1. Introduction

Childhood-onset rheumatological conditions, including Juvenile Idiopathic Arthritis (JIA), Juvenile-onset Systemic Lupus Erythematosus (jSLE) and Juvenile Dermatomyositis (JDM), are associated with increased risk of disease damage, functional impairment, disability, decreased quality of life, and comorbidities [1,2,3,4,5,6,7,8]. As children progress into adulthood, ongoing disease activity from JIA, jSLE and JDM and the requirement for medication can be associated with a higher risk of damage or comorbidities [4,5,6,7,8]. Studies evaluating medium to long-term outcomes of these diseases have demonstrated a significant burden for young people and their families [9,10,11,12,13,14].

Comorbidities associated with adult-onset rheumatic disease have been reported [15,16,17], but less is known about the burden of comorbidity in childhood-onset rheumatic disease. This is partly due to a suboptimal linkage between paediatric and adult research registries, and that long-term data collection for these rare diseases may be lost as young people transition to adult services [18,19,20]. We have found through focus group discussion that young people are concerned about the long-term outcomes of their disease and may be willing to make lifestyle changes to decrease the risk of comorbidities, provided that information is given sensitively with clear practical steps for reducing risk [13].

This systematic review aimed to (i) identify and describe reported comorbidities of JIA, jSLE, and JDM patients in published articles, (ii) describe comorbidity prevalence and incidence reported as being apparent in childhood and/or adulthood, and (iii) describe comparisons between comorbidity burden in juvenile rheumatic disease and in control groups.

## 2. Materials and Methods

This systematic literature review was performed and reported following the Preferred Reporting Items for Systematic Reviews and Meta-Analyses (PRISMA) guidelines [21]. A systematic review protocol was pre-registered and approved by the International Prospective Register of Systematic Reviews (PROSPERO), ID PROSPERO2022 CRD42022298901. PubMed, Web of Science, and Scopus databases were searched in April 2023, with no search restrictions, using defined search terms (see Supplementary Materials for details) via the University of Liverpool central library systems. A university subject librarian was consulted to develop a transparent and robust search strategy. This review included published articles from cohort, case–control, cross-sectional, registry, case series (with multiple patients), and randomised controlled trials on patients diagnosed with JIA, jSLE, and JDM. Non-primary research articles, including reviews and editorials, were excluded from the analysis, but were used to identify any relevant articles that may have been missed during the initial screening. Non-English language articles were excluded. Articles on JIA, jSLE and JDM that mentioned cases and the prevalence or incidence rate of at least one comorbidity were considered for eligibility. Studies that considered only subsets of JIA, jSLE, and JDM patients, e.g., JIA patients with uveitis or jSLE patients with lupus nephritis, were excluded from analysis, as inclusion would likely overestimate the prevalence of ocular and renal comorbidities, respectively, in examples given. However, studies that considered patients with all subtypes of JIA apart from systemic-onset JIA were included, as these studies were felt to represent most patients with JIA.

Two independent reviewers (SS and SC) screened the titles and abstracts, identified duplicate articles using bibliographic software, assessed full texts for eligibility and performed data extraction. Conflicts were resolved through discussion and moderation from a third reviewer (LJM or DMH). Articles were considered that reported study enrolment age as either ≤18 years, >18 years, or both. Sample size, disease duration, follow-up time, disease onset age, diagnosis age, study enrolment age, participant ethnicities, and data sources (if available) were extracted from each article. Comorbidity prevalence and incidence were calculated based on the available information. For control groups of patients without JIA, jSLE and JDM, selected studies that reported estimates, e.g., hazard ratios, odds ratios, risk ratios, incidence rate ratios, prevalence ratios, and percentage differences (absolute), including 95% confidence intervals (CIs) and *p*-values, were extracted for comparison.

The quality and risk of bias of the included studies were assessed using an adapted version of the Newcastle–Ottawa Scale (NOS). A higher score indicates better methodological quality and a lower risk of bias for the study.

For a comorbidity reported in at least three studies, we performed a meta-analysis to estimate the pooled prevalence, 95% CI, and *I^2^* statistic using the random-effects (DerSimonian–Laird) model. We used the inverse-variance method with logit transformation. The *I^2^* statistic explained heterogeneity among studies [22]. Forest plots were created to visualise the variation in prevalence across the studies, and Funnel plots were used to assess the risk of publication bias. A meta-analysis was performed using the statistical programming software R, version 4.1.1.

## 3. Results

**Figure 1:**
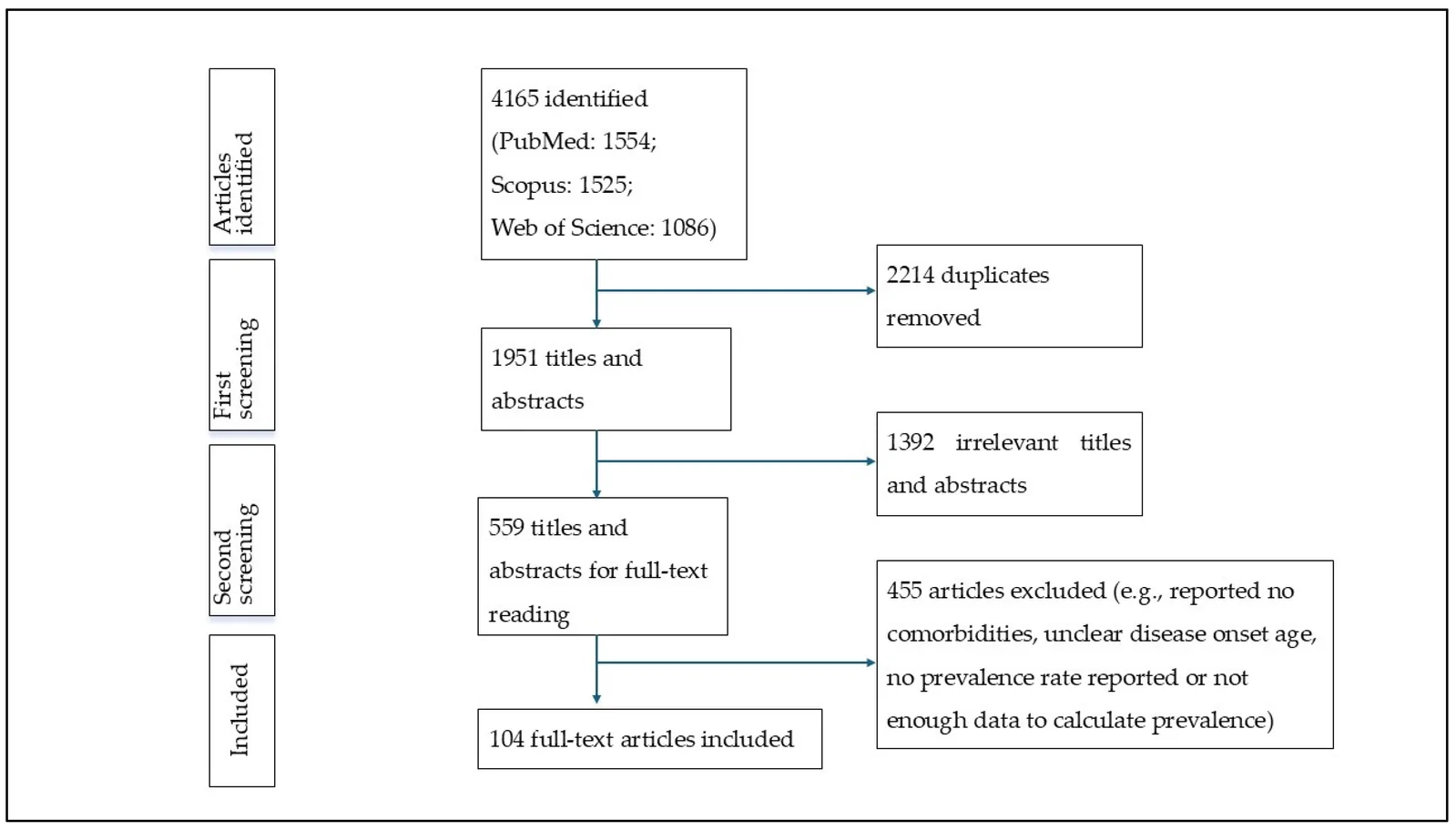
Flowchart showing the study selection process and number of publications identified.

A systematic search returned 4165 publications, of which 104 were included in the review after exclusions, as illustrated in Figure 1.

### 3.1. Aim 1: Identification and Description of Published Comorbidities of JIA, jSLE and JDM

Among 104 articles, 76 reported co-existing comorbidities presenting in childhood and 30 reported co-existing comorbidities apparent in adulthood (Table 1 and Supplementary Tables S1 and S2). Of them, 51 articles included 124,080 patients with JIA [14,23,24,25,26,27,28,29,30,31,32,33,34,35,36,37,38,39,40,41,42,43,44,45,46,47,48,49,50,51,52,53,54,55,56,57,58,59,60,61,62,63,64,65,66,67,68,69,70,71,72], 36 articles considering 9263 patients with jSLE [4,12,73,74,75,76,77,78,79,80,81,82,83,84,85,86,87,88,89,90,91,92,93,94,95,96,97,98,99,100,101,102,103,104,105,106] and 17 articles describing 2729 patients with JDM [1,2,5,107,108,109,110,111,112,113,114,115,116,117,118,119,120], totalling 136,072 patients (Table 1). Simon et al. [41] reported co-existing comorbidities for JIA presenting in both childhood and apparent in adulthood, which were considered separately for this systematic review. Similarly, Sinicato et al. reported comorbidities presenting in both childhood and adulthood [94].

Thirty-three studies scored 5 out of a potential 6 NOS scores. Among them, twenty-five studies reported comorbidities presenting in childhood and seven studies reported comorbidities apparent in adulthood (Table 1, Supplementary Tables S3–S5 and Figures S1–S3). Most studies scored between 3 and 5 on the NOS. Eleven studies scored 6, which may indicate better methodological quality. The comorbidities reported in the most articles were uveitis, diabetes (undefined as type 1 or type 2), and depression in patients with JIA; vascular necrosis, diabetes (undefined), and end-stage renal disease in patients with jSLE; and calcinosis, diabetes (undefined), and cataracts in patients with JDM (Supplementary Figure S4).

### 3.2. Comorbidities’ Pooled Prevalence

Any comorbidity reported in at least three separate studies, either presenting in childhood or apparent in adulthood, associated with JIA, jSLE and JDM, were included in a meta-analysis to estimate the pooled prevalence (Supplementary Figure S4 and Table S1). Among JIA patients, 24 comorbidities were reported in at least three studies each presenting in childhood and 10 were reported in at least three studies appearing in adulthood. For jSLE patients, 18 comorbidities were reported in at least three studies each presenting in childhood and 12 were reported in at least three studies appearing in adulthood. In JDM patients, 10 comorbidities were reported in at least three separate studies presenting in childhood and 2 comorbidities apparent in adulthood (Supplementary Table S6).

**Figure 2:**
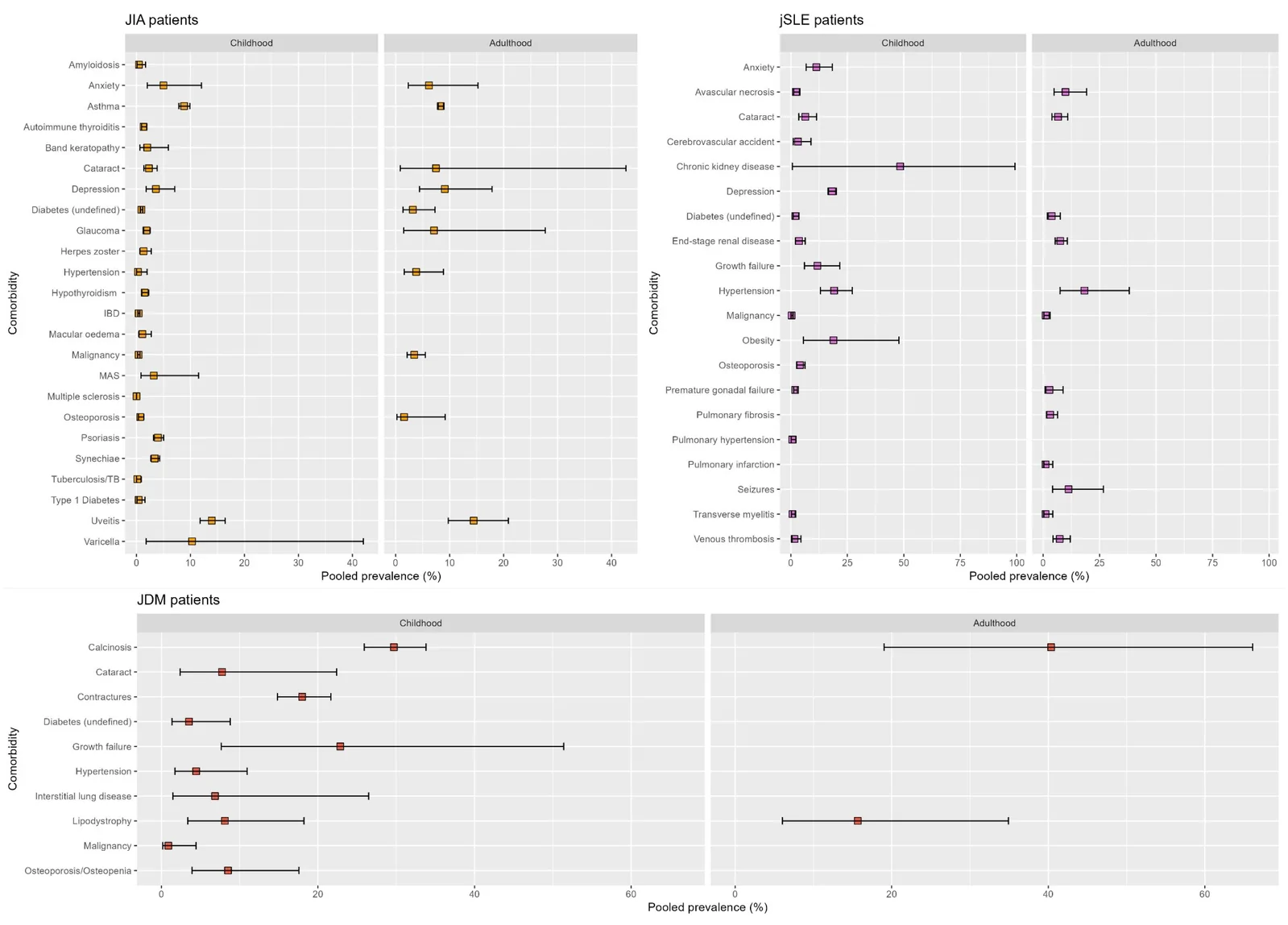
Pooled prevalence (%) and confidence intervals (95%) of comorbidities reported in JIA, jSLE and JDM patients present in childhood or apparent in adulthood. Rectangles represent the pooled prevalence of the comorbidity (%); the line represents the 95% CI. Abbreviations—MAS: macrophage activation syndrome; IBD: inflammatory bowel disease.

Based on estimates of pooled prevalence (Table 2 and Figure 2), uveitis (13.95%, 95% CI 11.79–16.43, range of published values 7–22%), varicella (10.31%, 95% CI 1.79–42.04, range 1–50%) and asthma (8.84%, 95% CI 7.86–9.93, range 8–10%) were highly prevalent comorbidities reported presenting in childhood in patients with JIA. However, the wide confidence intervals and high heterogeneity mean these estimates (and those following) should be interpreted with caution and should not be considered precise prevalence estimates. Uveitis (14.46%, 95% CI 9.76–20.90, range 4–22%), depression (9.11%, 95% CI 4.42–17.87, range 2–25%) and asthma (8.36%, 95% CI 7.88–8.88, range 8–9%) were highly prevalent in adults who had JIA as a child. For jSLE patients, the most prevalent comorbidities were chronic kidney disease (48.38%, 95% CI 0.67–99.24, range 5–100%), hypertension (19.14%, 95% CI 13.05–27.18, range 9–45%) and obesity (18.86%, 95% CI 5.56–47.84, range 7–45%) reported as presenting in childhood; and hypertension (18.30%, 95% CI 7.52–38.16, range 7–44%), seizures (11.24%, 95% CI 4.22–26.68, range 4–23%) and avascular necrosis (9.90%, 95% CI 4.82–19.28, range 3–18%) reported as being apparent in adulthood. Among JDM patients, calcinosis (29.70%, 95% CI 25.91–33.81, range 6–42%), growth failure (22.85%, 95% CI 7.66–51.39, range 8–40%) and contractures (17.98%, 95% CI 14.82–21.64, range 3–20%) had the highest prevalence presenting in childhood; and calcinosis (40.37%, 95% CI 19.02–66.11, range 21–69%) and lipodystrophy (15.67%, 95% CI 6.05–34.91, range 7–33%) were the most prevalent comorbidities apparent in adulthood (Supplementary Table S6). We recognise that many of the items recognised as comorbidities in this pooled prevalence overlap with what are considered activity or damage items related to disease, such as chronic kidney disease in SLE and calcinosis in JDM.

### 3.3. Aim 2: Common Comorbidities Apparent in Both Childhood and Adulthood

There were 10 comorbidities reported by at least three studies as apparent in both childhood and adulthood in JIA patients, 9 in jSLE patients and 2 in JDM patients. (Table 2, Figure 2, and Supplementary Table S6).

Uveitis was a highly prevalent comorbidity among JIA patients, present in both childhood (13.95%, 95% CI 11.79–16.43, range of published values 7–22%) and adulthood (14.46%, 95% CI 9.76–20.90, range 4–22%), indicating that it remains a problem during adulthood. Similarly, depression, cataracts and hypertension were found to be common problems identified in childhood and adulthood in JIA patients. Hypertension was a highly prevalent comorbidity among jSLE patients, present both in childhood (19.14%, 95% CI 13.05–27.18, range 9–45%) and in adulthood (18.30%, 95% CI 7.52–38.16, range 7–44%). Calcinosis was reported as the most prevalent comorbidity in JDM patients, both in childhood and adulthood (Table 2).

Forest plots were created to visualise variation in the pooled prevalence rates of reported comorbidities among JIA, jSLE, and JDM patients in childhood and adulthood and Funnel plots were generated to assess publication bias in those studies (Supplementary Figures S5–S78).

### 3.4. Aim 3: Comorbidity Comparison Between JIA, jSLE and JDM Patients and Non-JIA, Non-jSLE and Non-JDM Controls

Twenty studies reported comparisons with control cohorts of JIA with non-JIA patients (15 studies) and jSLE with non-jSLE patients (5 studies). No study reported a comparison between JDM and non-JDM patients. Control cohorts varied across studies and included patients with a diagnosis of asthma or Attention Deficit Hyperactivity Disorder (ADHD). Control studies reported comorbidity estimates in multiple ways (Table 3). We had hoped to meta-analyse the comparisons of prevalence in cases and controls. However, due to different types of outcomes being reported in different studies (odds ratios, % difference, hazard ratios etc), there were not enough papers reporting the same outcome for the same disease and so, a meta-analysis was not possible. All comorbidity estimates from the studies that compared cases to controls are presented in Supplementary Table S2, and the most common comorbidity estimates are listed in Table 3. Most studies that compared estimates of comorbidity prevalence with control groups either did not reach a statistically significant level or were not reported as statistically significant/insignificant. An exception to this was two studies reporting a statistically significant increase in the presence of type 1 diabetes in JIA patients when compared to non-JIA controls (*p*-value < 0.001) [33,47].

## 4. Discussion

This is the first systematic review to identify and examine a comprehensive list of comorbidities associated with JIA, jSLE, and JDM patients reported in childhood and adulthood. When comorbidities were compared between children with childhood-onset rheumatic disease and controls, often no statistically significant differences were observed. When such differences were observed, the comorbidity was almost always more common in the rheumatic cohorts than in controls. The most common comorbidities reported for JIA patients in this systematic review were uveitis, varicella, depression and asthma. Comorbidities with high point estimates of pooled prevalence in jSLE patients were chronic kidney disease, hypertension, obesity and seizures, whereas JDM patients were most affected by calcinosis, growth failure, contractures and lipodystrophy.

Causes of the higher rate of comorbidities in patients with childhood-onset rheumatic diseases compared to patients without rheumatic diseases are likely multifactorial. Disease activity itself is strongly associated with comorbidities, such as uveitis in JIA and calcinosis in JDM [121,122]. Damage accrual may be a particular risk in childhood or adolescent-onset disease compared to adult-onset disease, as demonstrated in studies of jSLE, where damage frequencies are reported in 28–60% of patients and long-term organ damage is an important determinant of morbidity and mortality [7,123,124]. In studies investigating prognostic factors and damage accrual in jSLE patients, the presence of haemolytic anaemia, renal disease and a higher median disease activity over time at diagnosis were found to increase the risk of damage, with risk of damage being particularly high (probability increasing to 98.97%) when a combination of haemolytic anaemia, neurological disease and vasculitis were present at diagnosis [120,123,124]. The identification of clinical features that predispose to damage may allow for better risk stratification in clinical practice to try to minimise this risk. Further work is also needed to confirm whether childhood-onset disease presents a greater risk of comorbidities than adulthood-onset disease.

The following commonly identified comorbidities are considered in greater detail: uveitis, hypertension, growth failure, depression and anxiety.

### 4.1. Uveitis

Uveitis and associated ocular pathologies are commonly recognised complications of JIA [26,40,45,49,51,52]. This review identified a high frequency of ocular-related comorbidities, including band keratopathy, cataract, glaucoma, macular oedema and synechiae (Figure 2 and Supplementary Table S1).

The prevalence of JIA-associated uveitis has been found to vary according to geographical region and is highest in northern Europe (19.1%) and lowest in Southeast Asia (5%) [121]. These differences may relate to genetic determinants, environmental factors, and/or disparities in the availability of ophthalmological screening. As shown in Table 1, studies included in this systematic review are frequently from Europe or North America, which may not reflect the frequency of comorbidities in other regions due to genetic or ethnic differences. Reported incidence may be altered by sample sizes, disparities in access to care or health services, and race or ethnicity.

### 4.2. Hypertension

This review consistently identified increased risk of hypertension in patients with jSLE and JIA, with the effect size being greatest in patients with jSLE. As disease control in childhood has improved, atherosclerotic cardiovascular disease in juvenile-onset rheumatic disease has emerged as a leading cause of morbidity and mortality in patients in adulthood [125,126]. Hypertension represents an important target for monitoring and early intervention, including modification of lifestyle factors.

### 4.3. Growth Failure

Growth failure in JDM patients was identified as a comorbidity in this systematic review. This could be secondary to disease activity or medication (corticosteroid treatment). Patients with recent onset of puberty during the active phase of disease or with previous growth failure have been found to be most at risk of growth and pubertal delay [127]. Similarly, the peri-pubertal period has been identified as a vulnerable time for growth disruption in JIA and jSLE [128,129].

### 4.4. Depression and Anxiety

Symptoms of depression and anxiety are prevalent in patients with JIA, jSLE and JDM and impose a significant burden with a negative impact on quality of life, but current service provision is sub-optimal to screen and proactively treat mental health issues related to rheumatic conditions [3,53,130,131,132].

Reported contributing factors include pain and medication-related side effects, as well as social isolation and concerns about future prospects (particularly in relation to education, employment, and pregnancy). From focus groups, we have determined that young people with rheumatic diseases consistently identified mental health as a priority comorbidity [13]. A high prevalence of anxiety and depression was found in a large-scale outpatient screening of adolescents and young adults with JIA in Germany [8]. We recommend early and frequent screening of symptoms and a multidisciplinary approach to treatment, as is also recommended in recently published guidance statements by the American College of Rheumatology [133].

### 4.5. Limitations

The limited number of studies reporting on each comorbidity, differences in the definitions of each disease comorbidity and the variance in reported estimates of prevalence/incidence present limitations to the findings of this review. Many of the comorbidities identified overlap with items included in disease damage indices, particularly in jSLE (e.g., chronic kidney disease) and JDM (e.g., calcinosis). As a result, standardised reporting of these conditions may lead to their overrepresentation in the data. Some apparent comorbidities may represent true coexisting conditions, whereas others may arise as consequences of disease-related damage or treatment-related adverse effects. In many cases, it is not possible to attribute an individual condition to a single underlying cause. For example, arterial hypertension in jSLE may reflect disease-related damage, treatment effects, or a coexisting condition. Given these challenges, we adopted an inclusive approach to the definition of comorbidity in this review.

Challenges related to studying the long-term outcomes of childhood-onset rheumatic disease may lead to the underreporting of comorbidities in this population. This might arise from difficulties in capturing long-term data in registries and cohort studies, including discrepancies in disease terminology or classification when patients transition from paediatric to adult care, and from poor linkage between childhood and adult registries [18,19,20]. Inconsistencies in patient identifiers, differences across digital platforms, and poor interoperability among UK health registry systems make it difficult to capture disease data as patients transition into adulthood [18,19]. Studies reporting comorbidities for adult populations did not include information on whether patients were under or over the age of 18 when diagnosed with the comorbid condition, so we have used the terminology “diagnosed in childhood” and “apparent in adulthood” to reflect this. This lack of detail also meant we could not perform our initially planned comparison of comorbidity prevalence in children and adults. Furthermore, the overlapping of large-scale registry cohorts in multiple studies may lead to the over-reporting of co-morbidities. In this review, the BIKER registry was used by a number of authors. These were mostly reporting the prevalence of different comorbidities (e.g., [31,35,46,48]). Two papers report numbers of patients with macrophage activation syndrome and tuberculosis from potentially overlapping cohorts [36,43]. This may lead to some over-reporting of comorbidity prevalence, but both studies are reported in this review. Failure to identify comorbidities early may accelerate disease progression, increase pain and lead to missed opportunities for treatment and prevention, thereby impeding quality of life in adulthood [19]. If risk factors for damage/comorbidities could be identified at early stages of the disease, clinicians may have the opportunity to closely monitor these patients, thereby potentially reducing the risk of damage/comorbidities [123,124].

Heterogeneity was substantial for most pooled estimates, with I^2^ >75% for 22/44 meta-analyses. The highest heterogeneity was observed for chronic kidney disease in jSLE childhood [I^2^ = 98.7%], likely due to differences in definitions of CKD, ethnicity, and follow-up duration across the three included studies. Clinical heterogeneity in CKD and other identified comorbidities was driven by several broader factors. First, geographic variation in study populations is likely to have contributed through differences in genetic susceptibility, environmental exposures, healthcare systems, and clinical management, all of which may influence both disease severity and the occurrence or detection of comorbidities. Second, studies differed in the disease subtypes included, with some including all patients with a disease diagnosis and others including only those that met certain classification criteria. Third, the diagnostic criteria evolved over the study period, with more recent classification criteria enabling earlier diagnosis and potentially capturing patients with different disease characteristics compared with historical cohorts.

Certain populations/comorbidities may not be captured in this review due to the exclusion of non-English publications and the absence of EMBASE in the literature search. Orthopaedic manifestations and musculoskeletal comorbidities were not specifically evaluated, as these outcomes are frequently captured in dedicated orthopaedic databases or registries rather than in paediatric or adult rheumatology registries. Consequently, the long-term burden of childhood-onset rheumatic diseases may be underestimated. These manifestations differ across the major paediatric rheumatic diseases included in this review and may include joint deformities, contractures, growth disturbances, limb-length discrepancies, and temporomandibular joint involvement in JIA, osteoporosis, fragility fractures, avascular necrosis, and non-erosive deforming arthropathy in jSLE and contractures and persistent functional impairment in JDM. Our report summarises the available evidence until April 2023.

## 5. Conclusions

This systematic review found that comorbidities are highly prevalent among JIA, jSLE and JDM patients, particularly uveitis, hypertension and growth failure. These findings indicate a substantial additional disease burden with lasting impacts on quality of life across the life course, extending beyond the primary condition. They underscore the potential of early identification of comorbidities and targeted lifestyle interventions to mitigate risk while recognising that coexisting conditions may complicate management of the primary disease, including influencing treatment selection and outcomes. A multidisciplinary, life-course oriented approach is necessary to support the comprehensive identification and management of comorbidities in juvenile rheumatic disease.

## Figures and Tables

**Table 1:** Details of study participant numbers, geographical location and Newcastle–Ottawa Score of studies on JIA, jSLE and JDM patients observed in childhood and adulthood ^1,2^.

	Number of Studies Reporting Comorbidities								Total
	Childhood				Adulthood				
	JIA	jSLE	JDM	Subtotal	JIA	jSLE	JDM	Subtotal	
Number of studies	35 ^#^	29	12	**76**	17 ^#^	8 ^#^	5	**30**	**106** ^#^
Number of study participants	104,006	8623	2433	**115,062**	20,074	640	296	**21,010**	**136,072**
**Number of studies by region**									
Africa	1	3	1	**5**	0	0	0	**0**	**5**
Asia/Middle East	4	8	4	**16**	3	2	0	**5**	**21**
Australia/New Zealand	0	1	1	**2**	0	0	0	**0**	**2**
Europe/UK	21	3	3	**27**	10	3	4	**17**	**44**
North America	9	8	3	**20**	4	1	1	**6**	**26**
South America	0	6	0	**6**	0	1	0	**1**	**7**
**Newcastle–Ottawa Scale score of bias by number of studies**									
Scores = 6	3	1	1	**5**	2	2	2	**6**	**11**
Scores = 5	9	13	4	**26**	3	2	3	**8**	**34**
Scores = 4	8	8	3	**19**	7	2	0	**9**	**28**
Scores = 3	8	3	2	**13**	2	0	0	**2**	**15**
Scores = 2	5	2	1	**8**	1	0	0	**1**	**9**
Scores = 1	2	2	1	**5**	2	1	1	**4**	**9**

^1^ NOS: Newcastle–Ottawa Scale. Each article was assessed based on the score as representativeness: 2 for population or primary care level data, 1 for hospital, 0 for more selective, i.e., treatments; sample size: 1 if justified, 0 if not; JIA/jSLE/JDM definitions (exposure): 2 for classical criteria/physician diagnosis, 1 for diagnostic codes, and 0 if self-reported/unclear/unreported; and ascertainment of comorbidities (outcome): 1 if diagnostic codes/classical criteria, 0 if self-reported/unclear/unreported. A higher score indicated better methodological quality and a lower risk of bias for the study, and vice versa. ^2^ Forty-four studies were based on UK and European populations, 26 USA/Canada, 21 Asia/Middle East, 7 South American, 5 African and 2 Australian/New Zealand populations. ^#^ Simon et al. [41] reported comorbidities both presenting in childhood and apparent in adulthood and these were considered separately for this systematic review. Sinicato et al. [94] reported comorbidities present in childhood and apparent in adulthood for jSLE.

**Table 2:** Prevalence estimates for comorbidity in JIA, jSLE and JDM patients presenting in childhood and apparent in adulthood. Only comorbidities that were reported in at least 3 papers for both childhood and adult studies are included in this table. The *p* values report Cochran’s Q test of heterogeneity.

		Comorbidity Reported Presenting in Childhood	Comorbidity Reported as Apparent in Adulthood
Disease	Comorbidity	% Pooled Prevalence; (95% CI)	% Pooled Prevalence; (95% CI)
**JIA**	Uveitis	13.95; [11.79–16.43] I^2^ = 91.5% [88.0–93.9], τ^2^ = 0.1497, *p* < 0.001	14.46; [9.76–20.90] I^2^ = 89.1% [79.9–94.0], τ^2^ = 0.299, *p* < 0.001
	Depression	3.61; [1.80–7.11] I^2^ = 97.3% [96.1–98.1], τ^2^ = 1.049, *p* < 0.001	9.11; [4.42–17.87] I^2^ = 85.8% [68.9–93.5], τ^2^ = 0.680, *p* < 0.001
	Asthma	8.84; [7.86–9.93] I^2^ = 88.7% [69.0–95.9], τ^2^ = 0.0115, *p* < 0.001	8.36; [7.88–8.88] I^2^ = 0% [0.0–89.6], τ^2^ = 0, *p* = 0.575
	Cataract	2.32; [1.39–3.85] I^2^ = 63.0% [16.0–83.7], τ^2^ = 0.295, *p* = 0.013	7.48; [0.87–42.66] I^2^ = 95.7% [90.6–98.0], τ^2^ = 3.707, *p* < 0.001
	Glaucoma	1.84; [1.33–2.56] I^2^ = 1.9% [0.0–79.6], τ^2^ = 0, *p* = 0.396	7.11; [1.50–27.74] I^2^ = 96.0% [91.5–98.1], τ^2^ = 1.937, *p* < 0.001
	Hypertension	0.29; [0.04–1.97] I^2^ = 95.8% [92.7–97.6], τ^2^ = 4.498, *p* < 0.001	3.81; [1.59–8.85] I^2^ = 96.2% [93.4–97.8], τ^2^ = 0.911, *p* < 0.001
	Anxiety	5.02; [2.00–12.06], I^2^ = 98.8% [98.2–99.2], τ^2^ = 0.889, *p* < 0.001	6.18; [2.36–15.25] I^2^ = 89.1% [70.3–96.0], τ^2^ = 0.743, *p* < 0.001
	Malignancy	0.40; [0.23–0.68] I^2^ = 85.6% [64.7–94.2], τ^2^ = 0.252, *p* < 0.001	3.45; [2.14–5.53] I^2^ = 93.2% [83.6–97.2], τ^2^ = 0135, *p* < 0.001
	Diabetes (undefined)	0.92; [0.72–1.18] I^2^ = 55.0% [0.4–79.6], τ^2^ = 0.053, *p* = 0.030	3.19; [1.36–7.31] I^2^ = 81.4% [60.1–91.3], τ^2^ = 0.845, *p* < 0.001
	Osteoporosis	3.99; [3.16–05.03] I^2^ = 0.0% [0.0–89.6], τ^2^ = 0, *p* = 0.455	1.58; [0.26–9.18] I^2^ = 94.4% [87.1–97.6], τ^2^ = 2.489, *p* < 0.001
**jSLE**	Hypertension	19.14; [13.05–27.18] I^2^ = 85.8% [74.0–92.3], τ^2^ = 0.352, *p* < 0.001	18.30; [7.52–38.16] I^2^ = 92.4% [83.8–96.5], τ^2^ = 0.931, *p* < 0.001
	Avascular necrosis	2.20; [1.20–4.02] I^2^ = 76.4% [52.8–88.2], τ^2^ = 0.486, *p* < 0.001	9.90; [4.82–19.28] I^2^ = 69.6% [12.4–89.4], τ^2^ = 0.431, *p* = 0.020
	End-stage renal disease	3.62; [2.03–6.39] I^2^ = 78.0% [54.3–89.4], τ^2^ = 0.431, *p* < 0.001	7.59; [5.31–10.75] I^2^ = 20.0% [0.0–87.8], τ^2^ = 0, *p* = 0.290
	Venous thrombosis	1.61; [0.57–4.44] I^2^ = 61.3% [0.0–89.0], τ^2^ = 0.498, *p* = 0.075	7.35; [4.39–12.04] I^2^ = 43.5% [0.0–83.1], τ^2^ = 0, *p* = 0.170
	Cataract	6.39; [3.49–11.41] I^2^ = 87.8% [75.9–93.8], τ^2^ = 0.495, *p* < 0.001	6.62; [3.95–10.87] I^2^ = 0% [0.0–89.6], τ^2^ = 0, *p* = 0.410
	Diabetes (undefined)	1.98; [1.07–3.62] I^2^ = 55.5% [1.9–79.9], τ^2^ = 0.376, *p* = 0.028	3.82; [1.88–7.58] I^2^ = 39.2% [0.0–79.3], τ^2^ = 0.213, *p* = 0.177
	Premature gonadal failure	1.92; [1.27–2.90] I^2^ = 0% [0.0–89.6], τ^2^ = 0, *p* = 0.622	2.83; [0.87–8.82] I^2^ = 39.5% [0.0–81.3], τ^2^ = 0.458, *p* = 0.192
	Malignancy	0.33; [0.11–0.93] I^2^ = 0% [0.0–84.7], τ^2^ = 0, *p* = 0.661	1.12; [0.39–3.14] I^2^ = 0% [0.0–89.6], τ^2^ = 0, *p* = 0.576
	Transverse myelitis	0.73; [0.28–1.93] I^2^ = 52.7% [0.0–86.4], τ^2^ = 0.395, *p* = 0.121	1.09; [0.27–4.27] I^2^ = 0% [0.0–89.6], τ^2^ = 0, *p* = 0.624
**JDM**	Calcinosis	29.70; [25.91–33.81] I^2^ = 26.7% [0.0–63.7], τ^2^ = 0.015, *p* = 0.190	40.37; [19.02–66.11] I^2^ = 92.7% [84.6–96.6], τ^2^ = 1.080, *p* < 0.001
	Lipodystrophy	8.11; [3.38–18.21] I^2^ = 57.7% [0.0–85.9], τ^2^ = 0.513, *p* = 0.069	15.67; [6.05–34.91] I^2^ = 85.1% [55.9–94.9], τ^2^ = 0.733, *p* = 0.001

**Table 3:** Most common comparative comorbidity estimates between cases and controls of JIA and jSLE patients.

	Childhood				Adulthood			
Patient Group	Comorbidity	Study	Estimates	Estimate Type	Comorbidity	Study	Estimates	Estimate Type
JIA	Anxiety	Berthold et al. [53]	F 1.20, M 0.60	Hazard ratio	Asthma	Raab et al. [59]	−2.50	% difference (absolute)
	Depression	Berthold et al. [53]	F 1.10, M 0.80	Hazard ratio	Depression	Raab et al. [59]	−4.10	
	Type 1 diabetes	Schenck et al. [33]	1.76	Prevalence ratio	Type 1 diabetes	Simon et al. [41]	2.15, 2.50	Odds ratio
		Lee et al. [38]	1.81, 1.48	Hazard ratio (adj)	Diabetes mellitus	Raab et al. [59]	−0.70	% difference (absolute)
		Lee et al. [38]	1.89, 1.46	Hazard ratio (adjusted for age, sex, index date)	Gestational diabetes	Zhang-Jian et al. [71]	0.75	Adjusted Odds ratio
		Lovell et al. [47]	1.25	Log risk ratio	Hypertension	Raab et al. [59]	−0.60	% difference (absolute)
		Simon et al. [41]	1.82, 1.70	Odds ratio	Interstitial lung disease	Simon et al. [41]	12.89, 11.54	Odds ratios
	Type 2 diabetes	Lee et al. [38]	1.22, 1.01	Hazard ratio (adjusted)	Multiple sclerosis	Simon et al. [41]	1.52, 1.50	
		Lee et al. [38]	1.89, 0.95	Hazard ratio (age, sex, index date)	Osteoporosis (lumbar spine)	Aggarwal et al. [60]	22.40	% difference (absolute)
	Malignancy	Horneff et al. [46]	3.00, 6.30	Risk ratio	Osteoporosis (hip)	Aggarwal et al. [60]	5.90	
	Multiple sclerosis	Lovell et al. [47]	1.90	Log risk ratio				
		Simon et al. [41]	3.38, 2.26	Odds ratio	Psoriasis	Simon et al. [41]	2.93, 2.31	Odds ratios
	Interstitial lung disease	Simon et al. [41]	12.03, 24.86	Odds ratio	Ulcerative colitis	Simon et al. [41]	2.41, 3.69	
	Psoriasis	Simon et al. [41]	5.89, 8.42	Odds ratio	Uveitis	Simon et al. [41]	29.54, 18.46	
	Ulcerative colitis	Simon et al. [41]	6.49, 8.34	Odds ratio				
	Uveitis	Simon et al. [41]	133.20, 165.10	Odds ratio				
jSLE	Asthma	Karve et al. [12]	8.50	% difference (absolute)	Hypertension	Sinicato et al. [94]	11.40	% difference (absolute)
	Type 1 diabetes	Karve et al. [12]	−0.70					
	Multiple sclerosis	Karve et al. [12]	1.40					
	Hypertension	Sinicato et al. [94]	9.80	% difference (absolute)				

Simon: Matched controls patients with ADHD in the US PharMetrics and US MarketScan datasets, respectively. Lee: Controls were healthy children with at least one record of a Wellcare visit, matched with JIA patients on age, sex and index date; and children with at least two diagnoses of asthma followed by at least one outpatient pharmacy claim for asthma treatment, matched with JIA patients on age, sex and index date, respectively. Horneff: Compared with rates taken from the German Childhood Cancer Registry report 2015 (Methotrexate Drug Cohort) and with rates taken from the German Childhood Cancer Registry report 2015 (Etanercept Drug Cohort), respectively.

## Data Availability

The original contributions presented in this study are included in the article/Supplementary Materials. Further inquiries can be directed to the corresponding author.

## Supplementary Materials

The following supporting information can be downloaded at https://www.mdpi.com/article/10.3390/children13080995/s1, Search Strategy, Supplementary Figures S1–S78, Supplementary Tables S1–S6.

## References

[1] Huber A.M., Lang B., LeBlanc C.M., Birdi N., Bolaria R.K., Malleson P., MacNeil I., Momy J.A., Avery G., Feldman B.M. (2000). Medium- and long-term functional outcomes in a multicenter cohort of children with juvenile dermatomyositis. Arthritis Rheum..

[2] Ravelli A., Trail L., Ferrari C., Ruperto N., Pistorio A., Pilkington C., Maillard S., Oliveira S.K., Sztajnbok F., Cuttica R. (2010). Long-term outcome and prognostic factors of juvenile dermatomyositis: A multinational, multicenter study of 490 patients. Arthritis Care Res..

[3] Fair D.C., Rodriguez M., Knight A.M., Rubinstein T.B. (2019). Depression and Anxiety in Patients with Juvenile Idiopathic Arthritis: Current Insights and Impact on Quality of Life, A Systematic Review. Open Access Rheumatol..

[4] Groot N., Shaikhani D., Teng Y.K.O., de Leeuw K., Bijl M., Dolhain R., Zirkzee E., Fritsch-Stork R., Bultink I.E.M., Kamphuis S. (2019). Long-Term Clinical Outcomes in a Cohort of Adults with Childhood-Onset Systemic Lupus Erythematosus. Arthritis Rheumatol..

[5] Tsaltskan V., Aldous A., Serafi S., Yakovleva A., Sami H., Mamyrova G., Targoff I.N., Schiffenbauer A., Miller F.W., Simmens S.J. (2020). Long-term outcomes in Juvenile Myositis patients. Semin. Arthritis Rheum..

[6] Glerup M., Rypdal V., Arnstad E.D., Ekelund M., Peltoniemi S., Aalto K., Rygg M., Toftedal P., Nielsen S., Fasth A. (2020). Long-Term Outcomes in Juvenile Idiopathic Arthritis: Eighteen Years of Follow-Up in the Population-Based Nordic Juvenile Idiopathic Arthritis Cohort. Arthritis Care Res..

[7] Tucker L.B., Uribe A.G., Fernández M., Vilá L.M., McGwin G., Apte M., Fessler B.J., Bastian H.M., Reveille J.D., Alarcón G.S. (2008). Adolescent onset of lupus results in more aggressive disease and worse outcomes: Results of a nested matched case–control study within LUMINA, a multiethnic US cohort (LUMINA LVII). Lupus.

[8] Milatz F., Albrecht K., Minden K., Marschall U., Klotsche J., Callhoff J. (2024). Mental comorbidities in adolescents and young adults with juvenile idiopathic arthritis: An analysis of German nationwide health insurance data. Pediatr. Rheumatol. Online J..

[9] Livermore P., Gray S., Mulligan K., Stinson J.N., Wedderburn L.R., Gibson F. (2019). Being on the juvenile dermatomyositis rollercoaster: A qualitative study. Pediatr. Rheumatol. Online J..

[10] Fawole O.A., Reed M.V., Harris J.G., Hersh A., Rodriguez M., Onel K., Lawson E., Rubinstein T., Ardalan K., Morgan E. (2021). Engaging patients and parents to improve mental health intervention for youth with rheumatological disease. Pediatr. Rheumatol. Online J..

[11] Fortuna-Reyna B.J., Pelaez-Ballestas I., Garcia-Rodriguez F., Faugier-Fuentes E., Mendieta-Zeron S., Villarreal-Trevino A.V., Rosiles-De la Garza S.G., Reyes-Cordero G., Jimenez-Hernandez S., Guadarrama-Orozco J.H. (2021). Psychosocial and economic impact of rheumatic diseases on caregivers of Mexican children. Pediatr. Rheumatol. Online J..

[12] Karve S., Candrilli S., Kappelman M.D., Tolleson-Rinehart S., Tennis P., Andrews E. (2012). Healthcare utilization and comorbidity burden among children and young adults in the United States with systemic lupus erythematosus or inflammatory bowel disease. J. Pediatr..

[13] Siddiq S., Ainsworth J.S., Pain C.E., Smith E.M.D., Zhao S.S., Hughes D.M., McCann L.J. (2025). Involving young people in research investigating comorbidity associated with childhood-onset rheumatic disease: Perspectives of a series of focus groups. BMC Rheumatol..

[14] Mena-Vázquez N., Ortiz-Marquez F., Cabezudo-García P., Padilla-Leiva C., Díaz-Cordoves Rego G., Muñoz-Becerra L., Ramirez-Garcia T., Lisbona-Montanez J.M., Manrique-Arija S., Mucientes A. (2022). Longitudinal Study of Cognitive Functioning in Adults with Juvenile Idiopathic Arthritis. Biomedicines.

[15] Zhao S.S., Radner H., Siebert S., Duffield S.J., Thong D., Hughes D.M., Moots R.J., Solomon D.H., Goodson N.J. (2019). Comorbidity burden in axial spondyloarthritis: A cluster analysis. Rheumatology.

[16] Clarson L.E., Hider S.L., Belcher J., Heneghan C., Roddy E., Mallen C.D. (2015). Increased risk of vascular disease associated with gout: A retrospective, matched cohort study in the UK clinical practice research datalink. Ann. Rheum. Dis..

[17] Dregan A., Matcham F., Harber-Aschan L., Rayner L., Brailean A., Davis K., Hatch S., Pariante C., Armstrong D., Stewart R. (2019). Common mental disorders within chronic inflammatory disorders: A primary care database prospective investigation. Ann. Rheum. Dis..

[18] Moorthie S., Hayat S., Zhang Y., Parkin K., Philips V., Bale A., Duschinsky R., Ford T., Moore A. (2022). Rapid systematic review to identify key barriers to access, linkage, and use of local authority administrative data for population health research, practice, and policy in the United Kingdom. BMC Public Health.

[19] Hersh A., von Scheven E., Yelin E. (2011). Adult outcomes of childhood-onset rheumatic diseases. Nat. Rev. Rheumatol..

[20] Papadopoulou C., Chew C., Wilkinson M.G.L., McCann L., Wedderburn L.R. (2023). Juvenile idiopathic inflammatory myositis: An update on pathophysiology and clinical care. Nat. Rev. Rheumatol..

[21] Page M.J., McKenzie J.E., Bossuyt P.M., Boutron I., Hoffmann T.C., Mulrow C.D., Shamseer L., Tetzlaff J.M., Akl E.A., Brennan S.E. (2021). The PRISMA 2020 statement: An updated guideline for reporting systematic reviews. BMJ.

[22] Borenstein M., Hedges L.V., Higgins J.P., Rothstein H.R. (2010). A basic introduction to fixed-effect and random-effects models for meta-analysis. Res. Synth. Methods.

[23] Demirkaya E., Ozen S., Bilginer Y., Ayaz N.A., Makay B.B., Unsal E., Erguven M., Poyrazoglu H., Kasapcopur O., Gok F. (2011). The distribution of juvenile idiopathic arthritis in the eastern Mediterranean: Results from the registry of the Turkish Paediatric Rheumatology Association. Clin. Exp. Rheumatol..

[24] Jain V., Singh S., Sharma A. (2001). Keratoconjunctivitis sicca is not uncommon in children with juvenile rheumatoid arthritis. Rheumatol. Int..

[25] Rangel L., Garralda M.E., Hall A., Woodham S. (2003). Psychiatric adjustment in chronic fatigue syndrome of childhood and in juvenile idiopathic arthritis. Psychol. Med..

[26] Saurenmann R.K., Levin A.V., Feldman B.M., Rose J.B., Laxer R.M., Schneider R., Silverman E.D. (2007). Prevalence, risk factors, and outcome of uveitis in juvenile idiopathic arthritis: A long-term followup study. Arthritis Rheum..

[27] Chhabra A., Robinson C., Houghton K., Cabral D.A., Morishita K., Tucker L.B., Petty R.E., Larche M., Batthish M., Guzman J. (2020). Long-term outcomes and disease course of children with juvenile idiopathic arthritis in the ReACCh-Out cohort: A two-centre experience. Rheumatology.

[28] Beukelman T., Xie F., Chen L., Baddley J.W., Delzell E., Grijalva C.G., Lewis J.D., Ouellet-Hellstrom R., Patkar N.M., Saag K.G. (2012). Rates of hospitalized bacterial infection associated with juvenile idiopathic arthritis and its treatment. Arthritis Rheum..

[29] Van Rossum M.A.J., Van Soesbergen R.M., Boers M., Zwinderman A.H., Fiselier T.J.W., Franssen M.J.A.M., Ten Cate R., Van Suijlekom-Smit L.W.A., Wulffraat N.M., Van Luijk W.H.J. (2007). Long-term outcome of juvenile idiopathic arthritis following a placebo-controlled trial: Sustained benefits of early sulfasalazine treatment. Ann. Rheum. Dis..

[30] Gerloni V., Pontikaki I., Gattinara M., Fantini F. (2008). Focus on adverse events of tumour necrosis factor alpha blockade in juvenile idiopathic arthritis in an open monocentric long-term prospective study of 163 patients. Ann. Rheum. Dis..

[31] Klotsche J., Niewerth M., Haas J.P., Huppertz H.I., Zink A., Horneff G., Minden K. (2016). Long-term safety of etanercept and adalimumab compared to Methotrexate in patients with juvenile idiopathic arthritis (JIA). Ann. Rheum. Dis..

[32] Beukelman T., Xie F., Baddley J.W., Chen L., Delzell E., Grijalva C.G., Mannion M.L., Patkar N.M., Saag K.G., Winthrop K.L. (2013). Brief report: Incidence of selected opportunistic infections among children with juvenile idiopathic arthritis. Arthritis Rheum..

[33] Schenck S., Rosenbauer J., Niewerth M., Klotsche J., Minden K., Schwarz T., Foeldvari I., Horneff G., Weller-Heinemann F., Holl R.W. (2018). Comorbidity of Type 1 Diabetes Mellitus in Patients with Juvenile Idiopathic Arthritis. J. Pediatr..

[34] Angeles-Han S.T., McCracken C., Yeh S., Jenkins K., Stryker D., Rouster-Stevens K., Vogler L.B., Lambert S.R., Drews-Botsch C., Prahalad S. (2015). Characteristics of a cohort of children with Juvenile Idiopathic Arthritis and JIA-associated Uveitis. Pediatr. Rheumatol..

[35] Thiele F., Klein A., Windschall D., Hospach A., Foeldvari I., Minden K., Weller-Heinemann F., Horneff G. (2021). Comparative risk of infections among real-world users of biologics for juvenile idiopathic arthritis: Data from the German BIKER registry. Rheumatol. Int..

[36] Kearsley-Fleet L., Klotsche J., van Straalen J.W., Costello W., D'Angelo G., Giancane G., Horneff G., Klein A., Laday M., Lunt M. (2022). Burden of comorbid conditions in children and young people with juvenile idiopathic arthritis: A collaborative analysis of 3 JIA registries. Rheumatology.

[37] Krause M.L., Zamora-Legoff J.A., Crowson C.S., Muskardin T.W., Mason T., Matteson E.L. (2017). Population-based study of outcomes of patients with juvenile idiopathic arthritis (JIA) compared to non-JIA subjects. Semin. Arthritis Rheum..

[38] Lee H.M., Jin Y.Z., Liu J., Cohen E.M., Chen S.K., Kim S.C. (2020). Risk of Diabetes Mellitus in Patients with Juvenile Idiopathic Arthritis. J. Rheumatol..

[39] Horneff G., Borchert J., Heinrich R., Kock S., Klaus P., Dally H., Hagemann C., Diesing J., Schönfelder T. (2022). Incidence, prevalence, and comorbidities of juvenile idiopathic arthritis in Germany: A retrospective observational cohort health claims database study. Pediatr. Rheumatol..

[40] Bolt I.B., Cannizzaro E., Seger R., Saurenmann R.K. (2008). Risk factors and longterm outcome of juvenile idiopathic arthritis-associated uveitis in Switzerland. J. Rheumatol..

[41] Simon T.A., Harikrishnan G.P., Kawabata H., Singhal S., Brunner H.I., Lovell D.J. (2020). Prevalence of co-existing autoimmune disease in juvenile idiopathic arthritis: A cross-sectional study. Pediatr. Rheumatol..

[42] Schulz C., Fuehner S., Schlüter B., Fobker M., Sengler C., Klotsche J., Niewerth M., Minden K., Foell D. (2022). Prevalence of autoantibodies in patients with juvenile idiopathic arthritis: Results from the German inception cohort ICON-JIA. Pediatr. Rheumatol..

[43] Swart J., Giancane G., Horneff G., Magnusson B., Hofer M., Alexeeva Capitalie C., Panaviene V., Bader-Meunier B., Anton J., Nielsen S. (2018). Pharmacovigilance in juvenile idiopathic arthritis patients treated with biologic or synthetic drugs: Combined data of more than 15,000 patients from Pharmachild and national registries. Arthritis Res. Ther..

[44] Giancane G., Muratore V., Marzetti V., Quilis N., Benavente B.S., Bagnasco F., Alongi A., Civino A., Quartulli L., Consolaro A. (2019). Disease activity and damage in juvenile idiopathic arthritis: Methotrexate era versus biologic era. Arthritis Res. Ther..

[45] Grassi A., Corona F., Casellato A., Carnelli V., Bardare M. (2007). Prevalence and Outcome of Juvenile Idiopathic Arthritis-Associated Uveitis and Relation to Articular Disease. J. Rheumatol..

[46] Horneff G., Klein A., Oommen P.T., Hospach A., Feddersen I., Minden K. (2016). Update on malignancies in children with juvenile idiopathic arthritis in the German BIKER Registry. Clin. Exp. Rheumatol..

[47] Lovell D.J., Huang B., Chen C., Angeles-Han S.T., Simon T.A., Brunner H.I. (2021). Prevalence of autoimmune diseases and other associated conditions in children and young adults with juvenile idiopathic arthritis. RMD Open.

[48] Barthel D., Ganser G., Kuester R.M., Onken N., Minden K., Girschick H.J., Hospach A., Horneff G. (2015). Inflammatory bowel disease in juvenile idiopathic arthritis patients treated with biologics. J. Rheumatol..

[49] Sahin S., Acari C., Sonmez H.E., Kilic F.Z., Sag E., Dundar H.A., Adrovic A., Demir S., Barut K., Bilginer Y. (2021). Frequency of juvenile idiopathic arthritis and associated uveitis in pediatric rheumatology clinics in Turkey: A retrospective study, JUPITER. Pediatr. Rheumatol..

[50] Pohjankoski H., Kautiainen H., Kotaniemi K., Korppi M., Savolainen A. (2010). Autoimmune diseases in children with juvenile idiopathic arthritis. Scand. J. Rheumatol..

[51] Çakan M., Ayaz N.A., Karadaǧ S.G., Ekinci D.Y. (2020). Why is the frequency of uveitis low in Turkish children with juvenile idiopathic arthritis?. Rheumatology.

[52] Cosickic A., Halilbasic M., Selimovic A., Avdagic H. (2017). Uveitis Associated with Juvenile Idiopathic Arthritis, our Observations. Med. Arch..

[53] Berthold E., Dahlberg A., Joud A., Tyden H., Mansson B., Kahn F., Kahn R. (2022). The risk of depression and anxiety is not increased in individuals with juvenile idiopathic arthritis—Results from the south-Swedish juvenile idiopathic arthritis cohort. Pediatr. Rheumatol. Online J..

[54] Lien G., Flatø B., Haugen M., Vinje O., Sørskaar D., Dale K., Johnston V., Egeland T., Førre Ø. (2003). Frequency of osteopenia in adolescents with early-onset juvenile idiopathic arthritis: A long-term outcome study of one hundred five patients. Arthritis Rheum..

[55] Saraux A., Benichou J., Guillevin L., Idbrik L., Job-Deslandre C., Soudant M., Wendling D., Guillemin F. (2015). Which patients with rheumatoid arthritis, spondyloarthritis, or juvenile idiopathic arthritis receive TNF-α antagonists in France? The CORPUS cohort study. Clin. Exp. Rheumatol..

[56] Alberdi-Saugstrup M., Enevold C., Zak M., Nielsen S., Nordal E., Berntson L., Fasth A., Rygg M., Müller K. (2017). Non-HLA gene polymorphisms in juvenile idiopathic arthritis: Associations with disease outcome. Scand. J. Rheumatol..

[57] Amine B., Ibn Yacoub Y., Rostom S., Hajjaj-Hassouni N. (2011). Prevalence of overweight among Moroccan children and adolescents with juvenile idiopathic arthritis. Jt. Bone Spine.

[58] Smitherman E.A., Chahine R.A., Bitencourt N., Rahman A., Lawson E.F., Chang J.C. (2023). Patient-Reported Outcomes Among Transition-Age Young Adults with Juvenile Idiopathic Arthritis in the Childhood Arthritis and Rheumatology Research Alliance Registry. J. Rheumatol..

[59] Raab A.C., Sengler M., Niewerth J., Klotsche G., Horneff H., Girschick, Weber K., Minden K. (2013). Comorbidity profiles among adult patients with juvenile idiopathic arthritis: Results of a biologic register. Clin. Exp. Rheumatol..

[60] Aggarwal P., Aggarwal A., Gupta S., Misra R. (2006). Osteopenia Is Common in Adult Male Patients with Active Juvenile Idiopathic Arthritis. J. Rheumatol..

[61] Arvidsson L.Z., Fjeld M.G., Smith H.J., Flatø B., Øgaard B., Larheim T.A. (2010). Craniofacial growth disturbance is related to temporomandibular joint abnormality in patients with juvenile idiopathic arthritis, but normal facial profile was also found at the 27-year follow-up. Scand. J. Rheumatol..

[62] Packham J.C., Hall M.A. (2002). Long-term follow-up of 246 adults with juvenile idiopathic arthritis: Functional outcome. Br. Soc. Rheumatol..

[63] Mars N.J., Kerola A.M., Kauppi M.J., Pirinen M., Elonheimo O., Sokka-Isler T. (2019). Patients with rheumatic diseases share similar patterns of healthcare resource utilization. Scand. J. Rheumatol..

[64] Dimopoulou D., Trachana M., Pratsidou-Gertsi P., Sidiropoulos P., Kanakoudi-Tsakalidou F., Dimitroulas T., Garyfallos A. (2017). Predictors and long-term outcome in Greek adults with juvenile idiopathic arthritis: A 17-year continuous follow-up study. Rheumatology.

[65] Rebane K., Tuomi A.K., Kautiainen H., Peltoniemi S., Glerup M., Aalto K. (2022). Abdominal pain in Finnish young adults with juvenile idiopathic arthritis. Scand. J. Gastroenterol..

[66] Minden K., Horneff G., Niewerth M., Seipelt E., Aringer M., Aries P., Foeldvari I., Haas J.P., Klein A., Tatsis S. (2019). Time of disease-modifying antirheumatic drug start in juvenile idiopathic arthritis and the likelihood of a drug-free remission in young adulthood. Arthritis Care Res..

[67] Smith C.J.F., Förger F., Bandoli G., Chambers C.D. (2019). Factors Associated With Preterm Delivery Among Women With Rheumatoid Arthritis and Women With Juvenile Idiopathic Arthritis. Arthritis Care Res..

[68] Barth S., Haas J.P., Schlichtiger J., Molz J., Bisdorff B., Michels H., Hügle B., Radon K. (2016). Long-term health-related quality of life in german patients with juvenile idiopathic arthritis in comparison to german general population. PLoS ONE.

[69] Packham J.C., Hall M.A. (2003). Premature ovarian failure in women with juvenile idiopathic arthritis (JIA). Clin. Exp. Rheumatol..

[70] Ma K.S.K., Illescas Ralda M.M., Veeravalli J.J., Wang L.T., Thota E., Huang J.Y., Kao C.T., Wei J.C.C., Resnick C.M. (2022). Patients with juvenile idiopathic arthritis are at increased risk for obstructive sleep apnoea: A population-based cohort study. Eur. J. Orthod..

[71] Zhang-Jian S.J., Yang H.Y., Chiu M.J., Chou I.J., Kuo C.F., Huang J.L., Yeh K.W., Wu C.Y. (2020). Pregnancy outcomes and perinatal complications of Asian mothers with juvenile idiopathic arthritis—A case-control registry study. Pediatr. Rheumatol. Online J..

[72] Rypdal V., Glerup M., Songstad N.T., Bertelsen G., Christoffersen T., Arnstad E.D., Aalto K., Berntson L., Fasth A., Herlin T. (2021). Uveitis in Juvenile Idiopathic Arthritis: 18-Year Outcome in the Population-based Nordic Cohort Study. Ophthalmology.

[73] Davis A., Faerber J., Ardalan K., Katcoff H., Klein-Gitelman M., Rubinstein T.B., Cidav Z., Mandell D.S., Knight A. (2023). The Effect of Psychiatric Comorbidity on Healthcare Utilization for Youth with Newly Diagnosed Systemic Lupus Erythematosus. J. Rheumatol..

[74] Garf K.E., Marzouk H., Farag Y., Rasheed L., Garf A.E. (2015). Vitamin D status in Egyptian patients with juvenile-onset systemic lupus erythematosus. Rheumatol. Int..

[75] Paim-Marques L., Carneiro P., Verçosa I.C., Appenzeller S. (2019). Corneal vortex keratopathy in childhood-onset systemic lupus erythematosus (c-SLE). Clin. Rheumatol..

[76] Silva M.F., Ferriani M.P., Terreri M.T., Pereira R.M., Magalhães C.S., Bonfá E., Campos L.M., Okuda E.M., Appenzeller S., Ferriani V.P. (2015). A multicenter study of invasive fungal infections in patients with childhood-onset systemic lupus erythematosus. J. Rheumatol..

[77] Knight A.M., Xie M., Mandell D.S. (2016). Disparities in psychiatric diagnosis and treatment for youth with systemic lupus erythematosus: Analysis of a national us medicaid sample. J. Rheumatol..

[78] das Chagas Medeiros M.M., Campos Bezerra M., Holanda Ferreira Braga F.N., Melo da Justa Feijão M.R., Rodrigues Gois A.C., do Rosário Rebouças V.C., Amorim Zaranza de Carvalho T.M., Solon Carvalho L.N., Mendes Ribeiro A.T. (2016). Clinical and immunological aspects and outcome of a Brazilian cohort of 414 patients with systemic lupus erythematosus (SLE): Comparison between childhood-onset, adult-onset, and late-onset SLE. Lupus.

[79] Frittoli R.B., de Oliveira Peliçari K., Bellini B.S., Marini R., Fernandes P.T., Appenzeller S. (2016). Association between academic performance and cognitive dysfunction in patients with juvenile systemic lupus erythematosus. Rev. Bras. Reumatol..

[80] Holland M.J., Beresford M.W., Feldman B.M., Huggins J., Norambuena X., Silva C.A., Susic G., Sztajnbok F., Uziel Y., Appenzeller S. (2018). Measuring Disease Damage and Its Severity in Childhood-Onset Systemic Lupus Erythematosus. Arthritis Care Res..

[81] Gad G.I., Mohamed S.T., Awwad K.S., Mohamed R.F. (2013). Study of audiovestibular dysfunction in children with systemic lupus erythematosus. Int. J. Pediatr. Otorhinolaryngol..

[82] Jongvilaikasem P., Rianthavorn P. (2021). Longitudinal growth patterns and final height in childhood-onset systemic lupus erythematosus. Eur. J. Pediatr..

[83] AlAhmed O., Sivaraman V., Moore-Clingenpeel M., Ardoin S.P., Bout-Tabaku S. (2020). Autoimmune thyroid diseases, autoimmune hepatitis, celiac disease and type 1 diabetes mellitus in pediatric systemic lupus erythematosus: Results from the CARRA Legacy Registry. Lupus.

[84] Ye Q., Wang G., Lu J., Huang Y., Zhang J., Zhu L., Zhu Y., Lan J., Li Z., Liu Y. (2021). Exposure levels of mycophenolic acid are associated with comorbidities in children with systemic lupus erythematosus. Lupus.

[85] Cann M.P., Sage A.M., McKinnon E., Lee S.J., Tunbridge D., Larkins N.G., Murray K.J. (2022). Childhood Systemic Lupus Erythematosus: Presentation, management and long-term outcomes in an Australian cohort. Lupus.

[86] Medhat B.M., Behiry M.E., Sobhy N., Farag Y., Marzouk H., Mostafa N., Khalifa I., Elkhalifa M., Eissa B.M., Hassan E.H.E. (2020). Late-onset systemic lupus erythematosus: Characteristics and outcome in comparison to juvenile- and adult-onset patients-a multicenter retrospective cohort. Clin. Rheumatol..

[87] Aggarwal A., Phatak S., Srivastava P., Lawrence A., Agarwal V., Misra R. (2018). Outcomes in juvenile onset lupus: Single center cohort from a developing country. Lupus.

[88] Al-Mayouf S.M., Al Sonbul A. (2008). Influence of gender and age of onset on the outcome in children with systemic lupus erythematosus. Clin. Rheumatol..

[89] Costagliola G., Mosca M., Migliorini P., Consolini R. (2018). Pediatric Systemic Lupus Erythematosus: Learning From Longer Follow Up to Adulthood. Front. Pediatr..

[90] Torrente-Segarra V., Salman Monte T.C., Rúa-Figueroa I., Alonso F., López-Longo F.J., Galindo-Izquierdo M., Calvo-Alén J., Olivé-Marqués A., Ibáñez-Ruán J., Horcada L. (2017). Juvenile- and adult-onset systemic lupus erythematosus: A comparative study in a large cohort from the Spanish Society of Rheumatology Lupus Registry (RELESSER). Paediatr. Rheumatol. Clin. Exp. Rheumatol..

[91] Ponin L., Poomthavorn P., Pirojsakul K., Lerkvaleekul B., Soponkanaporn S., Chitrapazt N., Vilaiyuk S. (2022). Long-term growth and final adult height outcome in childhood-onset systemic lupus erythematosus. Pediatr. Rheumatol. Online J..

[92] Aydin A., Shan J., Brunner H.I., Mitsnefes M.M. (2018). Blood pressure control over time in childhood-onset systemic lupus erythematous. Lupus.

[93] Ahluwalia J., Singh S., Naseem S., Suri D., Rawat A., Gupta A., Masih J., Bose S. (2014). Antiphospholipid antibodies in children with systemic lupus erythematosus: A long-term clinical and laboratory follow-up status study from northwest India. Rheumatol. Int..

[94] Sinicato N.A., Postal M., de Oliveira Pelicari K., Rittner L., Marini R., Appenzeller S. (2017). Prevalence and features of metabolic syndrome in childhood-onset systemic lupus erythematosus. Clin. Rheumatol..

[95] Cabral M., Escobar C., Conde M., Ramos M., Gomes J.A.M. (2013). Juvenile Systemic Lupus Erythematosus in Portugal: Clinical and immunological patterns of disease expression in a cohort of 56 patients. Acta Reumatol. Port..

[96] Bernatsky S., Clarke A.E., Niaki O.Z., Labrecque J., Schanberg L.E., Silverman E.D., Hayward K., Imundo L., Brunner H.I., Haines K.A. (2017). Malignancy in pediatric-onset systemic lupus erythematosus. J. Rheumatol..

[97] Yang Y., Kumar S., Lim L.S.H., Silverman E.D., Levy D.M. (2015). Risk factors for symptomatic avascular necrosis in childhood-onset systemic lupus erythematosus. J. Rheumatol..

[98] Lin T.C., Wu J.Y., Kuo M.L., Ou L.S., Yeh K.W., Huang J.L. (2018). Correlation between disease activity of pediatric-onset systemic lupus erythematosus and level of vitamin D in Taiwan: A case–cohort study. J. Microbiol. Immunol. Infect..

[99] Kahwage P.P., Ferriani M.P.L., Furtado J.M., de Carvalho L.M., Pileggi G.S., Gomes F.H.R., Terreri M.T., Magalhães C.S., Pereira R.M.R., Sacchetti S.B. (2017). Uveitis in childhood-onset systemic lupus erythematosus patients: A multicenter survey. Clin. Rheumatol..

[100] Lai C.C., Sun Y.S., Chen W.S., Liao H.T., Chen M.H., Tsai C.Y., Huang D.F., Chou C.T., Chang D.M. (2022). Risk factors for mortality in systemic lupus erythematosus patients: Analysis of adult and pediatric cohorts in Taiwan. J. Chin. Med. Assoc..

[101] Al Hamzi H., Alhaymouni B., Al Shaikh A., Al-Mayouf S.M. (2014). Outcome of adult Saudi patients with childhood-onset systemic lupus erythematosus. Clin. Exp. Rheumatol..

[102] Tanigava N.Y., Sakamoto A.P., Franco A.S., Balbi G.G., Sales L.P., Aikawa N.E., Terreri M.T., Pereira R.M. (2022). Social impact of disease parameters and damage accrual in adult Brazilian patients with childhood-onset Systemic Lupus Erythematosus. Lupus.

[103] Hersh A.O., Von Scheven E., Yazdany J., Panopalis P., Trupin L., Julian L., Katz P., Criswell L.A., Yelin E. (2009). Differences in long-term disease activity and treatment of adult patients with childhoodand adult-onset systemic lupus erythematosus. Arthritis Care Res..

[104] Sousa S., Goncalves M.J., Ines L.S., Eugenio G., Jesus D., Fernandes S., Terroso G., Romao V.C., Cerqueira M., Raposo A. (2016). Clinical features and long-term outcomes of systemic lupus erythematosus: Comparative data of childhood, adult and late-onset disease in a national register. Rheumatol. Int..

[105] Koutsonikoli A., Trachana M., Heidich A.B., Galanopoulou V., Pratsidou-Gertsi P., Garyphallos A. (2015). Dissecting the damage in Northern Greek patients with childhood-onset systemic lupus erythematosus: A retrospective cohort study. Rheumatol. Int..

[106] Gamal S.M., Fouad N., Yosry N., Badr W., Sobhy N. (2022). Disease characteristics in patients with juvenile- and adult-onset systemic lupus erythematosus: A multi-center comparative study. Arch. Rheumatol..

[107] Cho S.K., Kim H., Myung J., Nam E., Jung S.Y., Jang E.J., Yoo D.H., Sung Y.K. (2019). Incidence and prevalence of idiopathic inflammatory myopathies in Korea: A nationwide population-based study. J. Korean Med. Sci..

[108] Concannon A., Han D.Y. (2021). Incidence, severity and clinical manifestations of juvenile dermatomyositis among Maori and Pacific Island compared to European children. J. Paediatr. Child. Health.

[109] Okong'o L.O., Esser M., Wilmshurst J., Scott C. (2016). Characteristics and outcome of children with juvenile dermatomyositis in Cape Town: A cross-sectional study. Pediatr. Rheumatol..

[110] Tabarki B., Ponsot G., Prieur A.M., Tardieu M. (1998). Childhood dermatomyositis: Clinical course of 36 patients treated with low doses of corticosteroids. Eur. J. Paediatr. Neurol..

[111] Chevalier G., Fakih O., Lhose A., Ballot-Schmit C., Prati C., Puzenat E., Aubin F. (2021). Long-term outcome in patients with juvenile dermatomyositis: A case series. Arch. Pediatr..

[112] Al-Mayouf S.M., AlMutiari N., Muzaffer M., Shehata R., Al-Wahadneh A., Abdwani R., Al-Abrawi S., Abu-Shukair M., El-Habahbeh Z., Alsonbul A. (2017). Phenotypic characteristics and outcome of juvenile dermatomyositis in Arab children. Rheumatol. Int..

[113] Sag E., Demir S., Bilginer Y., Talim B., Haliloglu G., Topaloglu H., Ozen S. (2021). Clinical features, muscle biopsy scores, myositis specific antibody profiles and outcome in juvenile dermatomyositis. Semin. Arthritis Rheum..

[114] Ramanan A.V., Campbell-Webster N., Ota S., Parker S., Tran D., Tyrrell P.N., Cameron B., Spiegel L., Schneider R., Laxer R.M. (2005). The effectiveness of treating juvenile dermatomyositis with methotrexate and aggressively tapered corticosteroids. Arthritis Rheum..

[115] Sun C., Lee J.H., Yang Y.H., Yu H.H., Wang L.C., Lin Y.T., Chiang B.L. (2015). Juvenile dermatomyositis: A 20-year retrospective analysis of treatment and clinical outcomes. Pediatr. Neonatol..

[116] Silverberg J.I., Kwa L., Kwa M.C., Laumann A.E., Ardalan K. (2018). Cardiovascular and cerebrovascular comorbidities of juvenile dermatomyositis in US children: An analysis of the National Inpatient Sample. Rheumatology.

[117] Boros C., McCann L., Simou S., Cancemi D., Ambrose N., Pilkington C.A., Cortina-Borja M., Wedderburn L.R., on behalf of the JDM Cohort and Biomarker Study (JDCBS) (2022). Juvenile Dermatomyositis: What comes next? Long-term outcomes in childhood myositis from a patient perspective. Pediatr. Rheumatol..

[118] Mathiesen P.R., Buchvald F., Nielsen K.G., Herlin T., Friis T., Nielsen S. (2014). Pulmonary function and autoantibodies in a long-term follow-up of juvenile dermatomyositis patients. Rheumatology.

[119] Mathiesen P., Hegaard H., Herlin T., Zak M., Pedersen F.K., Nielsen S. (2012). Long-term outcome in patients with juvenile dermatomyositis: A cross-sectional follow-up study. Scand. J. Rheumatol..

[120] Sanner H., Kirkhus E., Merckoll E., Tollisen A., Roisland M., Lie B.A., Taraldsrud E., Gran J.T., Flato B. (2010). Long-term muscular outcome and predisposing and prognostic factors in juvenile dermatomyositis: A case-control study. Arthritis Care Res..

[121] Sen E.S., Ramanan A.V. (2020). Juvenile idiopathic arthritis-associated uveitis. Clin. Immunol..

[122] Gonçalves Júnior J., Shinjo S.K. (2024). Calcinosis in juvenile dermatomyositis—Epidemiology, pathogenesis, clinical features, and treatment: A systematic review. Curr. Rheumatol. Rep..

[123] Rodriguez-Lozano A.L., Rivas-Larrauri F.E., Garcia-de la Puente S., Alcivar-Arteaga D.A., Gonzalez-Garay A.G. (2022). Prognostic Factors at Diagnosis Associated with Damage Accrual in Childhood-Onset Systemic Lupus Erythematosus Patients. Front. Pediatr..

[124] Eravsar A., Demirbas K.C., Aslan E., Akay N., Gul U., Konte E.K., Aslan E., Gunalp A., Haslak F., Yildiz M. (2025). Predictors of damage accrual in childhood-onset SLE: A retrospective analysis from a tertiary lupus centre in Turkiye. Lupus Sci. Med..

[125] Barsalou J., Bradley T.J., Silverman E.D. (2013). Cardiovascular risk in pediatric-onset rheumatological diseases. Arthritis Res. Ther..

[126] Ciurtin C., Robinson G., Butt M., Peng J., Ardoin S., Schanberg L., Boteanu A., Bouchalova K., Demir S., Moraitis E. (2024). Cardiovascular risk in young people with childhood onset systemic lupus erythematosus. Lancet Rheumatol..

[127] Nordal E., Pistorio A., Rygg M., Giancane G., Maghnie M., Di Iorgi N., Flemming K., Hofer M., Melo-Gomes J.A., Bica B. (2020). Growth and Puberty in Juvenile Dermatomyositis: A Longitudinal Cohort Study. Arthritis Care Res..

[128] Rygg M., Pistorio A., Ravelli A., Maghnie M., Di Iorgi N., Bader-Meunier B., Da Silva C., Roldan-Molina R., Barash J., Dracou C. (2012). A longitudinal PRINTO study on growth and puberty in juvenile systemic lupus erythematosus. Ann. Rheum. Dis..

[129] Polito C., Strano C., Olivieri A.N., Alessio M., Lammarrone C., Todisco N., Papale M. (1997). Growth retardation in non-steroid treated juvenile rheumatoid arthritis. Scand. J. Rheumatol..

[130] Milatz F., Klotsche J., Niewerth M., Sengler C., Windschall D., Kallinich T., Dressler F., Trauzeddel R., Holl R.W., Foeldvari I. (2024). Anxiety and depression symptoms in adolescents and young adults with juvenile idiopathic arthritis: Results of an outpatient screening. Arthritis Res. Ther..

[131] Duangmala P., Sontichai W. (2025). Depression and anxiety in childhood-onset systemic lupus erythematosus: Prevalence, associated factors, and impact on quality of life and family. Pediatr. Rheumatol..

[132] Reid M.R., Fabricius J., Danguecan A., Ardalan K., Knight A., Cunningham N.R. (2021). Anxiety and depression in childhood rheumatologic conditions: A topical review. Indian J. Rheumatol..

[133] Cunningham N.R., Danguecan A.N., Ely S.L., Amponsah Y., Davis A., Edison S., Harris J., Jones J.T., Goldstein-Leever A., Manning A. (2025). American College of Rheumatology Guidance Statements for Addressing Mental Health Concerns in Youth with Pediatric Rheumatologic Diseases. Arthritis Care Res..

