# Supplementary material for: Comorbidities in Juvenile-Onset Rheumatic Diseases: A Systematic Review and Meta-Analysis
Source: Children (Basel). 2026 Jul 28;13(8):995. doi: 10.3390/children13080995 (PMC13511721; doi:10.3390/children13080995)
Supplement: Supplementary file 1 [file children-13-00995-s001.zip › SiddiqEtal_SupplementaryData_SearchStrategy.pdf]

Supplementary Data S1

Search strategy

We used Medical Subject Headings (MeSH) terms and text words for paediatrics and JIA/jSLE/JDM, which were identified using peer-reviewed articles. Boolean operators were used to define search terms based on the PICO framework (Population/Problem, Intervention/Exposure, Comparison, Outcomes).

For this study, PICO was defined as:

Population/Problem: Patients with a diagnosis of juvenile arthritis/inflammatory diseases: JIA, jSLE and JDM.

Intervention/Exposure: Observe patients who were diagnosed with JIA/jSLE/JDM before 18 years old and continued into adulthood for long-term comorbidities.

Comparison: Patients without any inflammatory disease of the same age, sex and GP practice.

Outcomes: Long-term comorbidities developed to those patients in adulthood.

Search terms used to search for articles

For PubMed & Web of Science: ("juvenile idiopathic arthritis" OR ("Arthritis, Juvenile" [MeSH]) OR (juvenile) OR (childhood) OR (paediatric) OR (pediatric)) AND ("rheumatoid arthritis" OR arthritis OR "systemic arthritis" OR "psoriatic arthritis" OR spondyloarthritis OR enthesitis OR oligoarthritis OR dermatomyositis OR myositis OR polymyositis OR lupus OR "Arthritis, Psoriatic" [MeSH] OR "Lupus Erythematosus, Systemic" [MeSH] OR Dermatomyositis [MeSH] OR Polymyositis [MeSH]) AND (multimorbid* OR comorbid* OR polymorbid* OR (Multimorbidity [MeSH]) OR (comorbidity [MeSH]) OR (long-term AND outcome)).

For Scopus: ("juvenile idiopathic arthritis" OR (juvenile) OR (childhood) OR (paediatric) OR (pediatric)) AND ("rheumatoid arthritis" OR arthritis OR "systemic arthritis" OR "psoriatic arthritis" OR spondyloarthritis OR enthesitis OR oligoarthritis OR dermatomyositis OR myositis OR polymyositis OR lupus) AND (multimorbid* OR comorbid* OR polymorbid* OR (long-term AND outcome)).

There were no search restrictions imposed on publication time. Articles in languages other than English were excluded.
