# Supplementary material for: Comorbidities in Juvenile-Onset Rheumatic Diseases: A Systematic Review and Meta-Analysis
Source: Children (Basel). 2026 Jul 28;13(8):995. doi: 10.3390/children13080995 (PMC13511721; doi:10.3390/children13080995)
Supplement: Supplementary file 1 [file children-13-00995-s001.zip › SiddiqEtal_SupplementaryFigures.pdf]

Supplementary Figures for Comorbidities in Juvenile-Onset Rheumatic Diseases: A Systematic Review and Meta-Analysis

Sab Siddiq [1,†], Shabnam Cheetham [2,†], Clare E. Pain [3,4], Eve M. D. Smith [5], Sizheng Steven Zhao [6,7], Liza J. McCann [3,4,*,‡] and David M. Hughes [1,*,‡]

[1] Department of Health Data Science, Institute of Population Health, University of Liverpool, Waterhouse Building, Block F, 1–5 Brownlow Street, Liverpool L69 3GL, UK; sab.siddiq@liverpool.ac.uk
[2] Department of Paediatric Rheumatology, Royal Manchester Children's Hospital, Manchester University NHS Foundation Trust, Manchester M13 9WL, UK; shabnam.cheetham@nhs.net
[3] Department of Paediatric Rheumatology, Institute in the Park, Alder Hey Children's NHS Foundation Trust, Liverpool L14 5AB, UK; clare.pain@alderhey.nhs.uk
[4] Department of Women and Children's Health, Institute of Life Course and Medical Sciences, University of Liverpool, Liverpool L8 7SS, UK.
[5] School of Infection and Immunity, University of Glasgow, Glasgow G12 8TA, UK; eve.smith@glasgow.ac.uk
[6] Centre for Musculoskeletal Research, Faculty of Biology, Medicine and Health, School of Biological Sciences, Division of Musculoskeletal & Dermatological Sciences, University of Manchester, Oxford Road, Manchester M13 9PL, UK; steven.zhao.25@gmail.com
[7] NIHR Manchester Biomedical Research Centre, Manchester University NHS Foundation Trust, Manchester M13 9WU, UK
* Correspondence: liza.mccann@alderhey.nhs.uk (L.J.M.); dmhughes@liverpool.ac.uk (D.M.H.)
† Sab Siddiq and Shabnam Cheetham contributed equally to this study as joint first authors, with both authors contributing substantially to conceptualisation, data collection, analysis, drafting and revision of this manuscript.
‡ Liza J. McCann and David M. Hughes contributed equally to this study as joint senior authors.

Supplementary Figure S1: Distribution of Newcastle-Ottawa Scale bias scores of selected articles on Childhood

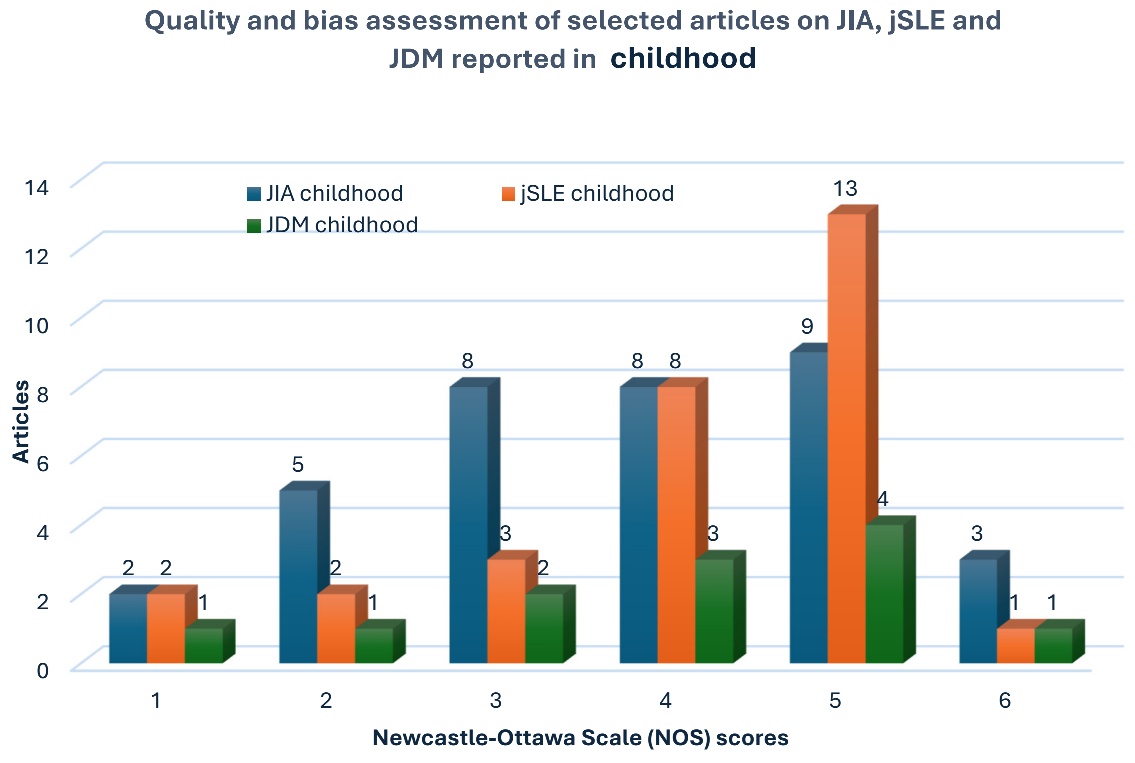

Supplementary Figure S2: Distribution of Newcastle-Ottawa Scale bias scores of selected articles on adulthood

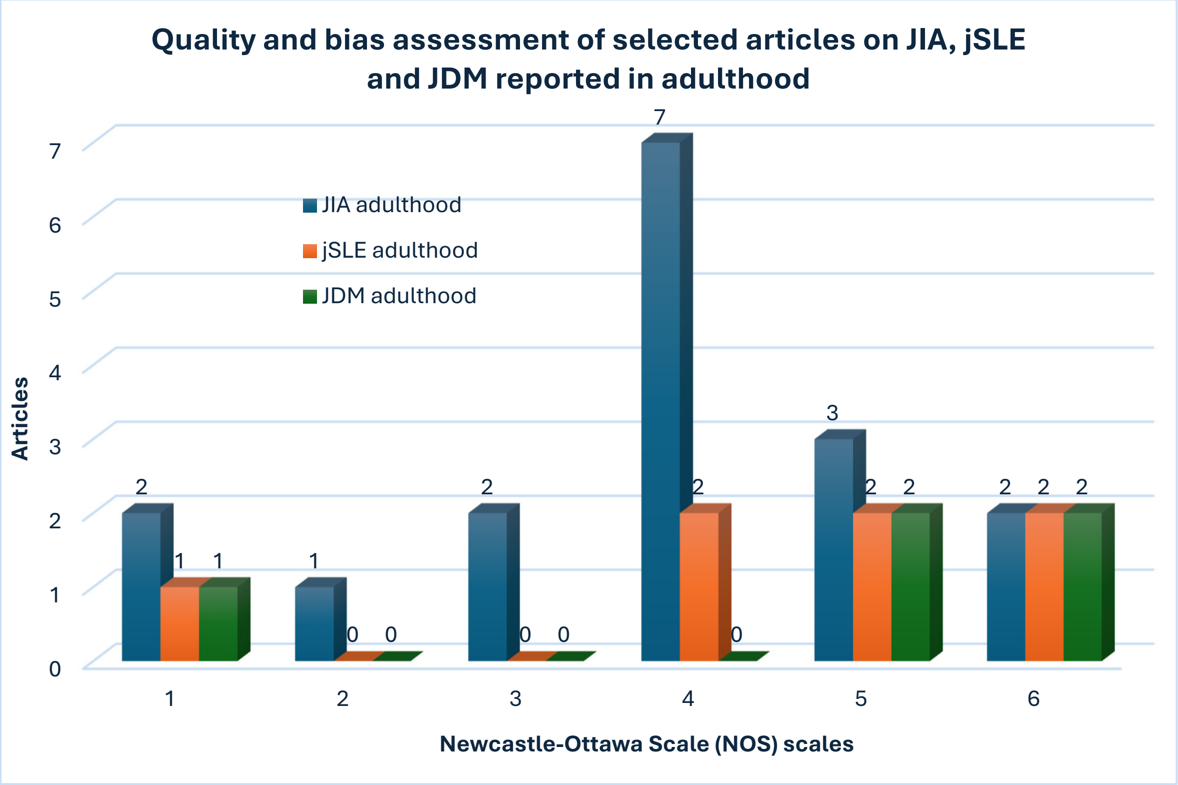

Supplementary Figure S3: Distribution of Newcastle-Ottawa Scale bias scores of selected combined articles

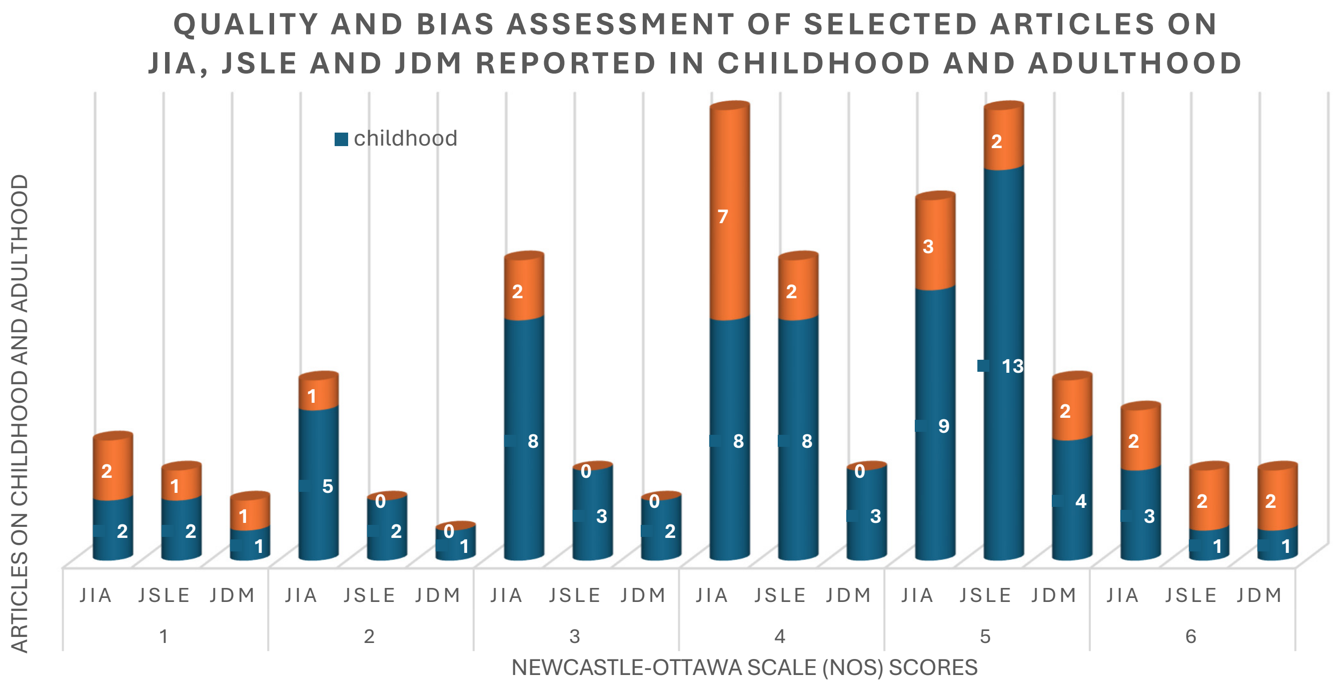

Supplementary Figure S4. Comorbidities reported in patients JIA, jSLE and JDM in selected articles in their childhood and adulthood[1]

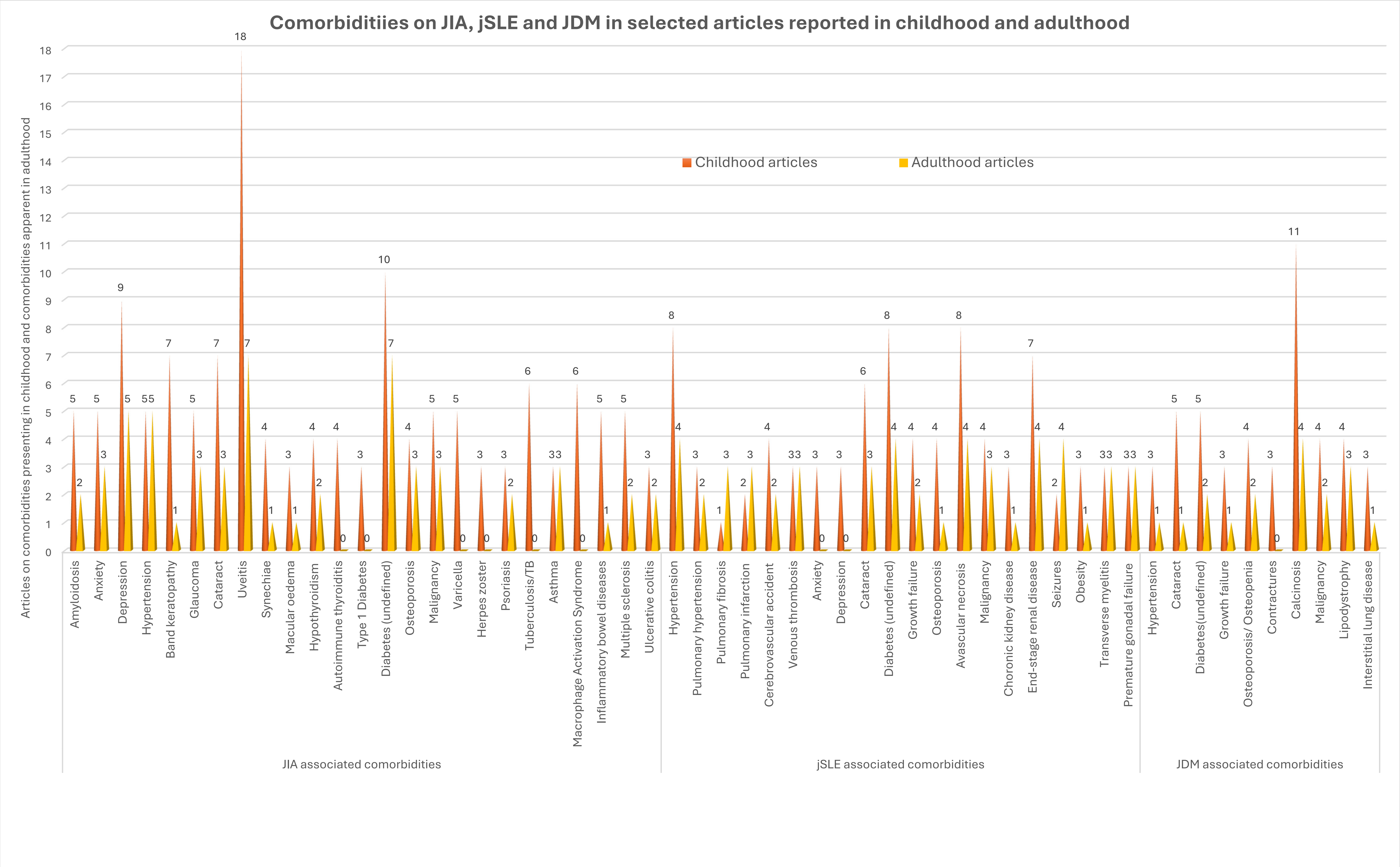

---

[1] Diabetes (undefined) included diabetes, gestational diabetes, diabetes mellitus, type 2 diabetes, diabetes mellitus (type 1 and 2) and insulin-dependent diabetes mellitus (IDDM). Macular edema included Cystoid macular oedema. Osteoporosis included osteoporosis with fracture, osteoporosis (BMD Z score≤-2.5) and osteoporosis/osteoporotic / BMD Z score≤-2.5). Cerebrovascular accident included cerebrovascular disease. End-stage renal disease (ESRD) included chronic renal failure, renal terminal failure, and persistent impairment of renal function. Obesity included visceral obesity.

Meta analysis: Pooled estimates, Forest plots and Funnel plots

JIA/Childhood

Amyloidosis

Supplementary Figure S5: Forest and Funnel Plots of studies reporting the prevalence of amyloidosis in children with Juvenile Idiopathic Arthritis

Forest

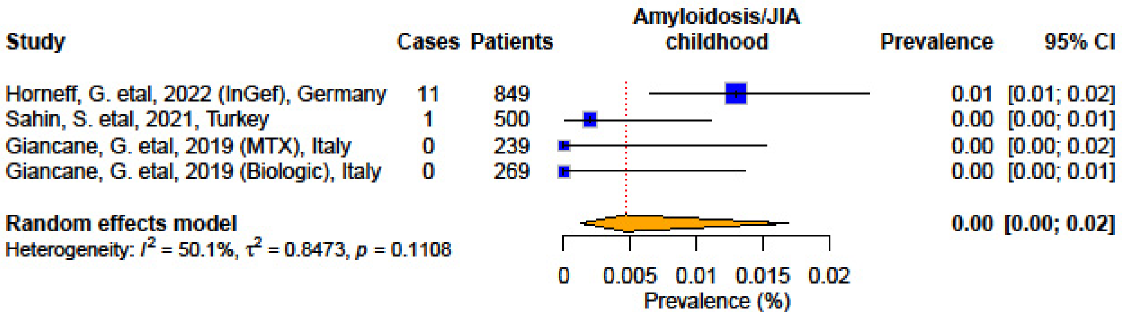

Funnel

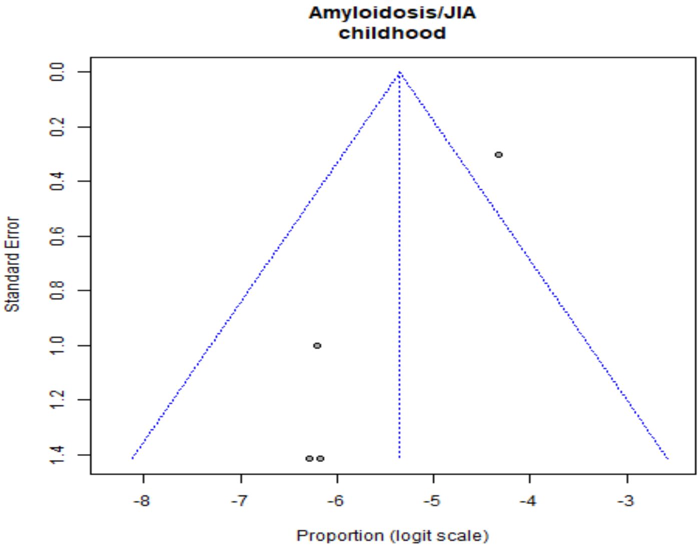

Summary statistics

```
                                        proportion          95%-CI     %W(random)
Horneff G. etal, 2022 (InGef), Germany      0.0130 [0.0065; 0.0231]      46.1
Sahin, S. etal, 2021, Turkey                0.0020 [0.0001; 0.0111]      23.4
Giancane G etal, 2019 (MTX), Italy          0.0000 [0.0000; 0.0153]      15.2
Giancane G etal, 2019 (Biologic), Italy     0.0000 [0.0000; 0.0136]      15.2
Number of studies: k = 4; Number of observations: o = 1857; Number of events: e = 12
                     proportion           95%-CI
Random effects model     0.0047 [0.0013; 0.0170]
Quantifying heterogeneity (with 95%-CIs): tau^2 = 0.8473 [0.0000; 11.9786]; tau = 0.9205 [0.0000; 3.4610]
 I^2 = 50.1% [0.0%; 83.5%]; H = 1.42 [1.00; 2.46]
Test of heterogeneity:
    Q d.f. p-value
 6.02    3  0.1108
```

Anxiety

Supplementary Figure S6: Forest and Funnel Plots of studies reporting the prevalence of anxiety in children with Juvenile Idiopathic Arthritis

Forest

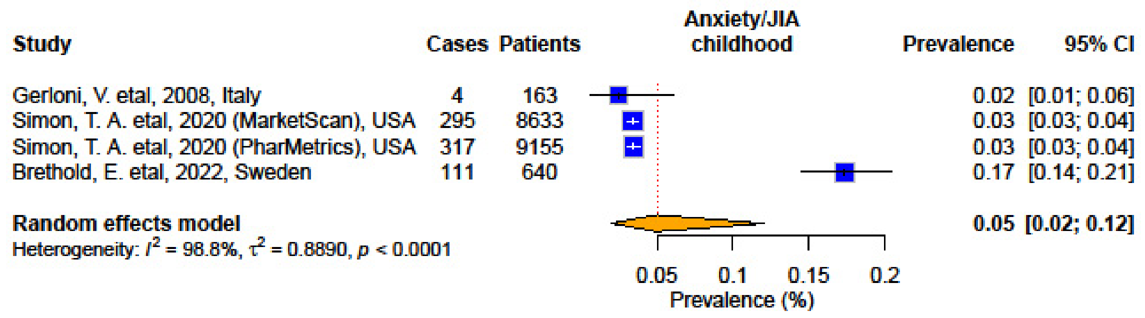

Funnel

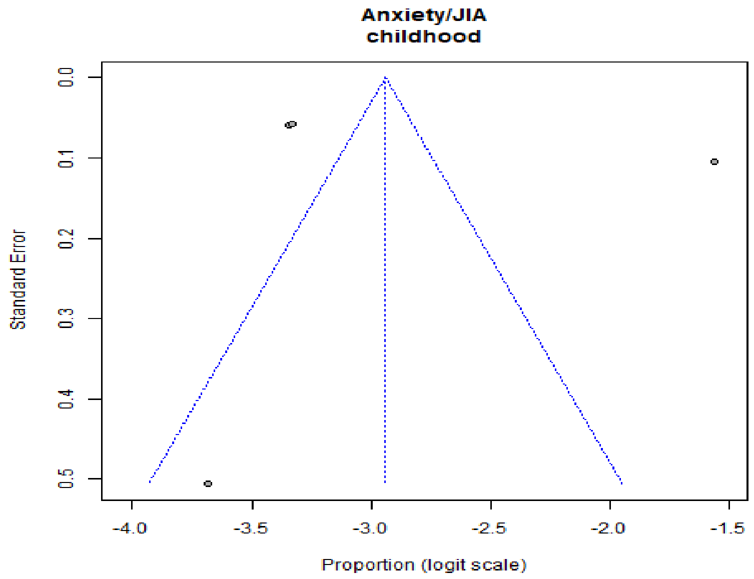

Summary statistics

```
                                              proportion           95%-CI  %W(random)
Gerloni, V. etal, 2008, Italy                     0.0245 [0.0067; 0.0616]   20.7
Simon, T. A. etal, 2020 (MarketScan), USA         0.0342 [0.0304; 0.0382]   26.5
Simon, T. A. etal, 2020 (PharMetrics), USA        0.0346 [0.0310; 0.0386]   26.5
Brethold E. etal, 2022, Sweden                    0.1734 [0.1449; 0.2050]   26.3
Number of studies: k = 4; Number of observations: o = 18591; Number of events: e = 727
                      proportion           95%-CI
Random effects model      0.0502 [0.0200; 0.1206]
Quantifying heterogeneity (with 95%-CIs): tau^2 = 0.8890 [0.2565; 12.7255]; tau = 0.9429 [0.5065; 3.5673]
 I^2 = 98.8% [98.2%; 99.2%]; H = 9.15 [7.38; 11.34]
Test of heterogeneity:
      Q d.f.  p-value
 251.03    3 < 0.0001
```

Asthma

Supplementary Figure S7: Forest and Funnel Plots of studies reporting the prevalence of asthma in children with Juvenile Idiopathic Arthritis

Forest

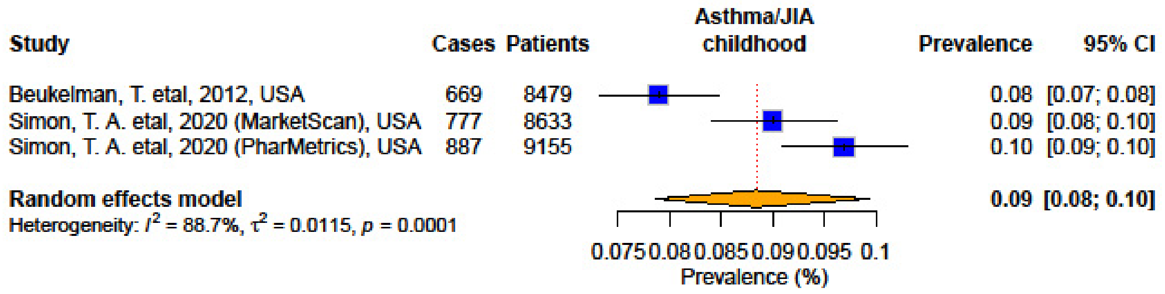

Funnel

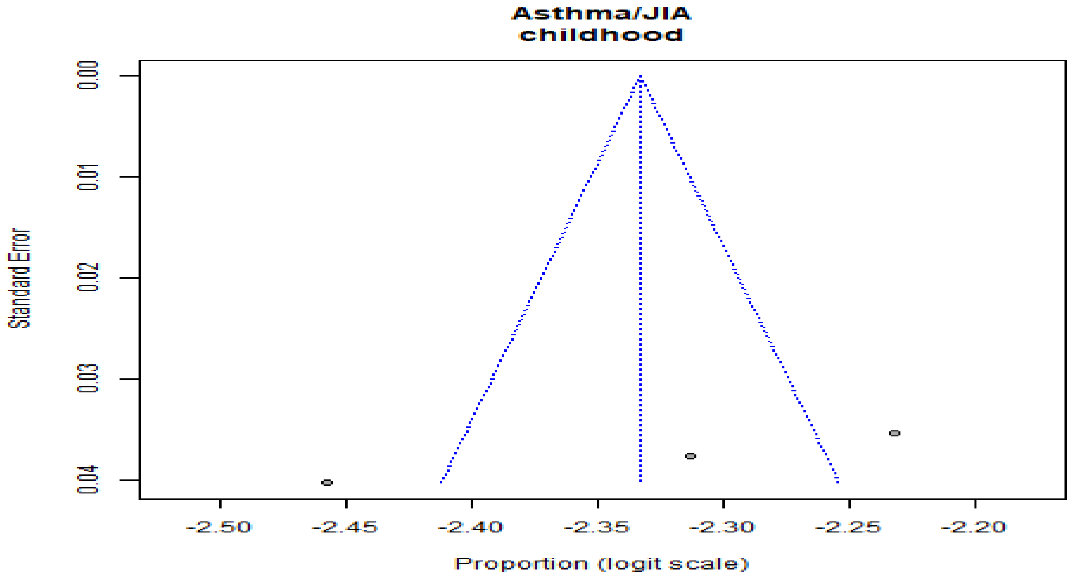

Summary statistics

```
                                            proportion           95%-CI  %W(random)
Beukelman, T. etal, 2012, USA                   0.0789 [0.0732; 0.0848]  32.8
Simon, T. A. etal, 2020 (MarketScan), USA       0.0900 [0.0840; 0.0962]  33.4
Simon, T. A. etal, 2020 (PharMetrics), USA      0.0969 [0.0909; 0.1031]  33.8
Number of studies: k = 3; Number of observations: o = 26267; Number of events: e = 2333
                      proportion           95%-CI
Random effects model      0.0884 [0.0786; 0.0993]
Quantifying heterogeneity (with 95%-CIs):
 tau^2 = 0.0115 [0.0021; 0.5116]; tau = 0.1072 [0.0454; 0.7153]
 I^2 = 88.7% [69.0%; 95.9%]; H = 2.98 [1.80; 4.94]
Test of heterogeneity:       Q d.f. p-value
                         17.77    2  0.0001
```

Autoimmune thyroiditis

Supplementary Figure S8: Forest and Funnel Plots of studies reporting the prevalence of autoimmune thyroiditis in children with Juvenile Idiopathic Arthritis

Forest

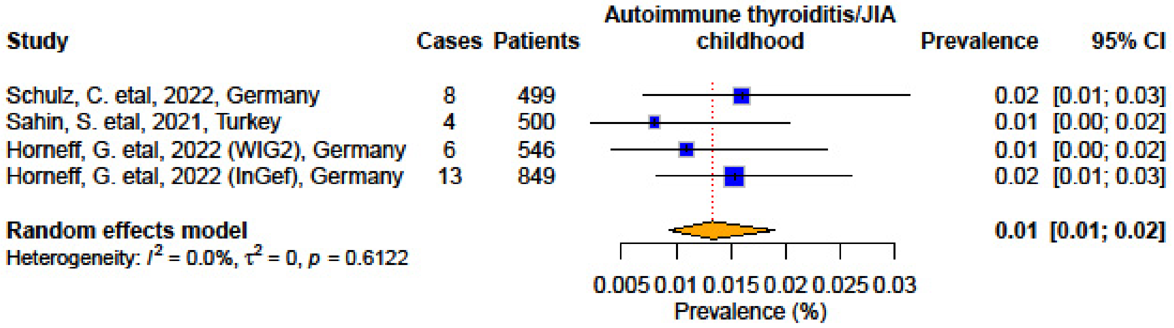

Funnel

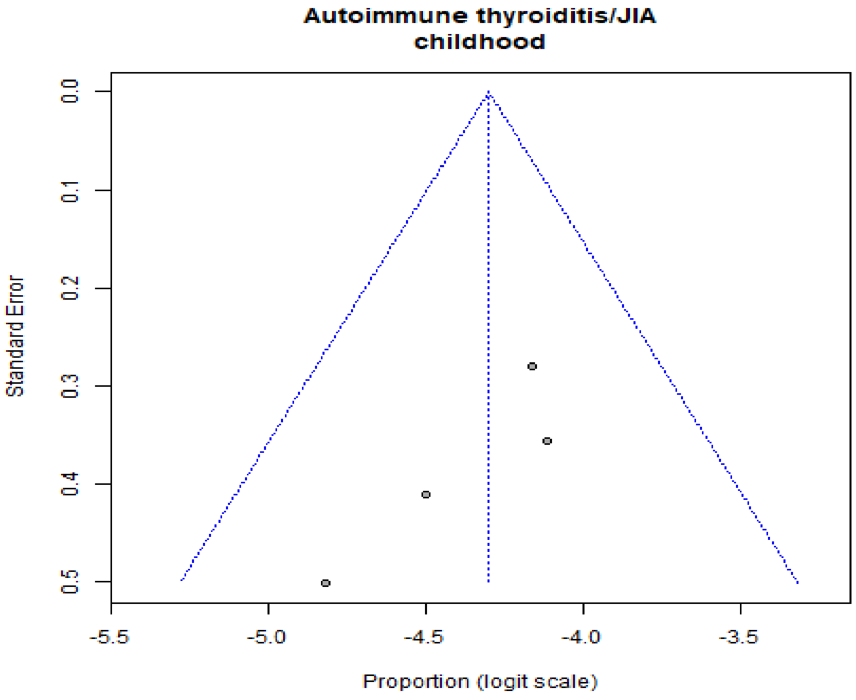

Summary statistics

```
                                       proportion           95%-CI      %W(random)
Schulz, C. etal, 2022, Germany             0.0160 [0.0069; 0.0313]       25.7
Sahin, S. etal, 2021, Turkey               0.0080 [0.0022; 0.0204]       13.0
Horneff G. etal, 2022 (WIG2), Germany      0.0110 [0.0040; 0.0238]       19.4
Horneff G. etal, 2022 (InGef), Germany     0.0153 [0.0082; 0.0260]       41.9
Number of studies: k = 4; Number of observations: o = 2394; Number of events: e = 31
                     proportion           95%-CI
Random effects model     0.0134 [0.0094; 0.0189]
Quantifying heterogeneity (with 95%-CIs):
 tau^2 = 0 [0.0000; 1.3075]; tau = 0 [0.0000; 1.1435]
 I^2 = 0.0% [0.0%; 84.7%]; H = 1.00 [1.00; 2.56]
Test of heterogeneity:      Q d.f. p-value
                         1.81    3  0.6122
```

Supplementary Figure S9: Forest and Funnel Plots of studies reporting the prevalence of band keratopathy in children with Juvenile Idiopathic Arthritis

Band keratopathy

Forest

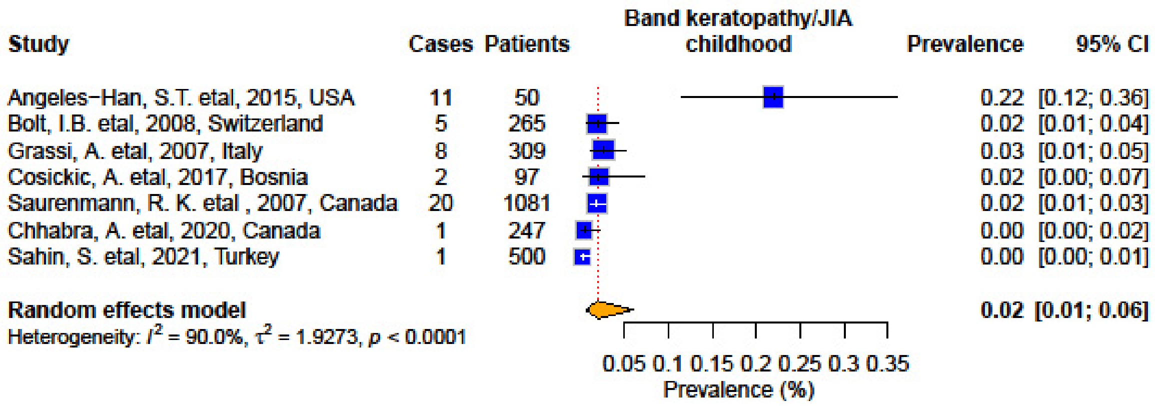

Funnel

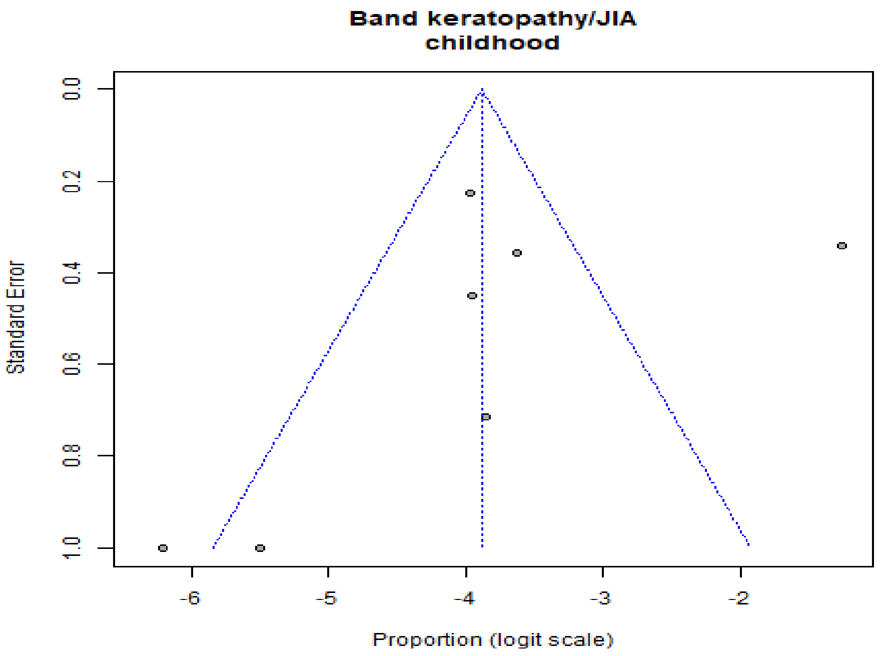

Summary statistics

```
                                     proportion           95%-CI       %W(random)
Angeles-Han S.T. etal, 2015, USA         0.2200 [0.1153; 0.3596]       16.1
Bolt I.B. etal, 2008, Switzerland        0.0189 [0.0062; 0.0435]       15.4
Grassi, A. etal, 2007, Italy             0.0259 [0.0112; 0.0504]       16.0
Cosickic, A. etal, 2017, Bosnia          0.0206 [0.0025; 0.0725]       13.5
Saurenmann, R. K. etal , 2007, Canada    0.0185 [0.0113; 0.0284]       16.6
Chhabra, A. etal, 2020, Canada           0.0040 [0.0001; 0.0223]       11.2
Sahin, S. etal, 2021, Turkey             0.0020 [0.0001; 0.0111]       11.2
Number of studies: k = 7; Number of observations: o = 2549; Number of events: e = 48
                      proportion           95%-CI
Random effects model     0.0201 [0.0066; 0.0593]
Quantifying heterogeneity (with 95%-CIs):  tau^2 = 1.9273 [0.6009; 11.4288]; tau = 1.3883 [0.7752; 3.3806]
 I^2 = 90.0% [81.9%; 94.5%]; H = 3.16 [2.35; 4.25]
Test of heterogeneity:       Q d.f.  p-value
                         | 59.88    6 < 0.0001
```

Supplementary Figure S10: Forest and Funnel Plots of studies reporting the prevalence of cataracts in children with Juvenile Idiopathic Arthritis

Cataract

Forest

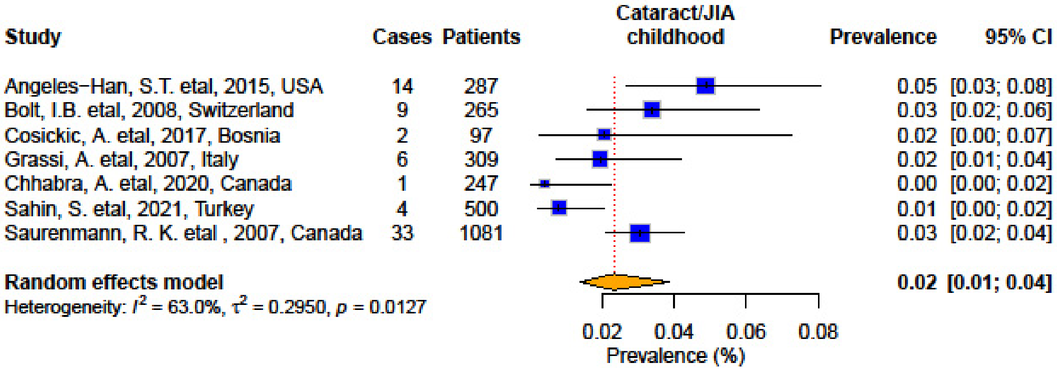

Funnel

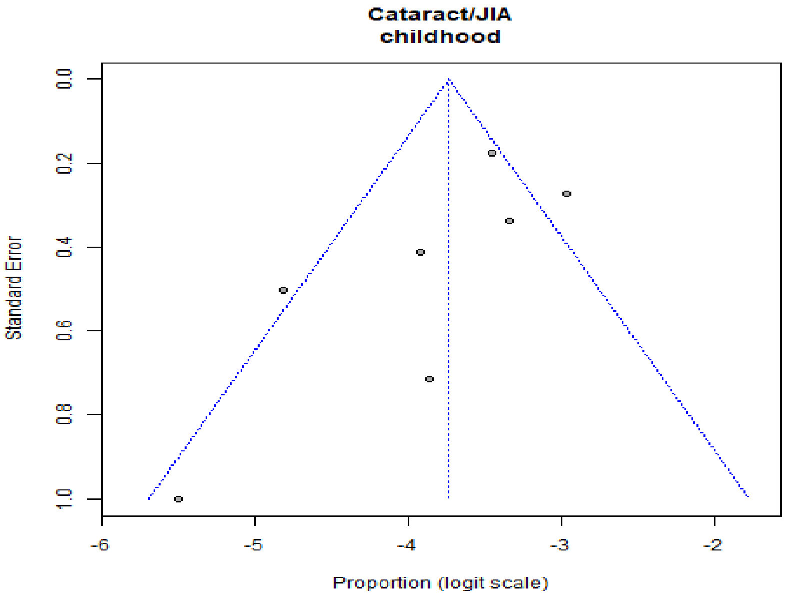

Summary statistics

```
                                      proportion           95%-CI       %W(random)
Angeles-Han S.T. etal, 2015, USA          0.0488 [0.0269; 0.0805]       19.0
Bolt I.B. etal, 2008, Switzerland         0.0340 [0.0156; 0.0635]       17.2
Cosickic, A. etal, 2017, Bosnia           0.0206 [0.0025; 0.0725]       8.7
Grassi, A. etal, 2007, Italy              0.0194 [0.0072; 0.0418]       15.2
Chhabra, A. etal, 2020, Canada            0.0040 [0.0001; 0.0223]       5.4
Sahin, S. etal, 2021, Turkey              0.0080 [0.0022; 0.0204]       12.9
Saurenmann, R. K. etal , 2007, Canada     0.0305 [0.0211; 0.0426]       21.6
Number of studies: k = 7; Number of observations: o = 2786; Number of events: e = 69
                     proportion           95%-CI
Random effects model    0.0232 [0.0139; 0.0385]
Quantifying heterogeneity (with 95%-CIs):  tau^2 = 0.2950 [0.0217; 3.3077]; tau = 0.5432 [0.1474; 1.8187]
 I^2 = 63.0% [16.0%; 83.7%]; H = 1.64 [1.09; 2.48]
Test of heterogeneity:      Q d.f. p-value
                         16.21     6  0.0127
```

Supplementary Figure S11: Forest and Funnel Plots of studies reporting the prevalence of depression in children with Juvenile Idiopathic Arthritis

Depression

Forest

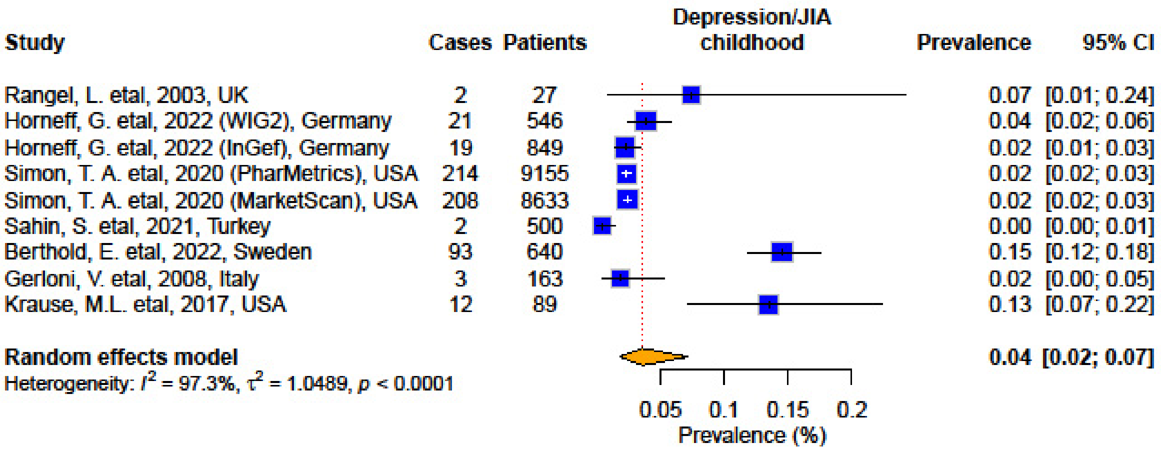

Funnel

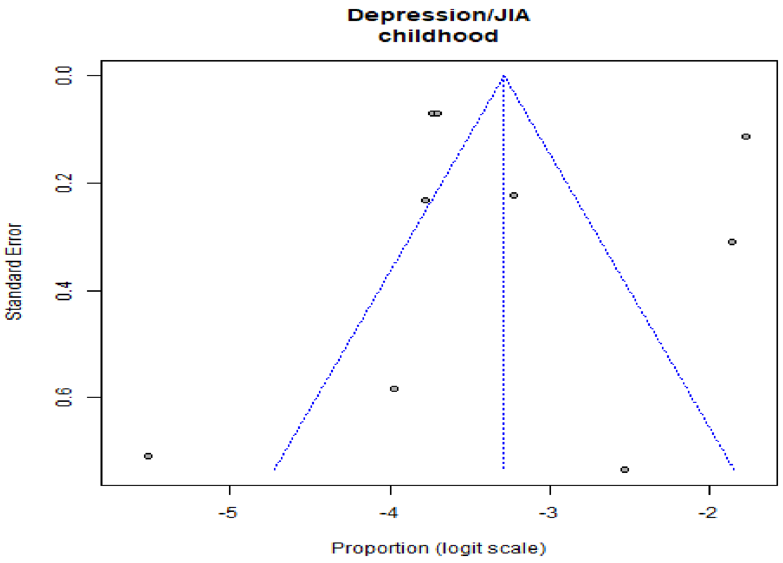

Summary statistics

```
                                                  proportion           95%-CI   %W(random)
Rangel, L. etal, 2003, UK                             0.0741 [0.0091; 0.2429]  8.4
Horneff G. etal, 2022 (WIG2), Germany                 0.0385 [0.0240; 0.0582]  12.1
Horneff G. etal, 2022 (InGef), Germany                0.0224 [0.0135; 0.0347]  12.1
Simon, T. A. etal, 2020 (PharMetrics), USA            0.0234 [0.0204; 0.0267]  12.6
Simon, T. A. etal, 2020 (MarketScan), USA             0.0241 [0.0210; 0.0276]  12.6
Sahin, S. etal, 2021                                  0.0040 [0.0005; 0.0144]  8.6
Berthold, E. etal, 2022                               0.1453 [0.1189; 0.1750]  12.5
Gerloni, V. etal, 2008, Italy                         0.0184 [0.0038; 0.0528]  9.6
Krause ML et al, 2017                                 0.1348 [0.0717; 0.2237]  11.6
Number of studies: k = 9; Number of observations: o = 20602; Number of events: e = 574
                      proportion           95%-CI
Random effects model      0.0361 [0.0180; 0.0711]
Quantifying heterogeneity (with 95%-CIs):  tau^2 = 1.0489 [0.4100; 4.7484]; tau = 1.0242 [0.6403; 2.1791]
 I^2 = 97.3% [96.1%; 98.1%]; H = 6.04 [5.07; 7.18]
Test of heterogeneity:         Q d.f.  p-value
                          291.44    8 < 0.0001
```

Supplementary Figure S12: Forest and Funnel Plots of studies reporting the prevalence of diabetes (undefined) in children with Juvenile Idiopathic Arthritis

Diabetes (undefined)

Forest

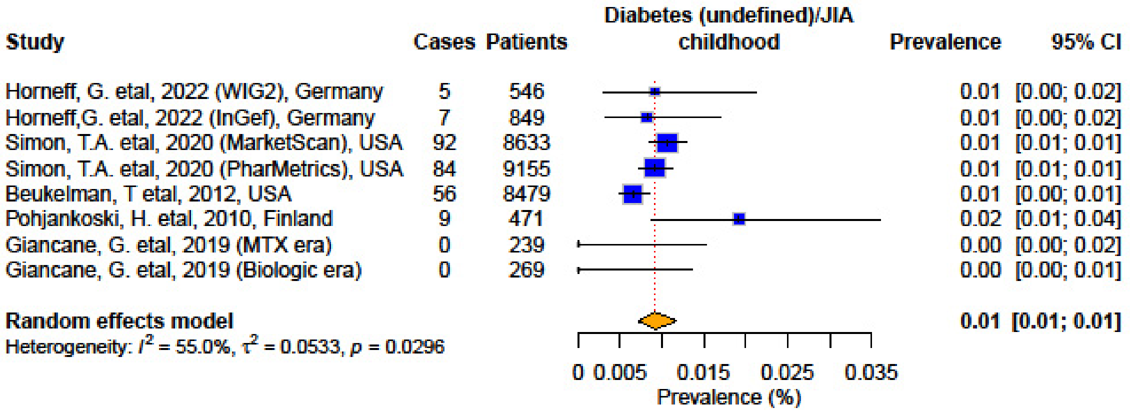

Funnel

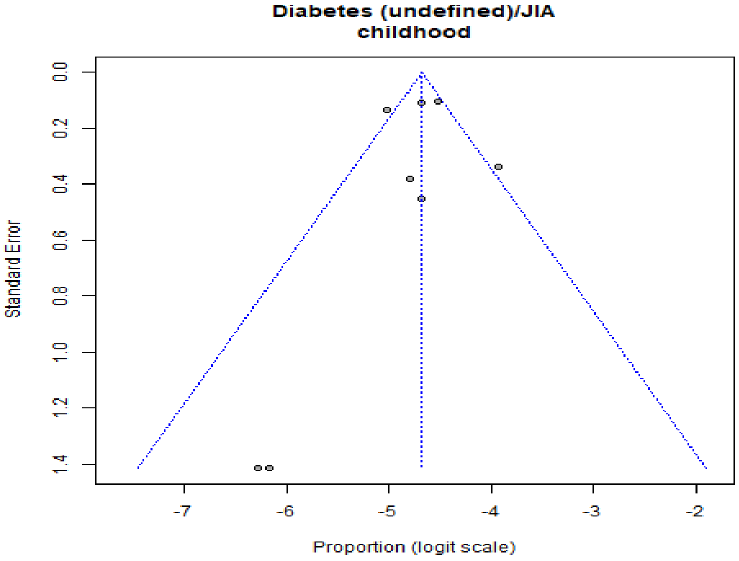

Summary statistics

```
                                          proportion           95%-CI    %W(random)
Horneff, G. etal, 2022 (WIG2), Germany        0.0092 [0.0030; 0.0212]    6.4
Horneff,G. etal, 2022 (InGef), Germany        0.0082 [0.0033; 0.0169]    8.3
Simon, TA. etal, 2020 (MarketScan), USA       0.0107 [0.0086; 0.0131]    25.6
Simon, TA. etal, 2020 (PharMetrics), USA      0.0092 [0.0073; 0.0113]    25.2
Beukelman, T etal, 2012, USA                  0.0066 [0.0050; 0.0086]    23.1
Pohjankoski, H. etal, 2010, Finland           0.0191 [0.0088; 0.0360]    9.9
Giancane G etal, 2019 (MTX era)               0.0000 [0.0000; 0.0153]    0.8
Giancane G etal, 2019 (Biologic era)          0.0000 [0.0000; 0.0136]    0.8
Number of studies: k = 8; Number of observations: o = 28641; Number of events: e = 253
                      proportion           95%-CI
Random effects model      0.0092 [0.0072; 0.0118]
Quantifying heterogeneity (with 95%-CIs):  tau^2 = 0.0533 [0.0000; 1.5500]; tau = 0.2309 [0.0000; 1.2450]
 I^2 = 55.0% [0.4%; 79.6%]; H = 1.49 [1.00; 2.22]
Test of heterogeneity:      Q d.f. p-value
                        15.55    7  0.0296
```

Supplementary Figure S13: Forest and Funnel Plots of studies reporting the prevalence of glaucoma in children with Juvenile Idiopathic Arthritis

Glaucoma

Forest

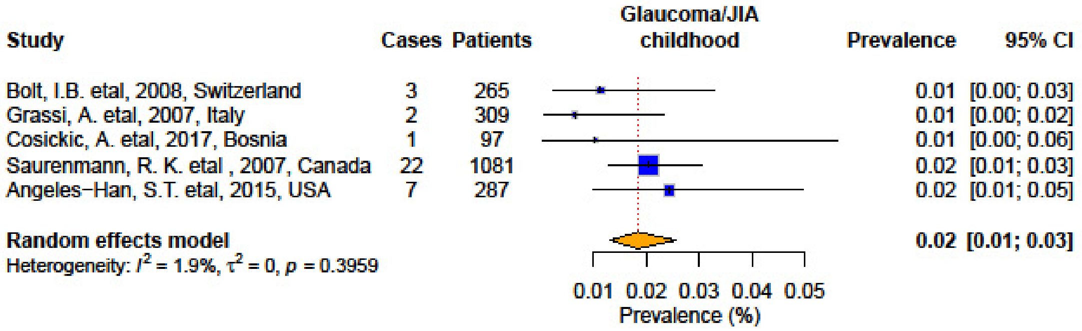

Funnel

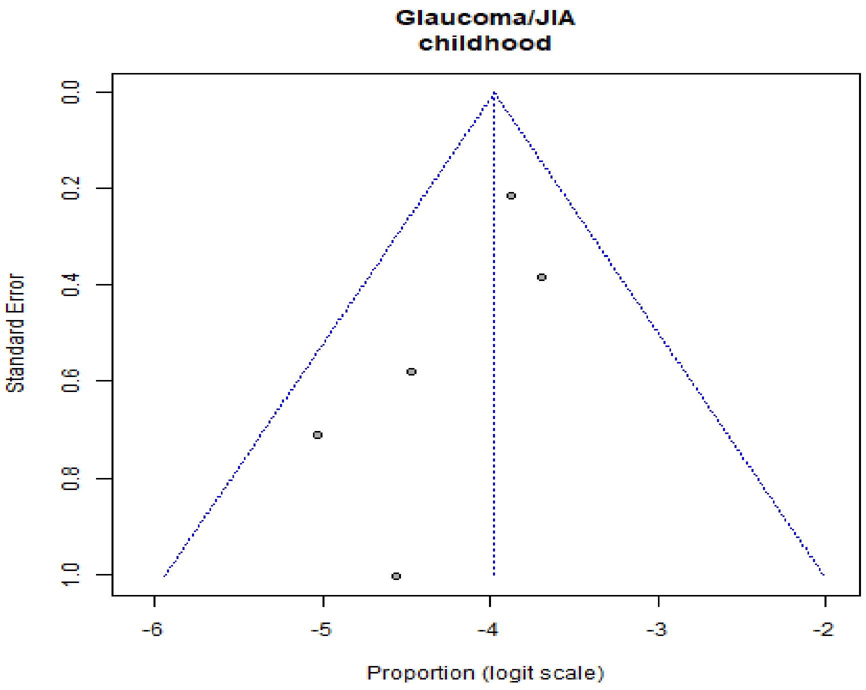

Summary statistics

```
                                           proportion           95%-CI       %W(random)
Bolt I.B. etal, 2008, Switzerland              0.0113 [0.0023; 0.0327]       8.6
Grassi, A. etal, 2007, Italy                   0.0065 [0.0008; 0.0232]       5.8
Cosickic, A. etal, 2017, Bosnia                0.0103 [0.0003; 0.0561]       2.9
Saurenmann, R. K. etal , 2007, Canada          0.0204 [0.0128; 0.0307]       62.8
Angeles-Han ST et al, 2015, USA                0.0244 [0.0099; 0.0496]       19.9
Number of studies: k = 5; Number of observations: o = 2039; Number of events: e = 35
                     proportion          95%-CI
Random effects model     0.0184 [0.0133; 0.0256]
Quantifying heterogeneity (with 95%-CIs):  tau^2 = 0 [0.0000; 2.1342]; tau = 0 [0.0000; 1.4609]
 I^2 = 1.9% [0.0%; 79.6%]; H = 1.01 [1.00; 2.21]
Test of heterogeneity:      Q d.f. p-value
                        4.08    4  0.3959
```

Supplementary Figure S14: Forest and Funnel Plots of studies reporting the prevalence of herpes zoster in children with Juvenile Idiopathic Arthritis

Herpes zoster

Forest

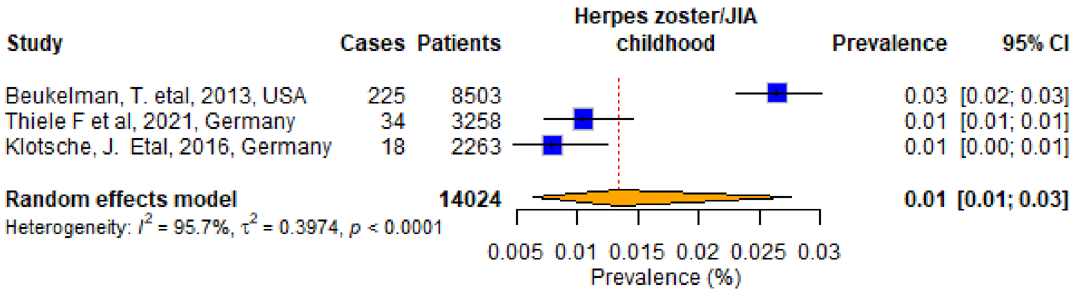

Funnel

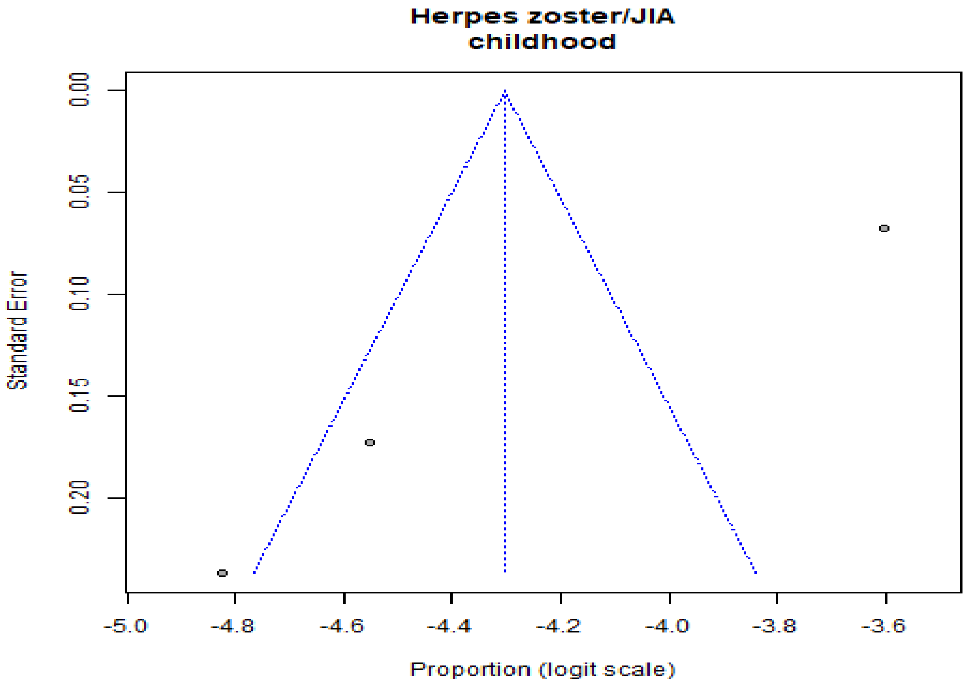

Summary statistics

```
                                    proportion           95%-CI    %W(random)
Beukelman, T. etal, 2013, USA           0.0265 [0.0232; 0.0301]       35.4
Thiele F et al, 2021, Germany           0.0104 [0.0072; 0.0146]       33.3
Klotsche, J.  Etal, 2016, Germany       0.0080 [0.0047; 0.0125]       31.4
Number of studies: k = 3; Number of observations: o = 14024; Number of events: e = 277
                     proportion           95%-CI
Random effects model     0.0133 [0.0064; 0.0275]
Quantifying heterogeneity (with 95%-CIs):  tau^2 = 0.3974 [0.0907; 16.1850]; tau = 0.6304 [0.3012; 4.0231]
 I^2 = 95.7% [90.5%; 98.0%]; H = 4.80 [3.25; 7.10]
Test of heterogeneity:      Q d.f.  p-value
                        46.13    2 < 0.0001
```

Supplementary Figure S15: Forest and Funnel Plots of studies reporting the prevalence of hypothyroidism in children with Juvenile Idiopathic Arthritis

Hypothyroidism

Forest

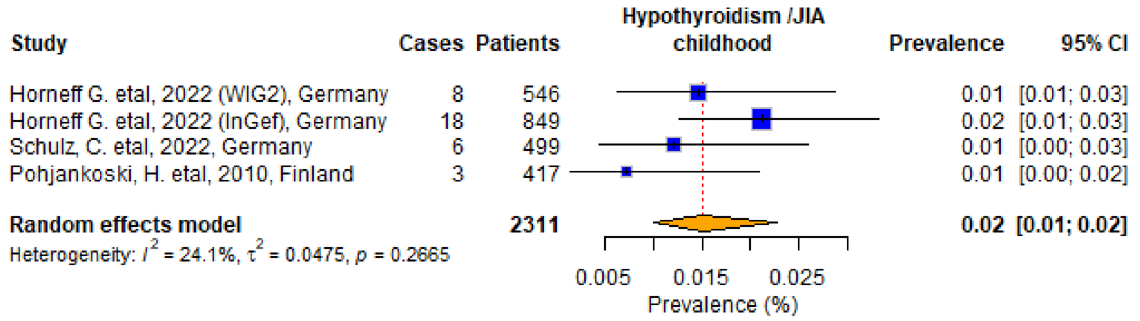

Funnel

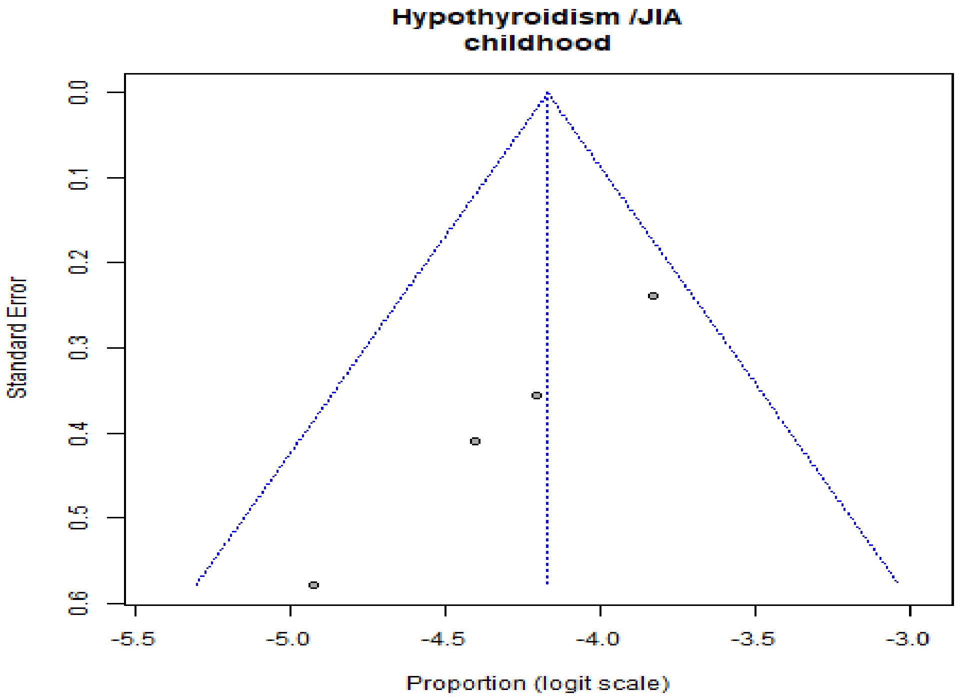

Summary statistics

```
                                        proportion           95%-CI       %W(random)
Horneff G. etal, 2022 (WIG2), Germany       0.0147 [0.0063; 0.0287]       25.4
Horneff G. etal, 2022 (InGef), Germany      0.0212 [0.0126; 0.0333]       42.5
Schulz, C. etal, 2022, Germany              0.0120 [0.0044; 0.0260]       20.5
Pohjankoski, H. etal, 2010, Finland         0.0072 [0.0015; 0.0209]       11.6
Number of studies: k = 4; Number of observations: o = 2311; Number of events: e = 35
                      proportion           95%-CI
Random effects model      0.0152 [0.0101; 0.0228]
Quantifying heterogeneity (with 95%-CIs):  tau^2 = 0.0475 [0.0000; 2.6861]; tau = 0.2179 [0.0000; 1.6389]
 I^2 = 24.1% [0.0%; 88.4%]; H = 1.15 [1.00; 2.93]
Test of heterogeneity:     Q d.f. p-value
                         3.95     3  0.2665
```

Supplementary Figure S16: Forest and Funnel Plots of studies reporting the prevalence of hypertension in children with Juvenile Idiopathic Arthritis

Hypertension

Forest

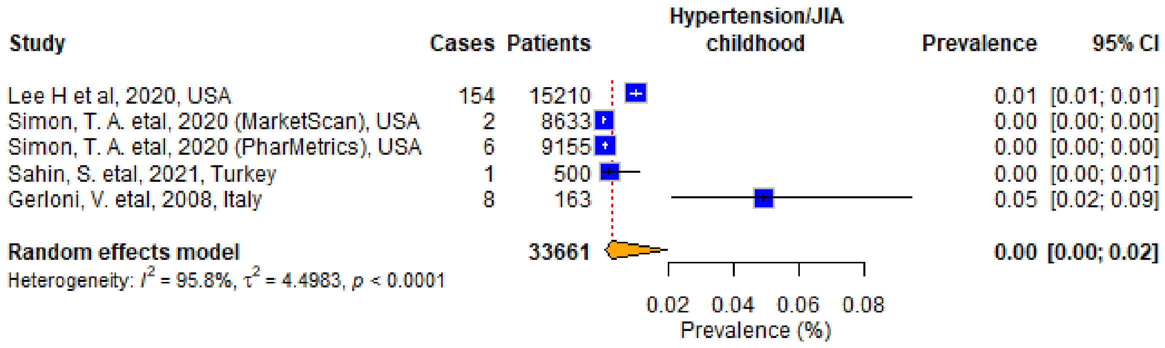

Funnel

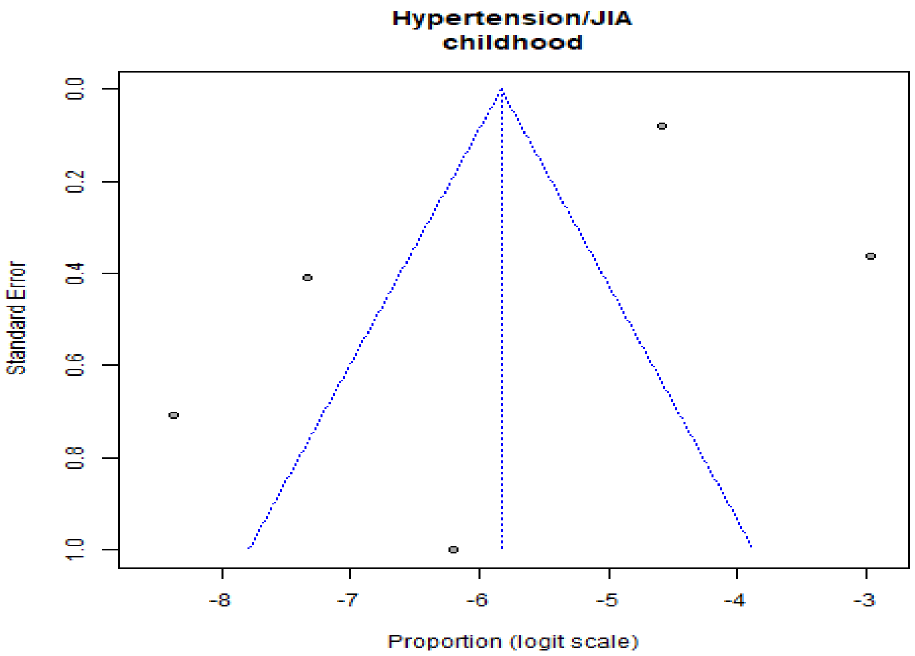

Summary statistics

```
                                          proportion           95%-CI  %W(random)
Lee H et al, 2020, USA                        0.0101 [0.0086; 0.0118]  21.5
Simon, T. A. etal, 2020 (MarketScan), USA     0.0002 [0.0000; 0.0008]  19.3
Simon, T. A. etal, 2020 (PharMetrics), USA    0.0007 [0.0002; 0.0014]  20.7
Sahin, S. etal, 2021, Turkey                  0.0020 [0.0001; 0.0111]  17.6
Gerloni, V. etal, 2008, Italy                 0.0491 [0.0214; 0.0944]  20.9
Number of studies: k = 5; Number of observations: o = 33661; Number of events: e = 171
                     proportion           95%-CI
Random effects model     0.0029 [0.0004; 0.0197]
Quantifying heterogeneity (with 95%-CIs):  tau^2 = 4.4983 [1.4273; 38.1419]; tau = 2.1209 [1.1947; 6.1759]
 I^2 = 95.8% [92.7%; 97.6%]; H = 4.89 [3.70; 6.45]
Test of heterogeneity:       Q d.f.  p-value
                         95.46    4 < 0.0001
```

Supplementary Figure S17: Forest and Funnel Plots of studies reporting the prevalence of Inflammatory Bowel Diseases in children with Juvenile Idiopathic Arthritis

Inflammatory bowel diseases

Forest

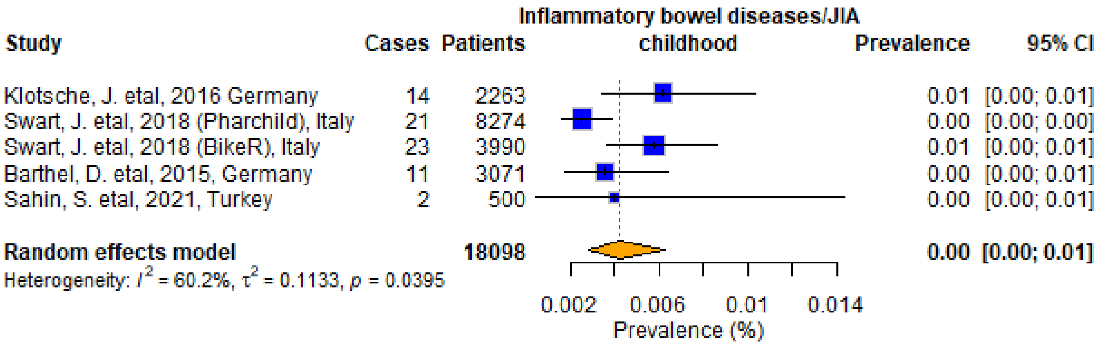

Funnel

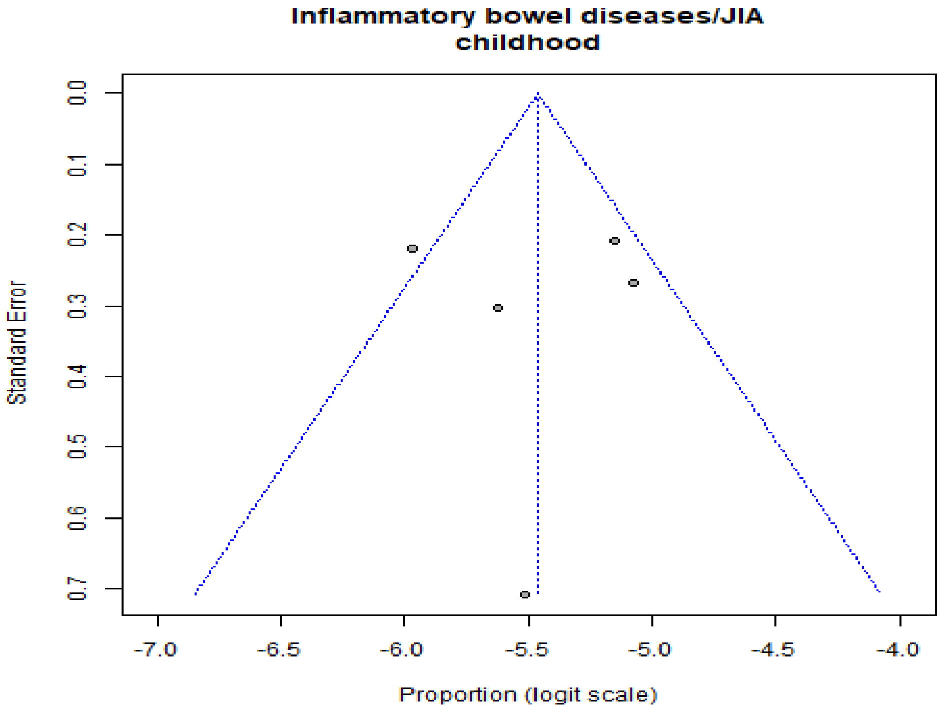

Summary statistics

```
                                         proportion           95%-CI      %W(random)
Klotsche, J. etal, 2016 Germany              0.0062 [0.0034; 0.0104]      22.0
Swart, J. etal, 2018 (Pharchild), Italy      0.0025 [0.0016; 0.0039]      25.4
Swart, J. etal, 2018 (BikeR), Italy          0.0058 [0.0037; 0.0086]      26.0
Barthel, D. etal, 2015, Germany              0.0036 [0.0018; 0.0064]      20.0
Sahin, S. etal, 2021, Turkey                 0.0040 [0.0005; 0.0144]      6.6
Number of studies: k = 5; Number of observations: o = 18098; Number of events: e = 71
                      proportion           95%-CI
Random effects model      0.0042 [0.0028; 0.0063]
Quantifying heterogeneity (with 95%-CIs):  tau^2 = 0.1133 [0.0000; 1.0495]; tau = 0.3366 [0.0000; 1.0245]
 I^2 = 60.2% [0.0%; 85.1%]; H = 1.59 [1.00; 2.59]
Test of heterogeneity:         Q d.f. p-value
                           10.06    4  0.0395
```

Supplementary Figure S18: Forest and Funnel Plots of studies reporting the prevalence of malignancy in children with Juvenile Idiopathic Arthritis

Malignancy

Forest

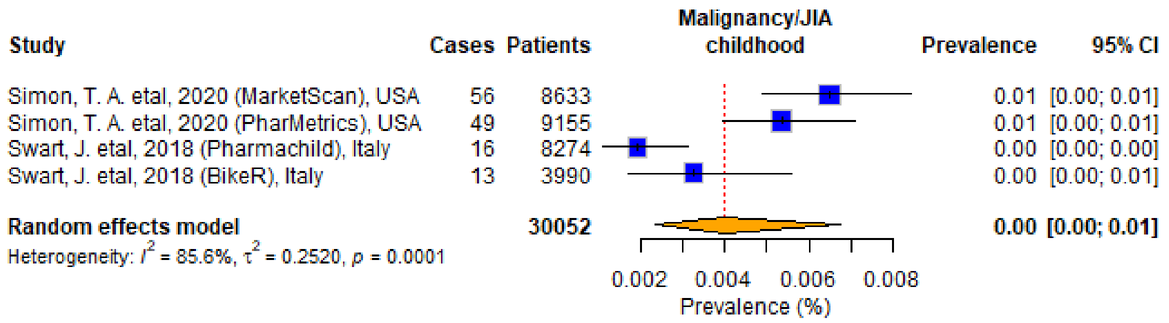

Funnel

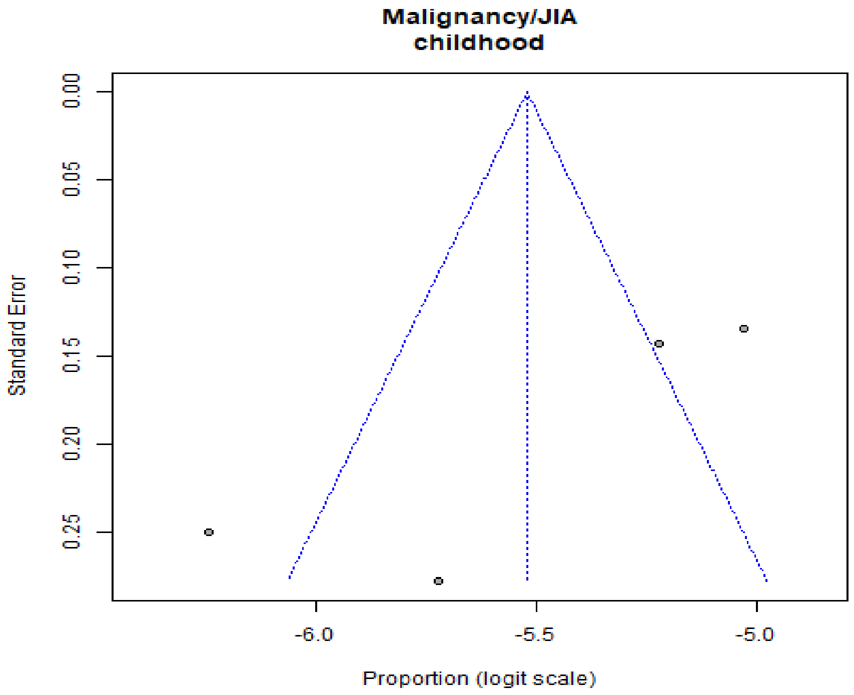

Summary statistics

```
                                           proportion           95%-CI  %W(random)
Simon, T. A. etal, 2020 (MarketScan), USA      0.0065 [0.0049; 0.0084]  27.3
Simon, T. A. etal, 2020 (PharMetrics), USA     0.0054 [0.0040; 0.0071]  27.0
Swart, J. etal, 2018 (Pharmachild), Italy      0.0019 [0.0011; 0.0031]  23.4
Swart, J. etal, 2018 (BikeR), Italy            0.0033 [0.0017; 0.0056]  22.4
Number of studies: k = 4; Number of observations: o = 30052; Number of events: e = 134
                      proportion           95%-CI
Random effects model     0.0040 [0.0023; 0.0068]
Quantifying heterogeneity (with 95%-CIs):  tau^2 = 0.2520 [0.0517; 4.0772]; tau = 0.5020 [0.2273; 2.0192]
 I^2 = 85.6% [64.7%; 94.2%]; H = 2.64 [1.68; 4.14]
Test of heterogeneity:       Q d.f. p-value
                         20.88    3  0.0001
```

Supplementary Figure S19: Forest and Funnel Plots of studies reporting the prevalence of macrophage activation syndrome in children with Juvenile Idiopathic Arthritis

Macrophage activation syndrome

Forest

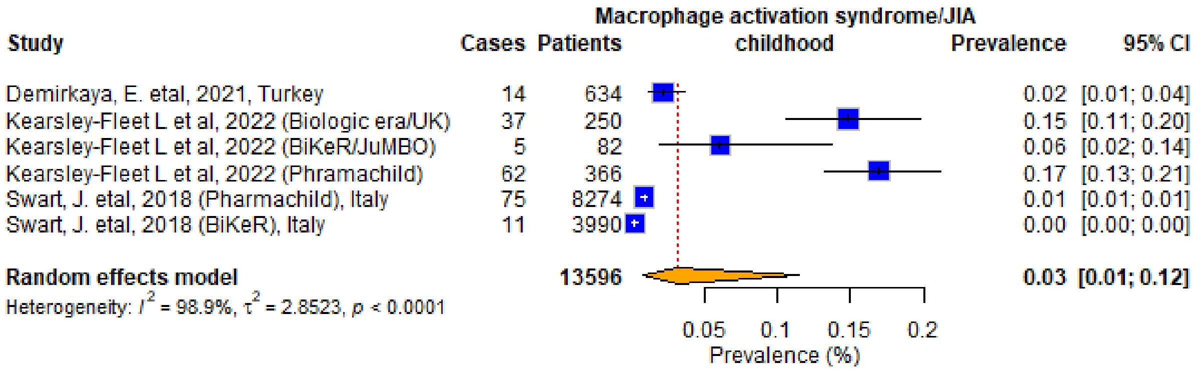

Funnel

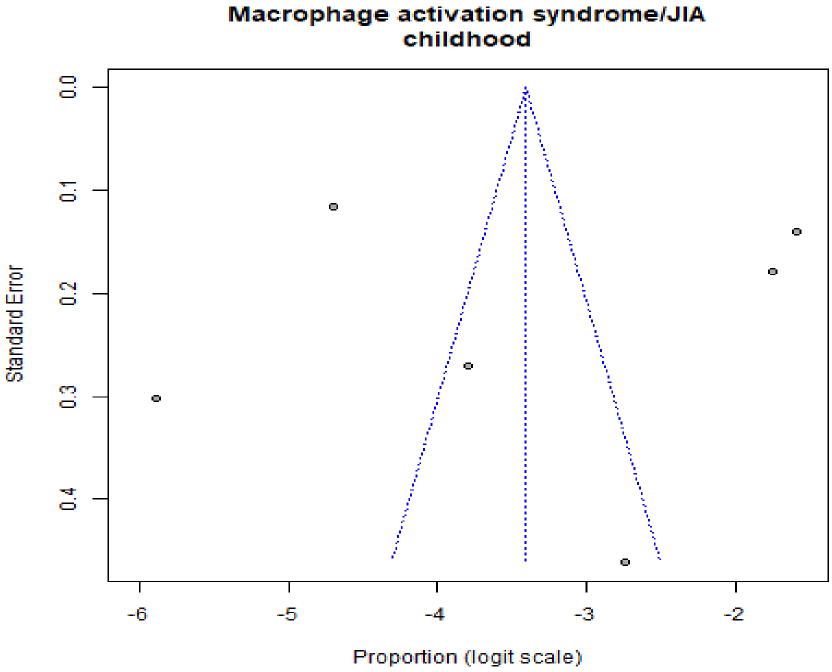

Summary statistics

```
                                              proportion           95%-CI %W(random)
Demirkaya, E. etal, 2021, Turkey                  0.0221 [0.0121; 0.0368]       16.7
Kearsley-Fleet L et al, 2022 (Biologic era/UK)    0.1480 [0.1064; 0.1982]       16.9
Kearsley-Fleet L et al, 2022 (BiKeR/JuMBO)        0.0610 [0.0201; 0.1366]       15.9
Kearsley-Fleet L et al, 2022 (Phramachild)        0.1694 [0.1324; 0.2118]       17.0
Swart, J. etal, 2018 (Pharmachild), Italy         0.0091 [0.0071; 0.0113]       17.0
Swart, J. etal, 2018 (BiKeR), Italy               0.0028 [0.0014; 0.0049]       16.6
Number of studies: k = 6; Number of observations: o = 13596; Number of events: e = 204
                     proportion           95%-CI
Random effects model     0.0321 [0.0084; 0.1153]
Quantifying heterogeneity (with 95%-CIs):  tau^2 = 2.8523 [1.0712; 17.3534]; tau = 1.6889 [1.0350; 4.1657]
 I^2 = 98.9% [98.5%; 99.2%]; H = 9.48 [8.05; 11.16]
Test of heterogeneity:        Q d.f.  p-value
                        449.21    5 < 0.0001
```

Supplementary Figure S20: Forest and Funnel Plots of studies reporting the prevalence of macular oedema in children with Juvenile Idiopathic Arthritis

Macular Oedema

Forest

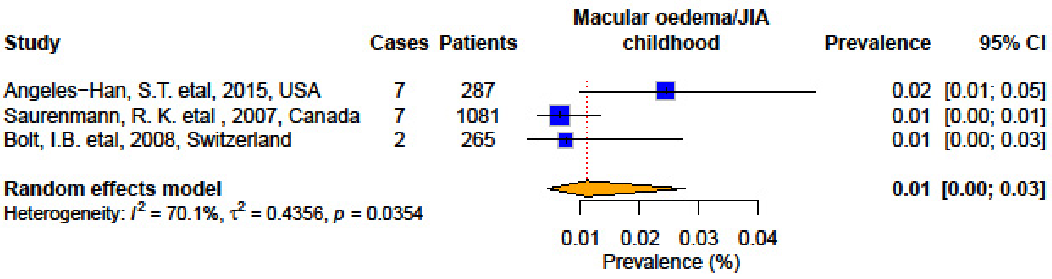

Funnel

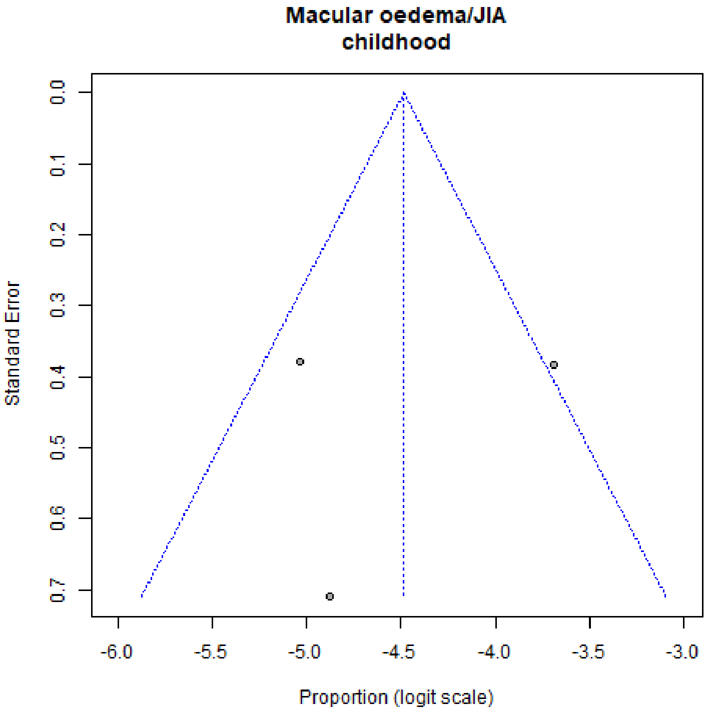

Summary statistics

```
                                       proportion           95%-CI %W(random)
Angeles-Han S.T. etal, 2015, USA           0.0244 [0.0099; 0.0496]       38.1
Saurenmann, R. K. etal , 2007, Canada      0.0065 [0.0026; 0.0133]       38.3
Bolt I.B. etal, 2008, Switzerland          0.0075 [0.0009; 0.0270]       23.6
Number of studies: k = 3; Number of observations: o = 1633; Number of events: e = 16
                     proportion           95%-CI
Random effects model     0.0112 [0.0045; 0.0276]
Quantifying heterogeneity (with 95%-CIs):  tau^2 = 0.4356 [0.0000; 21.1933]; tau = 0.6600 [0.0000; 4.6036]
 I^2 = 70.1% [0.0%; 91.2%]; H = 1.83 [1.00; 3.38]
Test of heterogeneity:     Q d.f. p-value
                        6.68    2  0.0354
```

Supplementary Figure S21: Forest and Funnel Plots of studies reporting the prevalence of Multiple Sclerosis in children with Juvenile Idiopathic Arthritis

Multiple sclerosis

Forest

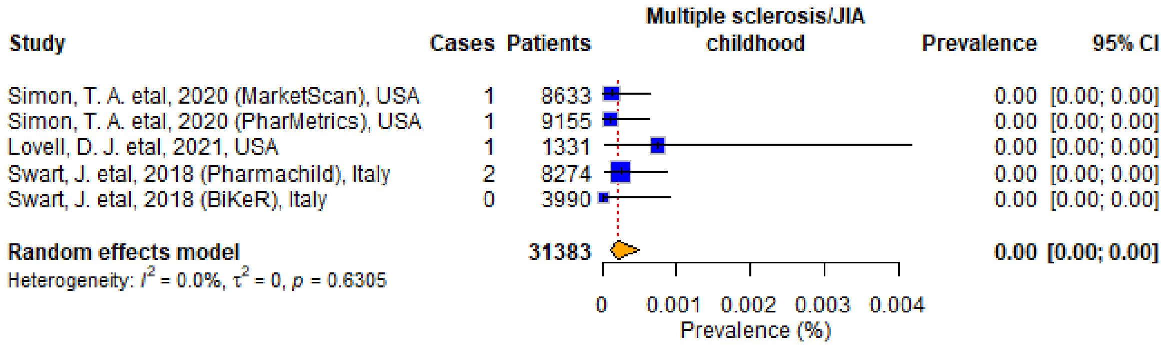

Funnel

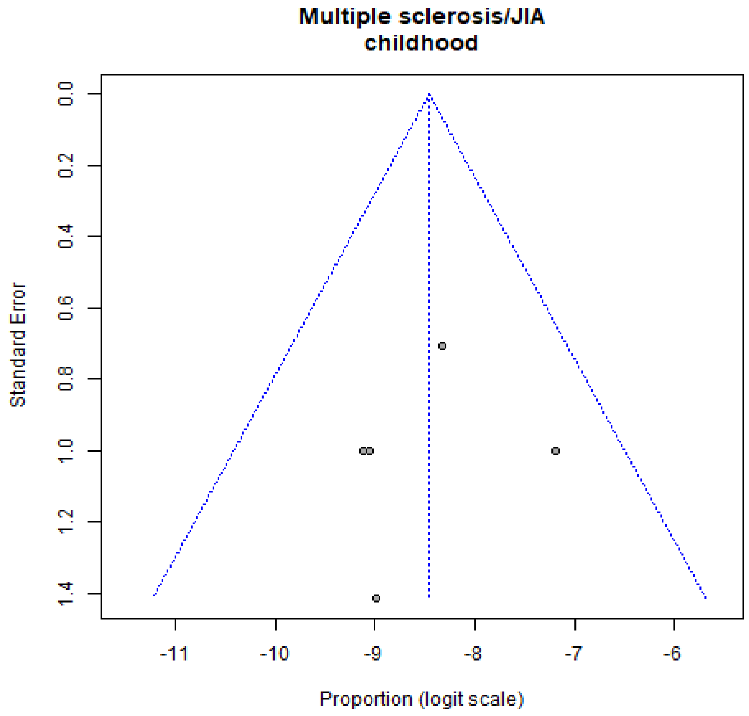

Summary statistics

```
                                            proportion           95%-CI %W(random)
Simon, T. A. etal, 2020 (MarketScan), USA       0.0001 [0.0000; 0.0006]       18.2
Simon, T. A. etal, 2020 (PharMetrics), USA      0.0001 [0.0000; 0.0006]       18.2
Lovell, D. J. etal, 2021, USA                   0.0008 [0.0000; 0.0042]       18.2
Swart, J. etal, 2018 (Pharmachild), Italy       0.0002 [0.0000; 0.0009]       36.4
Swart, J. etal, 2018 (BiKeR), Italy             0.0000 [0.0000; 0.0009]        9.1
Number of studies: k = 5; Number of observations: o = 31383; Number of events: e = 5
                      proportion           95%-CI
Random effects model      0.0002 [0.0001; 0.0005]
Quantifying heterogeneity (with 95%-CIs):  tau^2 = 0 [0.0000; 4.4533]; tau = 0 [0.0000; 2.1103]
 I^2 = 0.0% [0.0%; 79.2%]; H = 1.00 [1.00; 2.19]
Test of heterogeneity:     Q d.f. p-value
                        2.58    4  0.6305
```

Supplementary Figure S22: Forest and Funnel Plots of studies reporting the prevalence of osteoporosis in children with Juvenile Idiopathic Arthritis

Osteoporosis

Forest

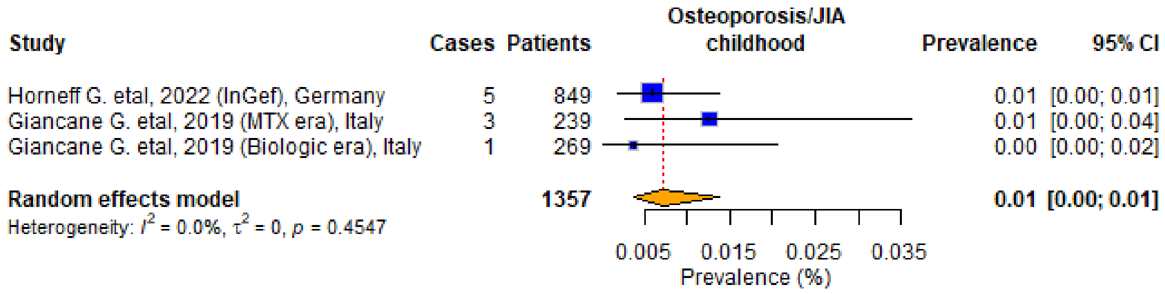

Funnel

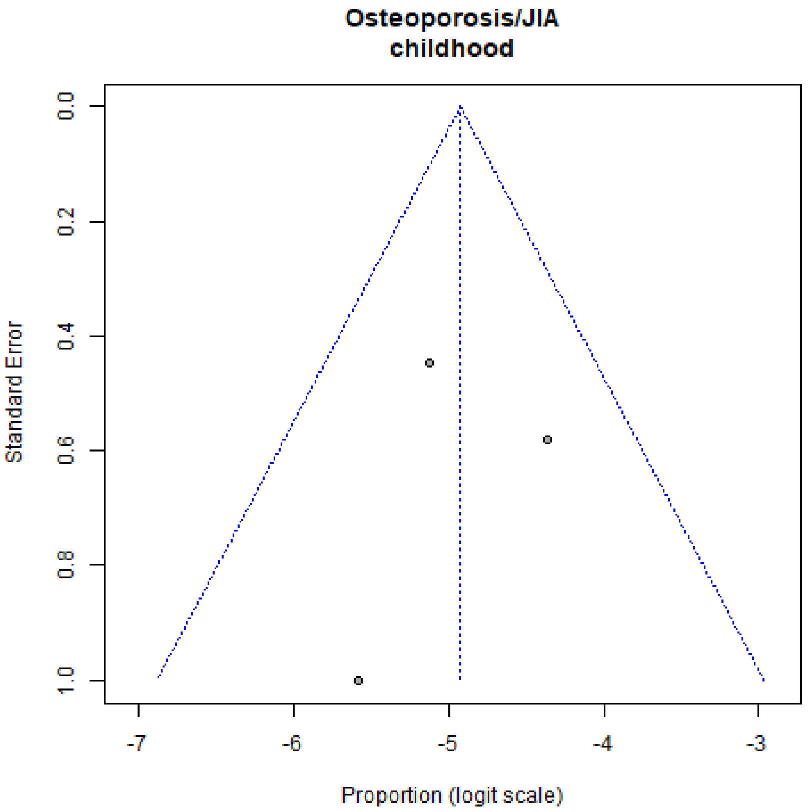

Summary statistics

```
                                              proportion           95%-CI %W(random)
Horneff G. etal, 2022 (InGef), Germany            0.0059 [0.0019; 0.0137]       55.7
Giancane G. etal, 2019 (MTX era), Italy           0.0126 [0.0026; 0.0362]       33.2
Giancane G. etal, 2019 (Biologic era), Italy      0.0037 [0.0001; 0.0205]       11.2
Number of studies: k = 3; Number of observations: o = 1357; Number of events: e = 9
                     proportion           95%-CI
Random effects model     0.0072 [0.0037; 0.0138]
Quantifying heterogeneity (with 95%-CIs):  tau^2 = 0 [0.0000; 14.5276]; tau = 0 [0.0000; 3.8115]
 I^2 = 0.0% [0.0%; 89.6%]; H = 1.00 [1.00; 3.10]
Test of heterogeneity:      Q d.f. p-value
                         | 1.58    2  0.4547
```

Supplementary Figure S23: Forest and Funnel Plots of studies reporting the prevalence of psoriasis in children with Juvenile Idiopathic Arthritis

Psoriasis

Forest

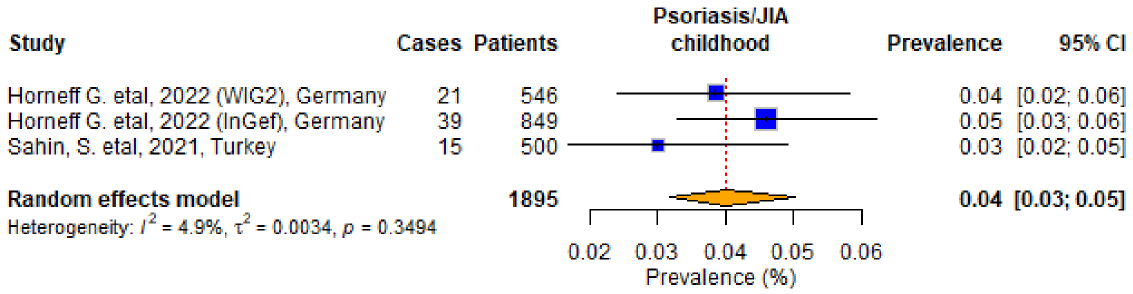

Funnel

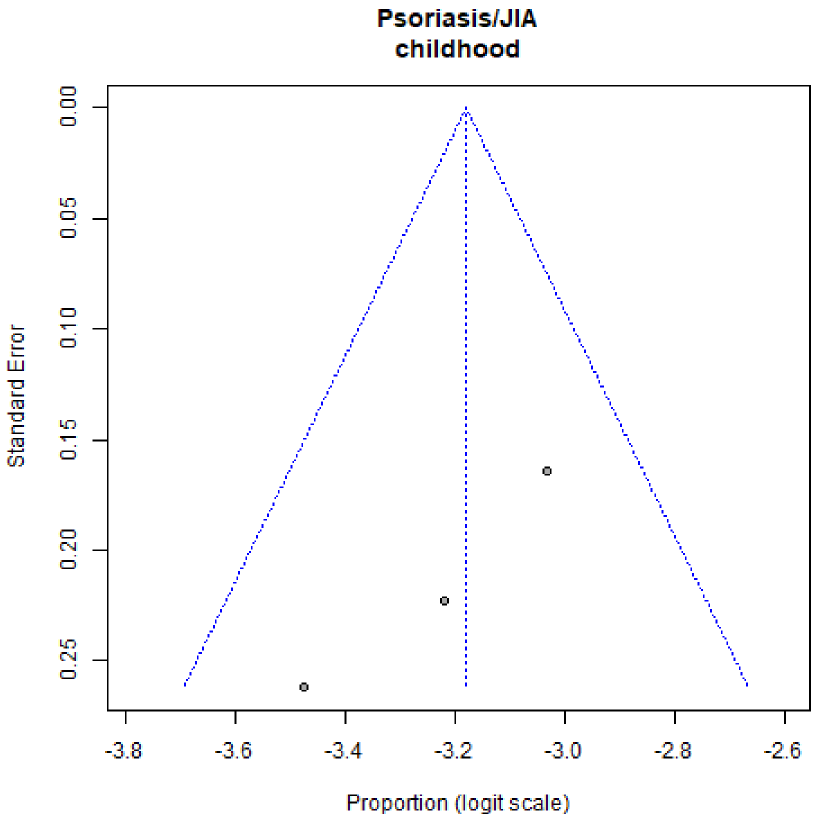

Summary statistics

```
                                       proportion           95%-CI %W(random)
Horneff G. etal, 2022 (WIG2), Germany      0.0385 [0.0240; 0.0582]       28.7
Horneff G. etal, 2022 (InGef), Germany     0.0459 [0.0329; 0.0623]       50.2
Sahin, S. etal, 2021, Turkey               0.0300 [0.0169; 0.0490]       21.1
Number of studies: k = 3; Number of observations: o = 1895; Number of events: e = 75
                     proportion           95%-CI
Random effects model     0.0399 [0.0316; 0.0503]
Quantifying heterogeneity (with 95%-CIs):  tau^2 = 0.0034 [0.0000; 1.9016]; tau = 0.0579 [0.0000; 1.3790]
 I^2 = 4.9% [0.0%; 90.1%]; H = 1.03 [1.00; 3.18]
Test of heterogeneity:     Q d.f. p-value
                        2.10    2  0.3494
```

Supplementary Figure S24: Forest and Funnel Plots of studies reporting the prevalence of synechiae in children with Juvenile Idiopathic Arthritis

Synechiae

Forest

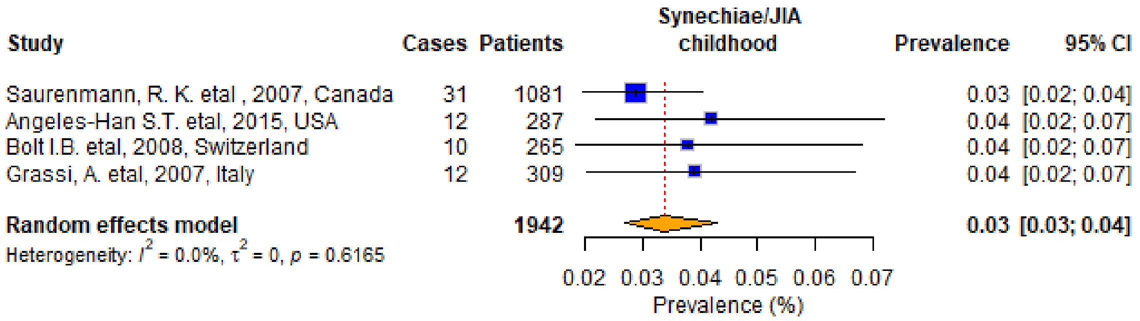

Funnel

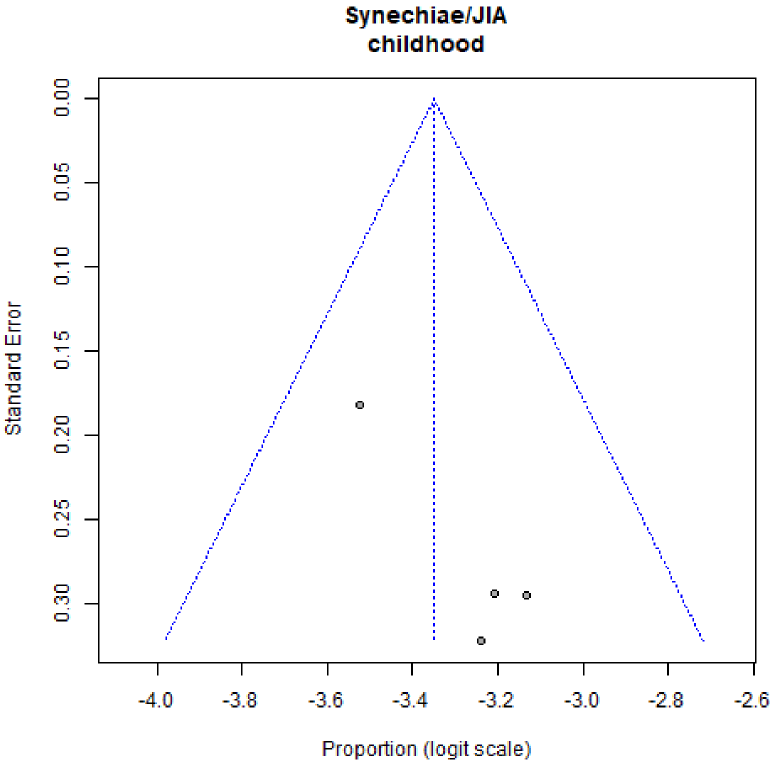

Summary statistics

```
                                       proportion           95%-CI %W(random)
Saurenmann, R. K. etal , 2007, Canada      0.0287 [0.0196; 0.0405]       48.0
Angeles-Han S.T. etal, 2015, USA           0.0418 [0.0218; 0.0719]       18.3
Bolt I.B. etal, 2008, Switzerland          0.0377 [0.0182; 0.0683]       15.3
Grassi, A. etal, 2007, Italy               0.0388 [0.0202; 0.0669]       18.4
Number of studies: k = 4; Number of observations: o = 1942; Number of events: e = 65
                      proportion           95%-CI
Random effects model      0.0339 [0.0267; 0.0430]
Quantifying heterogeneity (with 95%-CIs):  tau^2 = 0 [0.0000; 0.3558]; tau = 0 [0.0000; 0.5965]
 I^2 = 0.0% [0.0%; 84.7%]; H = 1.00 [1.00; 2.56]
Test of heterogeneity:      Q d.f. p-value
                         1.79    3  0.6165
```

Supplementary Figure S25: Forest and Funnel Plots of studies reporting the prevalence of Type 1 diabetes in children with Juvenile Idiopathic Arthritis

Type 1 Diabetes

Forest

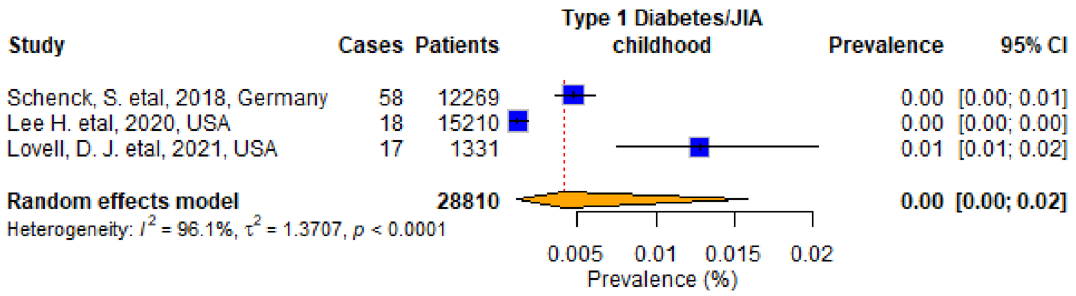

Funnel

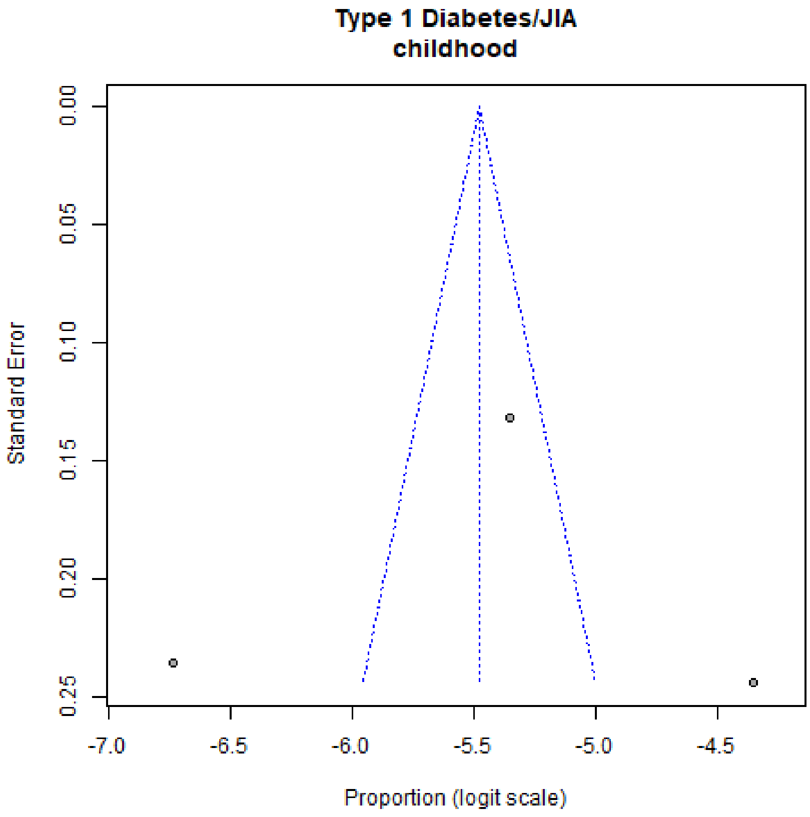

Summary statistics

```
                                 proportion           95%-CI %W(random)
Schenck, S. etal, 2018, Germany      0.0047 [0.0036; 0.0061]       34.0
Lee H. etal, 2020, USA               0.0012 [0.0007; 0.0019]       33.1
Lovell, D. J. etal, 2021, USA        0.0128 [0.0075; 0.0204]       33.0
Number of studies: k = 3; Number of observations: o = 28810; Number of events: e = 93
                      proportion           95%-CI
Random effects model      0.0042 [0.0011; 0.0158]
Quantifying heterogeneity (with 95%-CIs):  tau^2 = 1.3707 [0.3335; 56.8637]; tau = 1.1708 [0.5775; 7.5408]
 I^2 = 96.1% [91.6%; 98.2%]; H = 5.04 [3.45; 7.37]
Test of heterogeneity:       Q d.f.  p-value
                         50.80    2 < 0.0001
```

Supplementary Figure S26: Forest and Funnel Plots of studies reporting the prevalence of tuberculosis in children with Juvenile Idiopathic Arthritis

Tuberculosis/TB

Forest

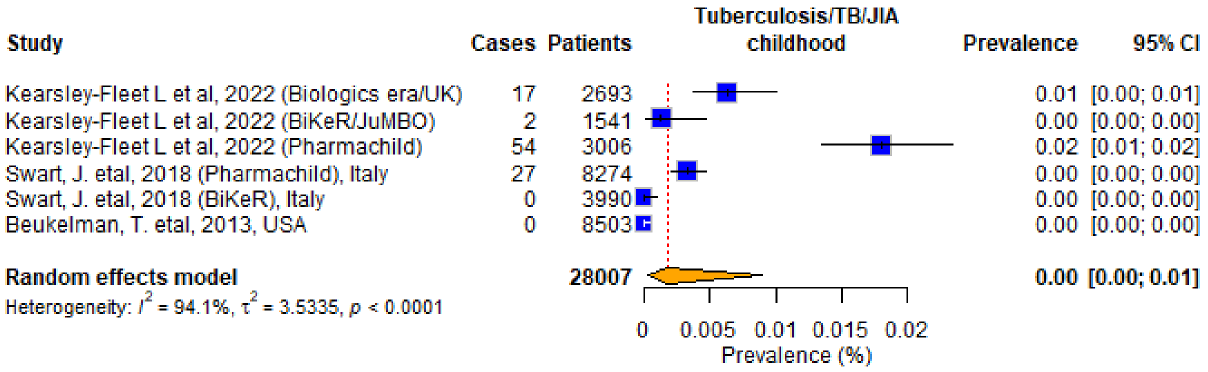

Funnel

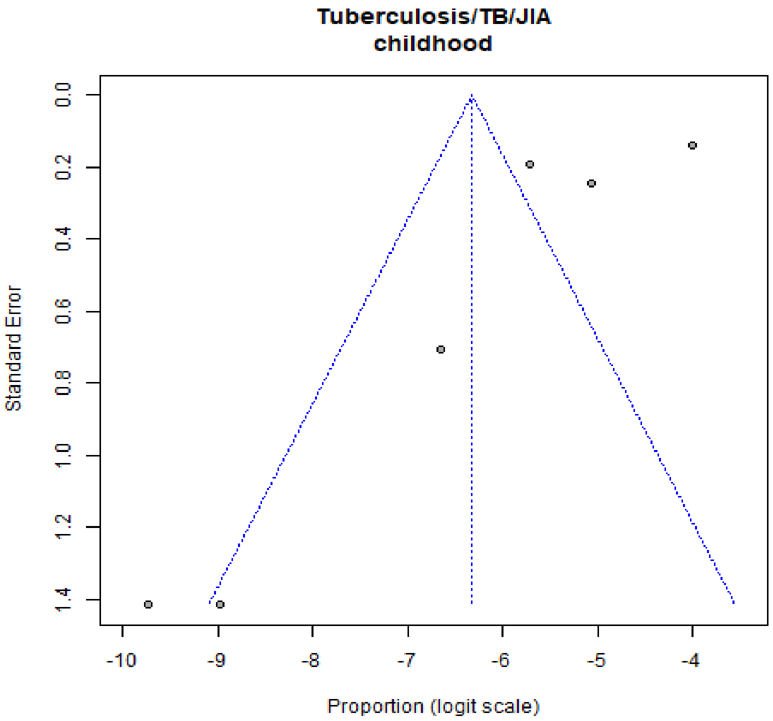

Summary statistics

```
                                                proportion           95%-CI %W(random)
Kearsley-Fleet L et al, 2022 (Biologics era/UK)     0.0063 [0.0037; 0.0101]       19.2
Kearsley-Fleet L et al, 2022 (BiKeR/JuMBO)          0.0013 [0.0002; 0.0047]       17.1
Kearsley-Fleet L et al, 2022 (Pharmachild)          0.0180 [0.0135; 0.0234]       19.4
Swart, J. etal, 2018 (Pharmachild), Italy           0.0033 [0.0022; 0.0047]       19.3
Swart, J. etal, 2018 (BiKeR), Italy                 0.0000 [0.0000; 0.0009]       12.5
Beukelman, T. etal, 2013, USA                       0.0000 [0.0000; 0.0004]       12.5
Number of studies: k = 6; Number of observations: o = 28007; Number of events: e = 100
                     proportion           95%-CI
Random effects model     0.0018 [0.0004; 0.0090]
Quantifying heterogeneity (with 95%-CIs):  tau^2 = 3.5335 [0.9812; 29.4146]; tau = 1.8797 [0.9905; 5.4235]
 I^2 = 94.1% [89.8%; 96.6%]; H = 4.13 [3.13; 5.45]
Test of heterogeneity:         Q d.f.  p-value
                           85.36    5 < 0.0001
```

Supplementary Figure S27: Forest and Funnel Plots of studies reporting the prevalence of uveitis in children with Juvenile Idiopathic Arthritis

Uveitis

Forest

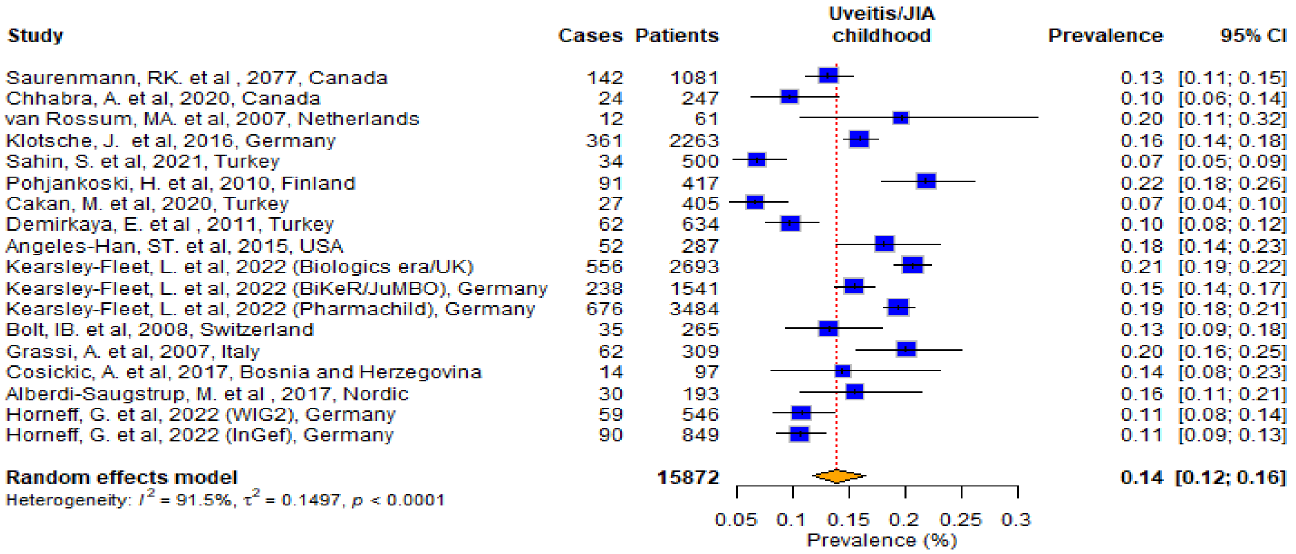

Funnel

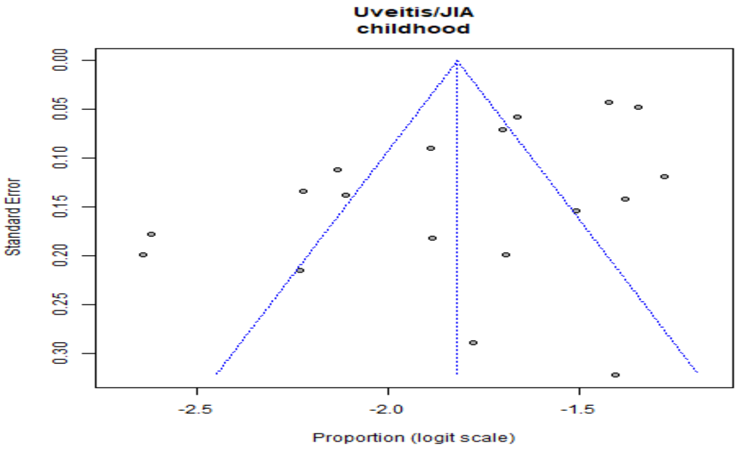

Summary statistics

```
                                                         proportion           95%-CI %W(random)
Saurenmann, RK. et al , 2077, Canada                         0.1314 [0.1118; 0.1530]        6.1
Chhabra, A. et al, 2020, Canada                              0.0972 [0.0633; 0.1411]        4.9
van Rossum, MA. et al, 2007, Netherlands                     0.1967 [0.1060; 0.3184]        3.8
Klotsche, J.  et al, 2016, Germany                           0.1595 [0.1447; 0.1753]        6.3
Sahin, S. et al, 2021, Turkey                                0.0680 [0.0475; 0.0937]        5.3
Pohjankoski, H. et al, 2010, Finland                         0.2182 [0.1795; 0.2610]        5.9
Cakan, M. et al, 2020, Turkey                                0.0667 [0.0444; 0.0955]        5.1
Demirkaya, E. et al , 2011, Turkey                           0.0978 [0.0758; 0.1236]        5.8
Angeles-Han, ST. et al, 2015, USA                            0.1812 [0.1384; 0.2307]        5.6
Kearsley-Fleet, L. et al, 2022 (Biologics era/UK)            0.2065 [0.1913; 0.2222]        6.4
Kearsley-Fleet, L. et al, 2022 (BiKeR/JuMBO), Germany        0.1544 [0.1367; 0.1735]        6.3
Kearsley-Fleet, L. et al, 2022 (Pharmachild), Germany        0.1940 [0.1810; 0.2076]        6.4
Bolt, IB. et al, 2008, Switzerland                           0.1321 [0.0937; 0.1789]        5.3
Grassi, A. et al, 2007, Italy                                0.2006 [0.1574; 0.2497]        5.7
Cosickic, A. et al, 2017, Bosnia and Herzegovina             0.1443 [0.0812; 0.2303]        4.2
Alberdi-Saugstrup, M. et al , 2017, Nordic                   0.1554 [0.1074; 0.2144]        5.1
Horneff, G. et al, 2022 (WIG2), Germany                      0.1081 [0.0833; 0.1372]        5.7
Horneff, G. et al, 2022 (InGef), Germany                     0.1060 [0.0861; 0.1287]        6.0
Number of studies: k = 18; Number of observations: o = 15872; Number of events: e = 2565
                     proportion           95%-CI
Random effects model     0.1395 [0.1179; 0.1643]
Quantifying heterogeneity (with 95%-CIs):  tau^2 = 0.1497 [0.0741; 0.3706]; tau = 0.3870 [0.2721; 0.6088]
 I^2 = 91.5% [88.0%; 93.9%]; H = 3.42 [2.89; 4.05]
Test of heterogeneity:       Q d.f.  p-value
                      | 198.93   17 < 0.0001
```

Supplementary Figure S28: Forest and Funnel Plots of studies reporting the prevalence of varicella in children with Juvenile Idiopathic Arthritis

Varicella

Forest

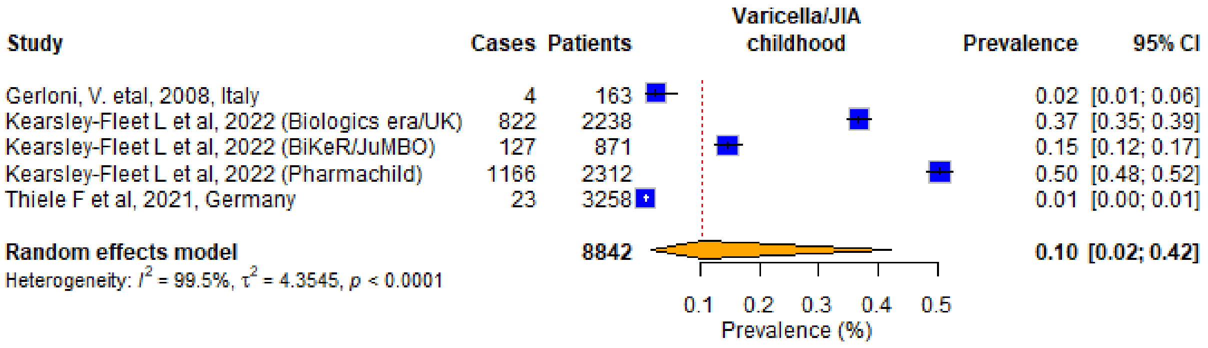

Funnel

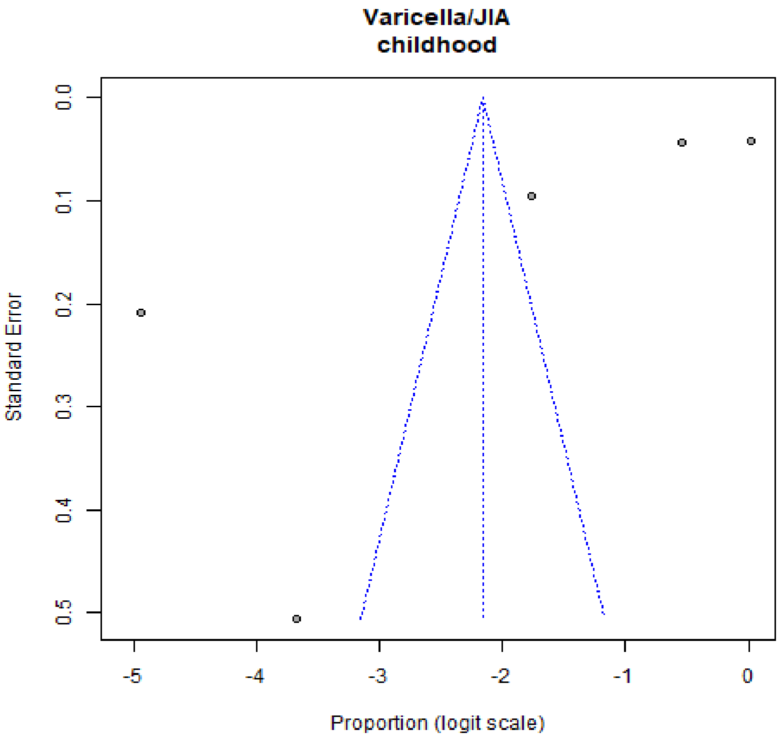

Summary statistics

```
                                                proportion           95%-CI %W(random)
Gerloni, V. etal, 2008, Italy                       0.0245 [0.0067; 0.0616]       19.2
Kearsley-Fleet L et al, 2022 (Biologics era/UK)     0.3673 [0.3473; 0.3877]       20.3
Kearsley-Fleet L et al, 2022 (BiKeR/JuMBO)          0.1458 [0.1230; 0.1710]       20.2
Kearsley-Fleet L et al, 2022 (Pharmachild)          0.5043 [0.4837; 0.5249]       20.3
Thiele F et al, 2021, Germany                       0.0071 [0.0045; 0.0106]       20.1
Number of studies: k = 5; Number of observations: o = 8842; Number of events: e = 2142
                     proportion           95%-CI
Random effects model     0.1031 [0.0179; 0.4204]
Quantifying heterogeneity (with 95%-CIs):  tau^2 = 4.3545 [1.5277; 36.2489]; tau = 2.0868 [1.2360; 6.0207]
 I^2 = 99.5% [99.4%; 99.6%]; H = 14.32 [12.48; 16.44]
Test of heterogeneity:         Q d.f.  p-value
                          820.56    4 < 0.0001
```

JIA/Adulthood

Supplementary Figure S29: Forest and Funnel Plots of studies reporting the prevalence of anxiety in adults with Juvenile Idiopathic Arthritis

Anxiety

Forest

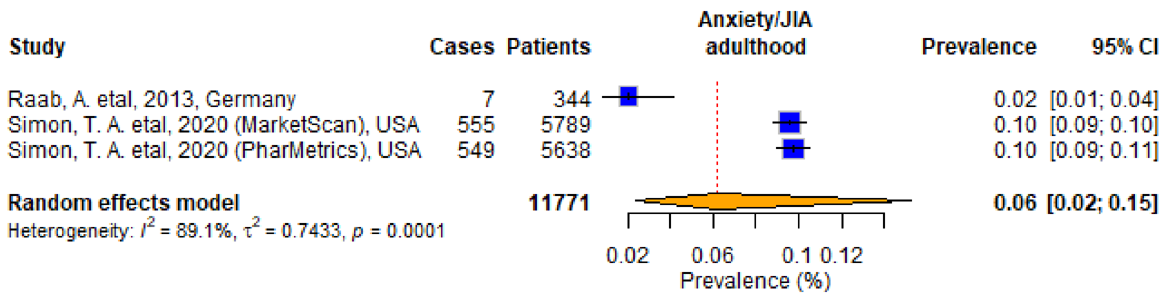

Funnel

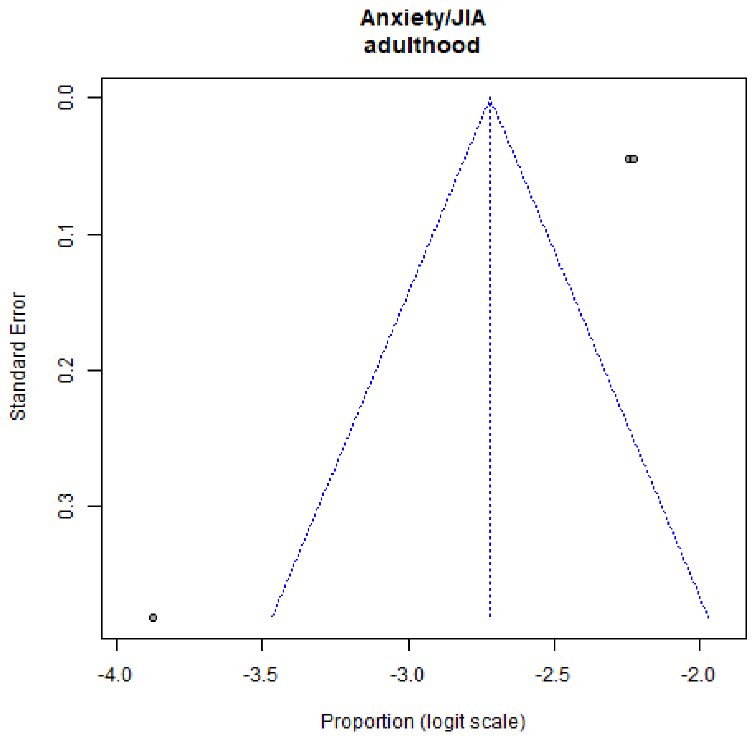

Summary statistics

```
                                            proportion           95%-CI %W(random)
Raab, A. etal, 2013, Germany                    0.0203 [0.0082; 0.0415]       29.5
Simon, T. A. etal, 2020 (MarketScan), USA       0.0959 [0.0884; 0.1037]       35.2
Simon, T. A. etal, 2020 (PharMetrics), USA      0.0974 [0.0898; 0.1054]       35.2
Number of studies: k = 3; Number of observations: o = 11771; Number of events: e = 1111
                     proportion           95%-CI
Random effects model     0.0618 [0.0236; 0.1525]
Quantifying heterogeneity (with 95%-CIs):  tau^2 = 0.7433 [0.1448; 35.2654]; tau = 0.8622 [0.3806; 5.9385]
 I^2 = 89.1% [70.3%; 96.0%]; H = 3.03 [1.83; 5.01]
Test of heterogeneity:       Q d.f. p-value
                         18.36    2  0.0001
```

Supplementary Figure S30: Forest and Funnel Plots of studies reporting the prevalence of asthma in adults with Juvenile Idiopathic Arthritis

Asthma

Forest

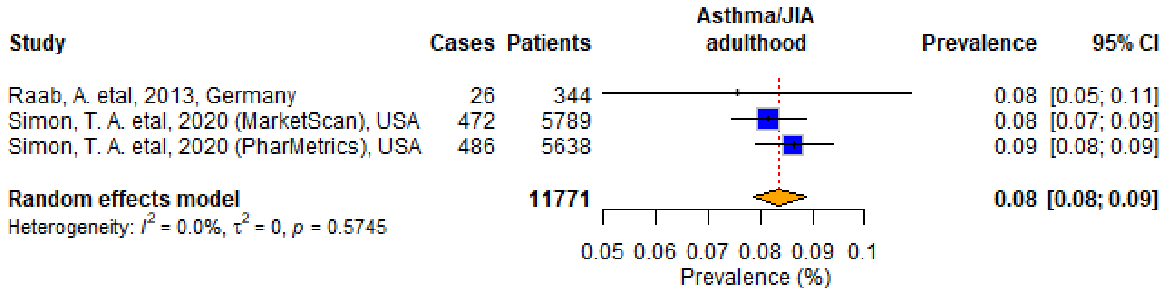

Funnel

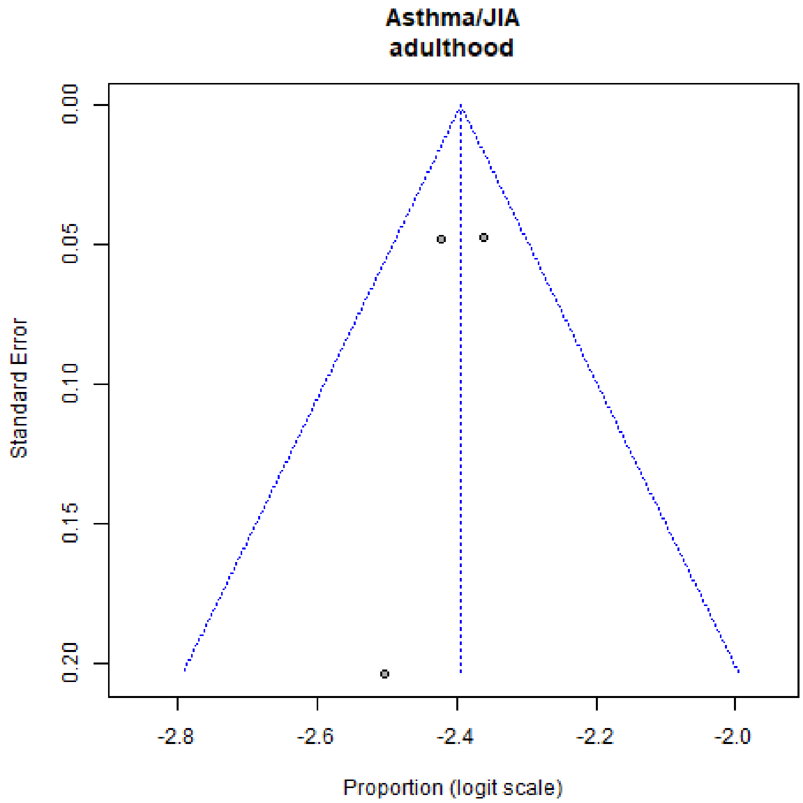

Summary statistics

```
                                              proportion           95%-CI %W(random)
Raab, A. etal, 2013, Germany                      0.0756 [0.0500; 0.1088]        2.7
Simon, T. A. etal, 2020 (MarketScan), USA         0.0815 [0.0746; 0.0889]       48.1
Simon, T. A. etal, 2020 (PharMetrics), USA        0.0862 [0.0790; 0.0938]       49.3
Number of studies: k = 3; Number of observations: o = 11771; Number of events: e = 984
                      proportion           95%-CI
Random effects model      0.0836 [0.0788; 0.0888]
Quantifying heterogeneity (with 95%-CIs):  tau^2 = 0 [0.0000; 0.1803]; tau = 0 [0.0000; 0.4246]
 I^2 = 0.0% [0.0%; 89.6%]; H = 1.00 [1.00; 3.10]
Test of heterogeneity:     Q d.f. p-value
                         1.11    2  0.5745
```

Supplementary Figure S31: Forest and Funnel Plots of studies reporting the prevalence of cataract in adults with Juvenile Idiopathic Arthritis

Cataract

Forest

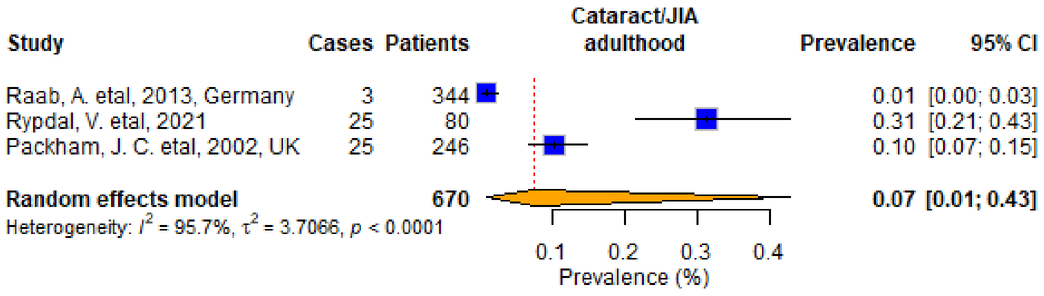

Funnel

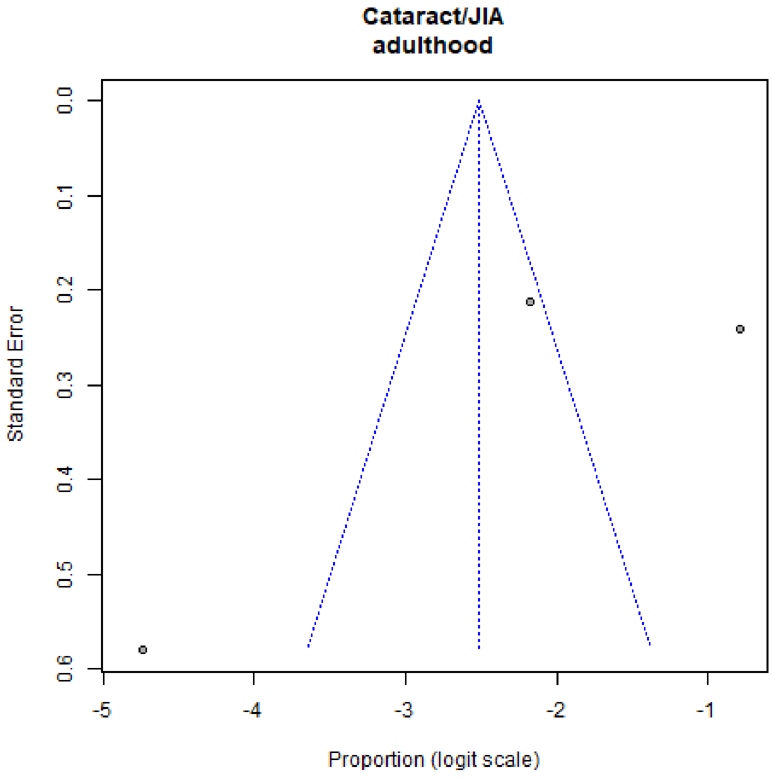

Summary statistics

```
                                proportion           95%-CI %W(random)
Raab, A. etal, 2013, Germany        0.0087 [0.0018; 0.0253]       31.7
Rypdal, V. etal, 2021               0.3125 [0.2135; 0.4259]       34.1
Packham, J. C. etal, 2002, UK       0.1016 [0.0669; 0.1464]       34.2
Number of studies: k = 3
Number of observations: o = 670
Number of events: e = 53
                       proportion           95%-CI
Random effects model      0.0748 [0.0087; 0.4266]
Quantifying heterogeneity (with 95%-CIs):  tau^2 = 3.7066 [0.8680; >100.0000]; tau = 1.9252 [0.9316; >10.0000]
 I^2 = 95.7% [90.6%; 98.0%]; H = 4.82 [3.26; 7.11]
Test of heterogeneity:      Q d.f.  p-value
                        46.40    2 < 0.0001
```

Supplementary Figure S32: Forest and Funnel Plots of studies reporting the prevalence of osteoporosis in adults with Juvenile Idiopathic Arthritis

Osteoporosis

Forest

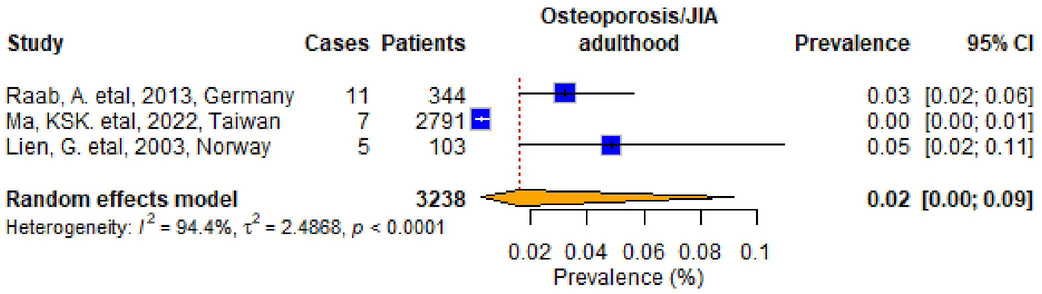

Funnel

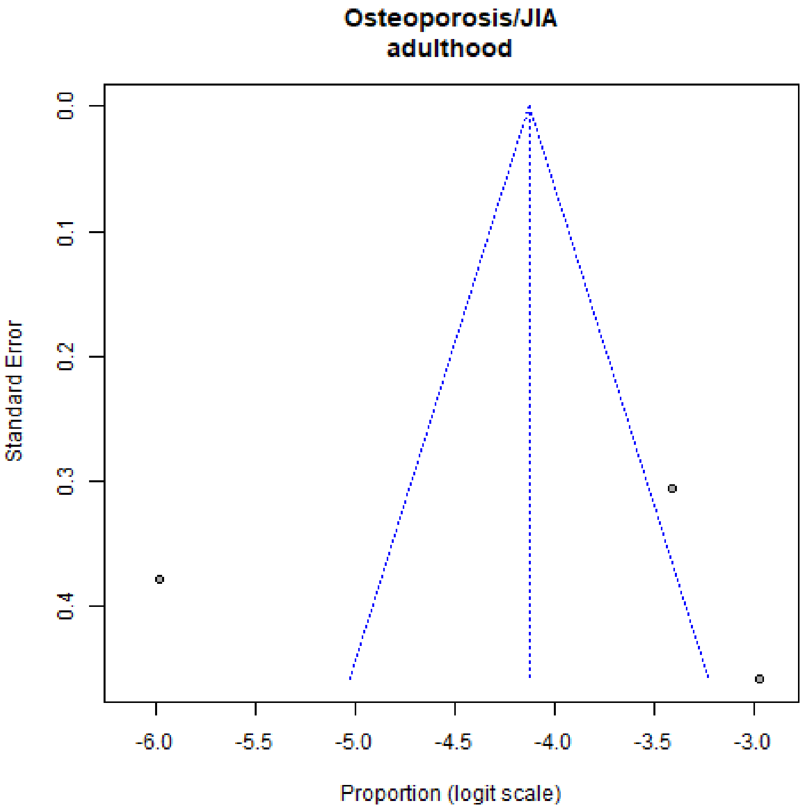

Summary statistics

```
                              proportion           95%-CI %W(random)
Raab, A. etal, 2013, Germany      0.0320 [0.0161; 0.0565]       34.0
Ma, KSK. etal, 2022, Taiwan       0.0025 [0.0010; 0.0052]       33.4
Lien, G. etal, 2003, Norway       0.0485 [0.0159; 0.1097]       32.6
Number of studies: k = 3; Number of observations: o = 3238; Number of events: e = 23
                      proportion           95%-CI
Random effects model      0.0158 [0.0026; 0.0918]
Quantifying heterogeneity (with 95%-CIs):  tau^2 = 2.4868 [0.5640; >100.0000]; tau = 1.5770 [0.7510; >10.0000]
 I^2 = 94.4% [87.1%; 97.6%]; H = 4.24 [2.78; 6.45]
Test of heterogeneity:       Q d.f.  p-value
                         35.90    2 < 0.0001
```

Supplementary Figure S33: Forest and Funnel Plots of studies reporting the prevalence of depression in adults with Juvenile Idiopathic Arthritis

Depression

Forest

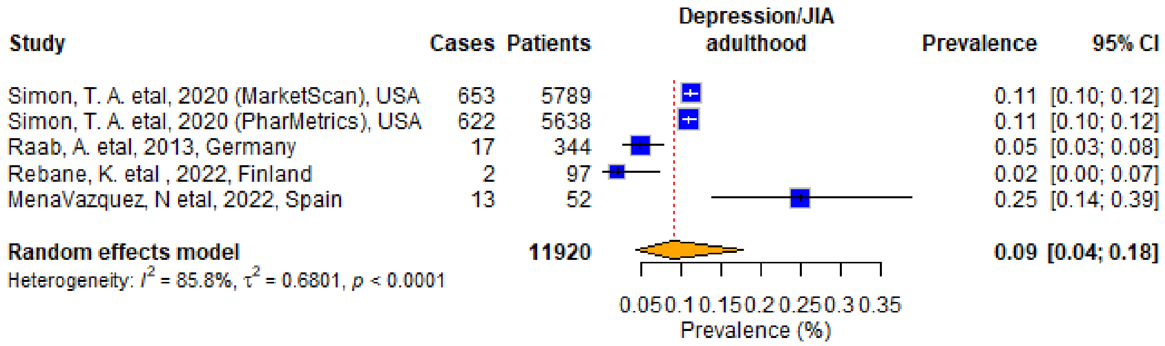

Funnel

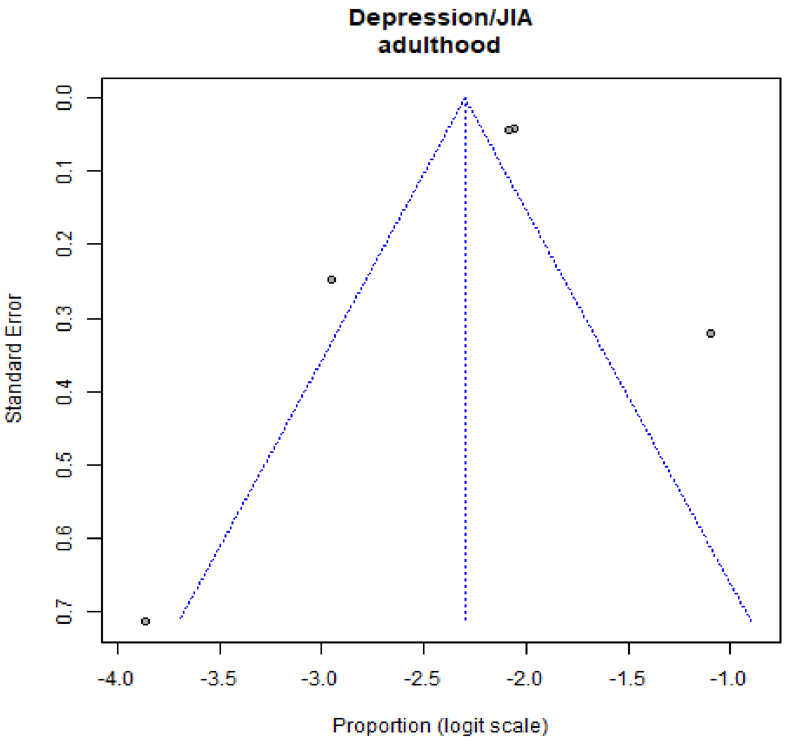

Summary statistics

```
                                             proportion           95%-CI %W(random)
Simon, T. A. etal, 2020 (MarketScan), USA       0.1128 [0.1048; 0.1212]       22.9
Simon, T. A. etal, 2020 (PharMetrics), USA      0.1103 [0.1023; 0.1188]       22.9
Raab, A. etal, 2013, Germany                    0.0494 [0.0290; 0.0779]       21.1
Rebane, K. etal , 2022, Finland                 0.0206 [0.0025; 0.0725]       13.1
MenaVazquez, N etal, 2022, Spain                0.2500 [0.1403; 0.3895]       20.0
Number of studies: k = 5; Number of observations: o = 11920; Number of events: e = 1307
                     proportion           95%-CI
Random effects model     0.0911 [0.0442; 0.1787]
Quantifying heterogeneity (with 95%-CIs):  tau^2 = 0.6801 [0.1703; 8.6878]; tau = 0.8247 [0.4127; 2.9475]
 I^2 = 85.8% [68.9%; 93.5%]; H = 2.66 [1.79; 3.94]
Test of heterogeneity:      Q d.f.  p-value
                        28.23    4 < 0.0001
```

Supplementary Figure S34: Forest and Funnel Plots of studies reporting the prevalence of diabetes (undefined) in adults with Juvenile Idiopathic Arthritis

Diabetes (undefined)

Forest

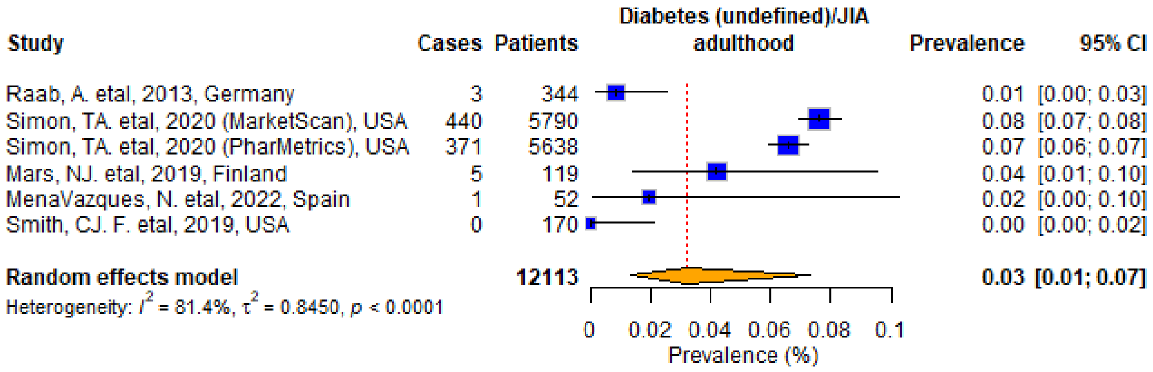

Funnel

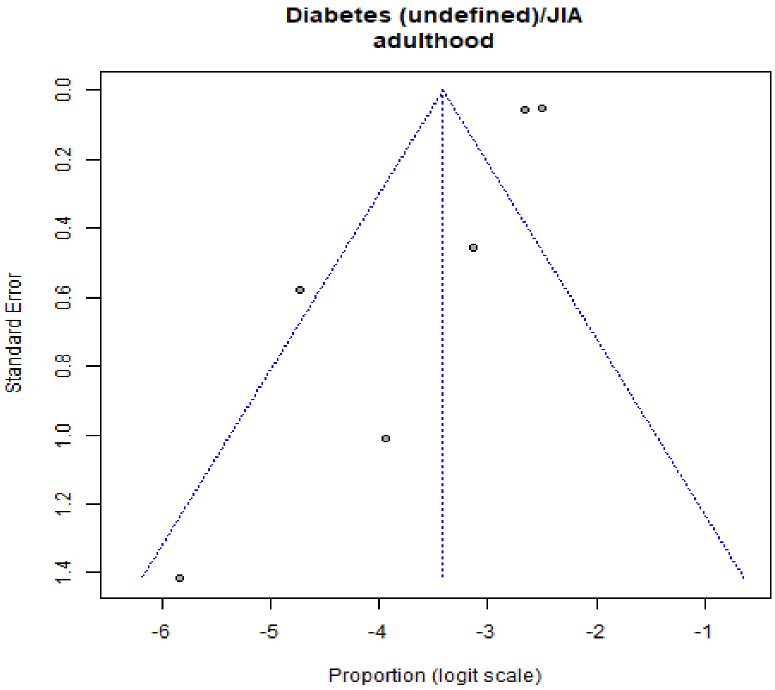

Summary statistics

```
                                          proportion           95%-CI %W(random)
Raab, A. etal, 2013, Germany                  0.0087 [0.0018; 0.0253]       16.8
Simon, TA. etal, 2020 (MarketScan), USA       0.0760 [0.0693; 0.0831]       23.4
Simon, TA. etal, 2020 (PharMetrics), USA      0.0658 [0.0595; 0.0726]       23.4
Mars, NJ. etal, 2019, Finland                 0.0420 [0.0138; 0.0953]       18.8
MenaVazques, N. etal, 2022, Spain             0.0192 [0.0005; 0.1026]       10.6
Smith, CJ. F. etal, 2019, USA                 0.0000 [0.0000; 0.0215]        7.0
Number of studies: k = 6; Number of observations: o = 12113; Number of events: e = 820
                      proportion           95%-CI
Random effects model      0.0319 [0.0136; 0.0731]
Quantifying heterogeneity (with 95%-CIs):  tau^2 = 0.8450 [0.1589; 9.2267]; tau = 0.9192 [0.3986; 3.0375]
 I^2 = 81.4% [60.1%; 91.3%]; H = 2.32 [1.58; 3.39]
Test of heterogeneity:       Q d.f.  p-value
                         26.84    5 < 0.0001
```

Supplementary Figure S35: Forest and Funnel Plots of studies reporting the prevalence of glaucoma in adults with Juvenile Idiopathic Arthritis

Glaucoma

Forest

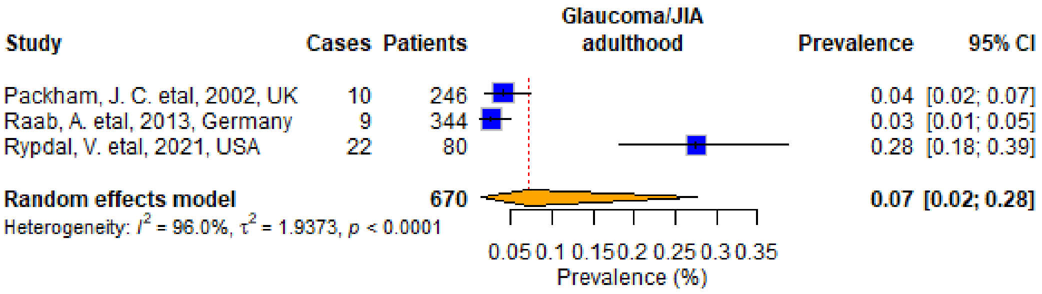

Funnel

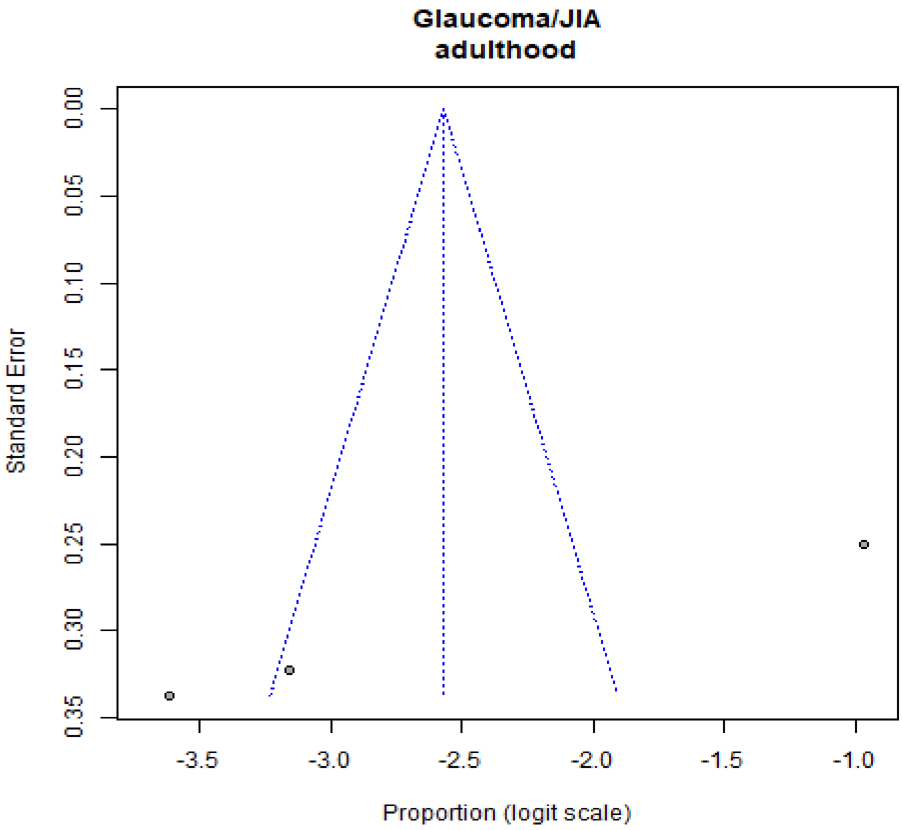

Summary statistics

```
                              proportion           95%-CI %W(random)
Packham, J. C. etal, 2002, UK     0.0407 [0.0197; 0.0735]       33.2
Raab, A. etal, 2013, Germany      0.0262 [0.0120; 0.0491]       33.0
Rypdal, V. etal, 2021, USA        0.2750 [0.1810; 0.3862]       33.8
Number of studies: k = 3; Number of observations: o = 670; Number of events: e = 41
                     proportion           95%-CI
Random effects model     0.0711 [0.0150; 0.2774]
Quantifying heterogeneity (with 95%-CIs):  tau^2 = 1.9373 [0.4633; 79.0548]; tau = 1.3919 [0.6807; 8.8913]
 I^2 = 96.0% [91.5%; 98.1%]; H = 5.02 [3.43; 7.35]
Test of heterogeneity:       Q d.f.  p-value
                         50.50    2 < 0.0001
```

Supplementary Figure S36: Forest and Funnel Plots of studies reporting the prevalence of hypertension in adults with Juvenile Idiopathic Arthritis

Hypertension

Forest

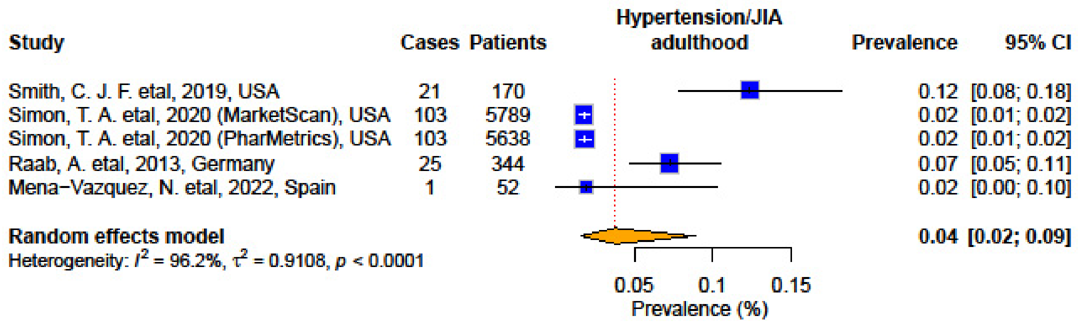

Funnel

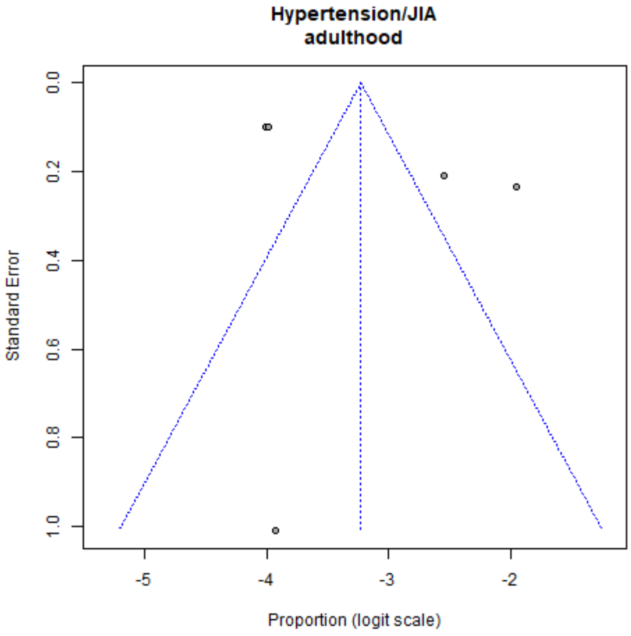

Summary statistics

```
                                           proportion   95% CI                 %W(random)
Smith, C. J. F. etal, 2019, USA                0.1235   [0.0781; 0.1826]       21.7
Simon, T. A. etal, 2020 (MarketScan), USA      0.0178   [0.0145; 0.0215]       22.7
Simon, T. A. etal, 2020 (PharMetrics), USA     0.0183   [0.0149; 0.0221]       22.7
Raab, A. etal, 2013, Germany                   0.0727   [0.0476; 0.1054]       22.0
Mena-Vazquez, N. etal, 2022, Spain             0.0192   [0.0005; 0.1026]       10.8

Number of studies: k = 5
Number of observations: o = 11993
Number of events: e = 253

                     proportion 95% CI
Random effects model     0.0381 [0.0159; 0.0885]

Quantifying heterogeneity (with 95% CIs):
 tau^2 = 0.9108 [0.2678; 7.5758]; tau = 0.9544 [0.5175; 2.7524]
 I^2 = 96.2% [93.4%; 97.8%]; H = 5.11 [3.90; 6.70]

Test of heterogeneity:
      Q d.f.  p-value
 104.61    4 < 0.0001
```

Supplementary Figure S37: Forest and Funnel Plots of studies reporting the prevalence of malignancy in adults with Juvenile Idiopathic Arthritis

Malignancy

Forest

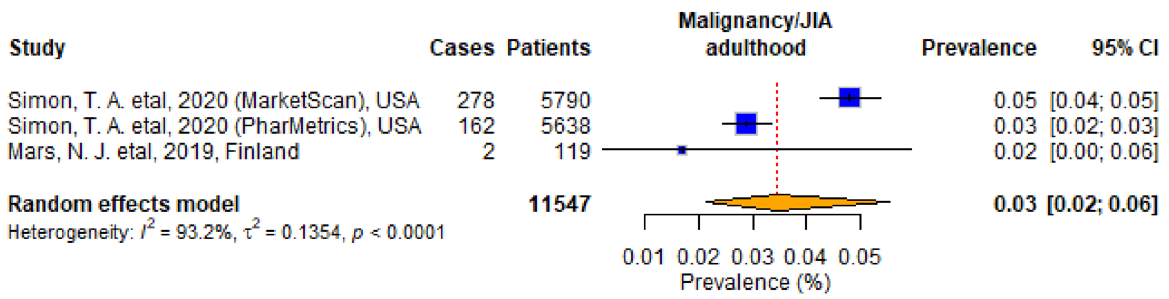

Funnel

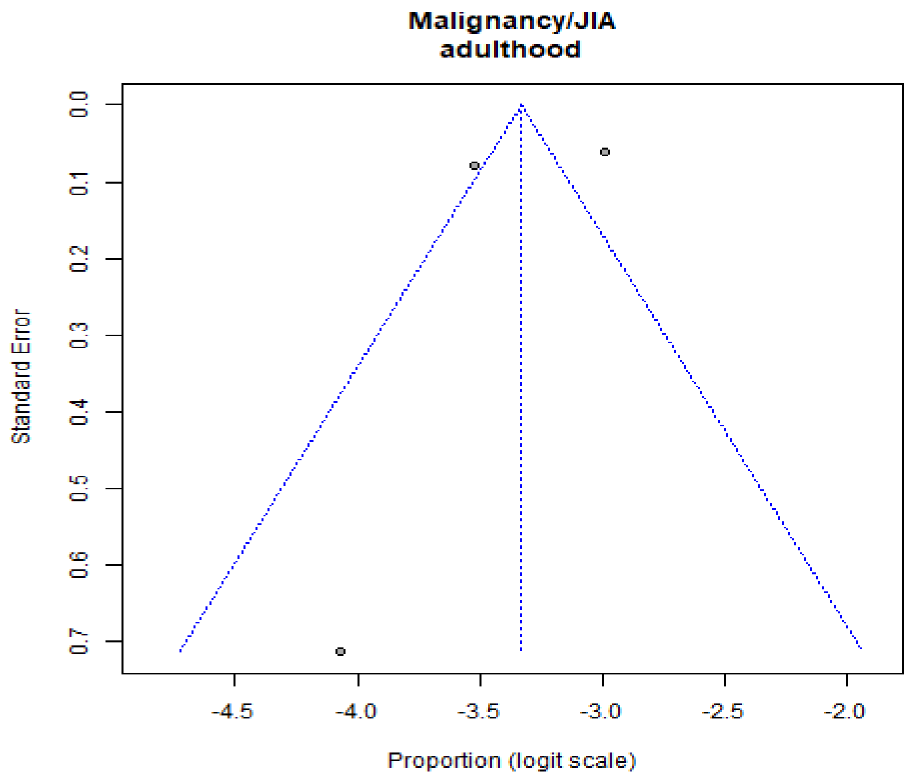

Summary statistics

```
                                           proportion           95%-CI %W(random)
Simon, T. A. etal, 2020 (MarketScan), USA      0.0480 [0.0426; 0.0538]       45.5
Simon, T. A. etal, 2020 (PharMetrics), USA     0.0287 [0.0245; 0.0334]       44.7
Mars, N. J. etal, 2019, Finland                0.0168 [0.0020; 0.0594]        9.8
Number of studies: k = 3; Number of observations: o = 11547; Number of events: e = 442
                     proportion           95%-CI
Random effects model     0.0345 [0.0214; 0.0553]
Quantifying heterogeneity (with 95%-CIs):  tau^2 = 0.1354 [0.0182; 11.3036]; tau = 0.3679 [0.1350; 3.3621]
 I^2 = 93.2% [83.6%; 97.2%]; H = 3.85 [2.47; 5.99]
Test of heterogeneity:       Q d.f.  p-value
                         29.61    2 < 0.0001
```

Supplementary Figure S38: Forest and Funnel Plots of studies reporting the prevalence of uveitis in adults with Juvenile Idiopathic Arthritis

Uveitis

Forest

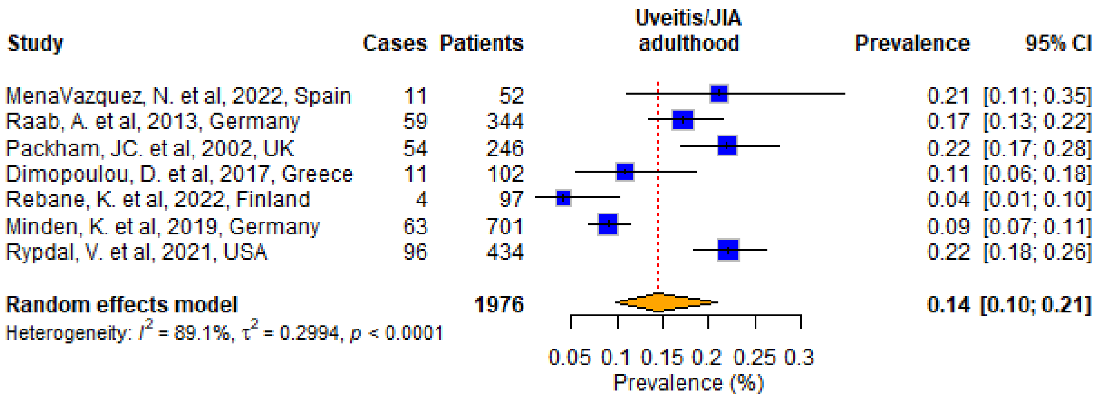

Funnel

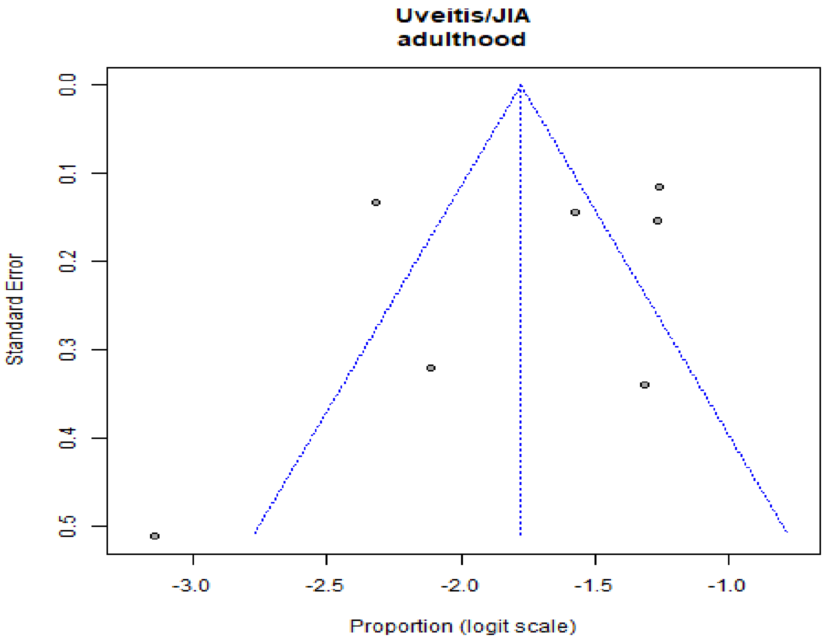

Summary statistics

```
                                   proportion           95%-CI %W(random)
MenaVazquez, N. et al, 2022, Spain     0.2115 [0.1106; 0.3470]       12.5
Raab, A. et al, 2013, Germany          0.1715 [0.1332; 0.2156]       16.2
Packham, JC. et al, 2002, UK           0.2195 [0.1694; 0.2765]       16.1
Dimopoulou, D. et al, 2017, Greece     0.1078 [0.0551; 0.1848]       12.9
Rebane, K. et al, 2022, Finland        0.0412 [0.0113; 0.1022]        9.3
Minden, K. et al, 2019, Germany        0.0899 [0.0698; 0.1135]       16.4
Rypdal, V. et al, 2021, USA            0.2212 [0.1830; 0.2632]       16.6
Number of studies: k = 7; Number of observations: o = 1976; Number of events: e = 298
                     proportion           95%-CI
Random effects model     0.1446 [0.0976; 0.2090]
Quantifying heterogeneity (with 95%-CIs):  tau^2 = 0.2994 [0.0932; 2.2766]; tau = 0.5472 [0.3053; 1.5089]
 I^2 = 89.1% [79.9%; 94.0%]; H = 3.02 [2.23; 4.09]
Test of heterogeneity:      Q d.f.  p-value
                        54.84    6 < 0.0001
```

jSLE/Childhood

Supplementary Figure S39: Forest and Funnel Plots of studies reporting the prevalence of anxiety in children with juvenile-onset Systemic Lupus Erythematosus

Anxiety

Forest

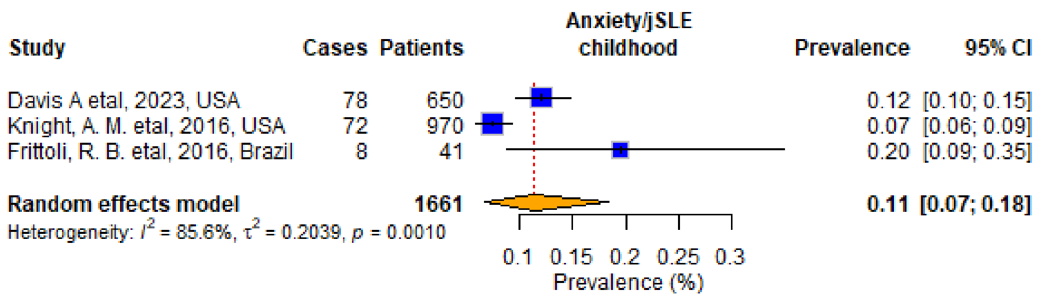

Funnel

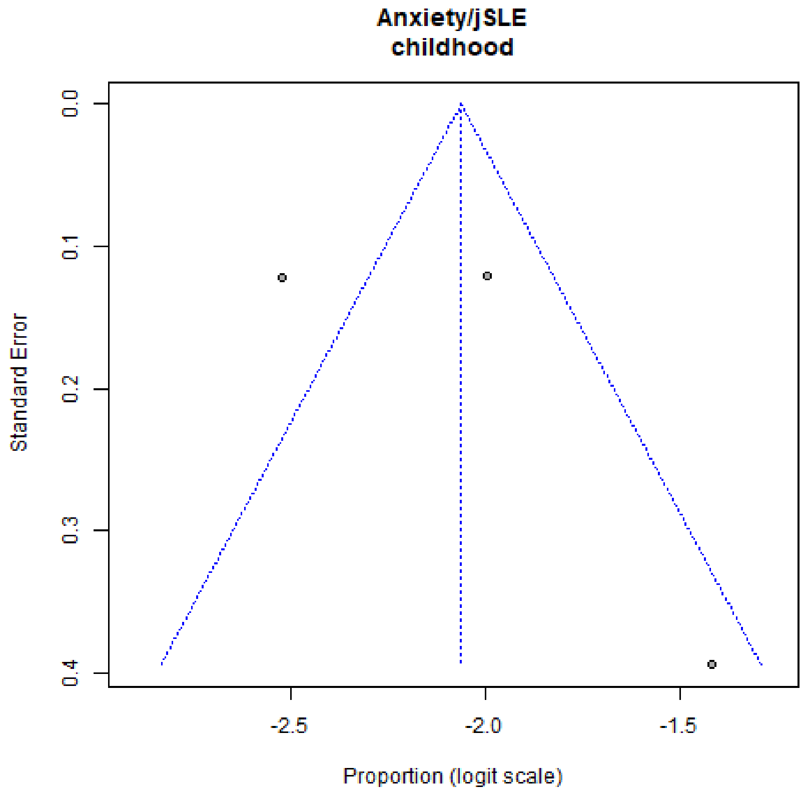

Summary statistics

```
                                    proportion           95%-CI %W(random)
Davis A etal, 2023, USA                 0.1200 [0.0960; 0.1475]       38.4
Knight, A. M. etal, 2016, USA           0.0742 [0.0585; 0.0926]       38.3
Frittoli, R. B. etal, 2016, Brazil      0.1951 [0.0882; 0.3487]       23.3
Number of studies: k = 3; Number of observations: o = 1661; Number of events: e = 158
                      proportion           95%-CI
Random effects model      0.1129 [0.0673; 0.1833]
Quantifying heterogeneity (with 95%-CIs):  tau^2 = 0.2039 [0.0224; 12.0079]; tau = 0.4515 [0.1498; 3.4652]
 I^2 = 85.6% [57.7%; 95.1%]; H = 2.63 [1.54; 4.50]
Test of heterogeneity:       Q d.f.  p-value
                         13.84    2   0.0010
```

Supplementary Figure S40: Forest and Funnel Plots of studies reporting the prevalence of avascular necrosis in children with juvenile-onset Systemic Lupus Erythematosus

Avascular necrosis

Forest

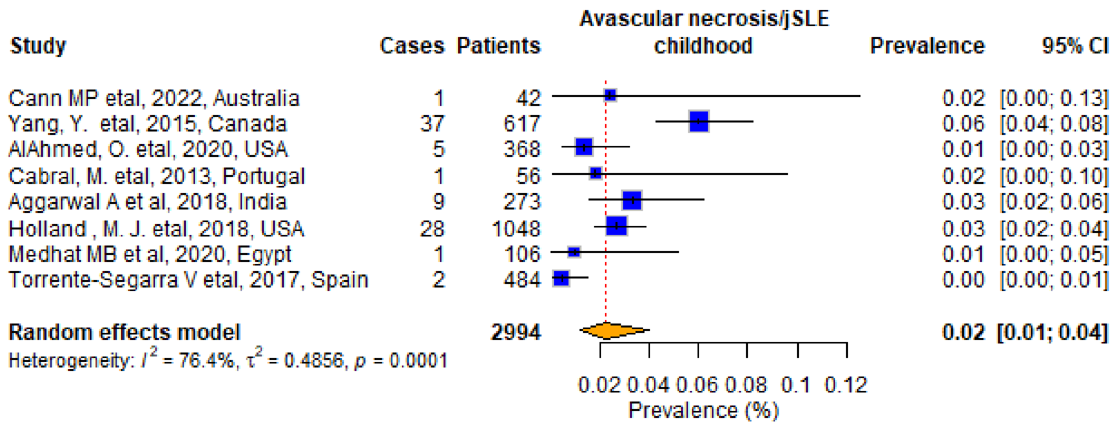

Funnel

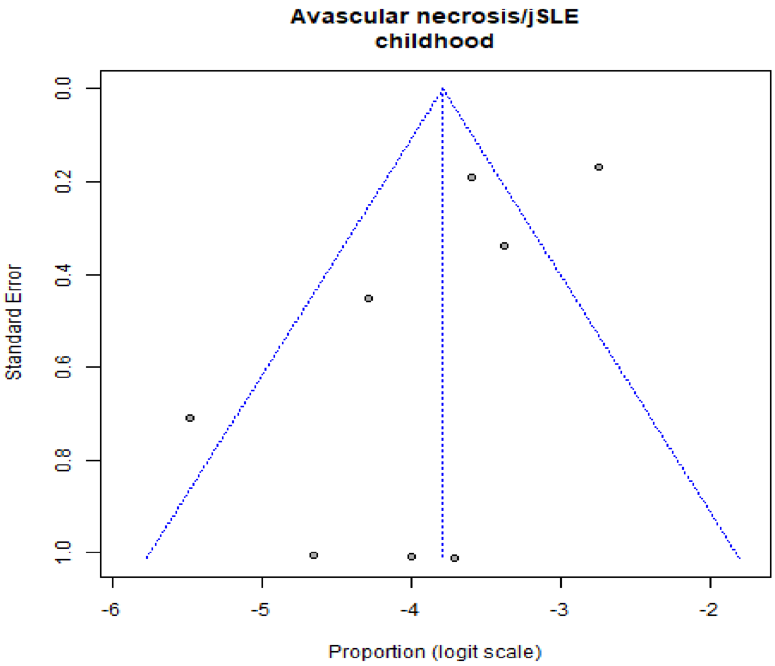

Summary statistics

```
                                    proportion           95%-CI %W(random)
Cann MP etal, 2022, Australia           0.0238 [0.0006; 0.1257]        6.6
Yang, Y.  etal, 2015, Canada            0.0600 [0.0426; 0.0817]       19.5
AlAhmed, O. etal, 2020, USA             0.0136 [0.0044; 0.0314]       14.5
Cabral, M. etal, 2013, Portugal         0.0179 [0.0005; 0.0955]        6.7
Aggarwal A et al, 2018, India           0.0330 [0.0152; 0.0617]       16.7
Holland , M. J. etal, 2018, USA         0.0267 [0.0178; 0.0384]       19.2
Medhat MB et al, 2020, Egypt            0.0094 [0.0002; 0.0514]        6.7
Torrente-Segarra V etal, 2017, Spain    0.0041 [0.0005; 0.0148]       10.1
Number of studies: k = 8; Number of observations: o = 2994; Number of events: e = 84
                     proportion           95%-CI
Random effects model     0.0220 [0.0120; 0.0402]
Quantifying heterogeneity (with 95%-CIs):  tau^2 = 0.4856 [0.0757; 2.5590]; tau = 0.6968 [0.2752; 1.5997]
 I^2 = 76.4% [52.8%; 88.2%]; H = 2.06 [1.46; 2.91]
Test of heterogeneity:      Q d.f.  p-value
                        29.62    7   0.0001
```

Supplementary Figure S41: Forest and Funnel Plots of studies reporting the prevalence of cataract in children with juvenile-onset Systemic Lupus Erythematosus

Cataract

Forest

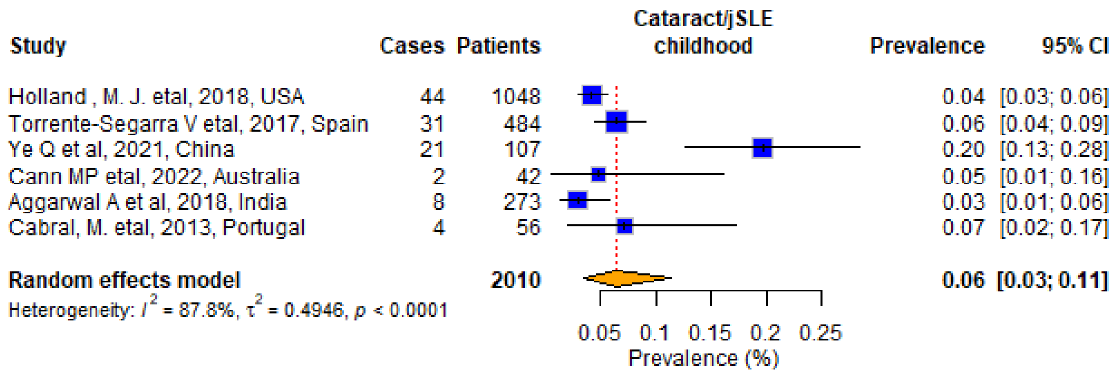

Funnel

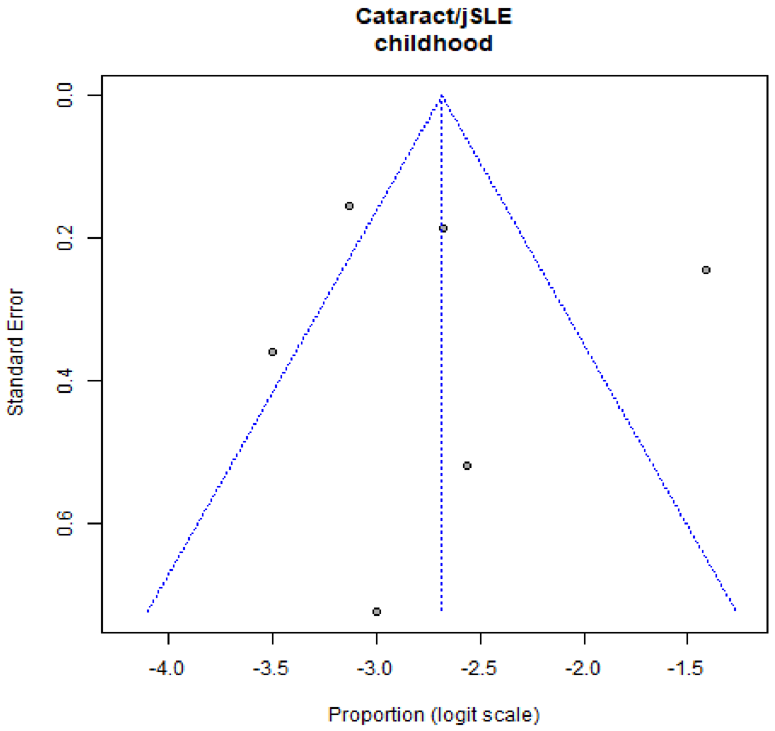

Summary statistics

```
|                                        proportion           95%-CI %W(random)
Holland , M. J. etal, 2018, USA              0.0420 [0.0307; 0.0560]       20.3
Torrente-Segarra V etal, 2017, Spain         0.0640 [0.0439; 0.0897]       19.9
Ye Q et al, 2021, China                      0.1963 [0.1258; 0.2842]       19.0
Cann MP etal, 2022, Australia                0.0476 [0.0058; 0.1616]       10.3
Aggarwal A et al, 2018, India                0.0293 [0.0127; 0.0569]       16.9
Cabral, M. etal, 2013, Portugal              0.0714 [0.0198; 0.1729]       13.8
Number of studies: k = 6; Number of observations: o = 2010; Number of events: e = 110
                     proportion           95%-CI
Random effects model     0.0639 [0.0349; 0.1141]
Quantifying heterogeneity (with 95%-CIs):  tau^2 = 0.4946 [0.1265; 3.0315]; tau = 0.7032 [0.3557; 1.7411]
 I^2 = 87.8% [75.9%; 93.8%]; H = 2.86 [2.04; 4.02]
Test of heterogeneity:      Q d.f.  p-value
                        40.98     5 < 0.0001
```

Supplementary Figure S42: Forest and Funnel Plots of studies reporting the prevalence of chronic kidney disease in children with juvenile-onset Systemic Lupus Erythematosus

Chronic kidney disease

Forest

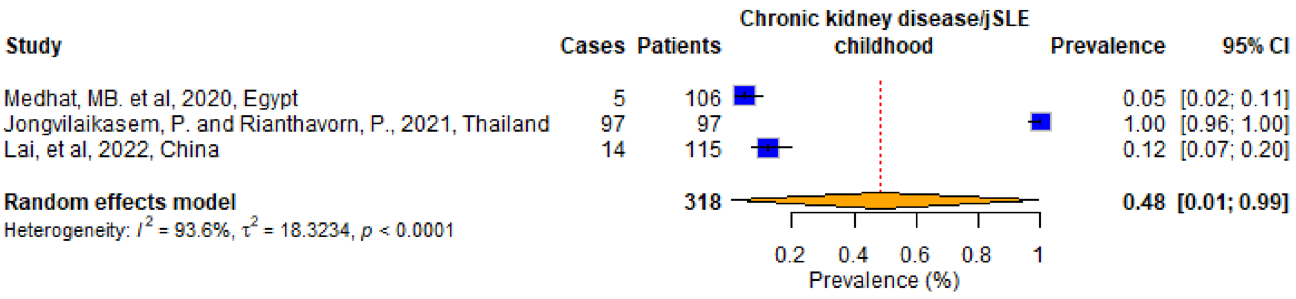

Funnel

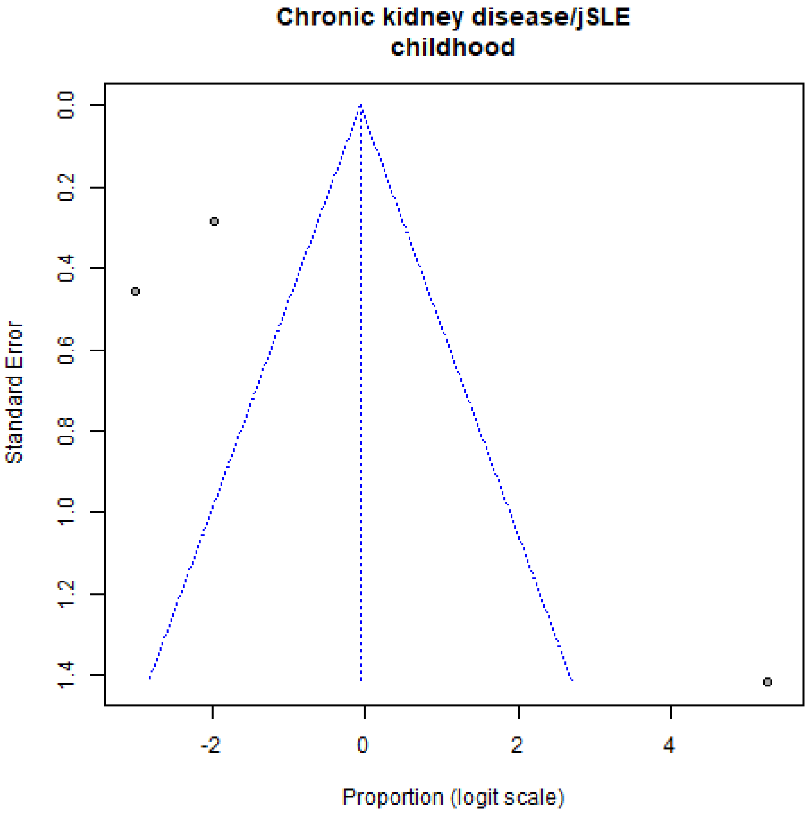

Summary statistics

```
                                                              proportion       95%-CI           %W(random)
Medhat, MB. et al, 2020, Egypt                                    0.0472       [0.0155; 0.1067]        34.3
Jongvilaikasem, P. and Rianthavorn, P., 2021, Thailand            1.0000       [0.9627; 1.0000]        31.2
Lai, et al, 2022, China                                           0.1217       [0.0682; 0.1958]        34.5
Number of studies: k = 3; Number of observations: o = 318; Number of events: e = 116
                     proportion            95%-CI
Random effects model      0.4838 [0.0067; 0.9924]
Quantifying heterogeneity (with 95%-CIs):  tau^2 = 18.3234 [4.1403; >183.2337]; tau = 4.2806 [2.0348; >13.5364]
 I^2 = 93.6% [84.6%; 97.3%]; H = 3.94 [2.55; 6.10]
Test of heterogeneity:       Q d.f.  p-value
                         31.09    2 < 0.0001
```

Supplementary Figure S43: Forest and Funnel Plots of studies reporting the prevalence of cerebrovascular accident in children with juvenile-onset Systemic Lupus Erythematosus

Cerebrovascular accident

Forest

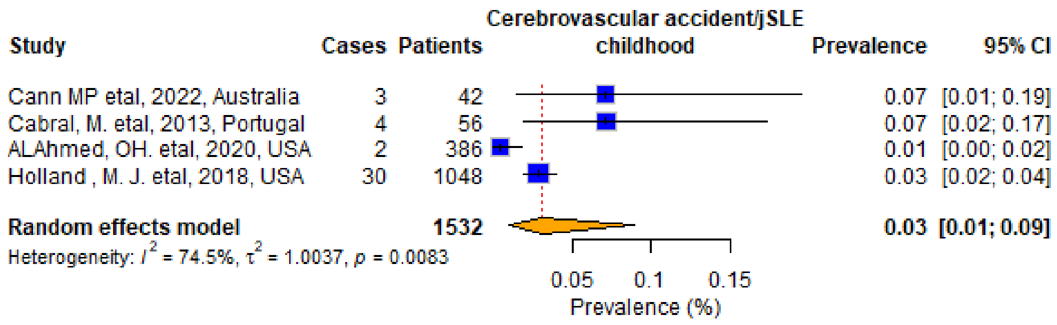

Funnel

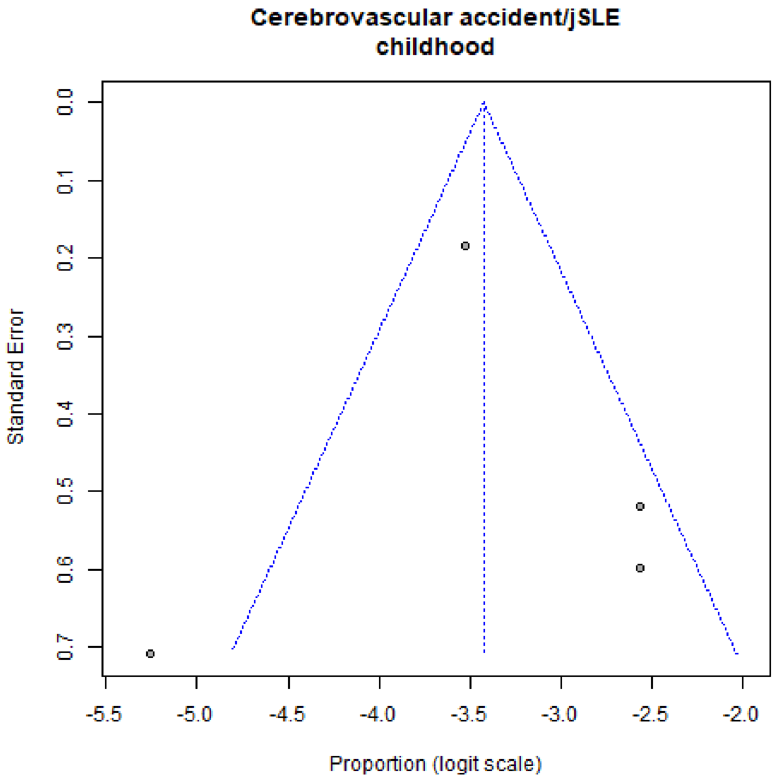

Summary statistics

```
                                 proportion      95%-CI           %W(random)
Cann MP etal, 2022, Australia        0.0714      [0.0150; 0.1948]        23.3
Cabral, M. etal, 2013, Portugal      0.0714      [0.0198; 0.1729]        25.0
ALAhmed, OH. etal, 2020, USA         0.0052      [0.0006; 0.0186]        21.1
Holland , M. J. etal, 2018, USA      0.0286      [0.0194; 0.0406]        30.6
Number of studies: k = 4; Number of observations: o = 1532; Number of events: e = 39
                      proportion           95%-CI
Random effects model      0.0315 [0.0106; 0.0893]
Quantifying heterogeneity (with 95%-CIs):  tau^2 = 1.0037 [0.0947; 21.9732]; tau = 1.0018 [0.3078; 4.6876]
 I^2 = 74.5% [28.9%; 90.8%]; H = 1.98 [1.19; 3.30]
Test of heterogeneity:        Q d.f. p-value
                         11.75    3  0.0083
```

Supplementary Figure S44: Forest and Funnel Plots of studies reporting the prevalence of depression in children with juvenile-onset Systemic Lupus Erythematosus

Depression

Forest

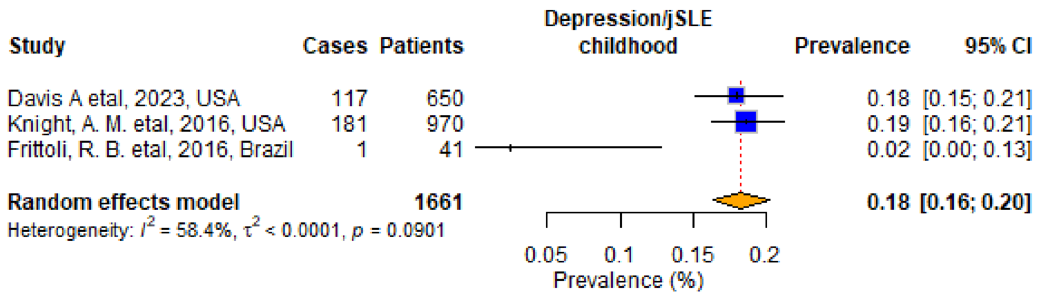

Funnel

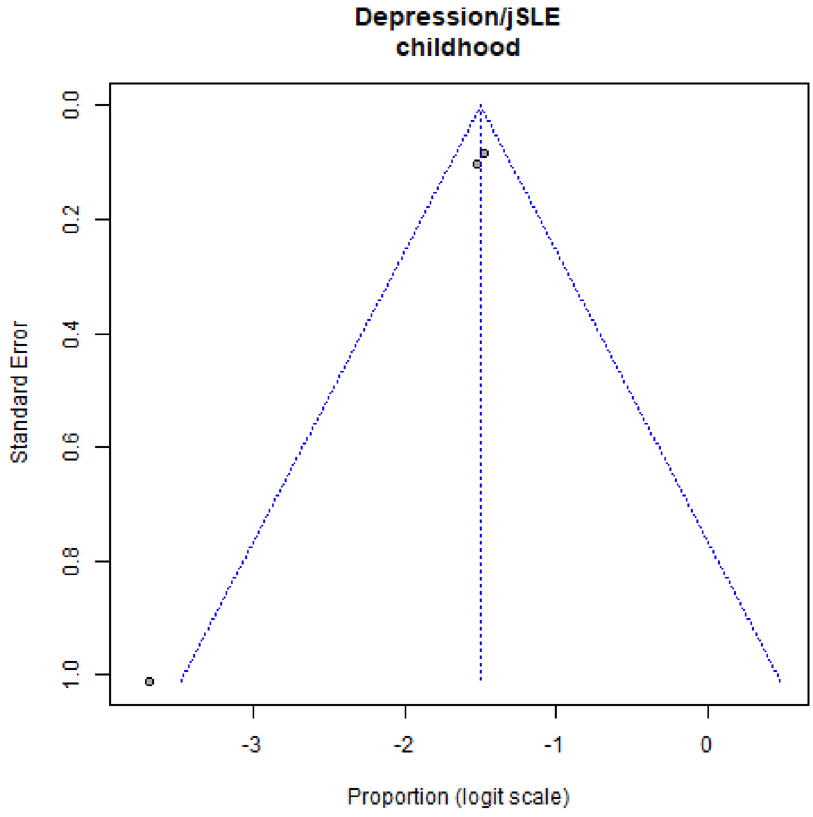

Summary statistics

```
                                    proportion    95%-CI          %W(random)
Davis A etal, 2023, USA                 0.1800   [0.1512; 0.2117]        39.3
Knight, A. M. etal, 2016, USA           0.1866   [0.1625; 0.2126]        60.3
Frittoli, R. B. etal, 2016, Brazil      0.0244   [0.0006; 0.1286]        0.4
Number of studies: k = 3; Number of observations: o = 1661; Number of events: e = 299
                      proportion           95%-CI
Random effects model      0.1827 [0.1647; 0.2021]
Quantifying heterogeneity (with 95%-CIs):  tau^2 < 0.0001 [0.0000; 62.7424]; tau = 0.0018 [0.0000; 7.9210]
 I^2 = 58.4% [0.0%; 88.2%]; H = 1.55 [1.00; 2.91]
Test of heterogeneity:     Q d.f. p-value
                        4.81    2  0.0901
```

Supplementary Figure S45: Forest and Funnel Plots of studies reporting the prevalence of diabetes (undefined) in children with juvenile-onset Systemic Lupus Erythematosus

Diabetes (undefined)

Forest

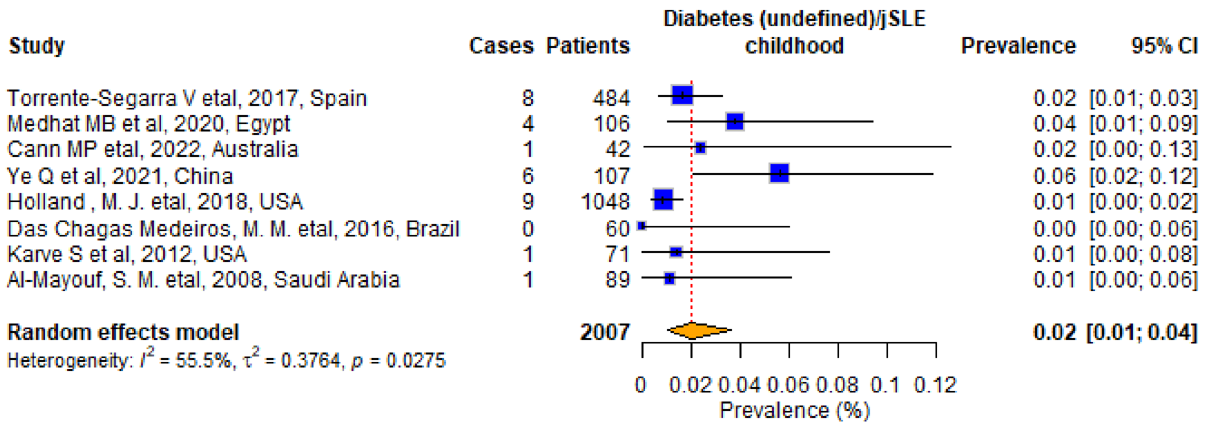

Funnel

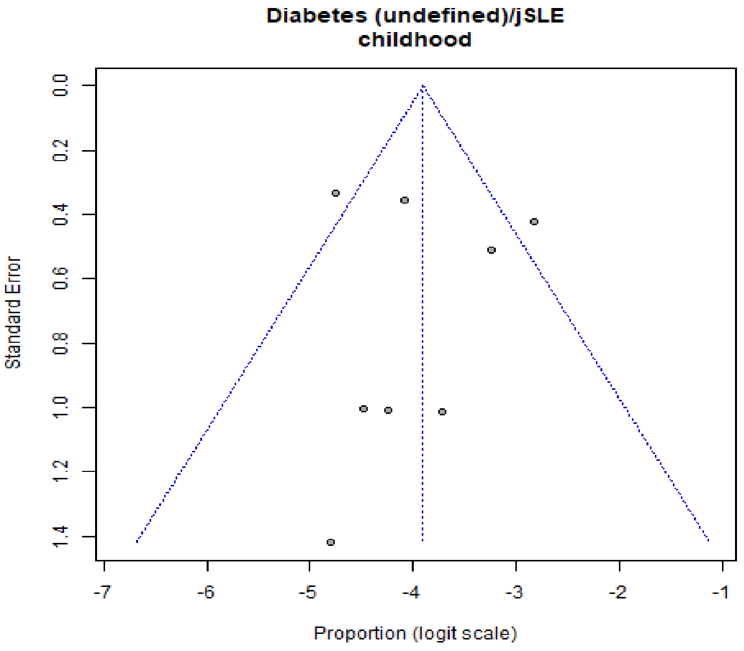

Summary statistics

```
                                              proportion          95%-CI           %W(random)
Torrente-Segarra V etal, 2017, Spain              0.0165     [0.0072; 0.0323]          19.9
Medhat MB et al, 2020, Egypt                      0.0377     [0.0104; 0.0938]          15.7
Cann MP etal, 2022, Australia                     0.0238     [0.0006; 0.1257]          7.1
Ye Q et al, 2021, China                           0.0561     [0.0209; 0.1181]          18.1
Holland , M. J. etal, 2018, USA                   0.0086     [0.0039; 0.0162]          20.5
Das Chagas Medeiros, M. M. etal, 2016, Brazil     0.0000     [0.0000; 0.0596]          4.2
Karve S et al, 2012, USA                          0.0141     [0.0004; 0.0760]          7.2
Al-Mayouf, S. M. etal, 2008, Saudi Arabia         0.0112     [0.0003; 0.0610]          7.2
Number of studies: k = 8; Number of observations: o = 2007; Number of events: e = 30
                     proportion           95%-CI
Random effects model     0.0198 [0.0107; 0.0362]
Quantifying heterogeneity (with 95%-CIs):  tau^2 = 0.3764 [0.0000; 1.6458]; tau = 0.6135 [0.0000; 1.2829]
 I^2 = 55.5% [1.9%; 79.9%]; H = 1.50 [1.01; 2.23]
Test of heterogeneity:        Q d.f. p-value
                          15.75    7  0.0275
```

Supplementary Figure S46: Forest and Funnel Plots of studies reporting the prevalence of end stage renal disease in children with juvenile-onset Systemic Lupus Erythematosus

End-stage renal disease

Forest

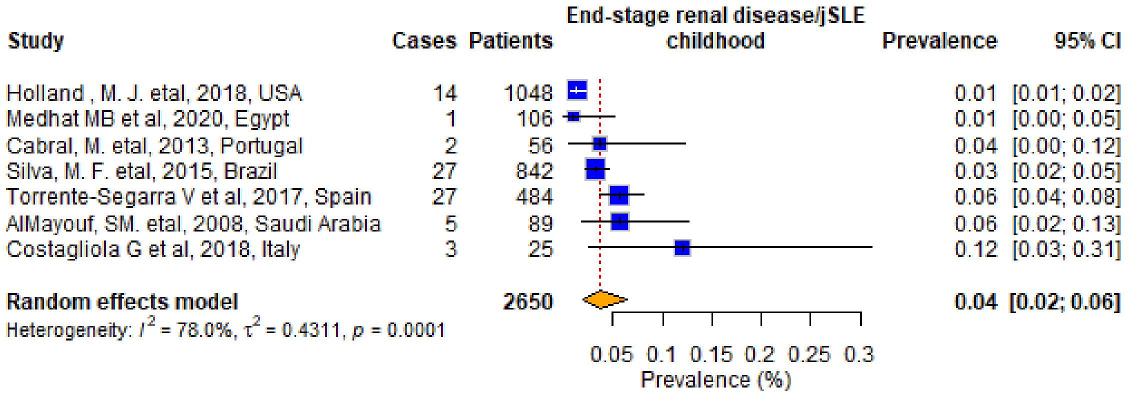

Funnel

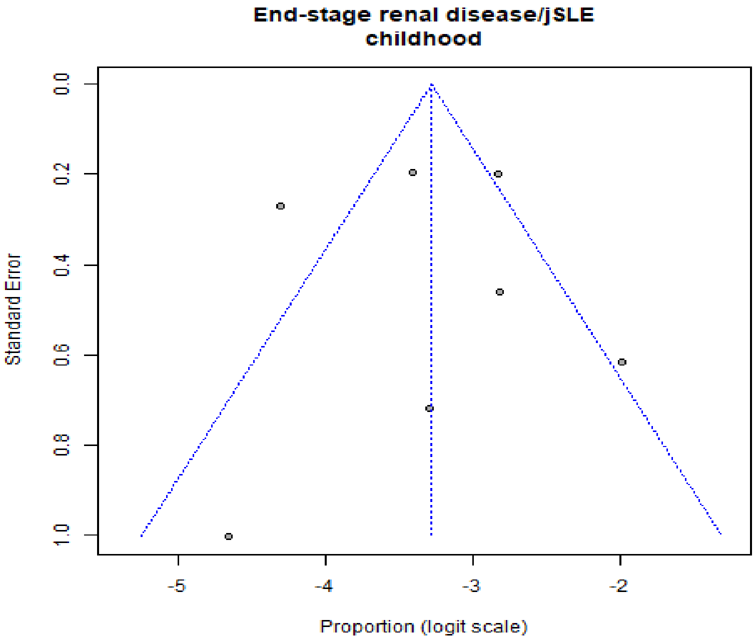

Summary statistics

```
                                         proportion       95%-CI          %W(random)
Holland , M. J. etal, 2018, USA              0.0134      [0.0073; 0.0223]       18.4
Medhat MB et al, 2020, Egypt                 0.0094      [0.0002; 0.0514]       6.4
Cabral, M. etal, 2013, Portugal              0.0357      [0.0044; 0.1231]       9.8
Silva, M. F. etal, 2015, Brazil              0.0321      [0.0212; 0.0463]       19.8
Torrente-Segarra V et al, 2017, Spain        0.0558      [0.0371; 0.0801]       19.7
AlMayouf, SM. etal, 2008, Saudi Arabia       0.0562      [0.0185; 0.1263]       14.4
Costagliola G et al, 2018, Italy             0.1200      [0.0255; 0.3122]       11.5
Number of studies: k = 7; Number of observations: o = 2650; Number of events: e = 79
                     proportion          95%-CI
Random effects model     0.0362 [0.0203; 0.0639]
Quantifying heterogeneity (with 95%-CIs):  tau^2 = 0.4311 [0.0842; 3.5598]; tau = 0.6566 [0.2901; 1.8867]
 I^2 = 78.0% [54.3%; 89.4%]; H = 2.13 [1.48; 3.07]
Test of heterogeneity:      Q d.f. p-value
                        27.24     6  0.0001
```

Supplementary Figure S47: Forest and Funnel Plots of studies reporting the prevalence of growth failure in children with juvenile-onset Systemic Lupus Erythematosus

Growth failure

Forest

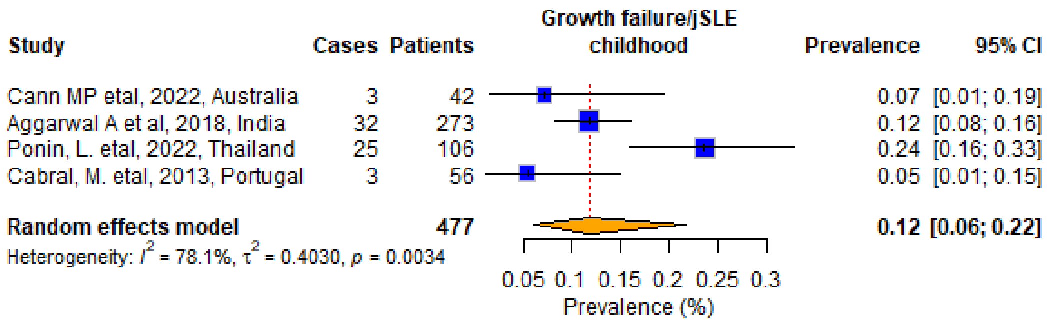

Funnel

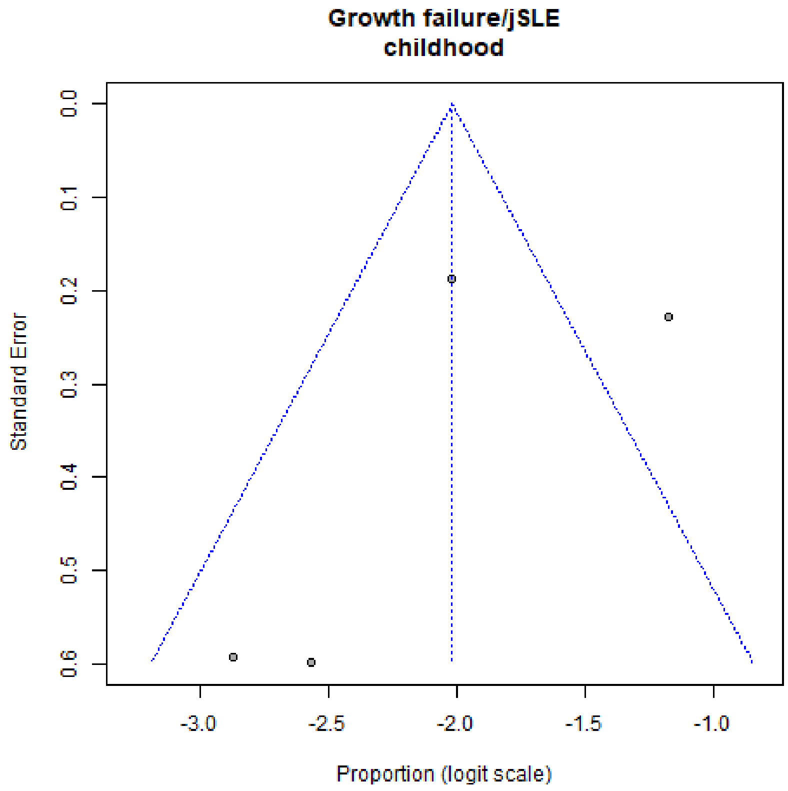

Summary statistics

```
                                 proportion      95%-CI           %W(random)
Cann MP etal, 2022, Australia        0.0714     [0.0150; 0.1948]        18.4
Aggarwal A et al, 2018, India        0.1172     [0.0816; 0.1614]        32.1
Ponin, L. etal, 2022, Thailand       0.2358     [0.1588; 0.3282]        30.9
Cabral, M. etal, 2013, Portugal      0.0536     [0.0112; 0.1487]        18.6
Number of studies: k = 4; Number of observations: o = 477; Number of events: e = 63
                      proportion           95%-CI
Random effects model      0.1173 [0.0599; 0.2170]
Quantifying heterogeneity (with 95%-CIs):  tau^2 = 0.4030 [0.0383; 7.5155]; tau = 0.6349 [0.1957; 2.7414]
 I^2 = 78.1% [40.8%; 91.9%]; H = 2.14 [1.30; 3.51]
Test of heterogeneity:      Q d.f. p-value
                        13.69    3  0.0034
```

Supplementary Figure S48: Forest and Funnel Plots of studies reporting the prevalence of obesity in children with juvenile-onset Systemic Lupus Erythematosus

Obesity

Forest

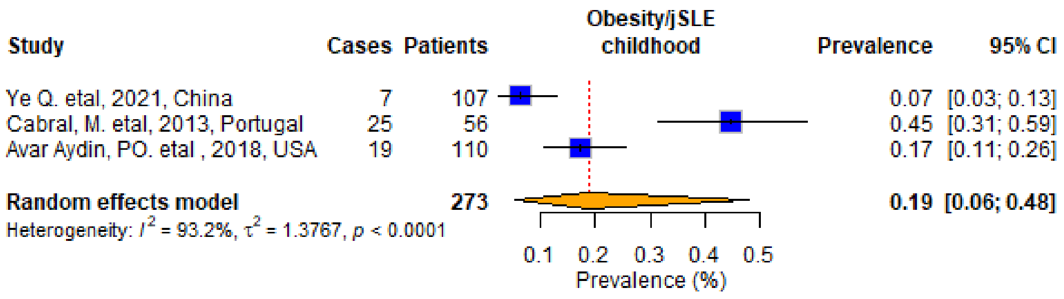

Funnel

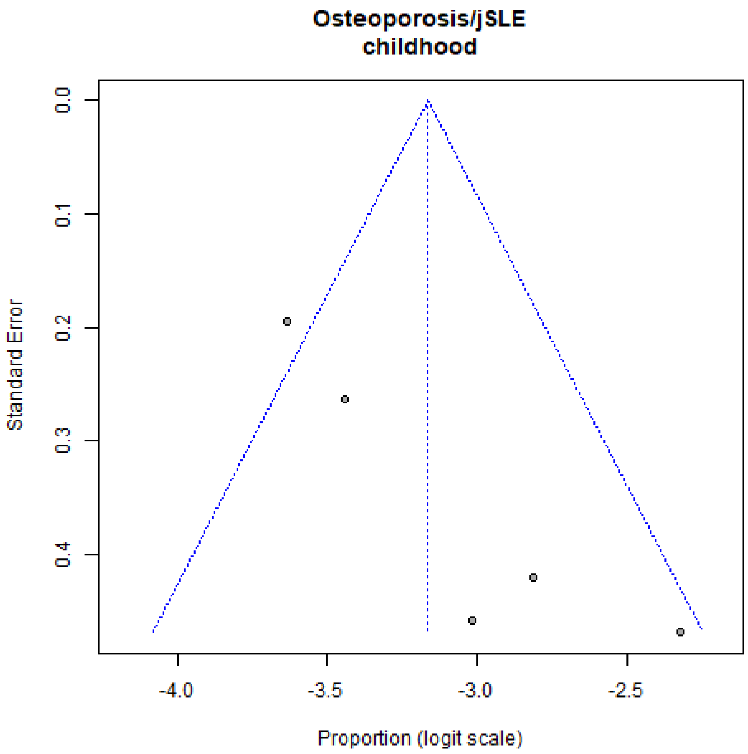

Summary statistics

```
                                 proportion     95%-CI           %W(random)
Ye Q. etal, 2021, China              0.0654     [0.0267; 0.1302]        32.1
Cabral, M. etal, 2013, Portugal      0.4464     [0.3134; 0.5853]        33.9
Avar Aydin, PO. etal , 2018, USA     0.1727     [0.1073; 0.2565]        34.1
Number of studies: k = 3; Number of observations: o = 273; Number of events: e = 51
                     proportion           95%-CI
Random effects model     0.1886 [0.0556; 0.4784]
Quantifying heterogeneity (with 95%-CIs):  tau^2 = 1.3767 [0.2984; 59.0990]; tau = 1.1733 [0.5463; 7.6876]
 I^2 = 93.2% [83.5%; 97.2%]; H = 3.83 [2.46; 5.97]
Test of heterogeneity:      Q d.f.  p-value
                        29.39    2 < 0.0001
```

Supplementary Figure S49: Forest and Funnel Plots of studies reporting the prevalence of hypertension in children with juvenile-onset Systemic Lupus Erythematosus

Hypertension

Forest

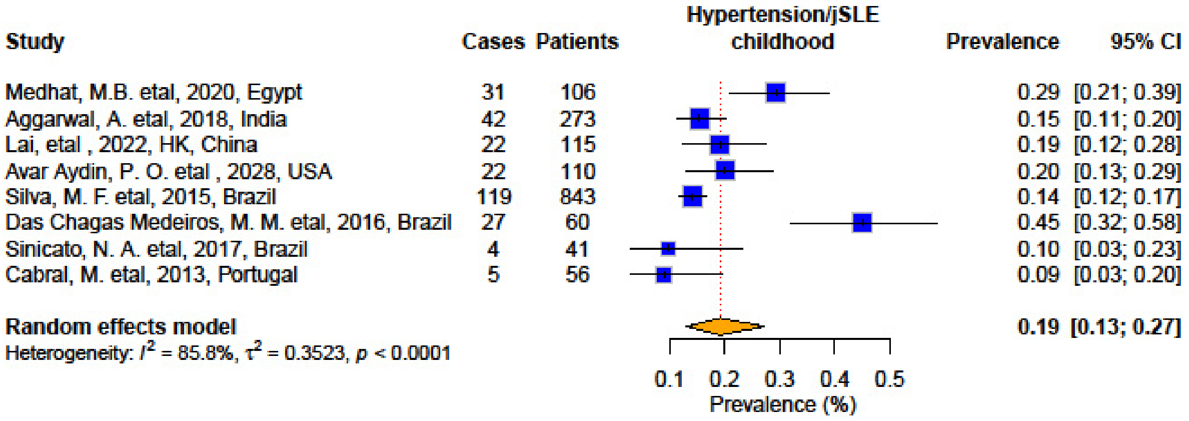

Funnel

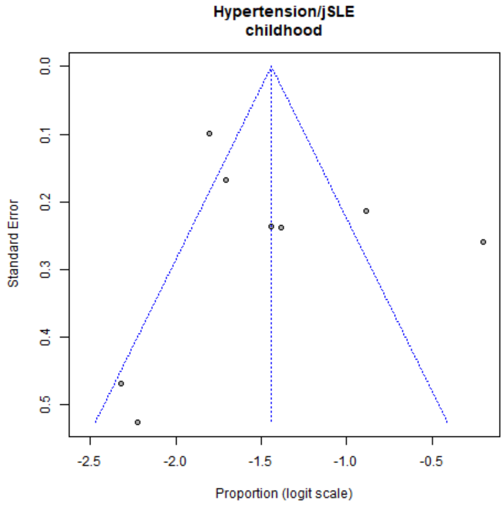

Summary statistics

```
                                               proportion           95% CI               %W(random)
Medhat, M.B. etal, 2020, Egypt                     0.2925    [0.2081; 0.3887]            13.6
Aggarwal, A. etal, 2018, India                     0.1538    [0.1132; 0.2022]            14.2
Lai, etal , 2022, HK, China                        0.1913    [0.1239; 0.2752]            13.2
Avar Aydin, P. O. etal , 2028, USA                 0.2000    [0.1298; 0.2870]            13.2
Silva, M. F. etal, 2015, Brazil                    0.1412    [0.1184; 0.1665]            14.9
Das Chagas Medeiros, M. M. etal, 2016, Brazil      0.4500    [0.3212; 0.5839]            12.9
Sinicato, N. A. etal, 2017, Brazil                 0.0976    [0.0272; 0.2313]             8.6
Cabral, M. etal, 2013, Portugal                    0.0893    [0.0296; 0.1962]             9.4

Number of studies: k = 8
Number of observations: o = 1604
Number of events: e = 272

                     proportion 95% CI
Random effects model     0.1914 [0.1305; 0.2718]

Quantifying heterogeneity (with 95% CIs):
 tau^2 = 0.3523 [0.1124; 1.9017]; tau = 0.5936 [0.3352; 1.3790]
 I^2 = 85.8% [74.0%; 92.3%]; H = 2.65 [1.96; 3.59]

Test of heterogeneity:
     Q d.f.  p-value
 49.34    7 < 0.0001
```

Supplementary Figure S50: Forest and Funnel Plots of studies reporting the prevalence of malignancy in children with juvenile-onset Systemic Lupus Erythematosus

Malignancy

Forest

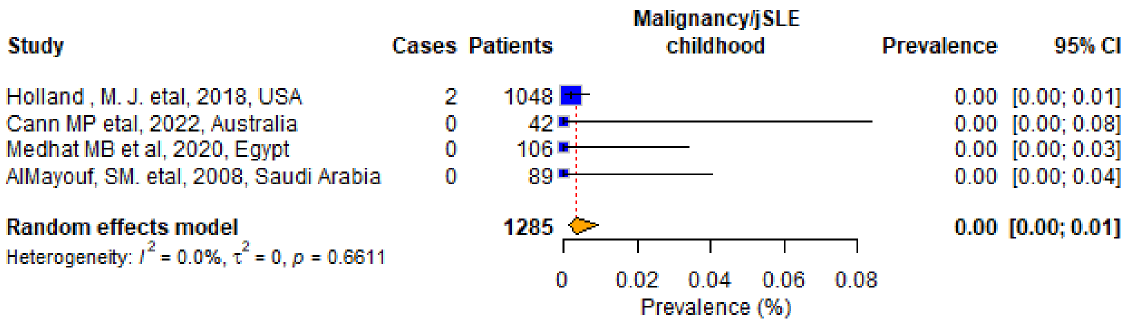

Funnel

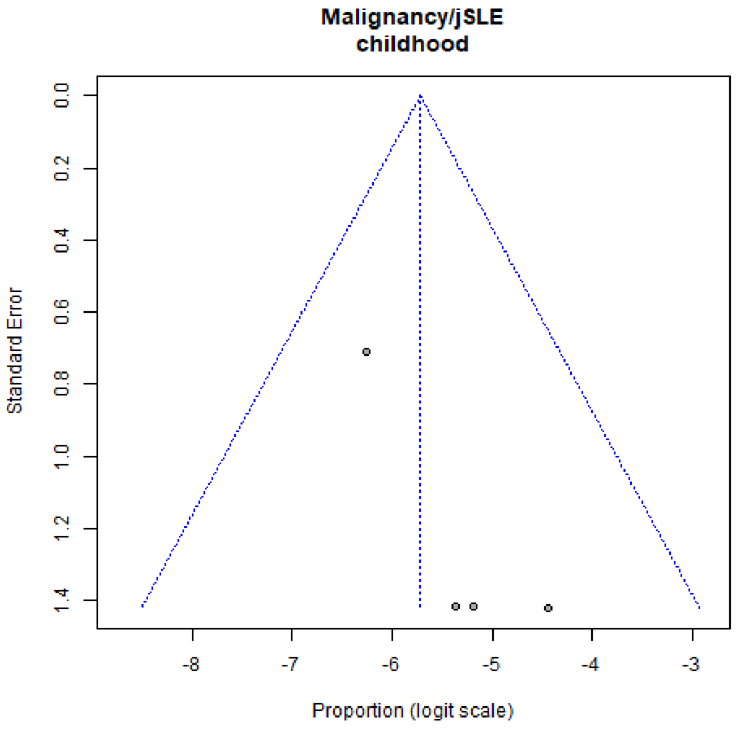

Summary statistics

```
                                          proportion        95%-CI           %W(random)
Holland , M. J. etal, 2018, USA               0.0019       [0.0002; 0.0069]         57.3
Cann MP etal, 2022, Australia                 0.0000       [0.0000; 0.0841]         14.2
Medhat MB et al, 2020, Egypt                  0.0000       [0.0000; 0.0342]         14.3
AlMayouf, SM. etal, 2008, Saudi Arabia        0.0000       [0.0000; 0.0406]         14.3
Number of studies: k = 4; Number of observations: o = 1285; Number of events: e = 2
                        proportion           95%-CI
Random effects model      0.0033 [0.0011; 0.0093]
Quantifying heterogeneity (with 95%-CIs):  tau^2 = 0 [0.0000; 6.5718]; tau = 0 [0.0000; 2.5635]
 I^2 = 0.0% [0.0%; 84.7%]; H = 1.00 [1.00; 2.56]
Test of heterogeneity:      Q d.f. p-value
                         1.59    3  0.6611
```

Supplementary Figure S51: Forest and Funnel Plots of studies reporting the prevalence of osteoporosis in children with juvenile-onset Systemic Lupus Erythematosus

Osteoporosis

Forest

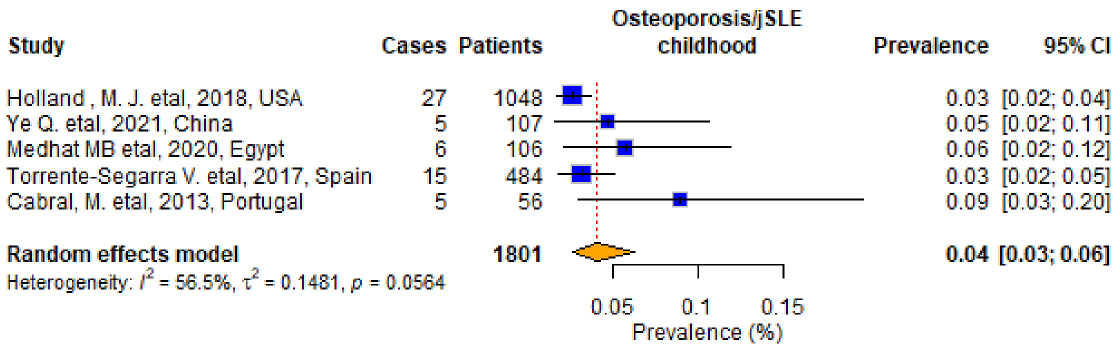

Funnel

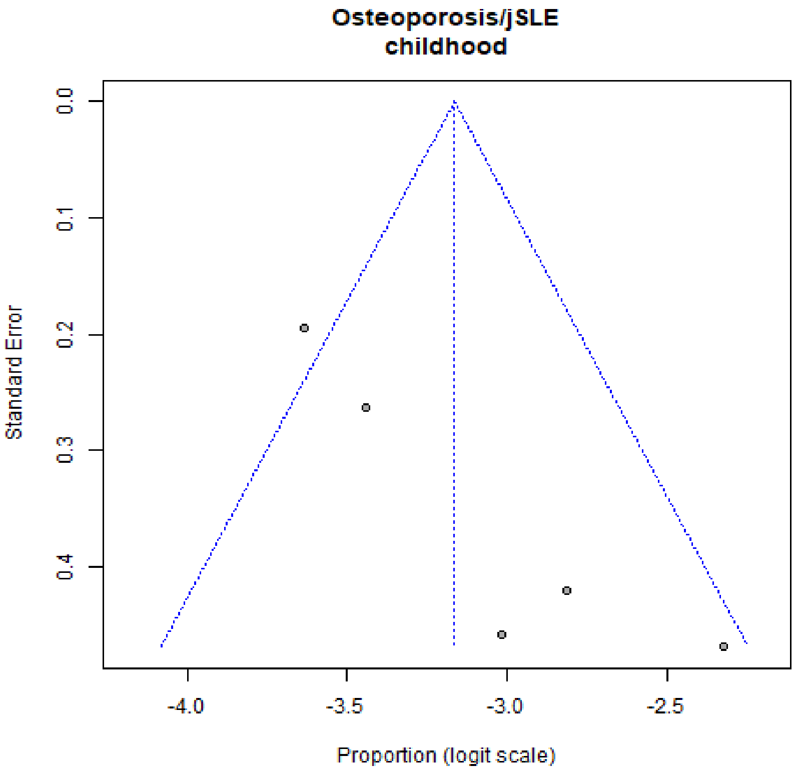

Summary statistics

```
                                         proportion        95%-CI          %W(random)
Holland , M. J. etal, 2018, USA              0.0258        [0.0170; 0.0373]       28.9
Ye Q. etal, 2021, China                      0.0467        [0.0153; 0.1057]       15.0
Medhat MB etal, 2020, Egypt                  0.0566        [0.0211; 0.1191]       16.6
Torrente-Segarra V. etal, 2017, Spain        0.0310        [0.0174; 0.0506]       24.8
Cabral, M. etal, 2013, Portugal              0.0893        [0.0296; 0.1962]       14.6
Number of studies: k = 5; Number of observations: o = 1801; Number of events: e = 58
                     proportion           95%-CI
Random effects model     0.0405 [0.0261; 0.0624]
Quantifying heterogeneity (with 95%-CIs):  tau^2 = 0.1481 [0.0000; 2.0934]; tau = 0.3848 [0.0000; 1.4469]
 I^2 = 56.5% [0.0%; 83.9%]; H = 1.52 [1.00; 2.49]
Test of heterogeneity:     Q d.f. p-value
                        9.19    4  0.0564
```

Supplementary Figure S52: Forest and Funnel Plots of studies reporting the prevalence of premature gonadal failure in children with juvenile-onset Systemic Lupus Erythematosus

Premature gonadal failure

Forest

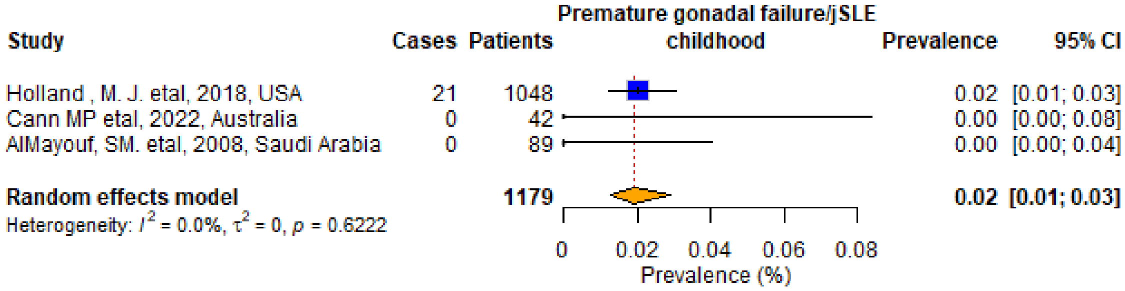

Funnel

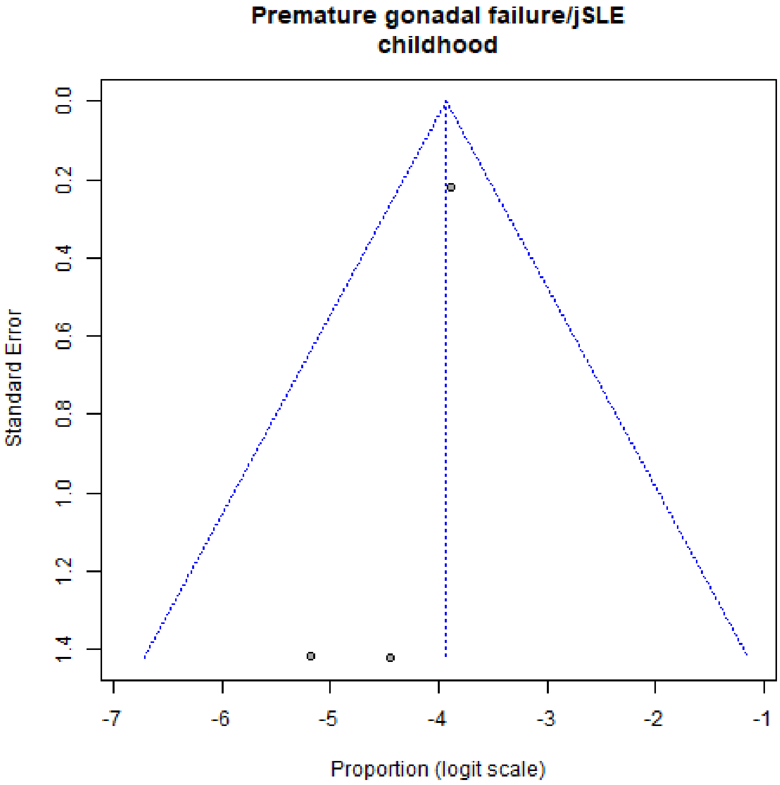

Summary statistics

```
                                             proportion       95%-CI          %W(random)
Holland , M. J. etal, 2018, USA                  0.0200       [0.0124; 0.0305]        95.4
Cann MP etal, 2022, Australia                    0.0000       [0.0000; 0.0841]         2.3
AlMayouf, SM. etal, 2008, Saudi Arabia           0.0000       [0.0000; 0.0406]         2.3
Number of studies: k = 3; Number of observations: o = 1179; Number of events: e = 21
                          proportion          95%-CI
Random effects model      0.0192 [0.0127; 0.0290]
Quantifying heterogeneity (with 95%-CIs):  tau^2 = 0 [0.0000; 15.6365]; tau = 0 [0.0000; 3.9543]
 I^2 = 0.0% [0.0%; 89.6%]; H = 1.00 [1.00; 3.10]
Test of heterogeneity:     Q d.f. p-value
                         0.95    2  0.6222
```

Supplementary Figure S53: Forest and Funnel Plots of studies reporting the prevalence of pulmonary hypertension in children with juvenile-onset Systemic Lupus Erythematosus

Pulmonary hypertension

Forest

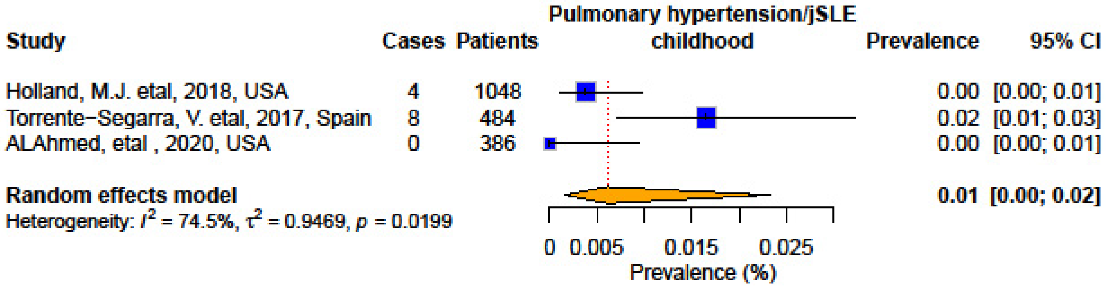

Funnel

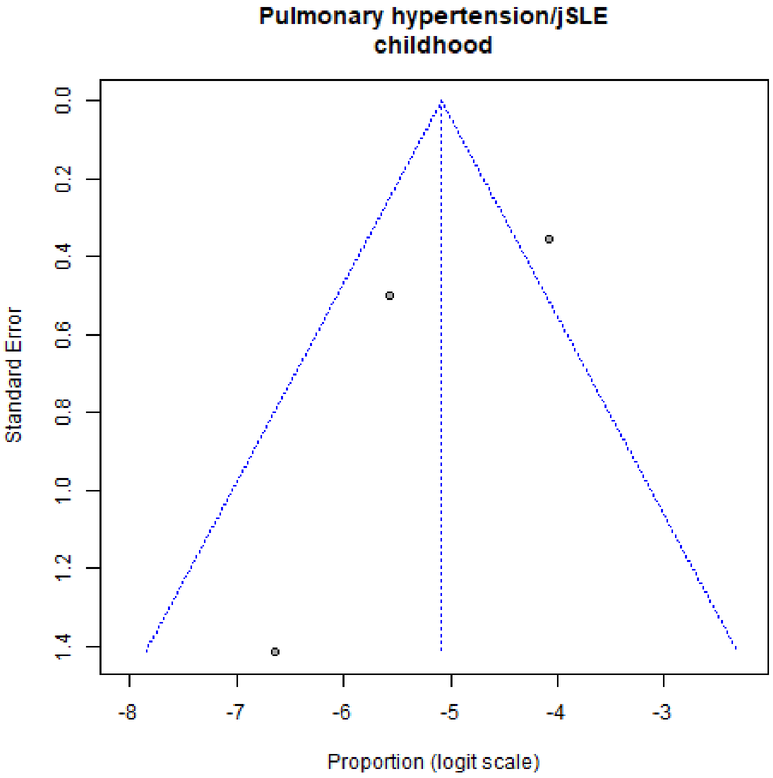

Summary statistics

```
                                          proportion          95%-CI      %W(random)
Holland, MJ. etal, 2018, USA                  0.0038 [0.0010; 0.0097]     39.7
Torrente-Segarra, V. etal, 2017, Spain        0.0165 [0.0072; 0.0323]     44.2
ALAhmed, etal , 2020, USA                     0.0000 [0.0000; 0.0095]     16.1

Number of studies: k = 3
Number of observations: o = 1918
Number of events: e = 12

                     proportion           95%-CI
Random effects model     0.0061 [0.0016; 0.0233]

Quantifying heterogeneity (with 95%-CIs):
 tau^2 = 0.9469 [0.0140; 64.4784]; tau = 0.9731 [0.1183; 8.0298]
 I^2 = 74.5% [15.0%; 92.3%]; H = 1.98 [1.08; 3.61]

Test of heterogeneity:
    Q d.f. p-value
 7.83    2  0.0199
```

Supplementary Figure S54: Forest and Funnel Plots of studies reporting the prevalence of transverse myelitis in children with juvenile-onset Systemic Lupus Erythematosus

Transverse myelitis

Forest

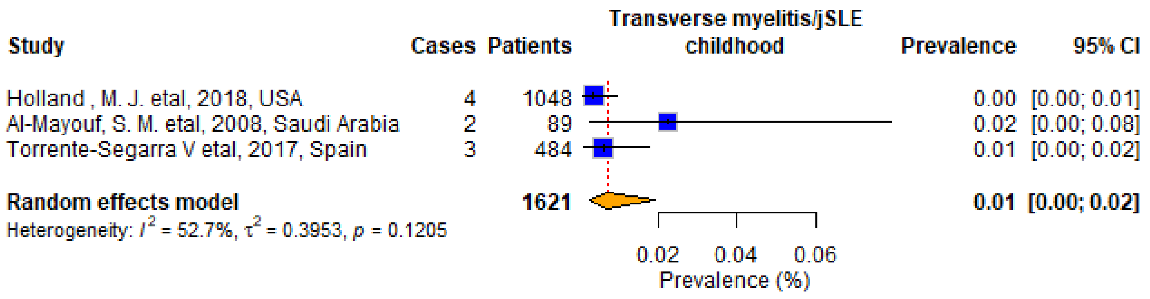

Funnel

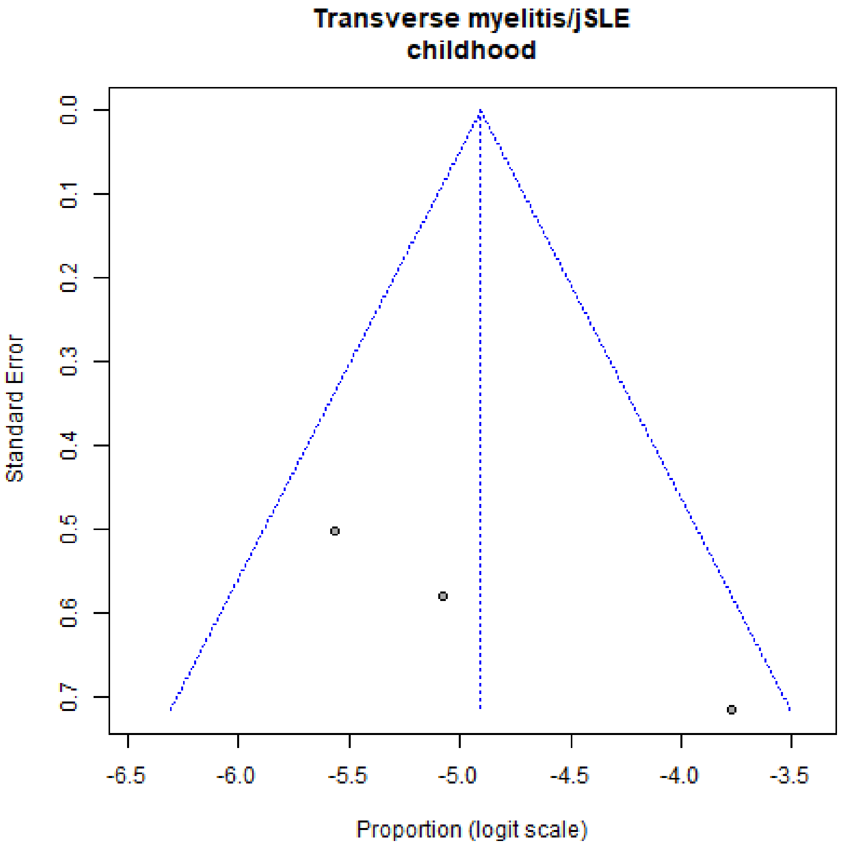

Summary statistics

```
                                          proportion     95%-CI           %W(random)
Holland , M. J. etal, 2018, USA               0.0038   [0.0010; 0.0097]        38.5
Al-Mayouf, S. M. etal, 2008, Saudi Arabia     0.0225   [0.0027; 0.0788]        27.4
Torrente-Segarra V etal, 2017, Spain          0.0062   [0.0013; 0.0180]        34.1
Number of studies: k = 3; Number of observations: o = 1621; Number of events: e = 9
                     proportion           95%-CI
Random effects model     0.0073 [0.0028; 0.0193]
Quantifying heterogeneity (with 95%-CIs):  tau^2 = 0.3953 [0.0000; 33.4831]; tau = 0.6287 [0.0000; 5.7865]
 I^2 = 52.7% [0.0%; 86.4%]; H = 1.45 [1.00; 2.71]
Test of heterogeneity:     Q d.f. p-value
                        4.23    2  0.1205
```

Supplementary Figure S55: Forest and Funnel Plots of studies reporting the prevalence of venous thrombosis in children with juvenile-onset Systemic Lupus Erythematosus

Venous thrombosis

Forest

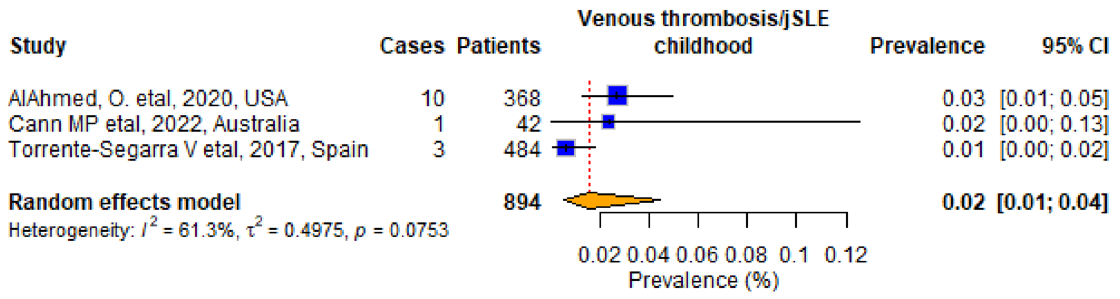

Funnel

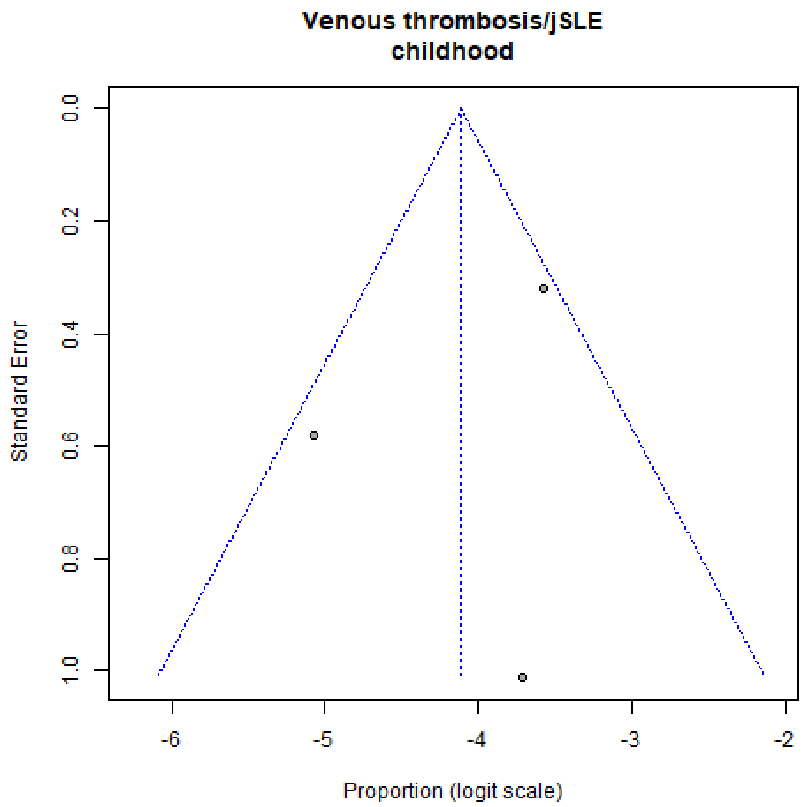

Summary statistics

```
                                      proportion         95%-CI  %W(random)
AlAhmed, O. etal, 2020, USA               0.0272 [0.0131; 0.0494]        47.3
Cann MP etal, 2022, Australia             0.0238 [0.0006; 0.1257]        18.6
Torrente-Segarra V etal, 2017, Spain      0.0062 [0.0013; 0.0180]        34.1
Number of studies: k = 3; Number of observations: o = 894; Number of events: e = 14
                     proportion           95%-CI
Random effects model     0.0161 [0.0057; 0.0444]
Quantifying heterogeneity (with 95%-CIs):  tau^2 = 0.4975 [0.0000; 26.7942]; tau = 0.7053 [0.0000; 5.1763]
 I^2 = 61.3% [0.0%; 89.0%]; H = 1.61 [1.00; 3.01]
Test of heterogeneity:     Q d.f. p-value
                         5.17    2  0.0753
```

jSLE/Adulthood

Supplementary Figure S56: Forest and Funnel Plots of studies reporting the prevalence of avascular necrosis in adults with juvenile-onset Systemic Lupus Erythematosus

Avascular necrosis

Forest

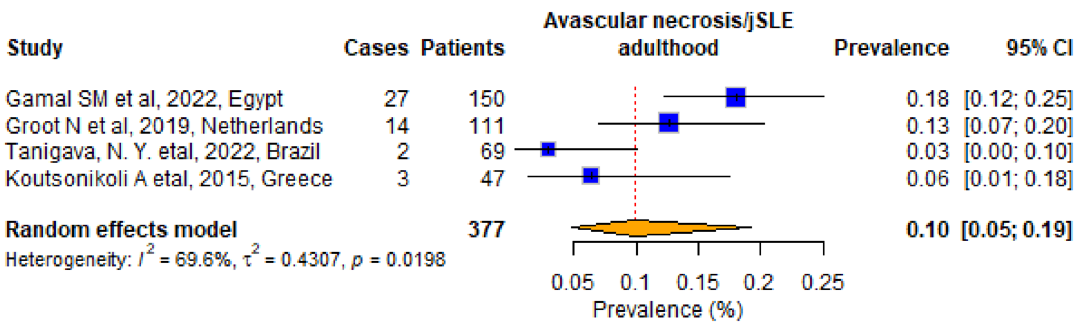

Funnel

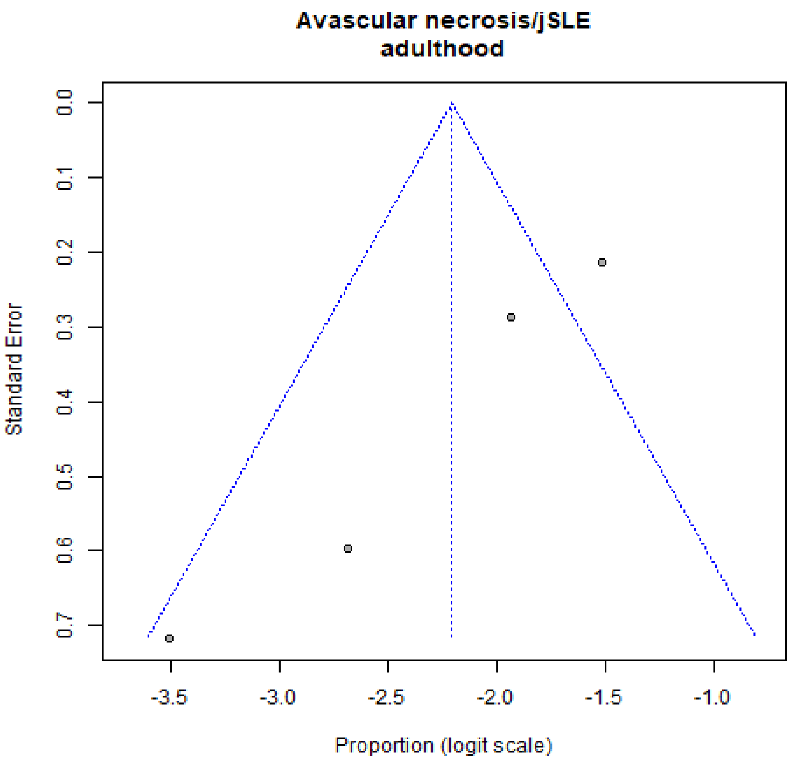

Summary statistics

```
                                   proportion   95%-CI            %W(random)
Gamal SM et al, 2022, Egypt            0.1800   [0.1221; 0.2510]        32.9
Groot N et al, 2019, Netherlands       0.1261   [0.0707; 0.2026]        30.6
Tanigava, N. Y. etal, 2022, Brazil     0.0290   [0.0035; 0.1008]        16.6
Koutsonikoli A etal, 2015, Greece      0.0638   [0.0134; 0.1754]        19.9
Number of studies: k = 4; Number of observations: o = 377; Number of events: e = 46
                        proportion           95%-CI
Random effects model      0.0990 [0.0482; 0.1928]
Quantifying heterogeneity (with 95%-CIs):  tau^2 = 0.4307 [0.0084; 10.4154]; tau = 0.6563 [0.0918; 3.2273]
 I^2 = 69.6% [12.4%; 89.4%]; H = 1.81 [1.07; 3.08]
Test of heterogeneity:     Q d.f. p-value
                        9.86    3  0.0198
```

Supplementary Figure S57: Forest and Funnel Plots of studies reporting the prevalence of cataract in adults with juvenile-onset Systemic Lupus Erythematosus

Cataract

Forest

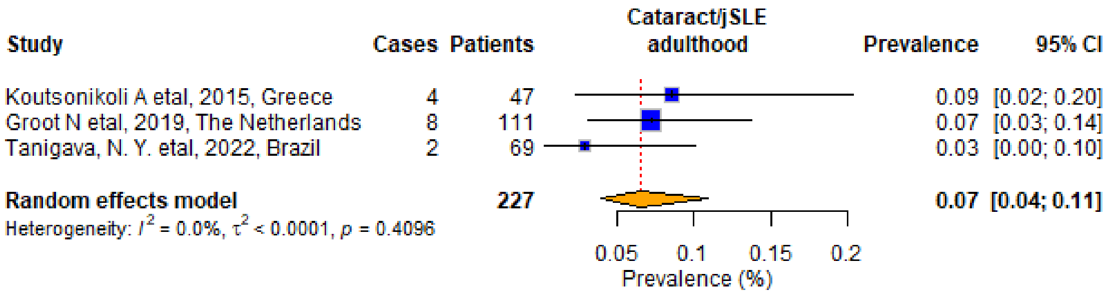

Funnel

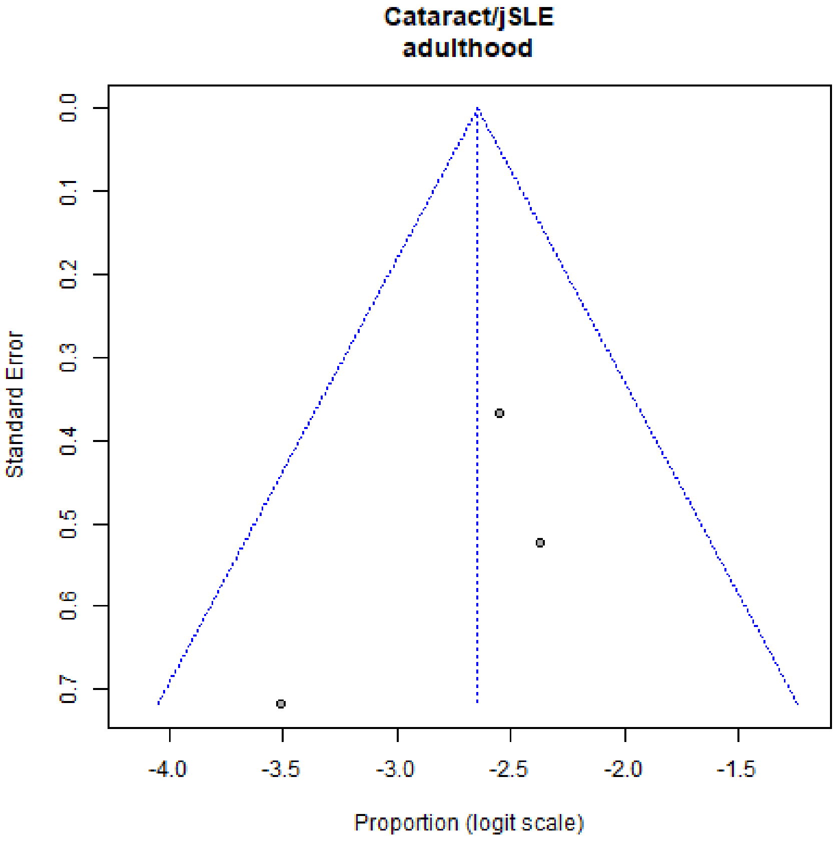

Summary statistics

```
                                    proportion  95%-CI           %W(random)
Koutsonikoli A etal, 2015, Greece       0.0851  [0.0237; 0.2038]        28.1
Groot N etal, 2019, The Netherlands     0.0721  [0.0316; 0.1371]        57.0
Tanigava, N. Y. etal, 2022, Brazil      0.0290  [0.0035; 0.1008]        14.9
Number of studies: k = 3; Number of observations: o = 227; Number of events: e = 14
                     proportion           95%-CI
Random effects model     0.0662 [0.0395; 0.1087]
Quantifying heterogeneity (with 95%-CIs):  tau^2 < 0.0001 [0.0000; 14.3203]; tau = 0.0004 [0.0000; 3.7842]
 I^2 = 0.0% [0.0%; 89.6%]; H = 1.00 [1.00; 3.10]
Test of heterogeneity:     Q d.f.  p-value
                         1.78    2  0.4096
```

Supplementary Figure S58: Forest and Funnel Plots of studies reporting the prevalence of diabetes (undefined) in adults with juvenile-onset Systemic Lupus Erythematosus

Diabetes (undefined)

Forest

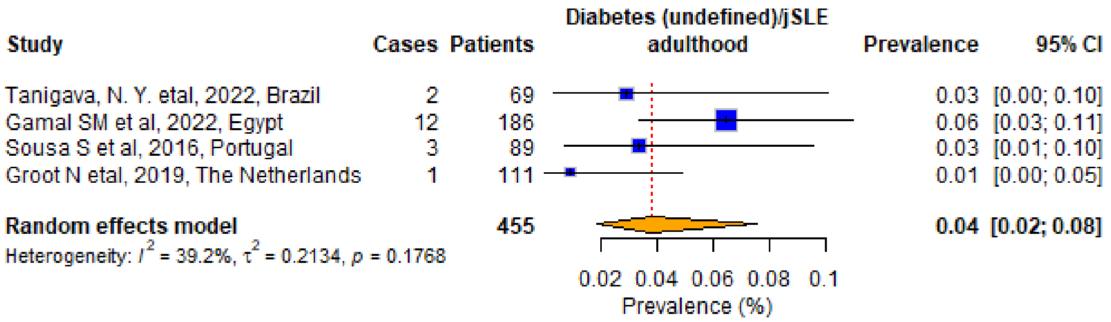

Funnel

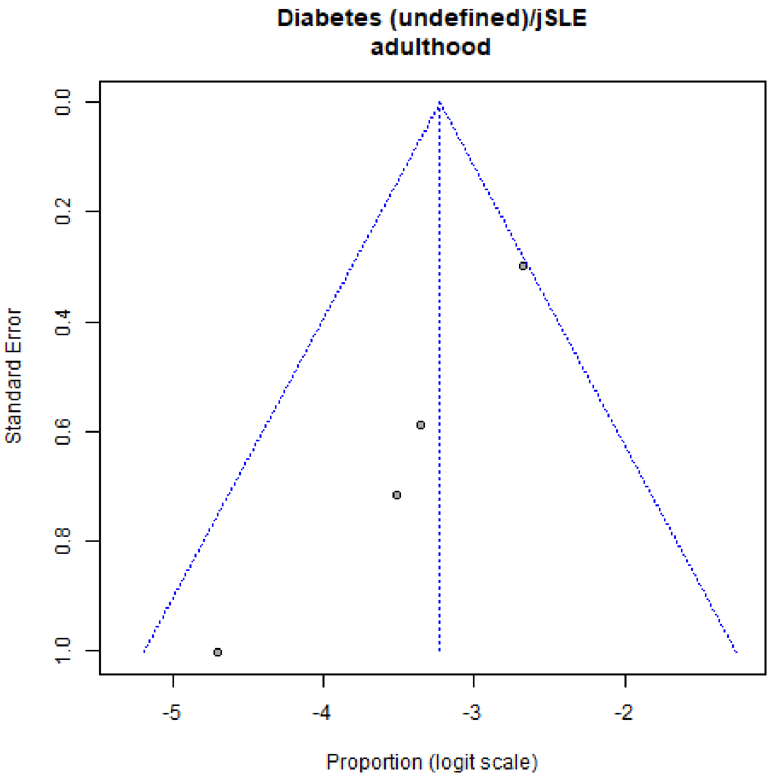

Summary statistics

```
                                    proportion  95%-CI           %W(random)
Tanigava, N. Y. etal, 2022, Brazil      0.0290  [0.0035; 0.1008]        18.8
Gamal SM et al, 2022, Egypt             0.0645  [0.0338; 0.1100]        45.4
Sousa S et al, 2016, Portugal           0.0337  [0.0070; 0.0954]        24.6
Groot N etal, 2019, The Netherlands     0.0090  [0.0002; 0.0492]        11.2
Number of studies: k = 4; Number of observations: o = 455; Number of events: e = 18
                     proportion          95%-CI
Random effects model     0.0382 [0.0188; 0.0758]
Quantifying heterogeneity (with 95%-CIs):  tau^2 = 0.2134 [0.0000; 9.2209]; tau = 0.4620 [0.0000; 3.0366]
 I^2 = 39.2% [0.0%; 79.3%]; H = 1.28 [1.00; 2.20]
Test of heterogeneity:      Q d.f.  p-value
                          4.93    3  0.1768
```

Supplementary Figure S59: Forest and Funnel Plots of studies reporting the prevalence of end-stage renal disease in adults with juvenile-onset Systemic Lupus Erythematosus

End-stage renal disease

Forest

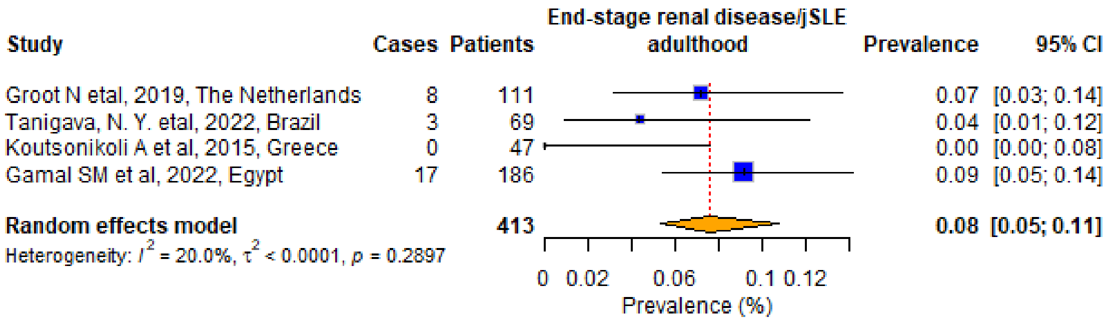

Funnel

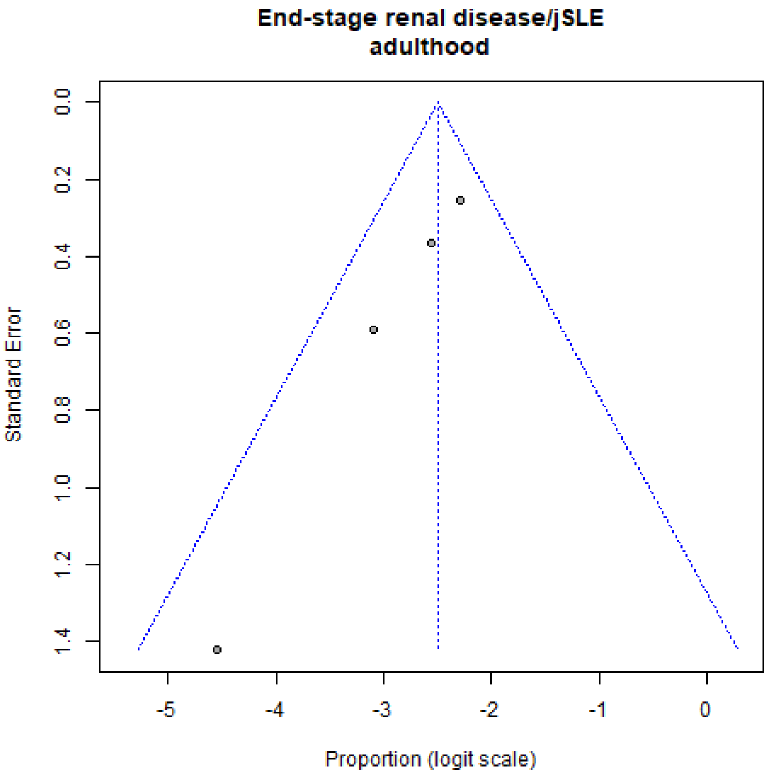

Summary statistics

```
                                    proportion  95%-CI          %W(random)
Groot N etal, 2019, The Netherlands     0.0721  [0.0316; 0.1371]        28.3
Tanigava, N. Y. etal, 2022, Brazil      0.0435  [0.0091; 0.1218]        10.9
Koutsonikoli A et al, 2015, Greece      0.0000  [0.0000; 0.0755]         1.9
Gamal SM et al, 2022, Egypt             0.0914  [0.0541; 0.1423]        58.9
Number of studies: k = 4; Number of observations: o = 413; Number of events: e = 28
                     proportion           95%-CI
Random effects model     0.0759 [0.0531; 0.1075]
Quantifying heterogeneity (with 95%-CIs):  tau^2 < 0.0001 [0.0000; 12.7842]; tau = 0.0006 [0.0000; 3.5755]
 I^2 = 20.0% [0.0%; 87.8%]; H = 1.12 [1.00; 2.86]
Test of heterogeneity:     Q d.f. p-value
                        3.75    3  0.2897
```

Supplementary Figure S60: Forest and Funnel Plots of studies reporting the prevalence of hypertension in adults with juvenile-onset Systemic Lupus Erythematosus

Hypertension

Forest

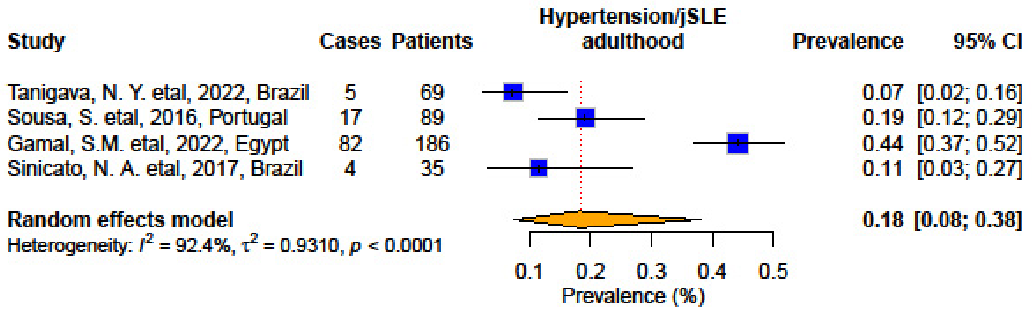

Funnel

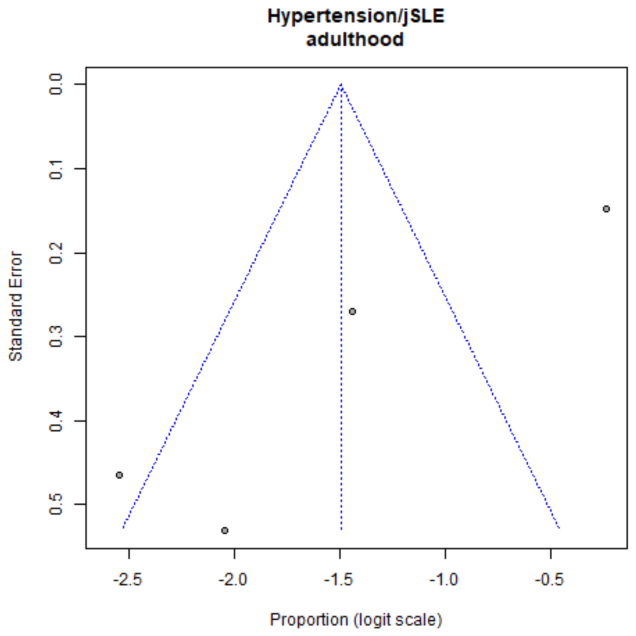

Summary statistics

```
                                   proportion    95% CI              %W(random)
Tanigava, N. Y. etal, 2022, Brazil     0.0725   [0.0239; 0.1611]         23.3
Sousa, S. etal, 2016, Portugal         0.1910   [0.1154; 0.2881]         26.6
Gamal, S.M. etal, 2022, Egypt          0.4409   [0.3683; 0.5154]         28.0
Sinicato, N. A. etal, 2017, Brazil     0.1143   [0.0320; 0.2674]         22.0

Number of studies: k = 4
Number of observations: o = 379
Number of events: e = 108

                      proportion 95% CI
Random effects model     0.1830 [0.0752; 0.3816]

Quantifying heterogeneity (with 95% CIs):
 tau^2 = 0.9310 [0.2222; 13.6986]; tau = 0.9649 [0.4714; 3.7012]
 I^2 = 92.4% [83.8%; 96.5%]; H = 3.63 [2.49; 5.31]

Test of heterogeneity:
     Q d.f.  p-value
 39.62    3 < 0.0001
```

Supplementary Figure S61: Forest and Funnel Plots of studies reporting the prevalence of malignancy in adults with juvenile-onset Systemic Lupus Erythematosus

Malignancy

Forest

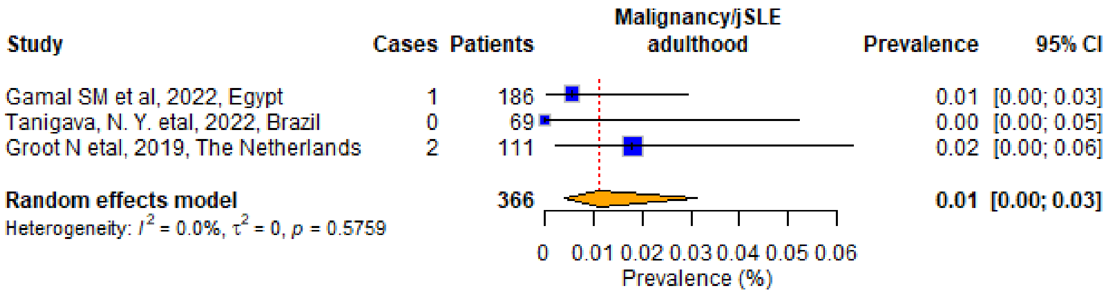

Funnel

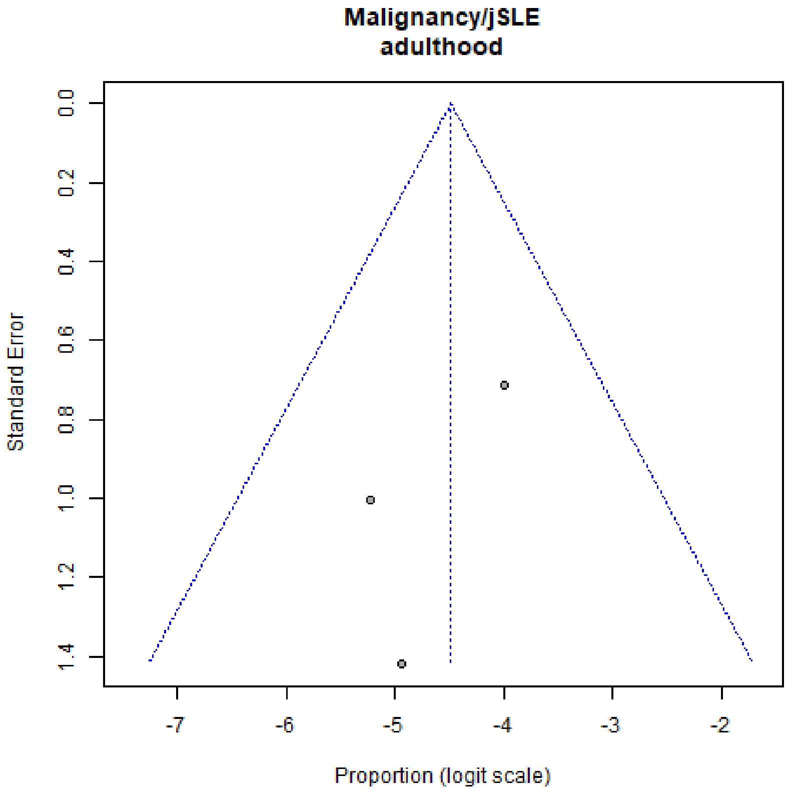

Summary statistics

```
                                    proportion  95%-CI          %W(random)
Gamal SM et al, 2022, Egypt             0.0054  [0.0001; 0.0296]        28.8
Tanigava, N. Y. etal, 2022, Brazil      0.0000  [0.0000; 0.0521]        14.4
Groot N etal, 2019, The Netherlands     0.0180  [0.0022; 0.0636]        56.8
Number of studies: k = 3; Number of observations: o = 366; Number of events: e = 3
                     proportion           95%-CI
Random effects model     0.0112 [0.0039; 0.0314]
Quantifying heterogeneity (with 95%-CIs):  tau^2 = 0 [0.0000; 15.3931]; tau = 0 [0.0000; 3.9234]
 I^2 = 0.0% [0.0%; 89.6%]; H = 1.00 [1.00; 3.10]
Test of heterogeneity:     Q d.f. p-value
                        1.10    2  0.5759
```

Supplementary Figure S62: Forest and Funnel Plots of studies reporting the prevalence of premature gonadal failure in adults with juvenile-onset Systemic Lupus Erythematosus

Premature gonadal failure

Forest

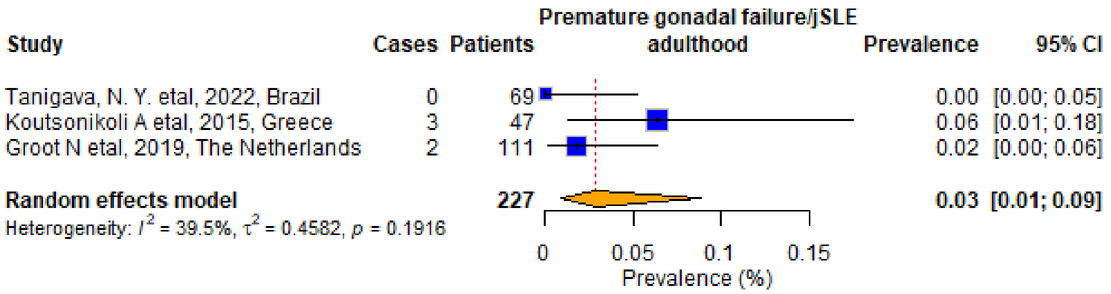

Funnel

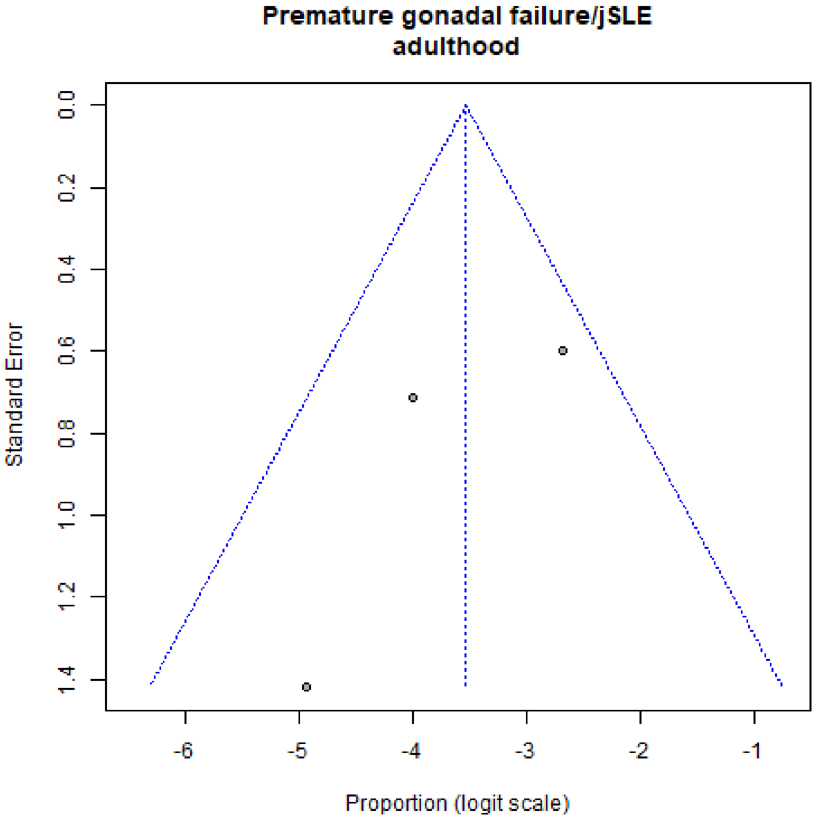

Summary statistics

```
                                    proportion  95%-CI           %W(random)
Tanigava, N. Y. etal, 2022, Brazil      0.0000  [0.0000; 0.0521]        15.2
Koutsonikoli A etal, 2015, Greece       0.0638  [0.0134; 0.1754]        46.1
Groot N etal, 2019, The Netherlands     0.0180  [0.0022; 0.0636]        38.8
Number of studies: k = 3; Number of observations: o = 227; Number of events: e = 5
                     proportion           95%-CI
Random effects model     0.0283 [0.0087; 0.0882]
Quantifying heterogeneity (with 95%-CIs):  tau^2 = 0.4582 [0.0000; 49.3226]; tau = 0.6769 [0.0000; 7.0230]
 I^2 = 39.5% [0.0%; 81.3%]; H = 1.29 [1.00; 2.31]
Test of heterogeneity:      Q d.f. p-value
                         3.30    2  0.1916
```

Supplementary Figure S63: Forest and Funnel Plots of studies reporting the prevalence of pulmonary fibrosis in adults with juvenile-onset Systemic Lupus Erythematosus

Pulmonary fibrosis

Forest

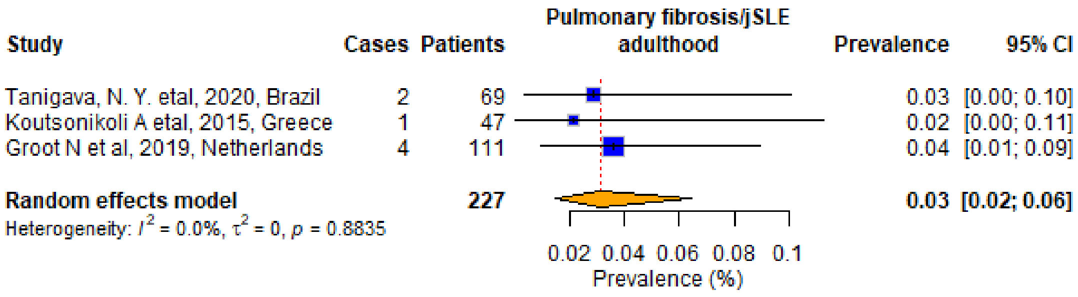

Funnel

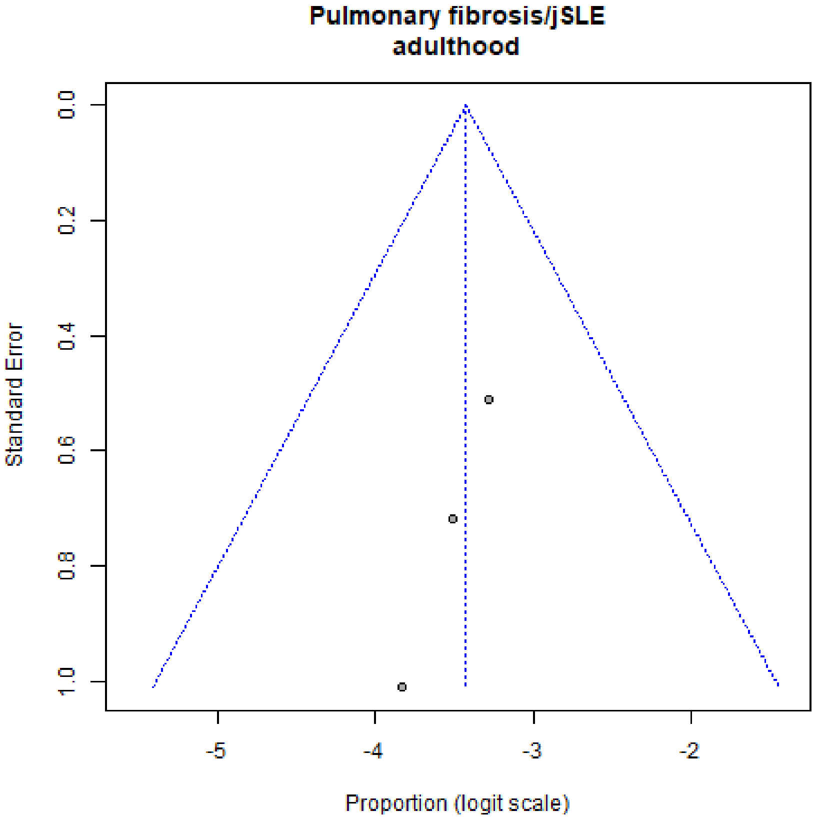

Summary statistics

```
                                     proportion    95%-CI           %W(random)
Tanigava, N. Y. etal, 2020, Brazil       0.0290    [0.0035; 0.1008]        28.7
Koutsonikoli A etal, 2015, Greece        0.0213    [0.0005; 0.1129]        14.4
Groot N et al, 2019, Netherlands         0.0360    [0.0099; 0.0897]        56.9
Number of studies: k = 3; Number of observations: o = 227; Number of events: e = 7
                         proportion           95%-CI
Random effects model      0.0314 [0.0150; 0.0644]
Quantifying heterogeneity (with 95%-CIs):  tau^2 = 0 [0.0000; 2.2629]; tau = 0 [0.0000; 1.5043]
 I^2 = 0.0% [0.0%; 89.6%]; H = 1.00 [1.00; 3.10]
Test of heterogeneity:     Q d.f. p-value
                         0.25     2  0.8835
```

Supplementary Figure S64: Forest and Funnel Plots of studies reporting the prevalence of pulmonary infarction in adults with juvenile-onset Systemic Lupus Erythematosus

Pulmonary infarction

Forest

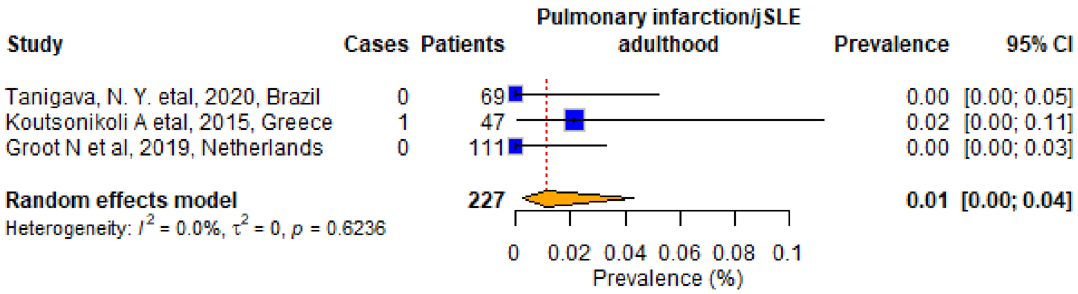

Funnel

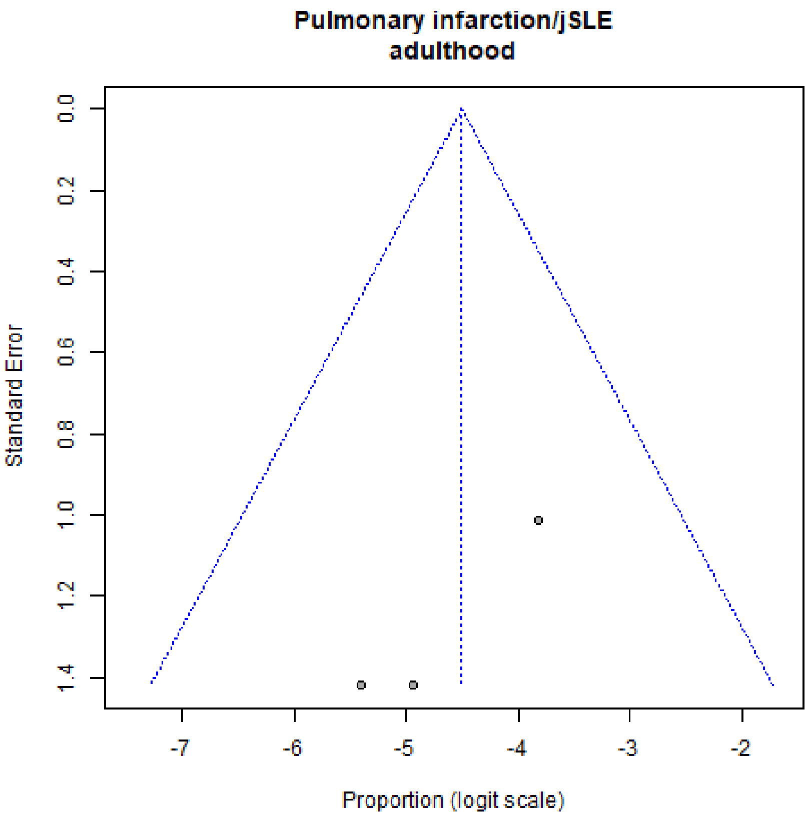

Summary statistics

```
                                        proportion    95%-CI           %W(random)
Tanigava, N. Y. etal, 2020, Brazil          0.0000    [0.0000; 0.0521]         25.2
Koutsonikoli A etal, 2015, Greece           0.0213    [0.0005; 0.1129]         49.6
Groot N et al, 2019, Netherlands            0.0000    [0.0000; 0.0327]         25.2
Number of studies: k = 3; Number of observations: o = 227; Number of events: e = 1
                          proportion           95%-CI
Random effects model        0.0109 [0.0027; 0.0427]
Quantifying heterogeneity (with 95%-CIs):  tau^2 = 0 [0.0000; 24.5185]; tau = 0 [0.0000; 4.9516]
 I^2 = 0.0% [0.0%; 89.6%]; H = 1.00 [1.00; 3.10]
Test of heterogeneity:      Q d.f. p-value
                         0.94     2  0.6236
```

Supplementary Figure S65: Forest and Funnel Plots of studies reporting the prevalence of seizures in adults with juvenile-onset Systemic Lupus Erythematosus

Seizures

Forest

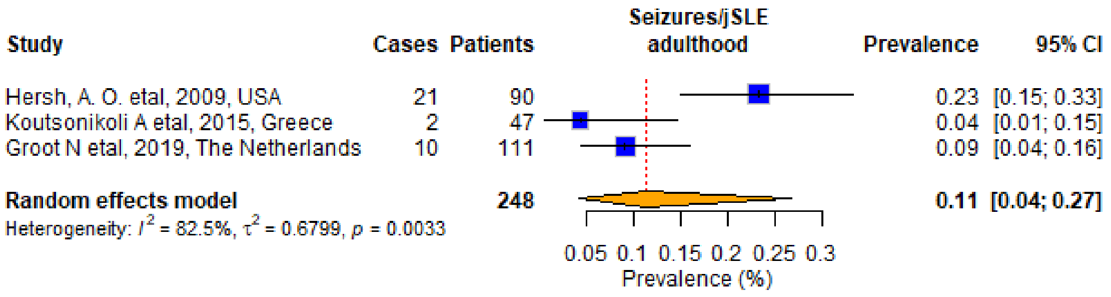

Funnel

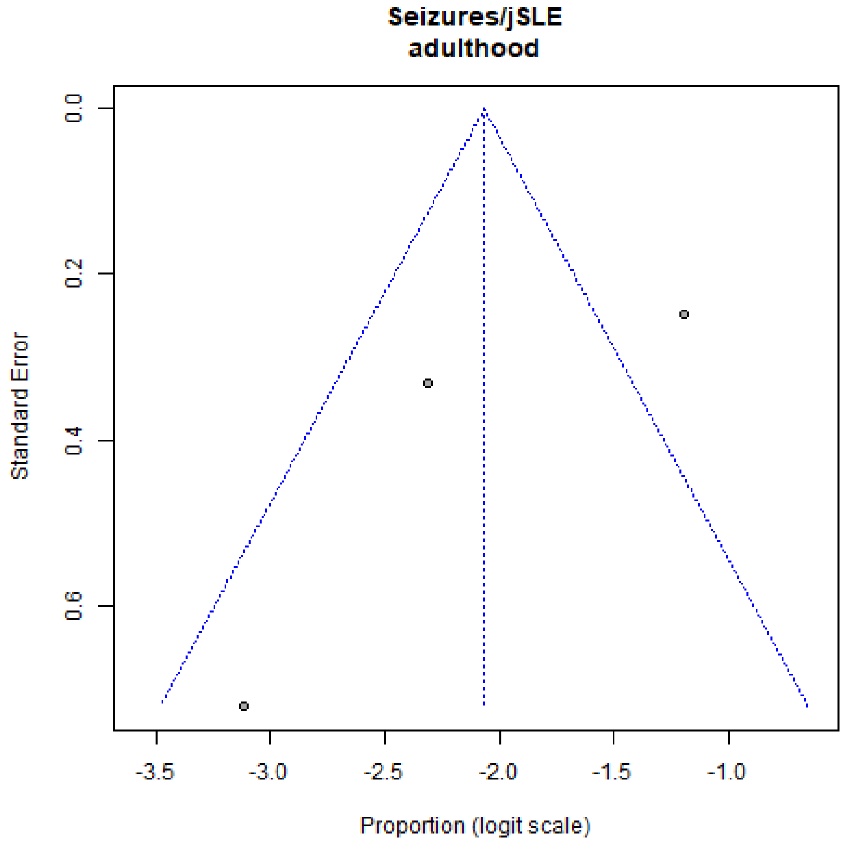

Summary statistics

```
                                    proportion  95%-CI           %W(random)
Hersh, A. O. etal, 2009, USA            0.2333  [0.1506; 0.3343]        39.1
Koutsonikoli A etal, 2015, Greece       0.0426  [0.0052; 0.1454]        24.1
Groot N etal, 2019, The Netherlands     0.0901  [0.0441; 0.1594]        36.7
Number of studies: k = 3; Number of observations: o = 248; Number of events: e = 33
                       proportion           95%-CI
Random effects model     0.1124 [0.0422; 0.2668]
Quantifying heterogeneity (with 95%-CIs):  tau^2 = 0.6799 [0.0646; 36.6266]; tau = 0.8246 [0.2542; 6.0520]
 I^2 = 82.5% [46.4%; 94.3%]; H = 2.39 [1.37; 4.19]
Test of heterogeneity:       Q d.f. p-value
                         11.43    2  0.0033
```

Supplementary Figure S66: Forest and Funnel Plots of studies reporting the prevalence of transverse myelitis in adults with juvenile-onset Systemic Lupus Erythematosus

Transverse myelitis

Forest

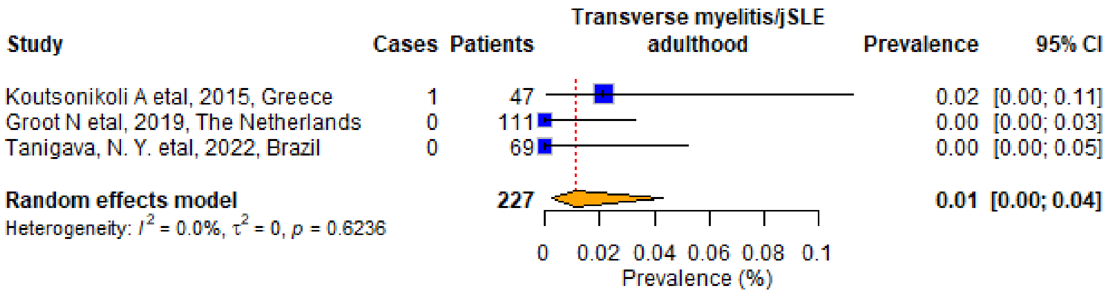

Funnel

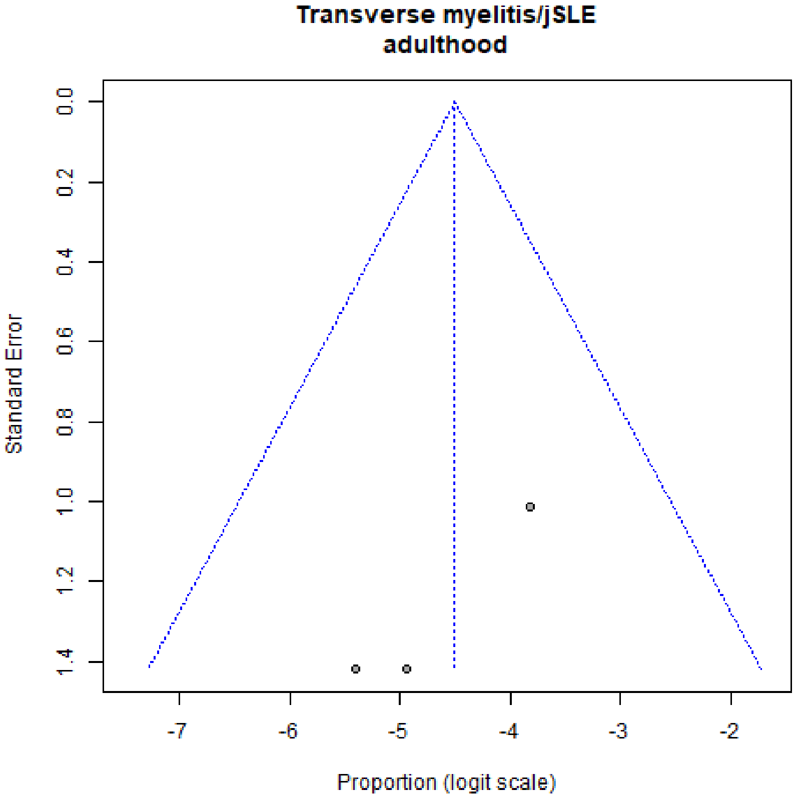

Summary statistics

```
                                    proportion  95%-CI           %W(random)
Koutsonikoli A etal, 2015, Greece       0.0213  [0.0005; 0.1129]        49.6
Groot N etal, 2019, The Netherlands     0.0000  [0.0000; 0.0327]        25.2
Tanigava, N. Y. etal, 2022, Brazil      0.0000  [0.0000; 0.0521]        25.2
Number of studies: k = 3; Number of observations: o = 227; Number of events: e = 1
                     proportion           95%-CI
Random effects model     0.0109 [0.0027; 0.0427]
Quantifying heterogeneity (with 95%-CIs):  tau^2 = 0 [0.0000; 24.5185]; tau = 0 [0.0000; 4.9516]
 I^2 = 0.0% [0.0%; 89.6%]; H = 1.00 [1.00; 3.10]
Test of heterogeneity:      Q d.f. p-value
                        0.94    2  0.6236
```

Supplementary Figure S67: Forest and Funnel Plots of studies reporting the prevalence of venous thrombosis in adults with juvenile-onset Systemic Lupus Erythematosus

Venous thrombosis

Forest

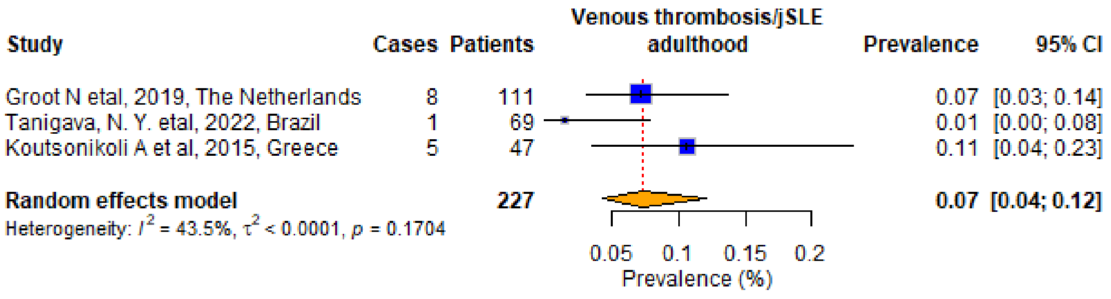

Funnel

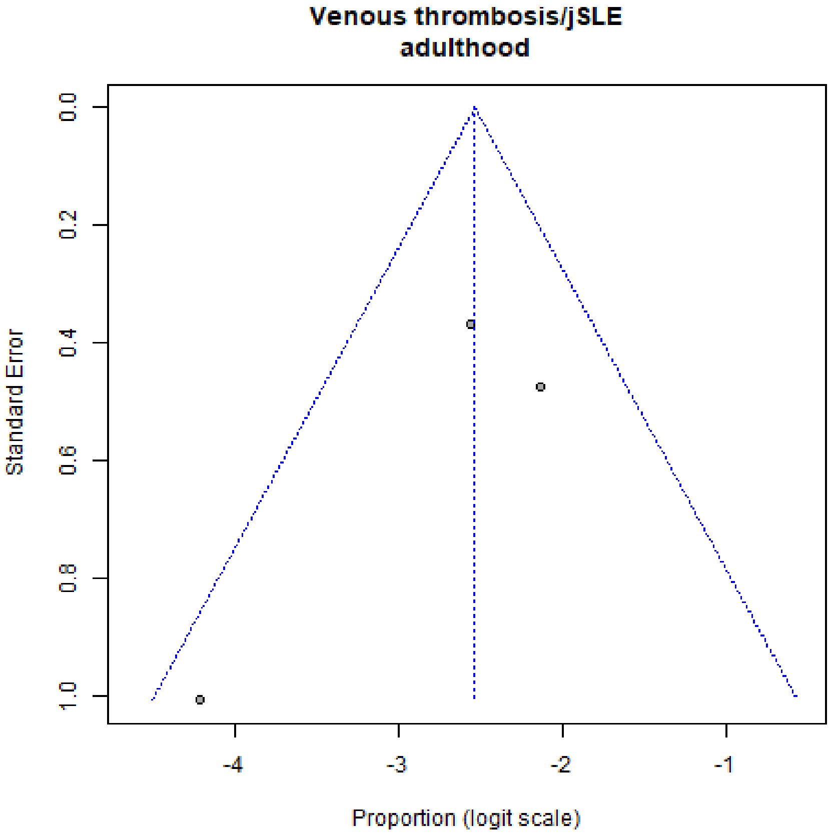

Summary statistics

```
                                    proportion  95%-CI           %W(random)
Groot N etal, 2019, The Netherlands     0.0721  [0.0316; 0.1371]       57.6
Tanigava, N. Y. etal, 2022, Brazil      0.0145  [0.0004; 0.0781]        7.7
Koutsonikoli A et al, 2015, Greece      0.1064  [0.0355; 0.2310]       34.7
Number of studies: k = 3; Number of observations: o = 227; Number of events: e = 14
                     proportion           95%-CI
Random effects model     0.0735 [0.0439; 0.1204]
Quantifying heterogeneity (with 95%-CIs):  tau^2 < 0.0001 [0.0000; 47.4982]; tau = 0.0006 [0.0000; 6.8919]
 I^2 = 43.5% [0.0%; 83.1%]; H = 1.33 [1.00; 2.43]
Test of heterogeneity:     Q d.f. p-value
                        3.54    2  0.1704
```

JDM/Childhood

Supplementary Figure S68: Forest and Funnel Plots of studies reporting the prevalence of calcinosis in children with Juvenile Dermatomyositis

Calcinosis

Forest

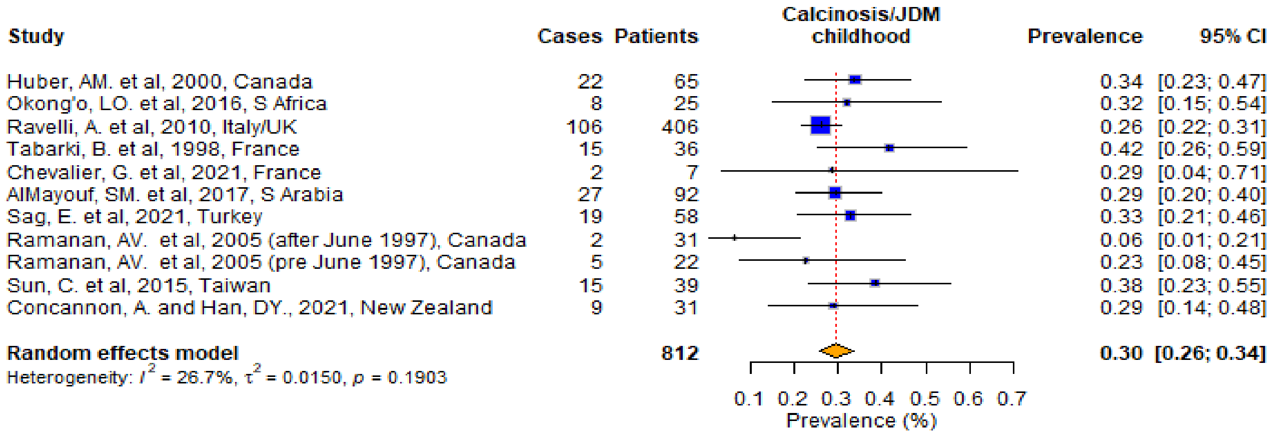

Funnel

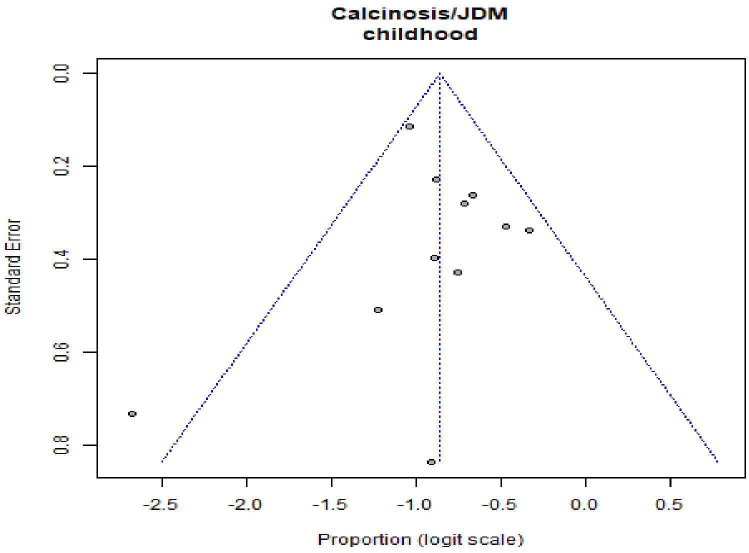

Summary statistics

```
                                                  proportion  95%-CI           %W(random)
Huber, AM. et al, 2000, Canada                        0.3385  [0.2257; 0.4665]        11.2
Okong'o, LO. et al, 2016, S Africa                    0.3200  [0.1495; 0.5350]         4.7
Ravelli, A. et al, 2010, Italy/UK                     0.2611  [0.2190; 0.3067]        33.6
Tabarki, B. et al, 1998, France                       0.4167  [0.2551; 0.5924]         7.2
Chevalier, G. et al, 2021, France                     0.2857  [0.0367; 0.7096]         1.3
AlMayouf, SM. et al, 2017, S Arabia                   0.2935  [0.2031; 0.3976]        13.9
Sag, E. et al, 2021, Turkey                           0.3276  [0.2101; 0.4634]        10.0
Ramanan, AV.  et al, 2005 (after June 1997), Canada   0.0645  [0.0079; 0.2142]         1.7
Ramanan, AV.  et al, 2005 (pre June 1997), Canada     0.2273  [0.0782; 0.4537]         3.4
Sun, C. et al, 2015, Taiwan                           0.3846  [0.2336; 0.5538]         7.6
Concannon, A. and Han, DY., 2021, New Zealand         0.2903  [0.1422; 0.4804]         5.4
Number of studies: k = 11; Number of observations: o = 812; Number of events: e = 230
                     proportion           95%-CI
Random effects model     0.2970 [0.2591; 0.3381]
Quantifying heterogeneity (with 95%-CIs):  tau^2 = 0.0150 [0.0000; 0.7653]; tau = 0.1226 [0.0000; 0.8748]
 I^2 = 26.7% [0.0%; 63.7%]; H = 1.17 [1.00; 1.66]
Test of heterogeneity:       Q d.f. p-value
                         13.63   10  0.1903
```

Supplementary Figure S69: Forest and Funnel Plots of studies reporting the prevalence of cataract in children with Juvenile Dermatomyositis

Cataract

Forest

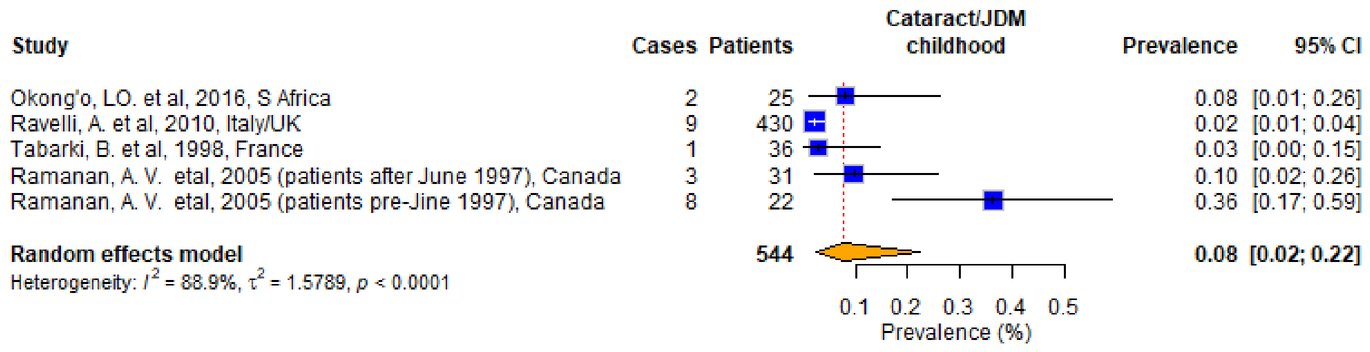

Funnel

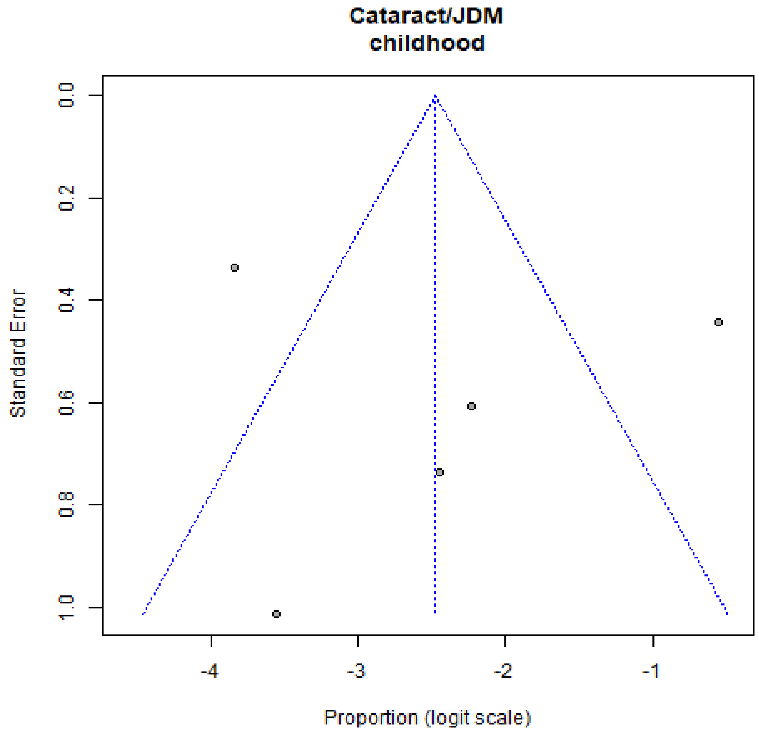

Summary statistics

```
                                                                  proportion          95%-CI           %W(random)
Okong'o, LO. et al, 2016, S Africa                                    0.0800    [0.0098; 0.2603]           18.7
Ravelli, A. et al, 2010, Italy/UK                                     0.0209    [0.0096; 0.0394]           23.4
Tabarki, B. et al, 1998, France                                       0.0278    [0.0007; 0.1453]           15.2
Ramanan, A. V.  etal, 2005 (patients after June 1997), Canada         0.0968    [0.0204; 0.2575]           20.4
Ramanan, A. V.  etal, 2005 (patients pre-Jine 1997), Canada           0.3636    [0.1720; 0.5934]           22.3
Number of studies: k = 5; Number of observations: o = 544; Number of events: e = 23
                     proportion           95%-CI
Random effects model     0.0775 [0.0239; 0.2239]
Quantifying heterogeneity (with 95%-CIs):  tau^2 = 1.5789 [0.3765; 13.6521]; tau = 1.2565 [0.6136; 3.6949]
 I^2 = 88.9% [76.9%; 94.7%]; H = 3.01 [2.08; 4.35]
Test of heterogeneity:       Q d.f.  p-value
                         36.16    4 < 0.0001
```

Supplementary Figure S70: Forest and Funnel Plots of studies reporting the prevalence of contractures in children with Juvenile Dermatomyositis

Contractures

Forest

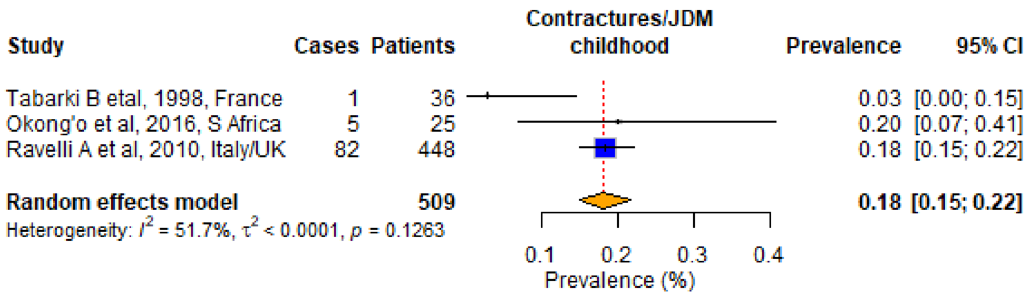

Funnel

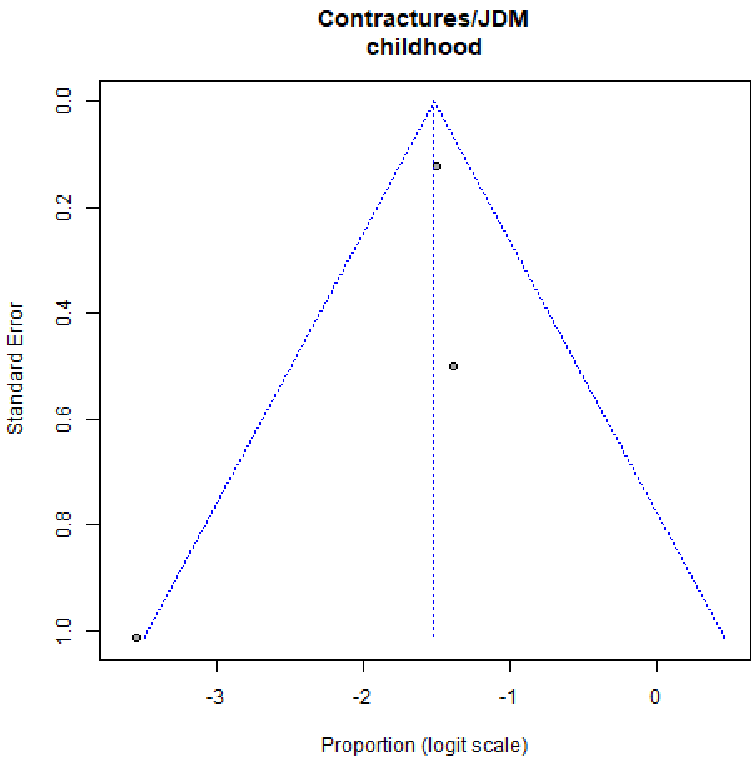

Summary statistics

```
                                proportion      95%-CI           %W(random)
Tabarki B etal, 1998, France        0.0278     [0.0007; 0.1453]        1.4
Okong'o et al, 2016, S Africa       0.2000     [0.0683; 0.4070]        5.6
Ravelli A et al, 2010, Italy/UK     0.1830     [0.1483; 0.2220]       93.1
Number of studies: k = 3; Number of observations: o = 509; Number of events: e = 88
                     proportion           95%-CI
Random effects model     0.1798 [0.1482; 0.2164]
Quantifying heterogeneity (with 95%-CIs):  tau^2 < 0.0001 [0.0000; 58.2361]; tau = 0.0020 [0.0000; 7.6313]
 I^2 = 51.7% [0.0%; 86.1%]; H = 1.44 [1.00; 2.68]
Test of heterogeneity:      Q d.f.  p-value
                         4.14    2   0.1263
```

Supplementary Figure S71: Forest and Funnel Plots of studies reporting the prevalence of diabetes (undefined) in children with Juvenile Dermatomyositis

Diabetes (undefined)

Forest

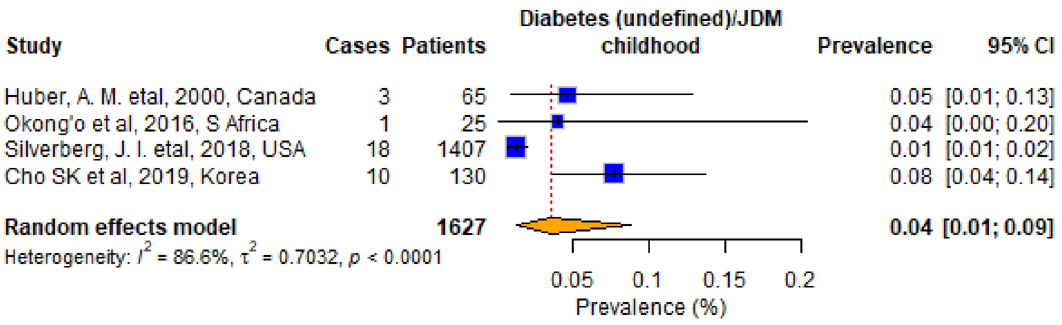

Funnel

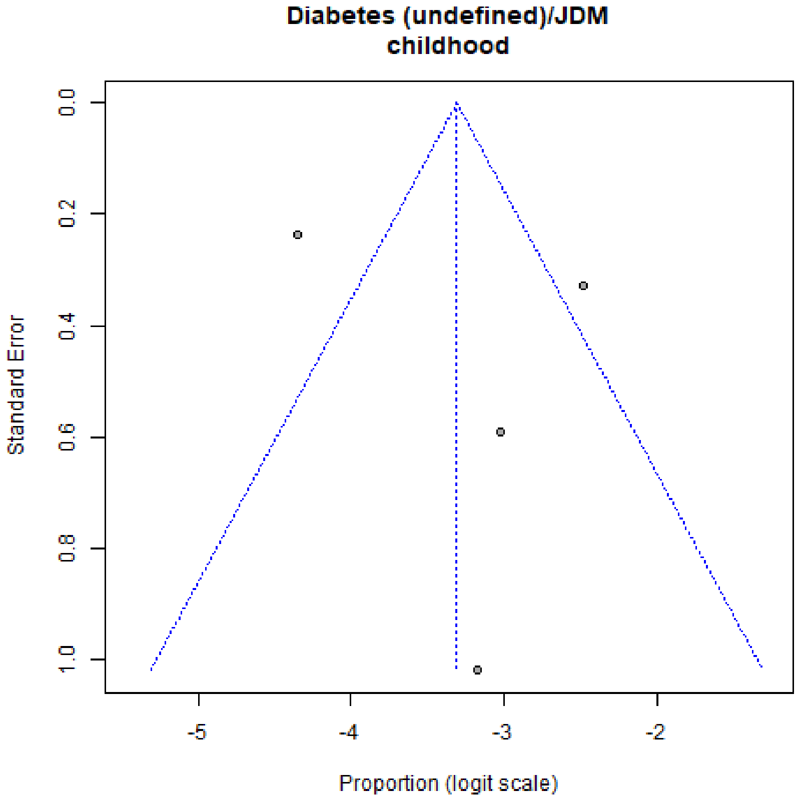

Summary statistics

```
                                   proportion    95%-CI          %W(random)
Huber, A. M. etal, 2000, Canada       0.0462    [0.0096; 0.1290]        23.3
Okong'o et al, 2016, S Africa         0.0400    [0.0010; 0.2035]        14.1
Silverberg, J. I. etal, 2018, USA     0.0128    [0.0076; 0.0201]        32.3
Cho SK et al, 2019, Korea             0.0769    [0.0375; 0.1369]        30.3
Number of studies: k = 4; Number of observations: o = 1627; Number of events: e = 32
                        proportion           95%-CI
Random effects model     0.0352 [0.0136; 0.0879]
Quantifying heterogeneity (with 95%-CIs):  tau^2 = 0.7032 [0.1139; 8.4433]; tau = 0.8386 [0.3376; 2.9057]
 I^2 = 86.6% [67.7%; 94.5%]; H = 2.74 [1.76; 4.25]
Test of heterogeneity:      Q d.f.  p-value
                        22.44    3 < 0.0001
```

Supplementary Figure S72: Forest and Funnel Plots of studies reporting the prevalence of growth failure in children with Juvenile Dermatomyositis

Growth failure

Forest

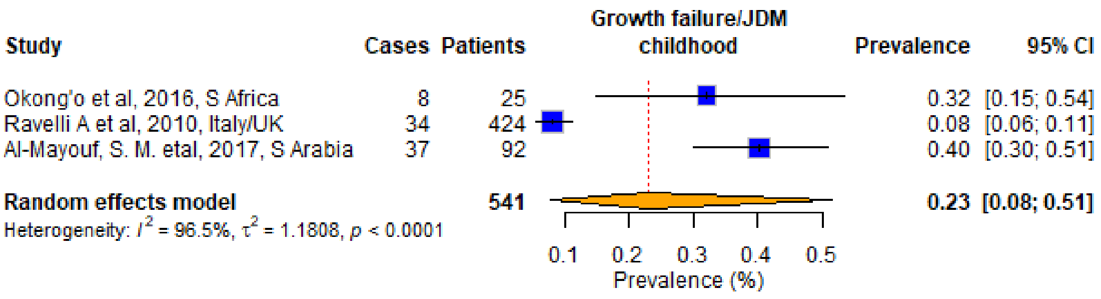

Funnel

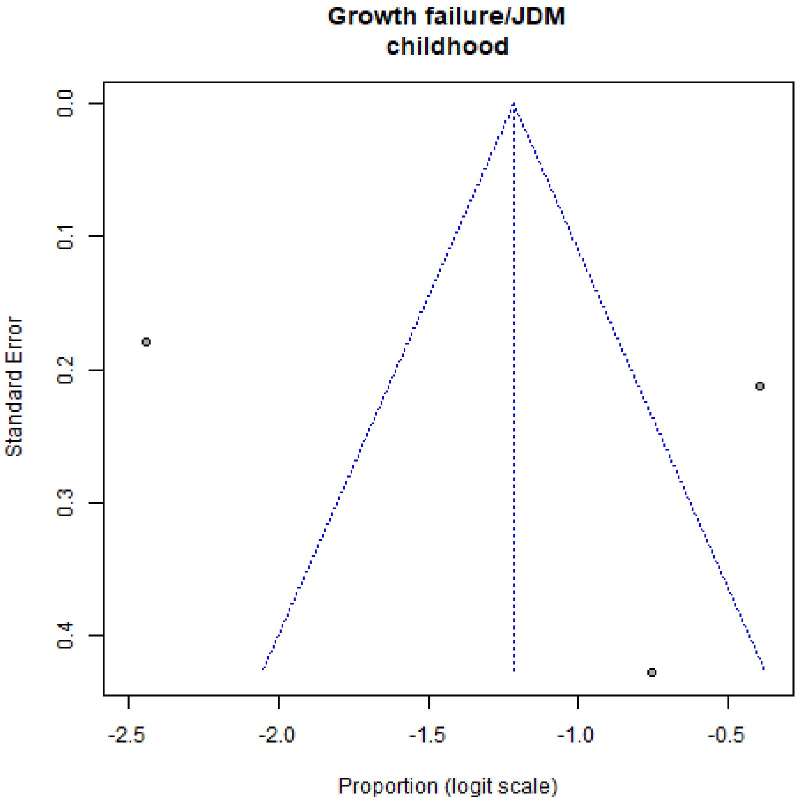

Summary statistics

```
                                        proportion        95%-CI          %W(random)
Okong'o et al, 2016, S Africa               0.3200    [0.1495; 0.5350]        30.9
Ravelli A et al, 2010, Italy/UK             0.0802    [0.0562; 0.1103]        34.7
Al-Mayouf, S. M. etal, 2017, S Arabia       0.4022    [0.3012; 0.5096]        34.4
Number of studies: k = 3; Number of observations: o = 541; Number of events: e = 79
                     proportion           95%-CI
Random effects model     0.2285 [0.0766; 0.5139]
Quantifying heterogeneity (with 95%-CIs):  tau^2 = 1.1808 [0.2769; 46.9921]; tau = 1.0866 [0.5262; 6.8551]
 I^2 = 96.5% [92.8%; 98.3%]; H = 5.37 [3.72; 7.74]
Test of heterogeneity:      Q d.f.  p-value
                        57.58    2 < 0.0001
```

Supplementary Figure S73: Forest and Funnel Plots of studies reporting the prevalence of Interstitial lung disease in children with Juvenile Dermatomyositis

Interstitial lung disease

Forest

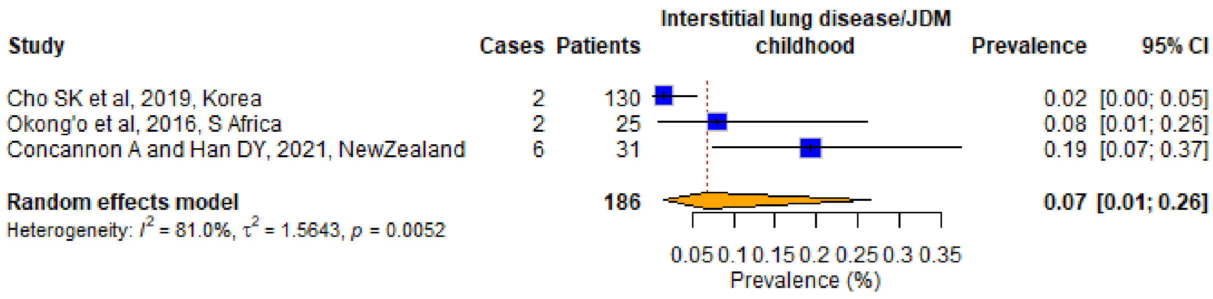

Funnel

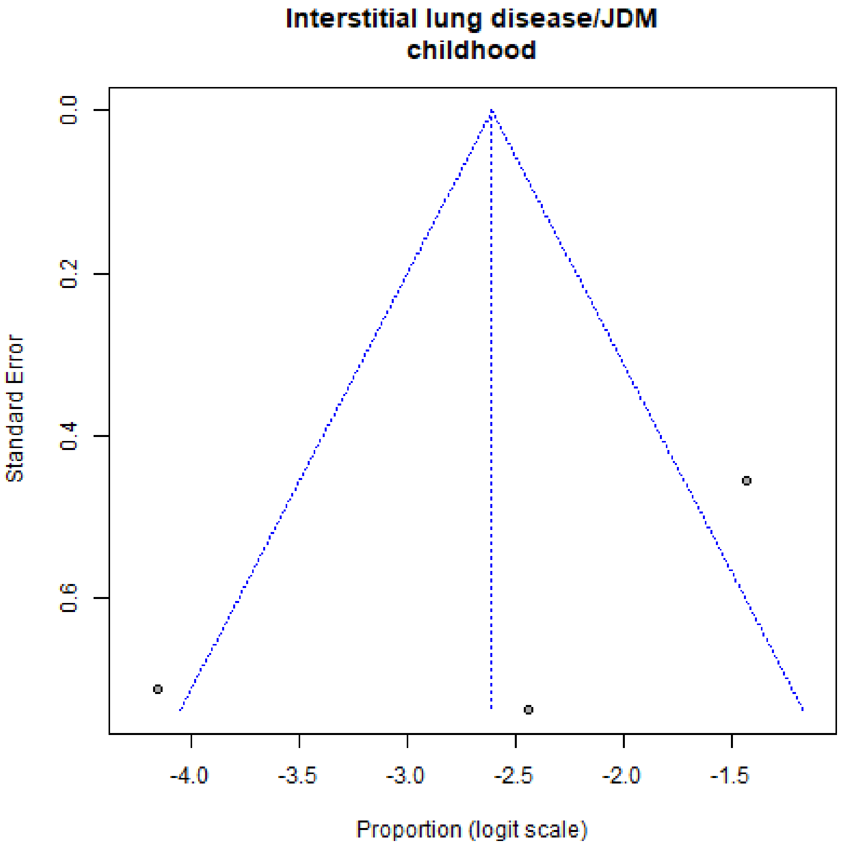

Summary statistics

```
                                          proportion     95%-CI           %W(random)
Cho SK et al, 2019, Korea                     0.0154    [0.0019; 0.0545]        31.7
Okong'o et al, 2016, S Africa                 0.0800    [0.0098; 0.2603]        31.2
Concannon A and Han DY, 2021, NewZealand      0.1935    [0.0745; 0.3747]        37.1
Number of studies: k = 3; Number of observations: o = 186; Number of events: e = 10
                     proportion           95%-CI
Random effects model     0.0685 [0.0148; 0.2648]
Quantifying heterogeneity (with 95%-CIs):  tau^2 = 1.5643 [0.1487; 74.9226]; tau = 1.2507 [0.3856; 8.6558]
 I^2 = 81.0% [40.6%; 93.9%]; H = 2.29 [1.30; 4.05]
Test of heterogeneity:       Q d.f. p-value
                         10.52    2  0.0052
```

Supplementary Figure S74: Forest and Funnel Plots of studies reporting the prevalence of Osteoporosis/Osteopenia in children with Juvenile Dermatomyositis

Osteoporosis/ Osteopenia

Forest

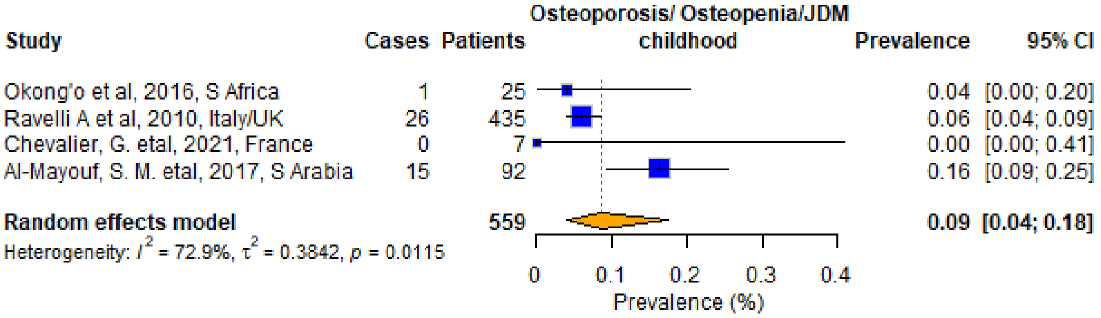

Funnel

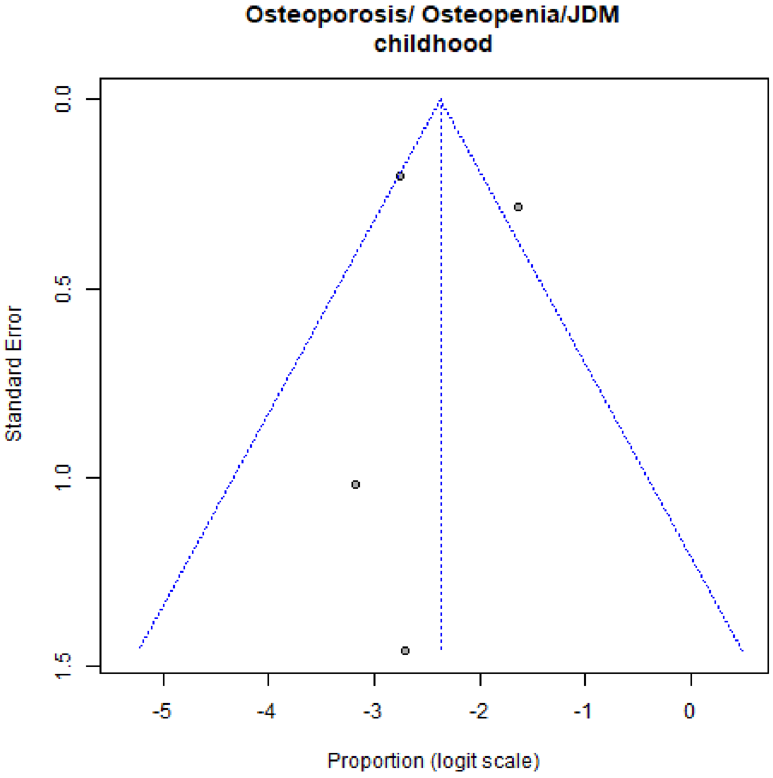

Summary statistics

```
                                      proportion        95%-CI          %W(random)
Okong'o et al, 2016, S Africa             0.0400       [0.0010; 0.2035]        12.5
Ravelli A et al, 2010, Italy/UK           0.0598       [0.0394; 0.0864]        42.0
Chevalier, G. etal, 2021, France          0.0000       [0.0000; 0.4096]        7.1
Al-Mayouf, S. M. etal, 2017, S Arabia     0.1630       [0.0942; 0.2546]        38.5
Number of studies: k = 4; Number of observations: o = 559; Number of events: e = 42
                     proportion           95%-CI
Random effects model     0.0851 [0.0391; 0.1756]
Quantifying heterogeneity (with 95%-CIs):  tau^2 = 0.3842 [0.0122; 5.6370]; tau = 0.6199 [0.1103; 2.3742]
 I^2 = 72.9% [23.4%; 90.4%]; H = 1.92 [1.14; 3.22]
Test of heterogeneity:       Q d.f. p-value
                         11.05    3  0.0115
```

Supplementary Figure S75: Forest and Funnel Plots of studies reporting the prevalence of lipodystrophy in children with Juvenile Dermatomyositis

Lipodystrophy

Forest

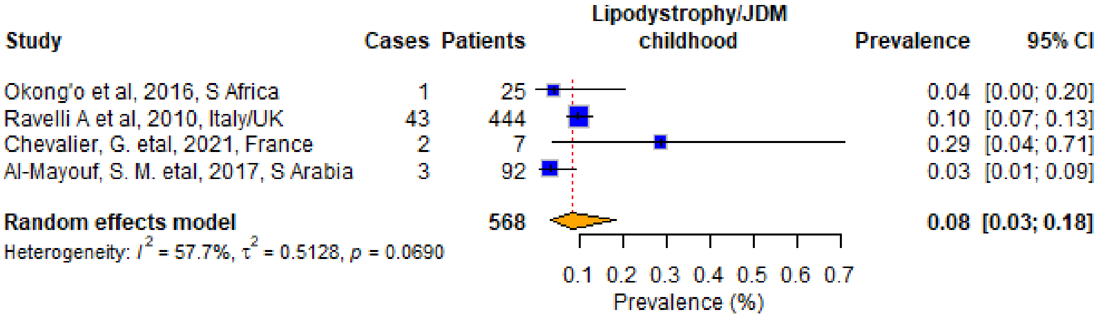

Funnel

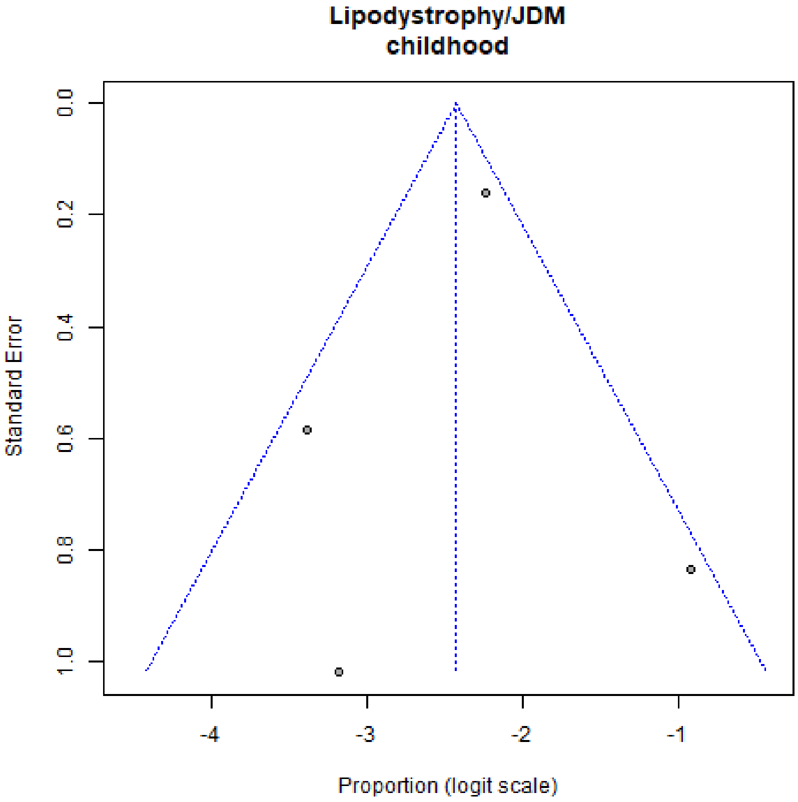

Summary statistics

```
                                        proportion        95%-CI          %W(random)
Okong'o et al, 2016, S Africa               0.0400    [0.0010; 0.2035]        14.3
Ravelli A et al, 2010, Italy/UK             0.0968    [0.0710; 0.1282]        41.3
Chevalier, G. etal, 2021, France            0.2857    [0.0367; 0.7096]        18.4
Al-Mayouf, S. M. etal, 2017, S Arabia       0.0326    [0.0068; 0.0923]        26.0
Number of studies: k = 4; Number of observations: o = 568; Number of events: e = 49
                     proportion           95%-CI
Random effects model     0.0811 [0.0338; 0.1821]
Quantifying heterogeneity (with 95%-CIs):  tau^2 = 0.5128 [0.0000; 17.0078]; tau = 0.7161 [0.0000; 4.1241]
 I^2 = 57.7% [0.0%; 85.9%]; H = 1.54 [1.00; 2.67]
Test of heterogeneity:     Q d.f. p-value
                        7.09    3  0.0690
```

Supplementary Figure S76: Forest and Funnel Plots of studies reporting the prevalence of malignancy in children with Juvenile Dermatomyositis

Malignancy

Forest

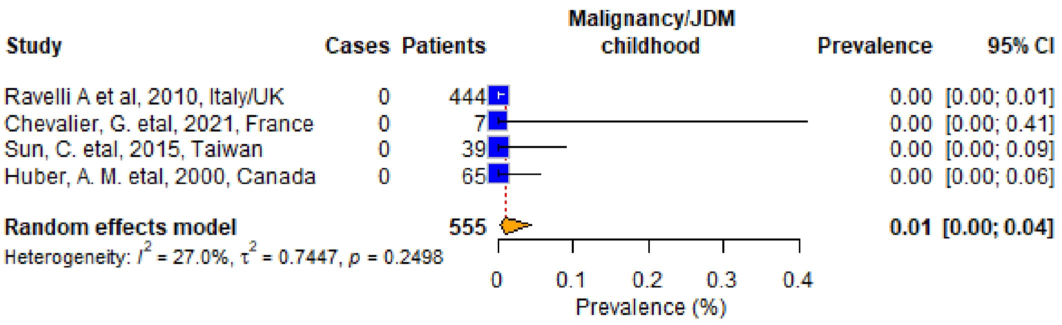

Funnel

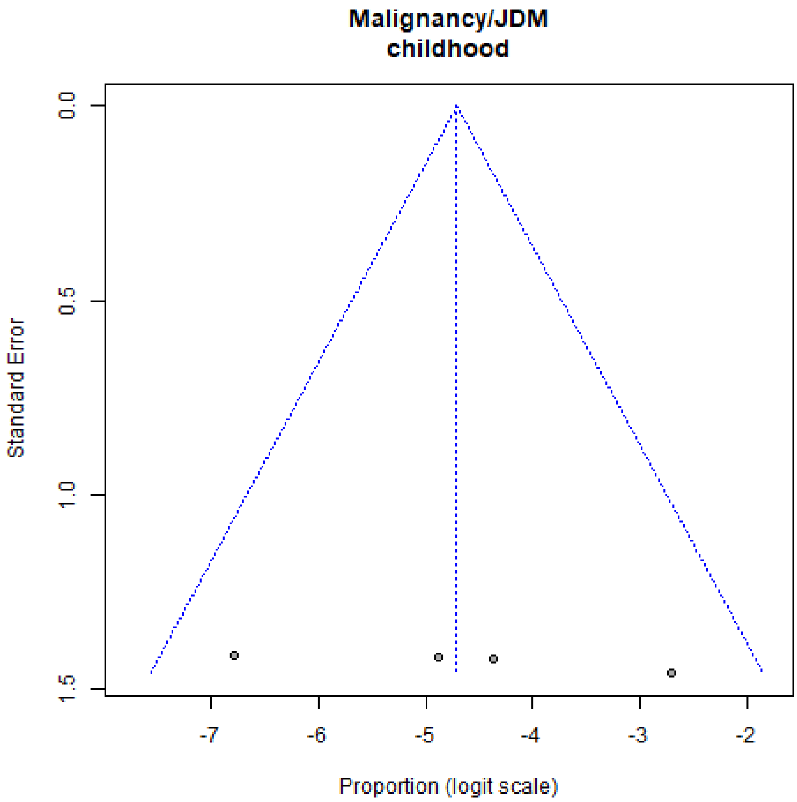

Summary statistics

```
                                  proportion     95%-CI          %W(random)
Ravelli A et al, 2010, Italy/UK       0.0000     [0.0000; 0.0083]
Chevalier, G. etal, 2021, France      0.0000     [0.0000; 0.4096]
Sun, C. etal, 2015, Taiwan            0.0000     [0.0000; 0.0903]
Huber, A. M. etal, 2000, Canada       0.0000     [0.0000; 0.0552]
Number of studies: k = 4; Number of observations: o = 555; Number of events: e = 0
                     proportion           95%-CI
Random effects model     0.0089 [0.0018; 0.0442]
Quantifying heterogeneity (with 95%-CIs):  tau^2 = 0.7447 [0.0000; 37.2127]; tau = 0.8629 [0.0000; 6.1002]
 I^2 = 27.0% [0.0%; 72.6%]; H = 1.17 [1.00; 1.91]
Test of heterogeneity:     Q d.f. p-value
                        4.11    3  0.2498
```

JDM/Adulthood

Supplementary Figure S77: Forest and Funnel Plots of studies reporting the prevalence of calcinosis in adults with Juvenile Dermatomyositis

Calcinosis

Forest

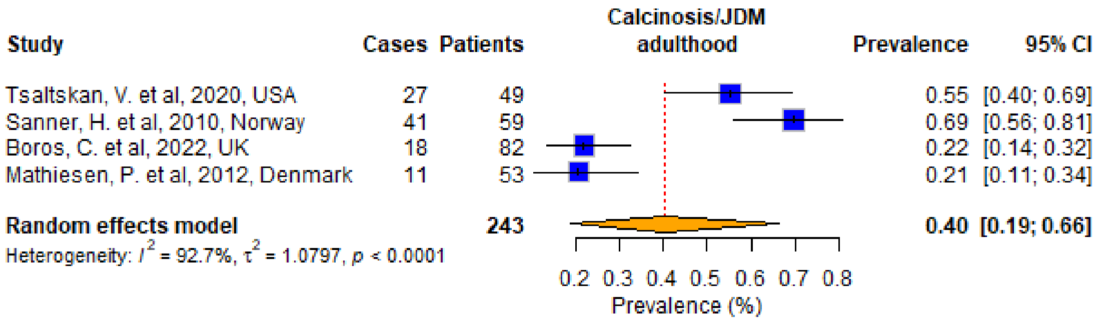

Funnel

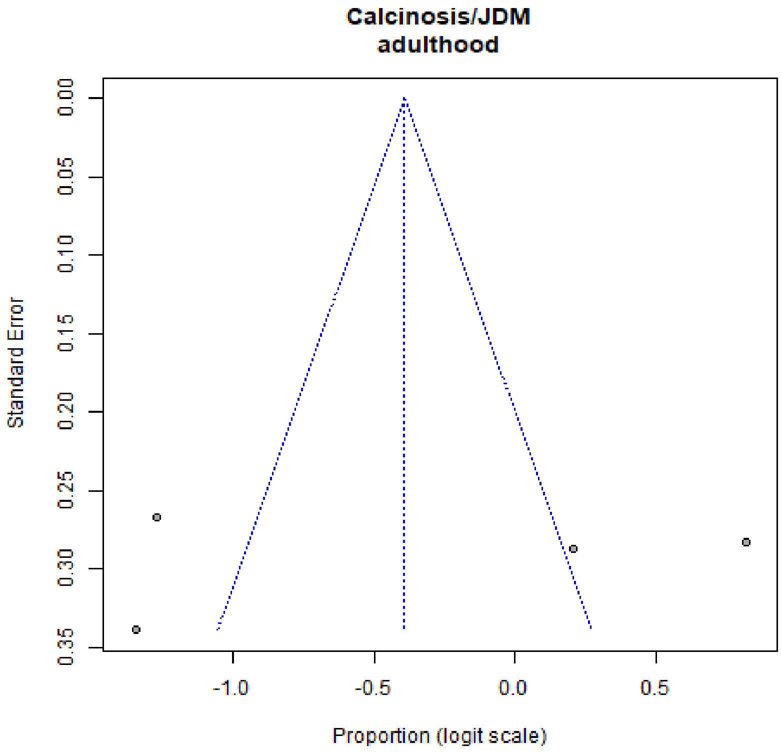

Summary statistics

```
                                    proportion   95%-CI          %W(random)
Tsaltskan, V. et al, 2020, USA          0.5510   [0.4023; 0.6933]        25.1
Sanner, H. et al, 2010, Norway          0.6949   [0.5613; 0.8081]        25.1
Boros, C. et al, 2022, UK               0.2195   [0.1356; 0.3246]        25.3
Mathiesen, P. et al, 2012, Denmark      0.2075   [0.1084; 0.3411]        24.4
Number of studies: k = 4; Number of observations: o = 243; Number of events: e = 97
                      proportion           95%-CI
Random effects model      0.4037 [0.1902; 0.6611]
Quantifying heterogeneity (with 95%-CIs):  tau^2 = 1.0797 [0.2878; 16.1291]; tau = 1.0391 [0.5365; 4.0161]
 I^2 = 92.7% [84.6%; 96.6%]; H = 3.71 [2.55; 5.40]
Test of heterogeneity:       Q d.f.  p-value
                         41.34    3 < 0.0001
```

Supplementary Figure S78: Forest and Funnel Plots of studies reporting the prevalence of lipodystrophy in adults with Juvenile Dermatomyositis

Lipodystrophy

Forest

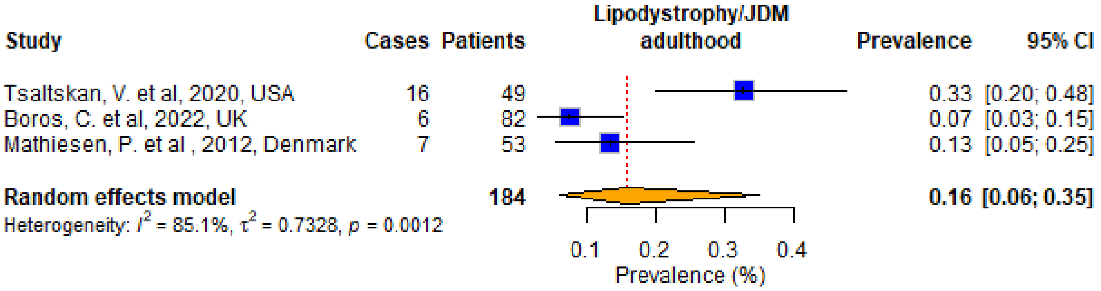

Funnel

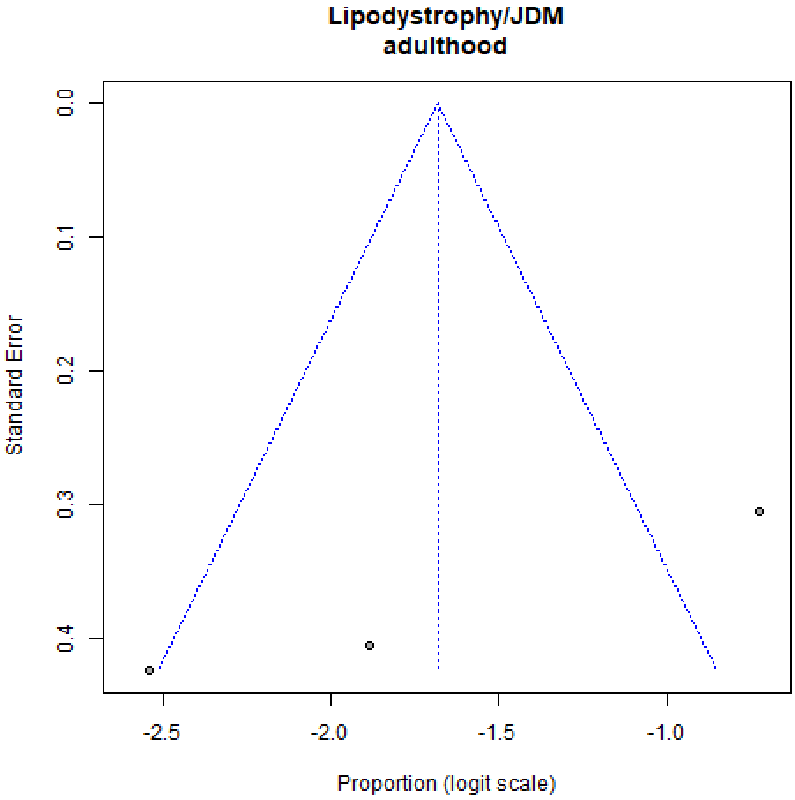

Summary statistics

```
                                    proportion  95%-CI            %W(random)
Tsaltskan, V. et al, 2020, USA          0.3265  [0.1995; 0.4754]        35.4
Boros, C. et al, 2022, UK               0.0732  [0.0273; 0.1525]        32.0
Mathiesen, P. et al , 2012, Denmark     0.1321  [0.0548; 0.2534]        32.6
Number of studies: k = 3; Number of observations: o = 184; Number of events: e = 29
                     proportion           95%-CI
Random effects model     0.1567 [0.0605; 0.3491]
Quantifying heterogeneity (with 95%-CIs):  tau^2 = 0.7328 [0.1017; 33.2331]; tau = 0.8560 [0.3189; 5.7648]
 I^2 = 85.1% [55.9%; 94.9%]; H = 2.59 [1.51; 4.44]
Test of heterogeneity:      Q d.f. p-value
                        13.38    2  0.0012
```
