# Supplementary material for: Comorbidities in Juvenile-Onset Rheumatic Diseases: A Systematic Review and Meta-Analysis
Source: Children (Basel). 2026 Jul 28;13(8):995. doi: 10.3390/children13080995 (PMC13511721; doi:10.3390/children13080995)
Supplement: Supplementary file 1 [file children-13-00995-s001.zip › SiddiqEtal_SupplementaryTables.pdf]

Supplementary Tables for Comorbidities in Juvenile-Onset Rheumatic Diseases: A Systematic Review and Meta-Analysis

Sab Siddiq [1,†], Shabnam Cheetham [2,†], Clare E. Pain [3,4], Eve M. D. Smith [5], Sizheng Steven Zhao [6,7], Liza J. McCann [3,4,*,‡] and David M. Hughes [1,*,‡]

1 Department of Health Data Science, Institute of Population Health, University of Liverpool, Waterhouse Building, Block F, 1–5 Brownlow Street, Liverpool L69 3GL, UK; sab.siddiq@liverpool.ac.uk
2 Department of Paediatric Rheumatology, Royal Manchester Children's Hospital, Manchester University NHS Foundation Trust, Manchester M13 9WL, UK; shabnam.cheetham@nhs.net
3 Department of Paediatric Rheumatology, Institute in the Park, Alder Hey Children's NHS Foundation Trust, Liverpool L14 5AB, UK; clare.pain@alderhey.nhs.uk
4 Department of Women and Children's Health, Institute of Life Course and Medical Sciences,
University of Liverpool, Liverpool L8 7SS, UK.
5 School of Infection and Immunity, University of Glasgow, Glasgow G12 8TA, UK; eve.smith@glasgow.ac.uk
6 Centre for Musculoskeletal Research, Faculty of Biology, Medicine and Health, School of Biological
Sciences, Division of Musculoskeletal & Dermatological Sciences, University of Manchester,
Oxford Road, Manchester M13 9PL, UK; steven.zhao.25@gmail.com
7 NIHR Manchester Biomedical Research Centre, Manchester University NHS Foundation Trust,
Manchester M13 9WU, UK
* Correspondence: liza.mccann@alderhey.nhs.uk (L.J.M.); dmhughes@liverpool.ac.uk (D.M.H.)
† Sab Siddiq and Shabnam Cheetham contributed equally to this study as joint first authors, with both authors contributing substantially to conceptualisation, data collection, analysis, drafting and revision of this manuscript.
‡ Liza J. McCann and David M. Hughes contributed equally to this study as joint senior authors.

Supplementary Table S1: Prevalence and incidence rates of comorbidities in JIA, jSLE and JDM patients presenting in childhood or apparent in adulthood

Study	Gender	Age in yrs	Onset age, yrs	Age diagnosis, yrs	Disease duration	Follow up period for the study	Comorbidity	Sample size, N (patients/ controls)	Prevalence	Incidence (per 1000 person-yrs)	Ethnicity /Race
**Childhood / JIA**											
Demirkaya, et al, 2011, Turkey[23]	F: 354	Mean 11.84±4.66 yrs			Mean 4.35 ±3.25 yrs	Mean 7.6±4.4 yrs		634			
							Macrophage Activation Syndrome	14	0.022082	2.905528806	
							Infections	14	0.022082	2.905528806	
							Uveitis	62	0.0977918	12.86734186	
Jain, et al, 2001, India[24]	F: 10	Mean 8.7± 3.2 yrs			Mean 16.4 ± 5.23 months			50			
							Keratoconjunctivitis sicca	7	0.14		
Rangel, et al, 2003, UK[25]	F:20	Mean 15.2±2.4 yrs	Mean 5.5±3.6 yrs		Mean 9.8 ± 3.8 yrs			30			Caucasian 27; Asian (SE) 3
							Major depressive episode	2/27	0.0740741		
							Over-anxious disorder/generalized anxiety disorder	3/27	0.1111111		
							Dysthymic disorder	2/27	0.0740741		
							Night terrors	1	0.0333333		
							Oppositional defiant disorder	1	0.0333333		
Saurenmann, et al, 2007, Canada[26]	F: 711	Mean 13.8 yrs (1.2 to 34.1)		Mean 6.9 yrs (0.5-17.8 yrs)	Mean 6.9 yrs (Range 0.08 to 25.7)	Mean 6.9 yrs (Range 0.08 to 25.7)		1081			
							Uveitis	142	0.1313599	19.03765971	
							Cataracts	33	0.0305273	4.424244862	
							Synechiae	31	0.0286772	4.15610881	
							Glaucoma	22	0.0203515	2.949496575	
							Band keratopathy	20	0.0185014	2.681360522	

						Macular oedema	7	0.0064755	0.938476183	
						Legal blindness	10	0.0092507	1.340680261	
						Impaired vision	6	0.0055504	0.804408157	
Chabra, et al, 2020, Canada[27]	F: 142	Median 16.9 yrs (IQR 12.9 - 18.0)			Median 5.6 yrs (IQR 3.3-8.3)		247			
						Uveitis	24	0.097166	17.35106998	
						Band keratopathy	1	0.0040486	0.722961249	
						Decreased visual acuity	1	0.0040486	0.722961249	
						Cataract	1	0.0040486	0.722961249	
						Micrognathia or facial asymmetry	11	0.0445344	7.952573742	
						Leg-length discrepancy	12	0.048583	8.675534991	
Beukelman, et al, 2012, USA[28]	F: 5,421	Mean 9.7±4.4 yrs			Median 1.1 yrs (IQR 0.5-2.2)		8479			White 4402; Black 1448; Latino 1721; other/unknown 932
						Upper respiratory tract	110	0.0129732	11.79384361	
						Pneumonia	87	0.0102606	9.327858131	
						Bacteraemia/septicaemia	67	0.0079019	7.183522928	
						Urinary tract/pyelonephritis	65	0.007666	6.969089408	
						Skin and soft tissue	44	0.0051893	4.717537445	
						Abdominal abscess	20	0.0023588	2.144335202	
						Gastroenteritis	30	0.0035382	3.216502804	
						Diabetes mellitus	56	0.0066046	6.004138567	
						Asthma	669	0.0789008	71.72801252	
van Rossum, et al, 2007, Netherlands[29]	F: 41	Median 18 yrs (range 10-25)		Median 10.7 yrs (range 8-23)	Median 9 yrs (IQR 8-9 yrs)		61			
						Uveitis	12	0.1967213	21.8579235	
Gerloni, et al, 2008, Italy[30]	F:124	Mean 17.1 yrs SD 9.2 yrs (range 1.9-49.8)	Mean 6.4 yrs SD 4.8 (Range 0.5-16.8)	Mean 10.7 yrs SD 8.6 (range 1.8-49.8)			163			
						Flare or new onset of chronic iridocyclitis	7	0.0429448		
						Gonadal effects	6	0.0368098		

Hypertension	8	0.0490798
Infective events	41 (split below)	
Recurrent URI or UTI or mycotic vaginitis	21	0.1288344
Viral thyroiditis	1	0.006135
Varicella	4	0.0245399
EBV infections	3	0.0184049
Single URI	3	0.0184049
Rotavirus gastro-enteritis	3	0.0184049
Helicobacter gastritis	1	0.006135
Scarlet fever	1	0.006135
Hip prosthetic infection	1	0.006135
CMV pneumonia complicated by MAS	1	0.006135
Pneumonia	1	0.006135
Impetigo	1	0.006135
Neuropsychiatric manifestations	53 (split below)	
Headache	10	0.0613497
Severe headache (dose-dependent)	4	0.0245399
Psychoses	2	0.0122699
Depression	3	0.0184049
Panic attacks	2	0.0122699
Anxiety	4	0.0245399
Anorexia nervosa	1	0.006135
Nervousness/hyperactivity	7	0.0429448
Memory and concentration deficit	1	0.006135
Aggressiveness	3	0.0184049
Fatigue	5	0.0306748
Hyperalgesia	1	0.006135
Pain amplification syndrome	1	0.006135
Sleepiness	2	0.0122699
Insomnia	1	0.006135
Vertigo	3	0.0184049
Paraesthesia	1	0.006135
Hypoglossal paralysis	1	0.006135

						Neuroalgodistrofy	1	0.006135		
Klotsche, et al, 2016, Germany[31]	F: 1,537	Mean 11.3 yrs (SD 4.6)	Mean 7.8 yrs (SD 3.7)	Mean 3.5 yrs (SD 3.6)			2263			
						Malignancies	8 (split below)			
						Acute lymphoblastic leukaemia	2	0.0008838		
						Thyroid gland carcinoma	1	0.0004419		
						Yolk sac carcinoma	1	0.0004419		
						Non-Hodgkin's lymphoma	1	0.0004419		
						Hodgkin's lymphoma	2	0.0008838		
						Oligodendroglioma	1	0.0004419		
						Uveitis	361	0.1595228		
						inflammatory bowel diseases (IBD)	14 (split below)			
						Colitis	4	0.0017676		
						Crohn's disease	10	0.0044189		
						Infections*	471 (split below)			
						Bacterial	29	0.0128148		
						Viral	107 (split below)			
						Epstein Barr infection	8	0.0035351		
						Varicella zoster infection	34	0.0150243		
						Chickenpox	16	0.0070703		
						Herpes Zoster	18	0.007954		
						HPV	2	0.0008838		
Beukelman, et al, 2013, USA[32]	F: 5,421	Median 10.1 yrs (IQR 5.9-13.3)			Median 1.2 yrs (IQR 0.6-2.4)		8503			White 4,402; Black 1,448; Latino 1,721; Other/unknown 932
						Infections			(300/100,000 [95% CI 216-406] yrs split below)	
						Herpes zoster			225 /100,000 yrs (95% CI 154–318)	

				Salmonella		86/100,000 yrs (95% CI 44–150)
				Coccidioides		35/100,000 yrs (95% CI 11–81)
				Nontuberculous Mycobacterium		21/100,000 yrs (95% CI 4–61)
				Nocardia		7/100,000 yrs (95% CI 0.2-39)
				Pneumocystis		7/100,000 yrs (95% CI 0.2-39)
				Toxoplasma		7/100,000 yrs (95% CI 0.2-39)
				Blastomyces		0/100,000 yrs (95% CI 0.2-39)
				Histoplasma		0/100,000 yrs (95% CI 0-36)
				JC virus		0/100,000 yrs (95% CI 0-36)
				Cryptococcus		0/100,000 yrs (95% CI 0-36)
				Tuberculosis		0/100,000 yrs (95% CI 0-36)
				Legionella		0/100,000 yrs (95% CI 0-36)
				Listeria		0/100,000 yrs (95% CI 0-36)
				Aspergillus		0/100,000 yrs (95% CI 0-36)
Schenck, et al, 2018, Germany[33]	F: 8,043	Mean 11.6 yrs	Mean 4.2 yrs		12,269	

							Type 1 Diabetes	58	0.0047274		
							Type 2 Diabetes	2	0.000163		
							Mature onset diabetes of the young	2	0.000163		
Angeles-Han, et al, 2015, USA[34]	F: 208			Median 6.4 (2.9-11.7) yrs	Median 3.4, range (1.8 – 5.9) yrs	Mean 15.1± 12 months		287			Caucasian 221, African American 36, Others 30
							Uveitis	52	0.1811847	11.9989847	
							Cataracts	14	0.0487805	3.230495881	
							Glaucoma	7	0.0243902	1.615247941	
							Synechiae	12	0.0418118	2.76899647	
							Band keratopathy	11	0.0383275	2.538246764	
							Cystoid macular oedema	7	0.0243902	1.615247941	
							Cataract extraction	3	0.010453	0.692249117	
Thiele, et al, 2021, Germany[35]	F: 2,490	Median 12.3, IQR (8.6-15.2)						3258			Caucasian 95%, African and Asian 5%
							Pneumonia	30	0.0092081		
							Herpes zoster	34	0.0104359		
							Varicella	23	0.0070595		
							SAE infection	108	0.0331492		
							Infections	813 (split below)			
							Upper respiratory tract infection	412	0.1264579		
							Otitis media	28	0.0085942		
							Urinary tract infection	32	0.009822		
							Tonsillitis	67	0.0205648		
							Gastroenteritis	63	0.019337		
							Streptococcal infection	20	0.0061387		

Kearsley-Fleet, et al, 2022, multiple countries[36]	F: 2,014	Mean, 6.5 (SD 4.6) Median 14, IQR (10–17) yrs		Median 1, IQR (0, 4) yrs, N=2894	Median 5, IQR (3, 9) yrs, N=2926		2693			
						MAS: macrophage activation syndrome (systemic JIA only)	37/250	0.148	29.6	
						Varicella	822/2238	0.3672922	73.45844504	
						Tuberculosis	17	0.0063127	1.262532492	
						Uveitis	556	0.2064612	41.29223914	
	F: 1,046	Mean 13.2 (SD 6.1) Median 22, IQR (19–25) yrs		Median 3, IQR (1, 7) yrs, N=1531	Median 14, IQR (7, 18) yrs, N=1514		1541			
						Uveitis	238	0.1544452	11.03179753	
						MAS: macrophage activation syndrome (systemic JIA only)	5/82	0.0609756	4.355400697	
						Varicella	127/871	0.1458094	10.41495818	
						Tuberculosis	2/1541	0.0012979	0.092704181	
	F : 2,476	Mean 6.5 SD (4.5) Median 13, IQR (9–17) yrs		Median 3, IQR (1–6) yrs	Median 6, IQR (3–9) yrs		3562			
						Uveitis	676/3484	0.1940299	32.33830846	
						MAS: macrophage activation syndrome (systemic JIA only)	62/366	0.1693989	28.23315118	
						Varicella	1166/2312	0.5043253	84.05420992	
						Tuberculosis	54/3006	0.0179641	2.994011976	
Krause, et al, 2017, USA[37]	F: 57	Mean 8.6 (±5.1) yrs at index		Median 1.3, range (0.2–4.0) yrs	Mean 6.3 (±4.4) yrs		89			Caucasian 75, Hispanic 2, Black 4, Asian 3, Other 1, Unknown 4
						Joint surgeries	6	0.0674157	10.70090958	
						Non-joint surgeries	14	0.1573034	24.96878901	
						Non-surgical hospitalisations (due to infection, JIA management etc.)	12	0.1348315	21.40181915	
						Hospitalisations for depression	5	0.0561798	8.917424648	

				Pregnancies	9/28	0.3214286	51.02040816
				Depression	7	0.0786517	12.48439451
Lee, et al, 2020, USA[38]	F: 10,233	Mean 13.86 ±3.44 yrs	Mean 2.68 yrs SD 2.34		15210		
				Type 1 diabetes	18	0.0011834	44/100,000 person yrs (95% CI 28-70)
				Type 2 diabetes	27	0.0017751	66/100,000 person yrs (95% CI 45-96)
				Hyperlipidaemia	191.646	0.0126	4.701492537
				Hypertension	158.184	0.0104	3.880597015
				Obesity	305.721	0.0201	7.5
Horneff, et al, 2022, Germany[39]		aged 2 to 15 yrs			546		
				Allergic rhinitis	76	0.1391941	
				Predominantly allergic asthma	15	0.0274725	
				Amyloidosis	<5	<5/546	
				Anaemia	15	0.0274725	
				Phobic anxiety disorders	17	0.0311355	
				Essential hypertension	<5	<5/546	
				Atopic dermatitis	80	0.1465201	
				Persistent somatoform pain disorder	46	0.0842491	
				Ulcerative colitis	<5	<5/546	
				Depression	21	0.0384615	
				Diabetes mellitus	5	0.0091575	
				Iron deficiency	8	0.014652	
				Post-viral fatigue syndrome	0	0	
				Fibromyalgia	13	0.0238095	
				Autoimmune thyroiditis	6	0.010989	
				Thyrotoxicosis	0	0	
				Hypothyroidism	8	0.014652	
				Lack of expected normal physiological development	26	0.047619	

Crohn’s disease	12	0.021978
Migraine	14	0.025641
Kidney disease (chronic)	<5	<5/546
Psoriasis	21	0.0384615
Sicca syndrome	5	0.0091575
Uveitis	59	0.1080586
	849	
Allergic rhinitis	94	0.1107185
Predominantly allergic asthma	25	0.0294464
Amyloidosis	11	0.0129564
Anaemia	24	0.0282686
Phobic anxiety disorders	30	0.0353357
Essential (primary) hypertension	<5	<5/849
Atopic dermatitis	105	0.1236749
Persistent somatoform pain disorder	71	0.0836278
Ulcerative colitis	<5	<5/849
Depression	19	0.0223793
Diabetes mellitus	7	0.008245
Iron deficiency	7	0.008245
Post-viral fatigue syndrome	<5	<5/849
Fibromyalgia	9	0.0106007
Autoimmune thyroiditis	13	0.0153121
Thyrotoxicosis	<5	<5/849
Hypothyroidism	18	0.0212014
Lack of expected normal physiological development	31	0.0365135
Crohn‘s disease	<5	<5/849
Migraine	29	0.0341578
Kidney disease (chronic)	0	0
Osteoporosis	5	0.0058893
Psoriasis	39	0.0459364
Sicca syndrome	<5	<5/849
Uveitis	90	0.1060071

Bolt, IB. et al, 2008, Switzerland[40]	F: 174	≤18 yrs (see comments)	Mean 6.49, range (0.67–15.67) yrs	Mean 4.82, range (0.08–16.58) yrs		265		
					Synechiae	10	0.0377358	7.829014327
					Cataracts	9	0.0339623	7.046112894
					Band keratopathy	5	0.0188679	3.914507164
					Glaucoma	3	0.0113208	2.348704298
					Macular oedema	2	0.0075472	1.565802865
					Uveitis	35	0.1320755	27.40155014
					Impaired visual acuity	1	0.0037736	0.782901433
					Legal blindness in one eye	2	0.0075472	1.565802865
Simon, et al, 2020, USA[41]		< 18 yrs						
	F: 6,173	Mean 10.6±4.5 yrs				8633		
					Multiple sclerosis	1	0.0001158	
					Asthma	777	0.0001158	
					Anxiety	295	0.0001158	
					Depression	208	0.0001158	
					Diabetes mellitus	92	0.0001158	
					Anaemia	498	0.0001158	
					Hypertension	2	0.0001158	
					Hyperlipidaemia	174	0.0001158	
					Malignancy	56	0.0001158	
	F: 6,587	Mean 10.5±4.5 yrs				9155		
					Multiple sclerosis	1	0.0001092	
					Asthma	887	0.0001092	
					Anxiety	317	0.0001092	
					Depression	214	0.0001092	
					Diabetes mellitus	84	0.0001092	
					Anaemia	479	0.0001092	
					Hypertension	6	0.0001092	
					Hyperlipidaemia	202	0.0001092	

						Malignancy	49	0.0001092	
Schulz, C. et al, 2022, Germany[42]	F 333	Mean 6.8 yrs (range: 2-22)	Median 6.0 yrs (<1-16.4)		Median 4.4 yrs (range: 0.9–14.1)		499		Europe 413, Asia 31, Turkish 28 , Africa Arab 2, mixed countries of origin 30 and unknown 23
						Hypothyroidism	6	0.012024	
						Autoimmune thyroiditis	8	0.0160321	
						Other thyroid disorder	2	0.004008	
						Coeliac disease	3	0.006012	
						Scleroderma	1	0.002004	
						Overlap syndrome	1	0.002004	
Swart, et al, 2018, Italy[43]	F: 5,584	≤ 18 yrs	Median 5.4 yrs (IQR : 2.4–10.0)	Med 6.2 (IQR 2.8–10.9) yrs	Med 5.3 (IQR 2.7–8.8) yrs		8274		
						Infections	1523 (split below)		
						Serious/targeted infections (Epstein– Barr virus, cytomegalovirus, papilloma virus, herpes zoster primary and reactivation, and opportunistic infections)	674	0.08146	
						Tuberculosis	27	0.0032632	
						Other infections	822	0.0993474	
						Macrophage activation syndrome	75	0.0090645	
						Autoimmune disease	50 (split below)		
						Inflammatory bowel disease (IBD)	21	0.0025381	
						Multiple sclerosis	2	0.0002417	
						Other autoimmune diseases excluding IBD, uveitis, and demyelinisation disorder	18	0.0021755	
						Malignancies	16 (split below)		
						Leukaemia’s	3	0.0003626	
						Lymphomas	2	0.0002417	

			Hematopoietic neoplasms (excluding leukaemia and lymphoma)	1	0.0001209
			Neoplasm (other)	10	0.0012086
			Gastrointestinal (GI) ulcer/GI bleed/GI perforation	17	0.0020546
			Pregnancy	9	0.0010877
			Congestive heart failure	1	0.0001209
F: 2,670	Median 7.6 yrs (IQR 3.2–11.7)	Med 6.1 (IQR 3.5–9.5) yrs		3990	
			Infections	1509 (split below)	
			Serious/targeted infections (Epstein– Barr virus, cytomegalovirus, papilloma virus, herpes zoster primary and reactivation, and opportunistic infections)	171	0.0428571
			Tuberculosis	0	0
			Other infections	1338	0.3353383
			Macrophage activation syndrome	11	0.0027569
			Autoimmune disease	50 (split below)	
			Inflammatory bowel disease (IBD)	23	0.0057644
			Multiple sclerosis	0	0
			Other autoimmune diseases excluding IBD, uveitis, and demyelinisation disorder	24	0.006015
			Malignancies	13 (split below)	
			Leukaemia	2	0.0005013
			Lymphoma	5	0.0012531
			Hematopoietic neoplasms (excluding leukaemia and lymphoma)	2	0.0005013
			Neoplasm (other)	4	0.0010025
			Gastrointestinal (GI) ulcer/GI bleed/GI perforation	4	0.0010025
			Pregnancy	7	0.0017544

					Congestive heart failure	0	0	
Giancane, et al, 2019, Italy[44]	F: 189	Med 3.1 yrs (IQR 1.8–5.8)	Med 7.7 yrs (IQR 6.1–10.2)			239		
					Ocular	21	0.0878661	
					Muscular atrophy	19	0.0794979	
					Osteoporosis	3	0.0125523	
					Subcutaneous atrophy	9	0.0376569	
					Growth failure	19	0.0794979	
					Pubertal delay	5	0.0209205	
					Avascular necrosis	0	0	
					Amyloidosis	0	0	
					Diabetes	0	0	
	F: 219	Med 2.5 yrs (IQR 1.6–4.7)	Med 6.9 yrs (IQR 5.4–9.7)			269		
					Ocular	7	0.0260223	
					Muscular atrophy	8	0.0297398	
					Osteoporosis	1	0.0037175	
					Avascular necrosis	1	0.0037175	
					Subcutaneous atrophy	6	0.0223048	
					Growth failure	1	0.0037175	
					Diabetes	0	0	
					Amyloidosis	0	0	
Grassi, et al, 2007, Italy[45]	F: 244	mean 4.9 ± 3.6 yrs		mean 7.6 ± 5.6 yrs		309		
					Synechiae	12	0.038835	5.109862034
					Band keratopathy	8	0.02589	3.406574689
					Cataract	6	0.0194175	2.554931017
					Glaucoma	2	0.0064725	0.851643672
					Retinal detachment	1	0.0032362	0.425821836
					Iris atrophy	1	0.0032362	0.425821836
					Blindness*	4	0.012945	1.703287345
					Uveitis	62	0.2006472	26.40095384

							Severe visual loss	8	0.02589	3.406574689
Horneff, et al, 2016, Germany[46]						13,198 observation yrs		3695		
							Suspected Malignancies	12 (split below)		
							Hodgkin's lymphoma	2	0.0005413	4.10117E-05
							non-Hodgkin's lymphoma	1	0.0002706	2.05058E-05
							acute lymphatic leukaemia (ALL)	2	0.0005413	4.10117E-05
							lymphoproliferative disorder	1	0.0002706	2.05058E-05
							thyroid carcinoma	1	0.0002706	2.05058E-05
							yolk sac carcinoma	1	0.0002706	2.05058E-05
							anaplastic ependymoma	1	0.0002706	2.05058E-05
Lovell, DJ. et al, 2021, USA[47]	F: 867		Mean 6.6 yrs (SD 4.5)	Mean 8.0 (SD 4.8)				1332		
							Autoimmune diseases	1085 (split below)		
							Addison disease	11	0.0082583	
							Autoimmune/Hashimoto's thyroiditis	16	0.012012	
							Graves' disease	1	0.0007508	
							Morphoea	12	0.009009	
							Multiple sclerosis	1	0.0007508	
							Takayasu arteritis	1	0.0007508	
							Type 1 diabetes mellitus	17	0.0127628	
							Vitiligo	5	0.0037538	
							Myasthenia Gravis	0	0	
							Pernicious Anaemia	0	0	
							Primary biliary cirrhosis	0	0	
							Associated conditions		0	
							Autoimmune disease not elsewhere classified	8	0.006006	
							Chronic glomerulonephritis	1	0.0007508	
							Chronic urticaria	49	0.0367868	
							Episcleritis/scleritis	5	0.0037538	
							Erythema nodosum	1	0.0007508	
							Haemolytic anaemia	1	0.0007508	

							Immune thrombocytopenia purpura	2	0.0015015	
							Leukocytoclastic vasculitis	2	0.0015015	
							Pulmonary fibrosis/interstitial lung disease	15	0.0112613	
							Raynaud's syndrome	19	0.0142643	
							Sjögren's syndrome/sicca syndrome	3	0.0022523	
							Thrombocytopenia purpura	1	0.0007508	
							Vasculitis	8	0.006006	
Barthel, et al, 2015, Germany[48]	F: 2,073		Mean 6.1 ± 3.9 yrs			8389 patient-yrs		3071		
							Inflammatory bowel disease	11 (split below)		
							Crohn Disease	8	0.002605	0.000310527
							Ulcerative colitis	3	0.0009769	0.000116448
Sahin, et al, 2021, Turkey[49]	F: 289			8.8 ± 4.7 yrs (mean) (range 9 months to 17 yrs)	4.0 ± 1.9 yrs (mean)			500		
							Uveitis	34	0.068	
							Osteoporosis	8	0.016	
							Delayed puberty	6	0.012	
							Autoimmune Thyroiditis	4	0.008	
							Inflammatory Bowel Disease (IBD)	2	0.004	
							Depression	2	0.004	
							Hypertension	1	0.002	
							Amyloidosis	1	0.002	
							familial Mediterranean fever (FMF)	63	0.126	
							Psoriasis	15	0.03	
							Cataract	4	0.008	
							Band keratopathy	1	0.002	
							Posterior synechia	1	0.002	
Pohjankoski, et al, 2010, Finland[50]	F: 283			Mean 7 (1–15) yrs				417		
							Uveitis	91	0.2182254	
							Diabetes mellitus type 1	9	0.0215827	

							Coeliac disease	3	0.0071942	
							Hypothyroidism	3	0.0071942	
Cakan, et al, 2020, Turkey[51]	F: 202					Mean 29.2±18.0 months		405		
							Uveitis	27	0.0666667	2.283105023
Cosickic, et al, 2017, Bosnia and Herzegovina[52]	F: 61	Med 7.1 rs (IQR, 2.3 - 10.6 yrs)						97		
							Synechia	3	0.0309278	
							Band keratopathy	2	0.0206186	
							Cataracts	2	0.0206186	
							Glaucoma	1	0.0103093	
							Uveitis	14	0.1443299	
Berthold, et al, 2022, Sweden[53]	F: 433			Med 8.7 yrs ((IQR) 3.7 – 12.7 yrs)				640		
							Depressive disorder	93	0.1453125	
							Anxiety disorder	111	0.1734375	
Lien, et al, 2003, Norway[54]	F: 80	Mean 17.0 ±1.8 yrs	Mean 2.8±1.4 yrs		Mean 14.2 ±1.4 yrs			105		
							Osteopenia/Low total-body BMC	42/103	0.407767	
							Osteoporosis (BMD Z score≤-2.5)	5/103	0.0485437	
Saraux, et al, 2015, France[55]	F: 18	Mean 13.5 yrs (SD 5.84)						26		
							Infections	3	0.1153846	
							Bacterial infection	3	0.1153846	
							Viral infection	1	0.0384615	

Alberdi-Saugstrup, et al, 2017, Nordic countries[56]	F: 135		Median 6.0 (IQR 2.8-10.0)				193			
						Uveitis	30	0.1554404		
Amine, et al, 2011, Morrocco[57]	F: 32	Mean 11 ± SD 3.3 yrs range [2-16]	Mean 7 ±SD 3.4 range [1–15]	Mean 3 ± SD 2.4 yrs range [1-12]			58			
						Obesity	13	0.2241379		
Adulthood / JIA										
Smitherman, et al, 2022, USA[58]	F: 144	Mean 20±1.4 yrs	CARRA (or any other) JIA registry, Age onset will have childhood onset disease.				187			White 147; Hispanic 23; Black 9; Other* 8
						Self-reported symptoms of pain or discomfort	122	0.6524064		
						Self-reported symptoms of anxiety or depression	84	0.4491979		
Mena-Vazquez, et al, 2022, Spain[14]	F: 35	Mean 25 yrs +/- 6.1		161.4 months (103.6 - 220.4 IQR)	24 months		52			Caucasian 50
						Depression	13	0.25	125	
						Arterial hypertension	1	0.0192308	9.615384615	
						Diabetes melllitus	1	0.0192308	9.615384615	
						Dyslipidaemia	4	0.0769231	38.46153846	
						Obesity (BMI ≥30)	7	0.1346154	67.30769231	
						Uveitis	11	0.2115385	105.7692308	

Raab, et al, 2013, Germany[59]	F: 196	Mean 19.7 yrs (SD 2.8)	Mean 10.6 yrs (since symptom onset) 5.6 SD		344		
				Cardiovascular diseases	34 (split below)		
				Hypertension	25	0.0726744	6.856077227
				Cardiac insufficiency	1	0.002907	0.274243089
				Pulmonary diseases	28 (split below)		
				Asthma	26	0.0755814	7.130320316
				Gastrointestinal diseases	14 (split below)		
				Crohn’s disease	4	0.0116279	1.096972356
				Ulcerative colitis	3	0.0087209	0.822729267
				Gastrointestinal ulcer	1	0.002907	0.274243089
				Liver and gallbladder diseases	3	0.0087209	0.822729267
				Diseases of the kidneys or the Urinary tract	16 (split below)		
				Renal insufficiency	1	0.002907	0.274243089
				Kidney stone	4	0.0116279	1.096972356
				Amyloidosis	3	0.0087209	0.822729267
				Gynaecological diseases	12	0.0348837	3.290917069
				Haematological diseases	23 (split below)		
				Anaemia	11	0.0319767	3.01667398
				Iron deficiency	6	0.0174419	1.645458534
				Allergic diseases	84 (split below)		
				Allergic rhinitis	50	0.1453488	13.71215445
				House dust mite allergy	16	0.0465116	4.387889425
				Thyroid/endocrine diseases	23 (split below)		
				Hypothyroidism	7	0.0203488	1.919701624
				Hashimoto thyroiditis	4	0.0116279	1.096972356
				Diabetes mellitus	3	0.0087209	0.822729267
				Skin diseases	67 (split below)		

					Psoriasis	22	0.0639535	6.03334796
					Atopic dermatitis	30	0.0872093	8.227292672
					Eye diseases	80 (split below)		
					Uveitis	59	0.1715116	16.18034226
					Cataract	3	0.0087209	0.822729267
					Glaucoma	9	0.0261628	2.468187802
					Other eye diseases	8 (split below)		
					Benign eye tumour	1	0.002907	0.274243089
					Episcleritis	1	0.002907	0.274243089
					Scleritis	2	0.005814	0.548486178
					Sicca syndrome/ sicca symptoms	3	0.0087209	0.822729267
					Chronic conjunctivitis	1	0.002907	0.274243089
					Astigmatism	1	0.002907	0.274243089
					Neurologic diseases	41 (split below)		
					Migraine	30	0.0872093	8.227292672
					Epilepsy	5	0.0145349	1.371215445
					Psychiatric disorders	32 (split below)		
					Depression	17	0.0494186	4.662132514
					Anxiety disorder	7	0.0203488	1.919701624
					Other comorbidities	30 (split below)		
					Osteoporosis	11	0.0319767	3.01667398
Aggarwal, et al, 2006, India[60]	M: 30	22.6 ± 5.12 yrs	Mean 11.37 ± 2.67 yrs	Mean 11.27 ± 5.39 yrs		30		
					Osteopenia (Lumbar Spine)	14	0.4666667	41.40786749
					Osteopenia (Hip)	15	0.5	44.36557232
					Osteopenia (Distal third of forearm)	15	0.5	44.36557232
					Osteoporosis (Lumbar Spine)	8	0.2666667	23.66163857
					Osteoporosis (Hip)	7	0.2333333	20.70393375
					Osteoporosis (Distal third of forearm)	12	0.4	35.49245785
					Joint deformities	5	0.1666667	14.78852411

Arvidsson, et al, 2010, Norway[61]	F: 44	Mean 35.2 yrs +/- 3.6 (27.9-44.4)	Mean 5.5 yrs +/- 3.1 (0.8-14.5 yrs)		Mean 29.7 yrs +/- 3.3 yrs (24.4-38.0)	Mean 27 yrs		60			
							Craniofacial growth disturbance	34	0.5666667	20.98765432	
							Micrognathia	16	0.2666667	9.87654321	
Packham, et al, 2002, UK[62]	F: 176	Mean 35.4 yr (range 19-78, SD = 11.1)	Mean 7.1 yr (range 0.8–15.9, S.D.=4.5)		Mean 28.3 yr (range 8–73, S.D.=10.8)			246			Asian 1, Afro-Caribbean 1, Caucasian descent 244
							Uveitis	22%	0.22	7.77385159	
							Glaucoma	4.10%	0.041	1.448763251	
							Amyloidosis	8.90%	0.089	3.144876325	
							Cataracts	10.20%	0.102	3.604240283	
							Required eye surgery	10.60%	0.106	3.745583039	
							Sicca syndrome	15.00%	0.15	5.300353357	
							At least one major prosthetic joint replacement	51.2% (split below)			
							Hip prosthetic	43.90%	0.439	15.51236749	
							Revision hip prostheses	15.40%	0.154	5.441696113	
							Knee prosthetic	27.60%	0.276	9.752650177	
							Shoulder prosthetic	4.90%	0.049	1.731448763	
							Elbow prosthetic	3.30%	0.033	1.166077739	
							Wrist prosthetic	1.60%	0.016	0.565371025	
							Ankle prosthetic	1.60%	0.016	0.565371025	
Mars, et al, 2019, Finland[63]	F: 92	Mean 32.4 ± 13.4 yrs						119			
							Cardiovascular diseases*	17	0.1428571		
							Diabetes	5	0.0420168		
							Eye disorders	16	0.1344538		
							Gastrointestinal disorders	11	0.092437		
							Infections	27	0.2268908		

							Malignancies	2	0.0168067	
							Mental disorders	12	0.1008403	
							Neurological disorders	18	0.1512605	
							Other musculoskeletal disorders	38	0.3193277	
							Rheumatic diseases	76	0.6386555	
							Skin disorders	19	0.1596639	
Dimopoulou, et al, 2017, Greece[64]	F: 72	Mean 25 yrs SD 5.9, median 24 yrs range 8-41 yrs	Median 7 yrs Mean 7.7 yrs SD 4 (range 1-15.5)		Median 17 yrs range 5-31.5	Mean 17.2 yrs SD 6.7 yrs		102		
							Uveitis	11	0.1078431	6.26994984
							Prosthetic arthroplasty	9	0.0882353	5.12995896
							Mandibular correction	1	0.0098039	0.56999544
							Arthrodesis of the radiocarpal joint	1	0.0098039	0.56999544
							Hands or foot small joint correction	4	0.0392157	2.27998176
							Blindness in one eye	2	0.0196078	1.13999088
							Leg length discrepancy ≥0.5cm	37	0.3627451	21.08983128
							Mandibular dysfunction (such as reduced mouth opening, frontal facial asymmetry or retrognathia)	29.40%	0.0028824	0.167578659
Rebane, et al, 2022, Finland[65]	F: 62	Mean 23 yrs (range 16-23)				17 yrs		97		
							Coeliac disease	1	0.0103093	0.606428138
							Crohn's disease	2	0.0206186	1.212856277
							Irritable bowel syndrome (IBS)	2	0.0206186	1.212856277
							Hypothyroidism	1	0.0103093	0.606428138
							Depression	2	0.0206186	1.212856277
							Panic disorder	2	0.0206186	1.212856277
							Uveitis	4	0.0412371	2.425712553
							Ulcerative colitis	0	0	0
Minden, et al, 2019, Germany[66]	F: 464	Mean 23.4±3.9 yrs			Mean 14.3±6.1 yrs	Mean 9.1±3.7 yrs		701		
							Arthroplasty	36	0.0513552	5.643429324
							Eye surgery	22	0.0313837	3.448762365

				Uveitis	63	0.0898716	9.876001317	
				Synovectomy	112	0.1597718	17.55733567	
Smith, et al, 2019, USA[67]	F: 170	Mean 30.55±0.39 yrs (Age at estimated due date)	Mean 20.32±7.32 yrs		170			White 145; African American 3; Asian/Pacific islander 2; Native America 3; Other 1
				Comorbid pregestational conditions				
				Thyroid dysfunction	13	0.0764706		
				Hypertension	21	0.1235294		
				Diabetes Mellitus (Type 1 and 2)	0	0		
				Other autoimmune diseases				
				Inflammatory bowel disease	11	0.0647059		
				Sjögren's syndrome or UCTD	3	0.0176471		
				Antiphospholipid syndrome	1	0.0058824		
				Psoriasis	11	0.0647059		
				Lupus	4	0.0235294		
				Obstetric complications				
				Preeclampsia	15	0.0882353		
				PIH without preeclampsia	10	0.0588235		
				Placenta previa and/or abruption	4	0.0235294		
				Preterm labour	25	0.1470588		
				GDM	13	0.0764706		
				Fever during pregnancy	47	0.2764706		
				Caesarean section	70	0.4117647		

Barth, et al, 2016, Germany[68]	F: 1,617	range (18–73) yrs	Median 8, range (0–15) yrs IQR (4-11)	Median 27; range (3–70) yrs IQR (15-40)			2592		
						Pain/discomfort	(split below)		
						Moderate problems	1395	0.5381944	
						Severe problems	94	0.0362654	
						Anxiety/depression:	(split below)		
						Moderate problems	624	0.2407407	
						Severe problems	54	0.0208333	
						Severe disability	783	0.3020833	
Simon, et al, 2020, USA[41]		≥ 18 yrs			1 year				
	F: 4,506	Mean 37.0 ±16.5 yrs					5789 (split below)		
						Multiple sclerosis	20	0.0034548	3.454828122
						Asthma	472	0.0815339	81.53394369
						Anxiety	555	0.0958715	95.87148039
						Depression	653	0.1128001	112.8001382
						Diabetes mellitus	440	0.0760062	76.00621869
						Anaemia	948	0.1637589	163.758853
						Hypertension	103	0.0177924	17.79236483
						Hyperlipidaemia	1162	0.2007255	200.7255139
						Malignancy	278	0.0480221	48.0221109
	F: 4,315	Mean 34.6 ±14.7 yrs					5638 (split below)		
						Multiple sclerosis	19	0.00337	3.369989358
						Asthma	486	0.0862008	86.20078042
						Anxiety	549	0.097375	97.37495566
						Depression	622	0.1103228	110.3228095
						Diabetes mellitus	371	0.0658035	65.80347641

						Anaemia	655	0.1161759	116.1759489
						Hypertension	103	0.0182689	18.26888968
						Hyperlipidaemia	1023	0.1814473	181.4473217
						Malignancy	162	0.0287336	28.73359347
Packham and Hall, 2003, UK[69]	F: 177	Mean 35.4±11.1 yrs (19-78)	Mean 7.1±4.2 yrs (range 0.8-15.9)	Mean 28.7±10.8 yrs (range 8-73)			177		
						Primary ovarian failure	9 (split below)		
						Primary ovarian failure (unrelated to medication use)	6	0.0338983	1.181125613
Ma, et al, 2022, Taiwan[70]	F: 1,155	0-30 yrs			249 593 months		2791		
						Obstructive sleep apnoea (OSA)	31	0.0111071	0.64 per 10000 person yrs 95% CI 0.5-0.82)
						Allergic rhinitis	401	0.1436761	0.006907699
						Obesity	19	0.0068076	0.000327297
						Osteoporosis	7	0.0025081	0.000120583
						Disorders of mineral metabolism	3	0.0010749	5.16785E-05
						Hypocalcaemia/hypomagnesemia	0	0	0
Zhang-Jian, et al, 2020, Taiwan[71]	F: 549	Age at pregnancy is 24.4 ± 3.4 yrs					549		
						Pregnancy outcomes			
						Stillbirth	4	0.007286	
						Foetal distress	32	0.0582878	
						Foetal abnormalities, any	38	0.0692168	
						Preeclampsia	17	0.0309654	
						Gestational diabetes	24	0.0437158	
						Pregnancy-related hypertension	24	0.0437158	
						Antepartum haemorrhage	55	0.1001821	

						Severe postpartum haemorrhage	25	0.0455373		
						Chorioamnionitis	5	0.0091075		
						Caesarean delivery	155	0.2823315		
						Low birth weight <2500g	64	0.1165756		
						Prematurity <37 weeks	49	0.0892532		
Rypdal, et al, 2021, USA[72]	F: 297	≥19 yrs	Mean 5.7 IQR 2.6-9.8		17.6 yrs IQR (16.7-18.4)		434			
						Uveitis	96	0.2211982	12.56807708	
						At least 1 ocular complication	31/80	0.3875	22.01704545	
						Cataract	25/80	0.3125	17.75568182	
						Glaucoma	22/80	0.275	15.625	
						Synechiae	14/80	0.175	9.943181818	
						Macular oedema	8/80	0.1	5.681818182	
						Band keratopathy	7/80	0.0875	4.971590909	
						Epiretinal membrane	3/80	0.0375	2.130681818	
						Hypotony	3/80	0.0375	2.130681818	
						Phthisis	3/80	0.0375	2.130681818	
						Blindness <3/60 - binocular	2/80	0.025	1.420454545	
						Blindness <3/60 - monocular	3/80	0.0375	2.130681818	
**Childhood / jSLE**										
Davis, et al, 2023, USA[73]	F: 571	18.4±3.7 yrs (10-24 yrs)					650			White 367; Black 110; Hispanic 91; Asian 41; Unknown 41
						Depression	117	0.18		
						Anxiety	78	0.12		

							Adjustment disorder	65	0.1		
							Seizure/stroke disorder	52	0.08		
Garf, et al, 2015, Egypt[74]	F: 49	Mean 13.1±3.0 yrs			Mean 3.9±3.0 yrs (range 0.2-12 yrs)			70			
							Vitamin D deficiency	28	0.4		
							Vitamin D insufficiency (≤30/ng/ml)	42	0.6		
Paim-Marques, et al, 2019, Brazil[75]	F: 61	Mean 17.9±3.07 yrs	Mean 13.56 ± 2.60 yrs (corneal verticillata) 12.63 ± 2.9 (no corneal verticillata)			Mean 5.06 ± 2.04 yrs (with corneal verticillata)		76			
							Visual acuity reduction	25	0.3289474	65.00936135	
							Posterior subcapsular cataracts	4	0.0526316	10.40149782	
							Keratitis	40	0.5263158	104.0149782	
							Crystalline abnormalities	1	0.0131579	2.600374454	
							Corneal vortex keratopathy	16	0.2105263	41.60599126	
Silva, et al, 2015, Brazil[76]	F: 732	Median 14.5 (6–20) yrs, IFI; 17.0 (2–25.9) non-IFI	Median 12.4 (range 5.1–17.2) yrs, IFI; 11.8 (0.2–		Median 1.0 (range 0–7.3) yrs, IFI; 4.7 (range 0–23.4) non- IFI			852			White 592/830

			17.8) non IFI						
					Invasive Fungal Infections (Proven)	22 (split as follows)			
					Candidiasis	13	0.0152582		
					Aspergillosis	6	0.0070423		
					Cryptococcosis	2	0.0023474		
					Histoplasmosis	1	0.0011737		
					Arterial hypertension	119/843	0.1411625		
					Acute renal failure	37/841	0.0439952		
					Chronic renal failure	27/842	0.0320665		
					Renal replacement Therapy	28/842	0.0332542		
Knight, et al, 2016, USA[77]	F: 805	Mean 14.7 SD 2.0 (10-18 yrs)				970			White 141; African American 410; Latino 265; Other 154
					Depression diagnosis	181	0.1865979		
					Anxiety	72	0.0742268		
					Acute stress/adjustment	60	0.0618557		
					Other psychiatric disorder	175	0.1804124		
					Seizures/stroke	155	0.1597938		
Das Chagas Medeiros, et al, 2016, Brazil[78]	F: 52	Mean 15.5±2.0 yrs		Mean 10.4±6.4 yrs		60			
					Antiphospholipid syndrome	3/49	0.0612245	5.886970173	
					Hypothyroidism	1/37	0.027027	2.598752599	
					Thrombosis	7/60 (split below)			
					Deep vein thrombosis	1	0.0166667	1.602564103	
					Mesenteric artery thrombosis	1	0.0166667	1.602564103	
					CVA	1	0.0166667	1.602564103	
					Pulmonary embolus	1	0.0166667	1.602564103	
					Portal vein thrombosis	1	0.0166667	1.602564103	

						Renal vein thrombosis	1	0.0166667	1.602564103
						DVT + PE	1	0.0166667	1.602564103
						Neoplasia	1/58	0.0172414	1.657824934
						hypertension	27/60	0.45	43.26923077
						Cardiovascular diseases	3/58 (split below)		
						Congestive heart failure			
						Coronary artery disease			
						Aortic and mitral valve disorder			
						Diabetes	0/60	0	0
						Seizure	7/60	0.1166667	11.21794872
						Psychosis	2/60	0.0333333	3.205128205
Frittoli, et al, 2016, Brazil[79]	F: 38	Mean 14.5±2.84 yrs					41		
						Cognitive dysfunction	17	0.4146341	
						Anxiety symptoms	8	0.195122	
						Depression symptoms	1	0.0243902	
Holland , et al, 2018, USA[80]	F: 869	Mean 12.2±3.1 yrs		Mean 3.81±2.98 yrs	Mean 3.8 yrs		1048		
						Ocular			
						Cataracts	4.20%	4.008E-05	0.010546404
						Retinal change	1.70%	1.622E-05	0.004268783
						Neuropsychiatric			
						Cognitive impairment	8.40%	8.015E-05	0.021092808
						Seizures requiring therapy	3.90%	3.721E-05	0.00979309
						Cerebrovascular accident	2.9%/0.4%	0.0069179	1.820510245
						Cranial/peripheral neuropathy	2.40%	2.29E-05	0.006026517
						Transverse myelitis	0.40%	3.817E-06	0.001004419
						Renal			
						End-stage renal disease	1.30%	1.24E-05	0.003264363
						Pulmonary			

Pulmonary hypertension	0.40%	3.817E-06	0.001004419
Pleural fibrosis	1.30%	1.24E-05	0.003264363
Shrinking lung	0.80%	7.634E-06	0.002008839
Pulmonary fibrosis	0.90%	8.588E-06	0.002259944
Pulmonary infarction	0%	0	0
Cardiovascular			
Angina/artery bypass	0.10%	9.542E-07	0.000251105
Myocardial infarction	0.1 / 0.1	0.0009542	0.251104861
Cardiomyopathy	0.90%	8.588E-06	0.002259944
Valvular disease	0.90%	8.588E-06	0.002259944
Pericarditis/pericardiectomy	1.60%	1.527E-05	0.004017678
Peripheral vascular			
Claudication for 6 months	0.30%	2.863E-06	0.000753315
minor tissue loss	2.40%	2.29E-05	0.006026517
significant tissue loss	0.7/0.3%	0.0022265	0.585911343
Venous thrombus swelling, ulcer/stasis	2.50%	2.385E-05	0.006277622
Gastrointestinal			
Bowel infarction or resection	1.3 / 0.3%	0.0041349	1.088121066
Mesenteric insufficiency	0.10%	9.542E-07	0.000251105
Peritonitis	0.20%	1.908E-06	0.00050221
Stricture/upper GI tract surgery ever	0.40%	3.817E-06	0.001004419
Pancreatic insufficiency	0.50%	4.771E-06	0.001255524
Musculoskeletal			
Muscle atrophy/weakness	7.90%	7.538E-05	0.019837284
Deforming/erosive arthritis	4.70%	4.485E-05	0.011801928
Osteoporosis with fracture	2.60%	2.481E-05	0.006528726
Avascular necrosis	2.7 / 1.0	0.0025763	0.677983126
Osteomyelitis	0.40%	3.817E-06	0.001004419
Ruptured tendons	0.10%	9.542E-07	0.000251105
Skin			
Scarring chronic alopecia	9.10%	8.683E-05	0.022850542
Extensive scar/panniculum	1.70%	1.622E-05	0.004268783
Skin ulceration for 6 months	2.70%	2.576E-05	0.006779831
Premature gonadal failure	2%	1.908E-05	0.005022097

							Diabetes	0.90%	8.588E-06	0.002259944	
							Malignancy	0.20%	1.908E-06	0.00050221	
Gad, et al, 2013, Egypt[81]	F: 17	Mean 12.90±2.673 yrs (8-16 yrs)			Mean 4.40±1.875 yrs			20			
							Sensorineural hearing loss (SNHL)	5	0.25		
							Mild Conductive hearing loss (CHL)	2	0.1		
Jongvilaikasem and Rianthavorn, 2021, Thailand[82]	F: 87	Mean 11.3 ± 2.4 yrs				26 months		97			
							Short final height	22	0.2268041	104.6788263	
							Mild CKD	88	0.9072165	418.7153053	
							Advanced CKD	9	0.0927835	42.82315623	
AlAhmed, et al, 2020, USA[83]	F: 306	Mean 11.39±3.8 yrs		Mean 12.04±3.62 year (Age first seen by paediatric rheumatologist)				386			Hispanic 102; Non-Hispanic 283, White 216, African American 105, Asia 33, Other 13
							Cerebrovascular accident	2	0.0051813		
							Cranial nerve disease	1	0.0025907		
							Renal dialysis	3	0.007772		
							Arterial thrombosis	3	0.007772		
							Venous thrombosis	10	0.0259067		
							Avascular necrosis	5	0.0129534		
							Autoimmune thyroiditis	10	0.0259067		

			Coeliac disease	1	0.0025907	
			T1DM	1	0.0025907	
			Other autoimmune disorder	22	0.0569948	
			Pulmonary hypertension	0	0	
			Coronary heart disease	0	0	
			Renal transplant	0	0	
Karve, S. et al, 2012, USA[12]	0-15 yrs	18 months		71		
			Asthma	8	0.1126761	75.11737089
			Atopic dermatitis	2	0.028169	18.77934272
			Allergic rhinitis	6	0.084507	56.33802817
			General diabetes	1	0.0140845	9.389671362
			Rheumatoid arthritis	14	0.1971831	131.4553991
			Multiple sclerosis	1	0.0140845	9.389671362
			Thyroiditis	1	0.0140845	9.389671362
			Fracture in post-index period	4	0.056338	37.55868545
			Psoriasis	0	0	0

							Type 1 diabetes	0	0	0	
							Other CNS demyelinating disease	0	0	0	
							Vitiligo	0	0	0	
							Grave's disease	0	0	0	
Ye, Q., et al, 2021, China[84]	F: 82	Median age 169.02 months (range 31.00-220.53)	133.83, range (27.70–202.83) months		28.00, range (0–144.00) months			107			
							Cataracts	21	0.1962617		
							Steroid-induced osteonecrosis of femoral head	3	0.0280374		
							Osteoporosis	5	0.046729		
							Short stature	8	0.0747664		
							Obesity	7	0.0654206		
							Diabetes	6	0.0560748		
							HDL resistance	1	0.0093458		
							Upper respiratory tract infection	11	0.1028037		
							Pneumonia	13	0.1214953		
							EB virus infection	15	0.1401869		
							Progressive systemic sclerosis	1	0.0093458		
							Secondary Sjogren	6	0.0560748		
							Juvenile rheumatoid arthritis	1	0.0093458		
							Antiphospholipid syndrome	2	0.0186916		
Cann, et al, 2022, Australia[85]	F: 37			*12.5 yrs (mean) 13 yrs (median)*		Mean 4.3 yrs , IQR (2.5–6.1) yrs		42			Caucasian 16, Indigenous
							Cataract	2	0.047619	11.07419712	Australian

						retinal change	2	0.047619	11.07419712	11, Asian 10, Other 5
						CVA	3	0.0714286	16.61129568	
						Significant tissue loss	1	0.0238095	5.53709856	
						Venous thrombosis	1	0.0238095	5.53709856	
						Muscle weakness	1	0.0238095	5.53709856	
						AVN	1	0.0238095	5.53709856	
						Scarring alopecia	2	0.047619	11.07419712	
						Diabetes	1	0.0238095	5.53709856	
						Growth failure	3	0.0714286	16.61129568	
						Delayed puberty	1	0.0238095	5.53709856	
						Premature gonadal failure	0	0	0	
						Malignancy	0	0	0	
Medhat, et al, 2020, Egypt[86]	F: 86	Mean 12.7 ±2.6 yrs (range 6–16)	Mean 9.9±2.5, range (4–15) yrs	Median 24, IQR (12–48) months			106			
						Osteoporosis	6	0.0566038		
						Hypothyroidism	2	0.0188679		
						Diabetes mellitus	4	0.0377358		
						Hypertension	31	0.2924528		
						Hepatitis C virus infection	5	0.0471698		
						Chronic kidney disease	5	0.0471698		
						End-stage renal disease	1	0.009434		
						Avascular necrosis	1	0.009434		
						Sicca manifestations	2	0.0188679		
						Ischaemic heart disease	0	0		
						CVA	0	0		
						Malignancy	0	0		
Aggarwal, et al, 2018, India[87]	F: 250	≤ 18 yrs	Median 14 yrs range (1-17)		Median 3.5 yrs, Range (0.25-28 yrs) (248 patients); 25 patients were not followed up		273			
						Cushingoid habitus	35	0.1282051	36.63003663	

							Growth retardation	32	0.1172161	33.4903192	
							Avascular necrosis	9	0.032967	9.419152276	
							Cataract	8	0.029304	8.372579801	
							Osteopenia	10	0.03663	10.46572475	
							Infection	72	0.2637363	75.35321821	
							Hypertension	42	0.1538462	43.95604396	
							DVT	8	0.029304	8.372579801	
							Pulmonary thromboembolism	2	0.007326	2.09314495	
							Stroke	3	0.010989	3.139717425	
							Renal vein thrombosis	1	0.003663	1.046572475	
Al-Mayouf, et al, 2008, Saudi Arabia[88]	F: 76	F 14.4±3.9 yrs, male 15.8±4.4 yrs (mean)		Mean 9.3±2.8 yrs(F); 10±2.5 yrs (M)	F 66.5 ± 39.2 months, Male 76.2 ± 56.4 months, median 5 yrs	F 60.5 ± 38 months, Male 69.1 ± 57.5 months		89			
							Diabetes	1	0.011236	0.185718265	
							Malignancy	0	0	0	
							Dialysis + transplant (ESRD)	5	0.0561798	0.928591327	
							Premature gonadal failure	0	0	0	
							Transverse myelitis	2	0.0224719	0.371436531	
							Pyoderma gangrenosum	1	0.011236	0.185718265	
							Pancreatic pseudocyst	1	0.011236	0.185718265	
Costagliola, et al, 2018, Italy[89]	F: 24		Mean 14.6 ± 1.6 yrs			Mean 14.17 ± 8.04 yrs (1 patient excluded)		25			Caucasian 24, Hispanic 1
							Renal damage (including nephrotic syndrome or ESRD)	9	0.36	25.40578687	
							Vascular involvement (including DVT or ischaemic arterial lesions)	7	0.28	19.76005646	

							Nervous system involvement (with ischemic stroke, cranial neuropathies and cerebral vasculitis)	4	0.16	11.29146083	
							Cutaneous damage (chronic non-scarring alopecia)	3	0.12	8.468595625	
							Myositis	1	0.04	2.822865208	
							Acute myocardial infarction	1	0.04	2.822865208	
							Pulmonary (restrictive syndrome and pulmonary heart)	1	0.04	2.822865208	
							CMV reactivation	3	0.12	8.468595625	
							Herpes zoster reactivation	2	0.08	5.645730416	
							Severe EBV infection	1	0.04	2.822865208	
							Mycobacterium tuberculosis infection	1	0.04	2.822865208	
							ESRD	3	0.12	8.468595625	
							Requiring dialysis	1	0.04	2.822865208	
							Multi-infarct encephalopathy	1	0.04	2.822865208	
Torrente-Segarra, et al, 2017, Spain[90]	F: 435	Mean 31.5±10.5 Median 30 range [24-28] yrs Childhood		Mean 16.6 ± 6.3 yrs, median 16 [13-18]	Mean 10.7±7.2 yrs			484			Caucasian 441
							Articular				
							Erosive arthritis	40	0.0826446		
							Atrophy, muscle weakness	15	0.0309917		
							Tendon rupture	5	0.0103306		
							Osteoporosis	15	0.0309917		
							Fibromyalgia	11	0.0227273		
							Avascular necrosis	1.6 ± 0.6 2 [1-2]			
							Pulmonary embolism	11	0.0227273		
							Lung fibrosis	6	0.0123967		
							Pulmonary hypertension	8	0.0165289		
							Alveolar haemorrhage	6	0.0123967		
							Shrinking lung	4	0.0082645		
							Pleural fibrosis	1	0.0020661		

Pulmonary infarction	1	0.0020661
Vascular disease	25	0.0516529
Valvular dysfunction	19	0.0392562
Libman-Sachs Endocarditis	8	0.0165289
Cardiomyopathy	7	0.0144628
Angina or cardiac bypass	6	0.0123967
Myocarditis	3	0.0061983
Number of heart attacks	1.1 ± 0.0<br>1.0 [1-1]	
Small tissue loss	22	0.0454545
Major tissue loss	3	0.0061983
Venous thrombosis	3	0.0061983
Intermittent claudication >6 months	1	0.0020661
Renal terminal failure	27	0.0557851
Seizures (requiring therapy for 6 months)	46	0.0950413
Cognitive impairment/ Psychosis	21	0.0433884
Neuropathy	13	0.0268595
Transverse myelitis	3	0.0061983
Number of strokes	1.2±0.6<br>1[1-1]	
Cataract	31	0.0640496
Retinopathy	22	0.0454545
Amenorrhoea <40	22 (51.2%)	0.0454545
Dyslipidaemia	104	0.214876
High blood pressure	100	0.2066116
Autoimmune thyroiditis	31	0.0640496
Alcoholism	14	0.0289256
Congestive heart failure	8	0.0165289
Diabetes mellitus with organ involvement	8	0.0165289
Diabetes mellitus without organ involvement	0	0
Chronic obstructive pulmonary disease	7	0.0144628
Peripheral arterial disease	3	0.0061983
Malignancy Solid without metastasis	15	0.0309917

						Malignancy Solid with metastasis	0	0		
						Leukaemia	1	0.0020661		
						Lymphoma	2	0.0041322		
						Dialysis	23	0.0475207		
						Kidney transplantation	15	0.0309917		
						Splenectomy	14	0.0289256		
Ponin, et al, 2022, Thailand[91]	F: 87	Mean 20.6 ± 3.0 yrs	Mean 12.1 ± 2.3 yrs	Mean 5.5 ± 2.7 yrs			106			
						Growth impairment	25	0.2358491		
Avar Aydin, et al, 2018, USA[92]	F: 87		Mean yrs 13.2±3.1		Median 29.5 months (IQR 6.0–55.2)		110			Caucasian 54; African American 39; Asian 8; Other 9
						Hypertension	22	0.2	81.3559322	
						Obesity	19	0.1727273	70.26194145	
						Dyslipidaemia	14	0.1272727	51.77195686	
Ahluwalia, et al, 2014, India[93]	F: 17				Mean 82 months (range 9–210 months)		21 (27 patients initially, 6 lost to follow up)			
						Thrombosis	3 (split below)			
						Cerebral infarct (death)	1	0.047619	6.968641115	
						Pulmonary thromboembolism (death)	1	0.047619	6.968641115	
						Spinal infarct	1	0.047619	6.968641115	
Sinicato, et al, 2017, Brazil[94]	F: 37	16.7 ± 4.0 (mean)		Mean 4.8 ± 4.1 yrs			41			
						iglyceridemia	11	0.268		
						Insulin resistance	2	0.049		
						Arterial hypertension	4	0.097		
						Albumin Exretion	5	0.122		

				high LDL-c levels	19	0.463	
				hypercholesterolemia	8	0.195	
Cabral, et al, 2013, Portugal[95]	F: 46	Mean 12.6±4.0 4 yrs Median 13.5 range (1-17)	Mean 5.5±5.4 yrs Median 4 range (0-24)		56		
				Cataract	4	0.0714286	12.98701299
				Epilepsy	3	0.0535714	9.74025974
				Obesity	25	0.4464286	81.16883117
				Cardiac failure	2	0.0357143	6.493506494
				Arterial hypertension	5	0.0892857	16.23376623
				Avascular necrosis	1	0.0178571	3.246753247
				Growth delay	3	0.0535714	9.74025974
				Osteoporosis	5	0.0892857	16.23376623
				Gastrointestinal tract bleeding	1	0.0178571	3.246753247
				Macrophage activation syndrome	2	0.0357143	6.493506494
				Infection	6 (split below)		
				Pneumonia	2	0.0357143	6.493506494
				Sepsis	1	0.0178571	3.246753247
				Bacterial endocarditis	1	0.0178571	3.246753247
				Cellulitis	3	0.0535714	9.74025974
				Cognitive impairment	4	0.0714286	12.98701299
				End stage renal disease	2	0.0357143	6.493506494
				Vascular thrombosis	5 (split below)		
				Cerebrovascular (thrombosis)	4	0.0714286	12.98701299
				Cerebral hemorrhage	2	0.0357143	6.493506494
				Myocardium infarct	1	0.0178571	3.246753247
				Epilepsy	3	0.0535714	9.74025974

Bernatsky, et al, 2017, North America[96]	F: 978	Mean 13±3.3 yrs		Mean 7.6 yrs, SD 6.4 yrs, IQ 3.4-10.0 yrs/8839 PY		1168			
					Non-Hodgkin lymphoma	3	0.0025685	0.337959625	
					Head and neck squamous cell carcinomas (tongue and nasopharynx)	2	0.0017123	0.225306417	
					Glioblastoma	1	0.0008562	0.112653208	
					Thyroid cancer (papillary adenoma)	1	0.0008562	0.112653208	
					Breast cancer (infiltrating duct carcinoma)	1	0.0008562	0.112653208	
					Adenocarcinoma not otherwise specified	1	0.0008562	0.112653208	
					Cancers whose morphology and histology codes were indeterminate	3	0.0025685	0.337959625	
Yang, et al, 2015, Canada[97]	F: 30 (AVN), F: 82 (non-AVN)		Mean 13.8 ± 2.5 (AVN), 13.8 ± 2.4 (non-AVN)	Mean 6.89 ± 5.39 yrs (AVN), 5.72 ± 5.09 yrs (non-AVN)		617			AVN: South Asian 10; *Other Asian 9; Black 9; White 8; Other 1. Non-AVN: South Asian 10, Other Asian 30, Black 12, WhIte 40, Hispanic 1 Other 13, Unknown 5
					Symptomatic Avascular Necrosis (AVN)	37	0.0599676	8.703568228	

Lin, et al, 2018, Taiwan[98]			Mean 12.9±3.1 2 yrs (range 6-18 yrs)		mean 6 yrs		35		
						Vitamin D deficiency	28	0.8	133.3333333
Kahwage, et al, 2017, Brazil[99]	F: 6/7 (uveitis)	≤25 yrs, Median 10.4 range (8.8 -14.5) (Uveitis)					852		
						Uveitis	7	0.008216	
						Cataracts & bilateral irreversible blindness	1	0.0011737	
						Unilateral blindness	1	0.0011737	
Lai, et al, 2022, China[100]	F: 95	Age at enrolment - Girls mean 14.4 (SD 3.4), Boys mean 13.9 (SD 5.0)			Girls mean 6.6 (SD 4.1) Boys mean 5.2 (SD 4.0)		115		
						Hypertension	22	0.1913043	
						Chronic kidney disease	14	0.1217391	
Adulthood / jSLE									
Al Hamzi, et al, 2014, Saudi Arabia[101]	F: 45	Mean 23.6±4 yrs	Mean 10.3±6 yrs	Mean 15±4 yrs			48		
						Growth retardation/Short stature	24	0.5	
						Delayed puberty	13	0.2708333	
						Renal disease	12 (as split below)		
						Progressive renal disease and required dialysis	7	0.1458333	
						Renal transplant	5	0.1041667	
						Neuropsychiatric	9	0.1875	
						Cardiovascular	6	0.125	

					Musculoskeletal	9	0.1875		
					Skin	2	0.0416667		
					Seizure disorder	6	0.125		
					Chorea	3	0.0625		
					Cerebrovascular accident	3	0.0625		
					Miscarriage	4	0.0833333		
Tanigava, et al, 2022, Brazil[102]	F: 65	Median 21 yrs (IQR 20-26)		Median 14 IQR 12-16	Median 9 yrs IQR 6-14		69		White 35; Black 31; Asian 3
					Mental disorders	16	0.2318841		
					Obesity	14	0.2028986		
					Thyroid diseases	7	0.1014493		
					Fibromyalgia	6	0.0869565		

Antiphospholipid syndrome	56	0.8115942
Osteoporosis and osteoporotic fracture	5	0.0724638
Hypertension	5	0.0724638
Cerebrovascular disease	4	0.057971
Allergic disorders	4	0.057971
Gastrointestinal diseases	4	0.057971
Valvular disease	4	0.057971
Chronic kidney disease	3	0.0434783
Diabetes mellitus	2	0.0289855
Dyslipidaemia	2	0.0289855
Retinal change or optic atrophy	5	0.0724638
Cataract	2	0.0289855
Cognitive impairment or major psychosis	3	0.0434783
Seizures requiring therapy for ≥6 months	2	0.0289855
Cerebrovascular accident	3	0.0434783
Cranial or peripheral neuropathy	3	0.0434783
Transverse myelitis	0	0
End-stage renal disease	3	0.0434783
Pulmonary hypertension	0	0
Pulmonary fibrosis	2	0.0289855
Shrinking lung	0	0
Pleural fibrosis	0	0
Pulmonary infarction	0	0

						Angina or coronary artery bypass	0	0	
						Myocardial infarction	0	0	
						Cardiomyopathy	0	0	
						Pericarditis or pericardiectomy	0	0	
						Venous thrombosis	1	0.0144928	
						Infarction or resection of bowel	1	0.0144928	
						Mesenteric insufficiency	0	0	
						Chronic peritonitis	0	0	
						Stricture or upper gastrointestinal tract surgery	0	0	
						Pancreatic insufficiency	1	0.0144928	
						Muscle atrophy or weakness	0	0	
						Deforming or erosive arthritis	0	0	
						Osteoporosis with fracture or vertebral collapse	2	0.0289855	
						Avascular necrosis	2	0.0289855	
						Osteomyelitis	0	0	
						Ruptured tendon	0	0	
						Scarring chronic alopecia	0	0	
						Extensive scarring	1	0.0144928	
						Skin ulceration (excluding thrombosis)	4	0.057971	
						Premature gonadal failure	0	0	
						Diabetes requiring therapy	0	0	
						Malignancy	0	0	
Hersh, et al,2009, USA[103]	F: 78	Mean 30.5±12.8 yrs		14.0 +/-2.7 range (2–17)	Mean 16.5 yrs +/- 8.5 (range 3-47)		90		White 48.9%; Asian/Pacific Islander 16.7%; Hispanic/ Latino 15.6 %; African American 7.8%; Other 11%
						Pulmonary	23.60%	0.236	
						Clotting disorders	11.30%	0.113	
						Seizures	23.60%	0.236	
						Myocardial infarction	7.90%	0.079	
						Transplant	12.4	0.1377778	
						Dialysis	19.1	0.1377778	

Sousa, et al, 2016, Portugal[104]	F: 77	Mean 25.1 ± 9.1 yrs		13.4 ± 5.5				89			Caucasian 78, African 3, Others 4
							Hypertension	17	0.1910112		
							Diabetes	3	0.0337079		
							Hypercholesterolemia	2	0.0224719		
							Thyroid disease	3	0.0337079		
							Antiphospholipid syndrome	10	0.1123596		
							Sjögren's syndrome	2	0.0224719		
Koutsonikoli, et al, 2015, Greece[105]	F: 39	Mean 22±7.8 yrs at the end of follow up	Mean 12.5 ± 3.1 (SD)		Median 7.4 yrs and IQR (3.9–14.2) yrs	440 person-yrs (people*avg follow up time)		47			All Caucasians
							Cataract	4	0.0851064	0.193423598	
							Retinopathy	1	0.0212766	0.048355899	
							Optic atrophy	1	0.0212766	0.048355899	
							Cataract and retinopathy	1	0.0212766	0.048355899	
							Seizures	2	0.0425532	0.096711799	
							Cognitive impairment and seizures	1	0.0212766	0.048355899	
							Major psychosis and seizures	1	0.0212766	0.048355899	
							Cerebral vascular accident	1	0.0212766	0.048355899	
							Transverse myelitis	1	0.0212766	0.048355899	
							Single venous thrombosis	5	0.106383	0.241779497	
							Significant tissue loss and venous thrombosis	1	0.0212766	0.048355899	
							Avascular necrosis	3	0.0638298	0.145067698	
							Persistent impairment of renal function or end-stage renal disease	0	0	0	
							Pulmonary fibrosis	1	0.0212766	0.048355899	
							Pulmonary infarction	1	0.0212766	0.048355899	
							Premature gonadal failure	3	0.0638298	0.145067698	

						Delayed growth	6	0.1276596	0.290135397	
Gamal, et al, 2022, Egypt[106]	F: 163	25 yrs (median); range, 20 to 30.3 yrs	14 (median) (range 12-15)		12 yrs (median); (range, 6.75 to 17.25) yrs		186			
						Hypertension	82	0.4408602		
						Diabetes mellitus	12	0.0645161		
						Dyslipidaemia	75	0.4032258		
						Thyroid dysfunction	10	0.0537634		
						Malignancy	1	0.0053763		
						Osteoporosis	27/150	0.18		
						Avascular necrosis	23	0.1236559		
						End stage renal disease	17	0.0913978		
						Thrombosis	22	0.1182796		
						Raynaud's phenomenon	51	0.2741935		
						Pulmonary hypertension	24	0.1290323		
Groot, et al, 2019, Netherlands[4]	F: 101	Median 33 yrs (range 18-65)		14 (range 4-17)	Median 20 yrs range (1-55 yrs)		111			White 80, Non-White 31
						Muscle atrophy or weakness	1	0.009009		
						Deforming or erosive arthritis	10	0.0900901		
						Osteoporosis with fracture	6	0.0540541		
						Avascular necrosis	14	0.1261261		
						Osteomyelitis	1	0.009009		
						Ruptured tendon	1	0.009009		
						Cognitive impairment or psychosis	12	0.1081081		
						Seizures	10	0.0900901		
						CVA	7	0.0630631		
						Cranial or Peripheral Neuropathy	4	0.036036		
						Transverse Myelitis	0	0		
						ESRD	8	0.0720721		
						Claudication	0	0		
						Minor tissue loss	0	0		
						Significant tissue loss	4	0.036036		

					Venous thrombosis	8	0.0720721	
					Scarring alopecia	8	0.0720721	
					Scarring/ panniculum	0	0	
					Skin ulceration	3	0.027027	
					Cataracts	8	0.0720721	
					Retinal change	3	0.027027	
					Angina/bypass	4	0.036036	
					MI	5	0.045045	
					Cardiomyopathy	1	0.009009	
					Valvular disease	2	0.018018	
					Pericarditis	0	0	
					PAH	1	0.009009	
					Pulmonary fibrosis	4	0.036036	
					Shrinking lung	1	0.009009	
					Pleural Fibrosis	0	0	
					Pulmonary Infarction	0	0	
					Infarction or resection of bowel	4	0.036036	
					Mesenteric insufficiency	0	0	
					Chronic peritonitis	0	0	
					Stricture or upper GI surgery	0	0	
					Pancreatitis	0	0	
					Premature gonadal failure	2	0.018018	
					Malignancy	2	0.018018	
					IDDM	1	0.009009	
Sinicato, et al, 2017, Brazil[94]	F:32					35		
					Low HDL-cholesterol	5	0.143	
					Hypertriglyceridemia	6	0.171	
					Insulin Resistance	0	0.00	
					Arterial Hypertension	4	0.14	
					Albumin excretion	4	0.114	
**Childhood / JDM**								
Huber, et al, 2000, Canada[1]	F: 46	Median 13 (range 7-26)	Median 5.8 yrs Range (1-16)	Median 7.2 yrs range		65		Caucasian 56; Asian 7,

			(3.2 - 13.9 yrs)					African Canadian 1, Indian 1
				Calcinosis	22	0.3384615	47.00854701	
				Insulin-dependent diabetes mellitus	3	0.0461538	6.41025641	
				Connective tissue diseases	2 (split below)			
				Scleroderma	1	0.0153846	2.136752137	
				Overlap connective tissue disease	1	0.0153846	2.136752137	
				Malignancies	0	0		
Cho, et al, 2019, Korea[107]	F: 68	Mean 12.7 ± 7.1 yrs	10 yrs		130			
				Congestive heart failure	1	0.0076923	0.769230769	
				Cardiac arrhythmias	1	0.0076923	0.769230769	
				Valvular disease	1	0.0076923	0.769230769	
				Pulmonary circulation disorders	2	0.0153846	1.538461538	
				Peripheral vascular disorders	3	0.0230769	2.307692308	
				Hypertension, uncomplicated	5	0.0384615	3.846153846	
				Hypertension, complicated	2	0.0153846	1.538461538	
				Paralysis	3	0.0230769	2.307692308	
				Other neurological disorders	2	0.0153846	1.538461538	
				Chronic pulmonary disease	36	0.2769231	27.69230769	
				Diabetes, uncomplicated	8	0.0615385	6.153846154	
				Diabetes, complicated	2	0.0153846	1.538461538	
				Renal failure	1	0.0076923	0.769230769	
				Liver disease	15	0.1153846	11.53846154	
				Peptic ulcer disease excluding bleeding	15	0.1153846	11.53846154	
				AIDS/HIV	1	0.0076923	0.769230769	
				Solid tumour without metastasis	1	0.0076923	0.769230769	
				Coagulopathy	4	0.0307692	3.076923077	
				Weight loss	1	0.0076923	0.769230769	
				Fluid and electrolyte disorders	8	0.0615385	6.153846154	
				Deficiency anaemia	17	0.1307692	13.07692308	
				Depression	6	0.0461538	4.615384615	
				Pulmonary disease specified				

							Interstitial lung disease	2	0.0153846	1.538461538	
Concannon and Han, 2021, New Zealand[108]	F: 18	≤15 yrs		Mean 8.6 yrs SD 3.7 yrs		Mean 6.5, range (0.4–16.7) yrs		31			Asian 5. Maori 7, pacific island 3, European 16
							Calcinosis	9	0.2903226	44.66501241	
							Cutaneous vasculopathy	5	0.1612903	24.81389578	
							Interstitial lung disease	6	0.1935484	29.77667494	
							Pneumomediastinum	2	0.0645161	9.925558313	
							Pneumothorax	3	0.0967742	14.88833747	
							Surgical emphysema	1	0.0322581	4.962779156	
Okong'o, et al, 2016, South Africa[109]	F: 16		Median 6.75, range (2.0–9.7) yrs	Median 7.9 , range (3.4 – 9.75) year	median 4, range (0.5-84) months	Minimum of 12 months, median 55.5 months (inactive disease) 49.1 months (active disease)		25			Indigenous African 13, mixed race 11, European ancestry 1
							Skin				
							Calcinosis	8	0.32		
							Skin ulcers	1	0.04		
							Oedema (Generalized, Periorbital or limb)	0	0		
							Alopecia	3	0.12		
							Lipodystrophy	1	0.04		
							Musculoskeletal:		0		
							Arthritis	2	0.08		
							Contractures	5	0.2		
							Osteoporosis with fracture	1	0.04		
							Endocrine:		0		
							Growth failure	8	0.32		
							Diabetes mellitus	1	0.04		
							Adrenal insufficiency	0	0		

					Respiratory:		0	
					Interstitial lung disease (ILD)	2	0.08	
					Pulmonary haemorrhage	1	0.04	
					Gastrointestinal:		0	
					Abdominal pain or bleeding	0	0	
					Ocular:		0	
					Cataracts	2	0.08	
					Nervous system:		0	
					Sensorineural hearing loss	1	0.04	
					Seizures, neuropathy		0	
					Cardiovascular		0	
					Raynaud's phenomenon	6	0.24	
					Cardiomyopathy	0	0	
					Infections:		0	
					Fungal (skin)	7	0.28	
					Bacterial (staph aureus 2, gram negative 1)	3	0.12	
					TB	2	0.08	
Ravelli, A. et al, 2010, Italy, UK[2]	F: 321		Mean 6.9, range (0.9–17.8) yrs	Mean 7.7, range (2–25.2) yrs		490		White 320
					Cutaneous scarring/atrophy	195/443	0.4401806	
					Calcinosis	106/406	0.2610837	
					Joint contractures	82/448	0.1830357	
					Lipodystrophy	43/444	0.0968468	
					Hirsutism	44/431	0.1020882	
					Growth failure	34/424	0.0801887	
					Osteoporosis with fractures	26/435	0.0597701	
					Cataract	9/430	0.0209302	
					Gastrointestinal infarction/resection	7/444	0.0157658	
					Malignancy	0/447	0	

Tabarki, et al, 1998, France[110]	F: 22	Mean 6.9 yrs (range 2-14 yrs)		Mean 4.9 yrs (range: 2.5-13 yrs)		36			
					Calcinosis	15	0.4166667	85.03401361	
					Impaired function	8	0.2222222	45.35147392	
					Cataracts	1	0.0277778	5.66893424	
					Vertebral collapse	2	0.0555556	11.33786848	
					Contractures	1	0.0277778	5.66893424	
Chevalier, et al, 2021, France[111]	F: 4	Median 6 yrs range 2-11 yrs	Median 5.5 months	Mean 14.9±8.8 yrs		7			Caucasian 100%
					Polycystic ovary syndrome	1	0.1428571	9.587727709	
					Depressive disorder	1	0.1428571	9.587727709	
					mild physical dysfunction (CHAQ/HAQ >0)	2	0.2857143	19.17545542	
					Calcinosis	2	0.2857143	19.17545542	
					Lipodystrophy	2	0.2857143	19.17545542	
					Osteopenia/osteoporosis	0	0	0	
					Cardiovascular damage	0	0	0	
					Peripheral vascular damage	0	0	0	
					Ocular damage	0	0	0	
					Malignancy	0	0	0	
					Fracture	0	0	0	
					Moderate or severe disability CHAQ/HAQ>1	0	0	0	
Al-Mayouf, et al, 2017, Saudi Arabia[112]	F: 58	Mean 6 ± 3 yrs		Mean 5 ± 4.4 yrs		92			

					Permanent skin changes	16	0.173913	34.7826087	
					Residual muscle weakness	12	0.1304348	26.08695652	
					Calcinosis	27	0.2934783	58.69565217	
					Growth failure	37	0.4021739	80.43478261	
					Osteoporosis	15	0.1630435	32.60869565	
					Lipodystrophy	3	0.0326087	6.52173913	
					Mucinous cystic ovarian tumour	1	0.0108696	2.173913043	
Sag, et al, 2021, Turkey[113]	F: 35	Mean 8.1±4.3 yrs	Mean 8.7±4.4	Median 4.85 yrs IQR 3.7, Mean 5.66±3.59 yrs		58			
					Calcinosis	19	0.3275862	67.54354781	
					Pulmonary involvement	8	0.137931	28.43938855	
					Skin ulcers	4	0.0689655	14.21969428	
					Gastrointestinal involvement	4	0.0689655	14.21969428	
					Severe muscle disease (wheel-chair dependency)	3	0.0517241	10.66477071	
					Cardiac involvement	2	0.0344828	7.109847138	
Ramanan, et al, 2005, Canada[114]									
	F: 20		Mean 8.4 yrs	Mean 34.6 months	JDM patients after June 1997	31			Black 2; East Indian 4; Native Canadian 1; Asian 1; White 23
					Calcinosis	2	0.0645161	22.37553608	
					Cataracts	3	0.0967742	33.56330412	
	F:16		6.7 yrs (mean)	4 yrs (1056 patient months)	JDM patients pre-June 1997	22			Black 1; East Indian 1; Native Canadian 1; Asian 2; White 17

						Calcinosis	5	0.2272727	56.81818182	
						Cataracts	8	0.3636364	90.90909091	
Sun, et al, 2015, Taiwan[115]	F: 26		Mean 81.97 ± 46.63 months (25-216)	Mean 87.97± 45.90 months (28-213)	For those receiving IVIG: Mean 79 months		39			
						Calcinosis	15	0.3846154		
						Pulmonary involvement	4	0.1025641	Follow up time and cases were not reported coherently. Hence, no incidence rate was calculated	
						Malignancy	0	0		
						Disability	4	0.1025641		
						GI perforation	1	0.025641		
						Avascular necrosis of the hip joint	1	0.025641		
Silverberg, et al, 2018, USA[116]	F: 915	0-17					1407			White 543; Black 203; Hispanic 260; Asian 19; Multiracial/other 71
						Obesity	38	0.0270078		
						Hypertension	119	0.0845771		
						Diabetes, uncomplicated	18	0.0127932		
						Late effects of cerebrovascular disease	≤10	≤10/1407		
						Pulmonary circulatory disorder	15	0.010661		
						Valvular heart disease	17	0.0120824		
						Peripheral and visceral atherosclerosis	≤10	≤10/1407		
						Any lipid abnormalities	≤10	≤10/1407		
						Lipodystrophy	≤10	≤10/1407		
						Personal history of TIA and cerebral infarction without residual deficits	≤10	≤10/1407		
						Arrythmia	47	0.0334044		

							Valvular heart disease	17	0.0120824		
Adulthood / JDM											
Boros, et al, 2022, UK[117]	F:68	Mean 20.6 yrs ±3.9 (SD)	Mean 9.2 yrs ± 4.3 (SD)		Mean 12.4 yrs ± 5.0			84			White 69
							Feel shorter than peers	20 /80	0.25		
							Feel weight was affected by myositis	25/80	0.3125		
							Puberty affected by myositis	16/80	0.2		
							Calcinosis	18/82	0.225		
							Lipodystrophy	6/82	0.0731707		
							Pulmonary hypertension	2/83	0.0240964		
Mathiesen, et al, 2014, Denmark[118]	F: 37	Mean 21.7 yrs (SD 11.1) (Range 4.2-45.9) (Non-restrictive lung); 14.2 (4.4) (10.0-19.7) (restrictive lung)	Mean 7.7 yrs (SD 4.7) (Range 1.4-17.8) (non-restrictive lung); 7.7 (4.2) (1.5-10.5) (restrictive lung)		Mean 4.2 yrs (SD 2.9) (Range 0.610.6) (non-restrictive lung); 5.3 (2.5) (2.6- 8.5) (restrictive lung)	Mean 14.3 yrs (SD 10.7) (Range 1.1-35.1) (non-restrictive lung); 6.5 (3.0) (2.6-9.2) (restrictive lung)		51			
							Restrictive interstitial lung disease	4	0.0784314	12.06636501	
							Obstructive lung function pattern	7	0.1372549	9.598244892	
Tsaltskan, et al, 2020, USA[5]	F: 39	Median 24.0 yrs [IQR 19.0-28.6] yrs		Median 11.0 [IQR 8.5-15.7]	median 11.5 yrs, [IQR 4.5-18.9] yrs			49			Caucasian 59.2%, African American 11.1% , Hispanic 17.7%, Other 12.0%
							Cutaneous Damage:				
							Cutaneous scarring or atrophy	28	0.5714286		
							Calcinosis	27	0.5510204		
							Lipodystrophy	16	0.3265306		
							Alopecia	8	0.1632653		
							Poikiloderma	6	0.122449		
							Skeletal Damage:				

					Joint Contracture	31	0.6326531	
					Deforming Arthropathy	8	0.1632653	
					Osteoporosis with fracture	4	0.0816327	
					Avascular Necrosis	2	0.0408163	
					Endocrine Damage:			
					Irregular Menses	10/40	0.25	
					Hirsutism	7/40	0.175	
					Diabetes	6	0.122449	
					Amenorrhea	4/40	0.1	
					Infertility	1	0.0204082	
					Pulmonary Damage:			
					Impaired lung function	7	0.1428571	
					Dysphonia	4	0.0816327	
					Pulmonary Fibrosis	1	0.0204082	
					Ocular Damage:			
					Cataract	4	0.0816327	
					Vision Loss	3	0.0612245	
					Gastrointestinal Damage:			
					Dysmotility	5	0.1020408	
					Dysphagia	3	0.0612245	
					Infection:			
					Chronic Infection	5	0.1020408	
					Multiple Infections	4	0.0816327	
					Cardiovascular Damage:			
					Hypertension	4	0.0816327	
					Peripheral Vascular Damage:			
					Tissue or pulp loss	4	0.0816327	
					Digit or limb loss	1	0.0204082	
					Malignancy	1	0.0204082	
Mathiesen, et al, 2012, Denmark[119]	F: 38	Mean 21.5 yrs SD 11.1 Range 4.2-45.9)	Mean 4.3 yrs SD 2.9 range (0.6-10.6)	Mean 13.9 SD 10.5 (range 2.1–35.1) yrs		53		
					Deforming arthropathy	1	0.0188679	1.357404642
					Pulmonary fibrosis	3	0.0566038	4.072213927
					Growth failure	6/49	0.122449	8.809279107

							Diabetes	1	0.0188679	1.357404642	
							Scarring or atrophy	21	0.3962264	28.50549749	
							Calcinosis	11	0.2075472	14.93145107	
							Lipodystrophy	7	0.1320755	9.501832496	
							Tissue or pulp loss	1	0.0188679	1.357404642	
							Alopecia, poikiloderma	0	0	0	
							Joint contractures	11	0.2075472	14.93145107	
							Osteoporosis/osteopenia estimated by DXA	5	0.0943396	6.787023212	
							Osteoporotic fractures	2	0.0377358	2.714809285	
							Delay in secondary sexual characteristics*	3/14	0.2142857	15.41623844	
							Hypertriglyceridemia	9	0.1698113	12.21664178	
							Hirsutism, irregular menses, amenorrhoea	0	0	0	
							Paraesthesia	2	0.0377358	2.714809285	
							Arthritis	3	0.0566038	4.072213927	
							Impaired pulmonary function test	4	0.0754717	5.429618569	
							Pulmonary hypertension	0	0	0	
							Severe abdominal pain	3	0.0566038	4.072213927	
							Dysphagia, gastrointestinal dysfunction	1	0.0188679	1.357404642	
							Gastrointestinal surgery	1	0.0188679	1.357404642	
							Hypertension, cardiomyopathy, angina, myocardial infarction	0	0	0	
							Tissue or pulp loss, claudication	0	0	0	
							Cataract, visual loss	0	0	0	
							Chronic infection, multiple infections	0	0	0	
							Malignancy	0	0	0	
Sanner, et al, 2010, Norway[120]	F: 36	Median 21.5 (range 6.7–55.4) yrs	Mean 8.3 ±4.5 yrs	Mean 9.1 ± 4.5 yrs	minimum 24 months	Mean 16.9 ± 10.6		59			European 57
							Calcinosis	14	0.2372881	14.04071808	

Supplementary Table S2: Studies comparing comorbidity incidence between cases and controls of JIA/jSLE patients reported during childhood and adulthood. NR=p value not reported, NS=P value described as NS.

Study	Measure of effect	Control group	Comorbidity	Effect size	95% CI	p-value
**Childhood/ JIA**						
Schenck, et al, 2018, Germany[33]	Prevalence ratio	Diabetes registry data	Type 1 diabetes	1.76	(1.34-2.28)	<0.001
Krause, et al, 2017, USA[37]	Rate Ratio	Non-JIA comparator subjects. Age-sex matched	Joint surgeries	3.93	(1.18-24.94)	NR
			Non-joint surgeries	1.9	(1.05-3.67)	NR
			Hospitalizations	2.25	(1.04-5.53)	NR
			Hospitalizations for depression	1.41	(0.47-4.70)	NR
Lee, et al, 2020, USA[38]	Adjusted Hazard ratios	Healthy children with at least 1 Wellcare visit	Type 1 diabetes	1.81	(1.03–3.17)	NR
			Type 2 diabetes	1.22	(0.79–1.90)	NR
			Composite diabetes	1.4	(0.94–2.09)	NR

	Age, sex, index date-matched Hazard ratios	Healthy children with at least 1 Wellcare visit	Type 1 diabetes	1.89	(1.11-3.19)	NR
			Type 2 diabetes	1.33	(0.88–2.02)	NR
			Composite diabetes	1.52	(1.05–2.21)	NR
	Age, sex, index date-matched Hazard ratios	Children with asthma	Type 1 diabetes	1.46		NR
			Type 2 diabetes	0.95	(0.64–1.43)	NR
			Composite diabetes	1.11	(0.77–1.60)	NR
	Adjusted Hazard ratios	Children with asthma	Type 1 diabetes	1.48	(0.86–2.56)	NR
			Type 2 diabetes	1.01	(0.66–1.54)	NR
			Composite diabetes	1.18	(0.80–1.73)	NR
Beukelman, et al, 2012, USA[28]	Incidence rate ratio	Children with ADHD	Coccidioides	101	(8.1–5319)	NR
			Salmonella	3.8	(1.20–9.50)	NR
			Herpes zoster	2.1	(1.40–3.00)	NR
			All infections	2.4	(1.70-3.30)	NR
			All infections except Herpes zoster	4.2	(2.10-7.60)	NR
Berthold, et al, 2022, Sweden[53]	Hazard ratios	Non-JIA comparator subjects	**Females**			
		Sex, year of birth and residential region matched	Depression	1.1	(0.90–1.50)	NS
			Anxiety	1.2	(0.90–1.50)	NS
			**Males**			
			Depression	0.8	(0.50–1.40)	NS
			Anxiety	0.6	(0.40–1.10)	NS
Lovell, et al, 2021, USA[47]	Log risk ratio	General paediatric patients without JIA	Addison's disease	1.65	(1.02-2.21)	P<0.05
			Autoimmune/Hashimoto's thyroiditis	2.25	(1.72 to 2.71)	P<0.05
			Graves' disease	0.38	(–2.77-2.26)	NS
			Morphoea	3.15	(2.54-3.70)	P<0.05
			Multiple sclerosis	1.9	(–1.27-3.83)	NS
			Takayasu's arteritis	1.45	(–1.71-3.35)	NS
			Type 1 diabetes	1.25	(0.75-1.70)	P<0.05
			Vitiligo	1.76	(0.74-2.59)	P<0.05

			Autoimmune disease not elsewhere classified	2.55	(1.78-3.21)	P<0.05
			Chronic glomerulonephritis	0.73	(–2.45-2.64)	NS
			Chronic urticaria	0.58	(0.28-0.84)	P<0.05
			Episcleritis/scleritis	3.18	(2.14-4.03)	P<0.05
			Erythema nodosum	1.12	(–2.03-3.02)	NS
			Haemolytic anaemia	–0.36	(–3.49-1.54)	NS
			Immune thrombocytopenia purpura	0.4	(–1.42-1.68)	NS
			Leukocytoclastic vasculitis	4.08	(2.19-5.49)	P<0.05
			Pulmonary fibrosis/interstitial lung disease	2.46	(2.28-2.62)	P<0.05
			Raynaud's syndrome	3.37	(2.80-3.86)	P<0.05
			Sjögren's syndrome/sicca syndrome	3.27	(2.40-5.01)	P<0.05
			Thrombocytopenia purpura	0.57	(–2.61-2.46)	NS
			Vasculitis	3.71	(2.90-4.37)	P<0.05
Simon, TA et al, 2020, USA[41]	Odd Ratio (OR)	Patients with ADHD<br>1:5 matched for age (± 2 years), sex, number of medical encounters (in-patient, out-patient, and emergency room visits), and calendar year of diagnosis (± 1 year)	**MarketScan**			
			Addison disease	6.16	(2.83-13.42)	NR
			Alopecia areata	2.03	(0.93-4.46)	NR
			Celiac disease	3.99	(2.66-5.98)	NR
			Chronic glomerulonephritis	Infinite		NR
			Chronic urticaria	3.4	(2.74-4.22)	NR
			Crohn's disease	16.84	(10.91-25.99)	NR
			Erythema nodosum	12.05	(3.20-45.4)	NR
			Giant cell arteritis	4.51	(0.28-72.18	NR
			Grave's disease	3.23	(1.43-7.27)	NR
			Hashimoto's thyroiditis	3.57	(2.48-5.14)	NR
			Haemolytic anaemia	6.9	(3.98-11.97)	NR
			Immune thrombocytopenia	4.52	(1.88-10.86	NR
			Interstitial lung disease	24.86	(5.51-112.15)	NR
			Morphoea	21.36	(9.45-48.29)	NR
			Multiple sclerosis	2.26	(0.21-24.90)	NR
			Myasthenia gravis	4.51	(0.64-32.06)	NR
			Pernicious anaemia	0		NR

	Polymyalgia rheumatica	Infinite		NR
	Polymyositis	Infinite		NR
	Primary biliary cirrhosis	0		NR
	Psoriasis	8.42	(5.54-12.78)	NR
	Raynaud's syndrome	5.7	(3.44-9.46)	NR
	Scleritis and episcleritis	17.19	(6.42-46.04)	NR
	Sjogren's syndrome/sicca syndrome	Infinite		NR
	Systemic sclerosis	24.89	(8.57-72.23)	NR
	Type 1 diabetes	1.7	(1.27-2.27)	NR
	Ulcerative colitis	8.34	4.77-14.59)	NR
	Uveitis	165.1	(108.97-250.14)	NR
	Vasculitis	22.64	(9.42-54.42)	NR
	Vitiligo	2.85	(1.39-5.88)	NR
Patients with ADHD	**Pharmetrics Data**			
	Addison disease	2.51	(1.16-5.43)	NR
	Alopecia areata	2.43	(1.274.65)	NR
	Celiac disease	3.82	(2.61-5.60)	NR
	Chronic glomerulonephritis	31.57	(3.88-256.56)	NR
	Chronic urticaria	2.04	(1.65-2.51)	NR
	Crohn's disease	12.99	(8.78-19.2)	NR
	Erythema nodosum	9.02	(2.72-29.96)	NR
	Giant cell arteritis	Infinite		NR
	Grave's disease	1.69	(0.79-3.64)	NR
	Hashimoto's thyroiditis	4.26	(3.03-6.01)	NR
	Haemolytic anaemia	4.52	(2.50-8.16)	NR
	Immune thrombocytopenia	4.89	(2.23-10.72)	NR
	Interstitial lung disease	12.03	(3.19-45.33)	NR
	Morphoea	18.97	(7.15-50.33)	NR
	Multiple sclerosis	3.38	(0.76-15.11)	NR
	Myasthenia gravis	Infinite		NR
	Pernicious anaemia	0		NR

			Polymyalgia rheumatica	9.02	(0.82-99.43)	NR
			Polymyositis	22.55	(2.63-193.01)	NR
			Primary biliary cirrhosis	Infinite		NR
			Psoriasis	5.89	(4.07-8.53)	NR
			Raynaud's syndrome	12.16	(7.45-19.84	NR
			Scleritis and episcleritis	13.55	(5.76-31.88)	NR
			Sjogren's syndrome/sicca syndrome	72.24	(9.58-544.36)	NR
			Systemic sclerosis	90.34	(12.12-673.22)	NR
			Type 1 diabetes	1.82	(1.37-2.41)	NR
			Ulcerative colitis	6.49	(3.81-11.05)	NR
			Uveitis	133.23	(92.47-191.95)	NR
			Vasculitis	10.32	(4.24-25.08)	NR
			Vitiligo	2.61	(1.24-5.49)	NR
Horneff, et al, 2016, Germany[46]	Risk ratios	German Childhood Cancer Registry report 2015				
			**Methotrexate Drug Cohort**			
			All malignancy	3	(0.75-12.0)	0.12
			Lymphoma & Leukaemia	6.7	(1.67-26.82)	0.007
			**Etanercept Drug Cohort**			
			All malignancy	6.3	(3.00-13.20)	<0.0001
			Lymphoma & Leukaemia	8	(2.99-21.38)	<0.0001
**Adulthood / JIA**						
Raab, et al, 2013, Germany[59]	% difference (absolute)	Community sample in Germany	Cardiovascular diseases (as split below)	1.60		0.343
		Age and gender matched by propensity scores	Hypertension	-0.60		0.759
			Cardiac insufficiency	-0.90		0.111
			Asthma	-2.50		0.098
			Gastrointestinal diseases	-6.40		<0.001
			Gastrointestinal ulcer	-1.00		0.065
			Liver and gallbladder diseases	0.10		0.125
			Renal insufficiency	-1.00		0.064

			Diabetes mellitus	-0.70		0.332
			Depression	-4.10		0.004
Aggarwal, et al, 2006, India[60]	% difference (absolute)	Healthy male hospital employees	Osteopenia (Lumbar Spine)	-5.50		NS
			Osteopenia (Hip)	28.30		< 0.05
			Osteopenia (Distal third of forearm)	10.90		< 0.001
			Osteoporosis (lumbar spine)	22.40		NS
			Osteoporosis (hip)	5.90		< 0.05
			Osteoporosis (Distal third of forearm)	35.70		< 0.001
Smith, et al, 2019, USA[67]	Unadjusted Risk ratio	Women without autoimmune disease who delivered live born infants	Pre-eclampsia	2.48	(1.30–4.74)	P<0.05
			Pregnancy induced hypertension without pre-eclampsia	1.75	(0.83–3.69)	NR
			Placenta previa and/or abruption	1.46	(0.46–4.68)	NR
			Preterm labour	2.03	(1.27–3.23)	P<0.05
			Gestational diabetes	1.66	(0.87–3.16)	NR
			Fever during pregnancy	1.19	(0.89–1.58)	NR
			Caesarean section	1.55	(1.23–1.94)	P<0.05
	Adjusted risk ratio	Adjusted risk ratio computed using Poisson regression	Pre-eclampsia	1.87	(0.71–4.96)	NR
			Pregnancy induced hypertension without preeclampsia	0.60		(0.08–4.78)
			Gestational diabetes	0.91	(0.25–3.38)	NR
			Fever during pregnancy	2.61	(1.21–5.63)	P<0.05
	Adjusted risk ratio	Multivariable adjustment for the prior factors	Pre-eclampsia	2.19	(0.82–5.88)	NR
			Gestational diabetes	0.86	(0.24–2.97)	NR
			Fever during pregnancy	3.06	(1.42–6.56)	P<0.05
Simon, et al, 2020, USA[41]	Odds ratios	MarketScan patients with ADHD<br>1:5 matched age (± 2 years), sex, number of medical encounters (in-patient, out-patient, and emergency room visits), and calendar year of diagnosis (± 1 year).				
			Addison disease	4.87	(3.09-7.66)	NR
			Alopecia areata	1.09	(0.45-2.64)	NR
			Coeliac disease	1.32	(0.73-2.39)	NR
			Chronic glomerulonephritis	2.36	(0.59-9.43)	NR

	Chronic urticaria	2.53	1.94-3.30)	NR
	Crohn's disease	3.88	(2.93-5.16)	NR
	Erythema nodosum	0.94	(0.21-4.30)	NR
	Giant cell arteritis	Infinite		NR
	Grave's disease	2.15	(1.26-3.65)	NR
	Hashimoto's thyroiditis	1.56	(1.09-2.25)	NR
	Haemolytic anaemia	6.86	(3.88-12.14)	NR
	Immune thrombocytopenia	3.97	(2.35-6.69)	NR
	Interstitial lung disease	11.54	(6.94-19.2)	NR
	Morphoea	3.63	(1.59-8.28)	NR
	Multiple sclerosis	1.50	(0.96-2.33)	NR
	Myasthenia gravis	2.36	(0.59-9.43)	NR
	Pernicious anaemia	1.30	(0.75-2.27)	NR
	Polymyalgia rheumatica	8.20	(4.34-15.5)	NR
	Polymyositis	7.55	(2.47-23.09)	NR
	Primary biliary cirrhosis	2.83	(0.68-11.84)	NR
	Psoriasis	2.31	(1.73-3.08)	NR
	Raynaud's syndrome	3.24	(2.33-4.50)	NR
	Scleritis and episcleritis	5.25	(2.78-9.94)	NR
	Sjogren's syndrome/sicca syndrome	6.82	(4.76-9.77)	NR
	Systemic sclerosis	10.80	(4.44-26.26)	NR
	Type 1 diabetes	2.50	(1.98-3.14)	NR
	Ulcerative colitis	3.69	(2.69-5.06)	NR
	Uveitis	18.46	(13.8-24.69)	NR
	Vasculitis	12.13	(7.03-20.94)	NR
PharMetrics patients with ADHD	Addison disease	4.67	(2.83-7.73)	NR
	Alopecia areata	1.66	(0.81-3.41)	NR
	Coeliac disease	2.63	(1.68-4.11)	NR
	Chronic glomerulonephritis	24.06	(2.81-205.96)	NR
	Chronic urticaria	2.03	(1.55-2.66)	NR
	Crohn's disease	3.23	(2.36-4.42)	NR
	Erythema nodosum	7.70	(2.52-23.55)	NR
	Giant cell arteritis	3.21	(0.54-19.19)	NR
	Grave's disease	1.45	(0.83-2.54)	NR

			Hashimoto's thyroiditis	1.99	(1.42-2.80)	NR
			Haemolytic anaemia	5.46	(2.73-10.94)	NR
			Immune thrombocytopenic	4.82	(2.51-9.27)	NR
			Interstitial lung disease	12.89	(6.63-25.04)	NR
			Morphoea	4.81	(1.69-13.73)	NR
			Multiple sclerosis	1.52	(0.89-2.58)	NR
			Myasthenia gravis	3.85	(1.03-14.34)	NR
			Pernicious anaemia	1.24	(0.70-2.20)	NR
			Polymyalgia rheumatica	3.78	(1.72-8.34)	NR
			Polymyositis	4.01	(1.22-13.14)	NR
			Primary biliary cirrhosis	2.40	(0.22-26.52)	NR
			Psoriasis	2.93	(2.22-3.86)	NR
			Raynaud's syndrome	2.53	(1.76-3.65)	NR
			Scleritis and episcleritis	3.66	(2.02-6.66)	NR
			Sjogren's syndrome/sicca syndrome	6.86	(4.70-10.02)	NR
			Systemic sclerosis	8.83	(3.26-23.88)	NR
			Type 1 diabetes	2.15	(1.66-2.78)	NR
			Ulcerative colitis	2.41	(1.63-3.57)	NR
			Uveitis	29.54	(21.51-40.58)	NR
			Vasculitis	7.63	(4.29-13.56)	NR
			Vitiligo	2.16	(1.12-4.15)	NR
Ma, et al, 2022, Taiwan[70]	Relative risk	Individuals without JIA	Obstructive sleep apnoea	1.936	(1.26-2.97)	NR
	Adjusted hazard ratios	Adjusted for demographic variables including age, sex, length of hospital stays, and comorbidities at baseline.	Obstructive sleep apnoea	1.922	(1.24–2.97)	NR
	Adjusted hazard ratios	Adjusted for demographic variables including age, sex, length of hospital stays, and comorbidities at baseline and allergic rhinitis	Obstructive sleep apnoea	1.363	(0.70-2.65)	NS
Zhang-Jian, et al, 2020, Taiwan[71]	Adjusted odds ratio (for infant sex, Charlson comorbidity index, urbanization, income,	Non-JIA patients<br>1:5 matched maternal age and birth year<br>To avoid possible confounding factors and for case control selection, only the first child from either group was included for further analysis.	Stillbirth	0.92	(0.32–2.69)	NR
			Foetal distress	0.96	(0.65–1.41)	NR
			Foetal abnormalities, any	0.91	(0.64–1.29)	NR
			Pre-eclampsia	1.42	(0.82–2.46)	NR
			Gestational diabetes	0.75	(0.48–1.16)	NR

	occupation, maternal nationality)		Pregnancy-related hypertension	1.24	(0.79–1.96)	NR
			Antepartum haemorrhage	1.09	(0.81–1.47)	NR
			Severe postpartum haemorrhage	1.20	(0.77–1.87)	NR
			Chorioamnionitis	1.34	(0.49–3.66)	NR
			Caesarean delivery	1.07	(0.89–1.27)	NR
			Low birth weight <2500g	1.35	(1.02–1.79)	<0.05
			Prematurity <37 weeks	1.19	(0.87–1.64)	NR
Krause, et al, 2017, USA[37]	Rate ratio	Non-JIA comparator	Joint surgeries	8.5	(2.27-120.10)	NR
		Age and sex matched	Non-joint surgeries	1.92	(0.89-4.57)	NR
			Hospitalizations	1.79	(0.69-5.25)	NR
			Hospitalizations for depression	0.26	(0.02-1.11)	NR
			Pregnancies	0.68	(0.38-1.20)	NS
**Childhood / jSLE**						
Frittoli, et al, 2016, Brazil[79]	% difference (absolute)	Patients with cognitive dysfunction. Gender, age and socioeconomic status matched	Cognitive dysfunction	4.86		0.080
Gad, et al, 2013, Egypt[81]	% difference (absolute)	Healthy Children. Age and sex matched	Sensorineural hearing loss	25.00		NR
			Conductive hearing loss	0		NR
Karve, et al, 2012, USA[12]	% difference (absolute)	Comparator without IBD or SLE. Age (calculated as of year 2005) and sex, and with a minimum health plan enrolment of 6 months before and 12 months after the assigned index date of the corresponding patient were matched.	Asthma	8.50	NA	NR
			Atopic dermatitis	0.70	NA	NR
			Allergic rhinitis	2.90	NA	NR
			Thyroiditis	0.70	NA	NR
			Fracture in post-index period	2.10	NA	NR
			Psoriasis	0.00	NA	NR
			Type 1 diabetes	-0.70	NA	NR
			General diabetes	0.00	NA	NR
			Multiple sclerosis	1.40	NA	NR
			Other CNS demyelinating disease	0.00	NA	NR
			Vitiligo	0.00	NA	NR
			Rheumatoid arthritis	19.7	NA	NR
Sinicato, et al, 2017, Brazil[94]	% difference (absolute)	Volunteers with no history of chronic, cardiovascular, and autoimmune disease. Age, gender, and demographic background matched	High LDL-c levels	33.00		0.300

			Hypercholesterolemia	16.17	0.210
			Insulin resistance	4.90	0.177
			Arterial hypertension	9.80	0.074
			Albumin excretion	12.20	0.080
**Adulthood/ jSLE**					
Sinicato, et al, 2017, Brazil[94]	% difference (absolute)	Volunteers with no history of chronic, cardiovascular, and autoimmune disease. Age, gender, and demographic background matched	Insulin resistance	0	0.141
			Hypertension	11.4	0.086
			Albumin excretion	11.4	0.086

Supplementary Table S3: Quality and bias assessment of included *Childhood* papers using modified NOS

Study	Representa-tiveness	Sample size	JIA/jSLE/JDM definitions (exposure)	Ascertainment of comorbidities (outcome)	Total scores
**JIA childhood**					
Demirkaya, E. et al , 2011[23]	0	0	2	1	3
Jain, V. et al, 2001[24]	0	0	2	1	3
Rangel, L. et al, 2003[25]	0	1	0	1	2
Saurenmann, RK. Et al , 2007[26]	1	1	2	1	5
Chhabra, A. et al, 2020[27]	1	0	0	1	2
Beukelman, T et al, 2012[28]	2	1	0	1	4
van Rossum, MA. Et al, 2007[29]	0	1	2	1	4
Gerloni, V. et al, 2008[30]	0	0	2	1	3
Klotsche, J. et al, 2016[31]	0	1	2	1	4
Beukelman, T. et al, 2013[32]	0	1	1	0	2
Schenck, S. et al, 2018[33]	2	1	2	0	5
Angeles-Han S.T. et al, 2015[34]	1	1	0	0	2
Thiele F. et al, 2021[35]	2	0	0	0	2
Kearsley-Fleet L. et al, 2022[36]	2	1	0	1	4
Krause M.L. et al, 2017[37]	0	0	2	1	3
Lee H. et al, 2020[38]	0	1	2	0	3
Horneff G. et al, 2022[39]	0	1	2	0	3
Bolt I.B. et al, 2008[40]	1	0	2	0	3
Simon TA et al, 2020[41]	2	1	2	0	5
Schulz, C. et al, 2022[42]	1	1	0	1	3
Swart, J. et al, 2018[43]	2	0	2	1	5
Giancane G et al,2019[44]	2	1	2	1	6
Grassi, A. et al, 2007[45]	1	1	2	1	5
Horneff, G. et al, 2016[46]	2	1	2	1	6
Lovell, D. J. et al, 2021[47]	1	1	2	1	5
Barthel, D. et al, 2015[48]	2	1	0	1	4
Sahin, S. et al, 2021[49]	2	1	2	1	6
Pohjankoski, H. et al, 2010[50]	0	0	0	1	1
Cakan, M. et al, 2020[51]	1	1	2	1	5
Cosickic, A. et al, 2017[52]	1	0	2	1	4
Berthold, E. et al, 2022[53]	2	0	1	1	4
Lien, G. et al, 2003[54]	1	0	0	0	1
Saraux A. et al, 2015[55]	2	0	2	0	4
Alberdi-Saugstrup M et al, 2017[56]	2	0	2	1	5
Amine B et al, 2011[57]	1	1	2	1	5
**jSLE childhood**					
Davis A et al, 2022[73]	0	1	1	1	3
Garf, K. E. et al, 2015[74]	0	0	2	0	2
Paim-Marques, L. et al, 2019[75]	0	0	0	1	1
Silva, M. F. et al, 2015[76]	2	0	2	1	5
Knight, A. M. et al, 2016[77]	2	1	1	1	5

Das Chagas Medeiros, MM. et al, 2016[78]	1	1	2	0	4
Frittoli, R. B. et al, 2016[79]	1	1	2	1	5
Holland , M. J. et al, 2018[80]	2	0	2	1	5
Gad, G. I. et al, 2013[81]	1	1	2	1	5
Jongvilaikasem, P. and Rianthavorn, P., 2021[82]	1	0	2	1	4
AlAhmed O et al, 2020[83]	2	1	2	1	6
Karve S et al, 2012[12]	0	1	2	1	4
Ye Q et al, 2021[84]	1	0	0	0	1
Cann MP et al, 2022[85]	1	1	2	1	5
Medhat MB et al, 2020[86]	1	0	2	1	4
Aggarwal A et al, 2018[87]	1	1	2	0	4
Al-Mayouf, SM. and Al Sonbul, A., 2008[88]	1	0	2	1	4
Costagliola G et al, 2018[89]	1	0	2	1	4
Torrente-Segarra V et al, 2017[90]	2	1	2	0	5
Ponin, L. et al, 2022[91]	1	0	2	1	4
Avar Aydin, P. O. et al, 2018[92]	1	1	2	1	5
Ahluwalia, J. et al, 2014[93]	1	1	2	1	5
Sinicato, N. A. et al, 2017[94]	1	1	2	1	5
Cabral, M. et al, 2013[95]	1	1	2	1	5
Bernatsky, S. et al, 2017[96]	2	0	0	0	2
Yang, Y. Etal, 2015[97]	1	1	2	1	5
Lin, T. C. et al, 2018[98]	0	0	2	1	3
Kahwage, P. P. et al, 2017[99]	2	0	2	1	5
Lai et al, 2022[100]	1	1	1	0	3
**JDM Childhood**					
Huber, A. M. et al, 2000[1]	2	1	0	0	3
Cho SK et al, 2019[107]	2	1	1	1	5
Concannon A and Han DY, 2021[108]	1	1	2	0	4
Okong'o et al, 2016[109]	1	1	2	1	5
Ravelli A et al, 2010[2]	2	0	2	1	5
Tabarki B et al, 1998[110]	1	0	2	0	3
Chevalier, G. et al, 2021[111]	**1**	1	2	1	5
Al-Mayouf, S. M. et al, 2017[112]	**0**	1	2	1	4
Sag, E. et al, 2021[113]	**0**	1	2	1	4
Ramanan, A. V. et al, 2005[114]	**0**	1	0	0	1
Sun, C. et al, 2015[115]	**0**	0	2	0	2
Silverberg, J. I. et al, 2018[116]	**2**	1	2	1	6

Supplementary Table S4: Quality and bias assessment of selected *Adulthood* papers using modified NOS

Study	Representa-tiveness	Sample size	JIA/jSLE/JDM definitions (exposure)	Ascertainment of comorbidities (outcome)	Total scores
**JIA adulthood**					
Smitherman EA et al, 2023[58]	1	0	2	0	3
Mena-Va¡zquez, N et al, 2022[14]	0	1	2	1	4
Raab, A. et al, 2013[59]	2	1	2	1	6
Aggarwal, P. et al, 2006[60]	1	0	2	1	4
Arvidsson, L. Z. et al, 2010[61]	1	0	2	1	4
Packham, J. C. et al, 2002[62]	1	1	2	1	5
Mars, N. J. et al, 2019[63]	1	0	0	1	2
Dimopoulou, D. et al, 2017[64]	1	1	2	0	4
Rebane, K. et al , 2022[65]	2	0	0	1	3
Minden, K. et al, 2019[66]	2	1	2	1	6
Smith, C. J. F. et al, 2019[67]	0	1	0	0	1
Barth S et al, 2016[68]	0	1	0	0	1
Simon, T. A. et al, 2020[41]	2	1	1	0	4
Packham JC and Hall MA, 2003[69]	1	1	2	0	4
Ma, K. S. K. et al, 2022[70]	2	0	2	1	5
Zhang-Jian, S. J. et al, 2020[71]	2	0	1	1	4
Rypdal, V. et al, 2021[72]	2	1	1	1	5
**jSLE adulthood**					
Al Hamzi, H. et al, 2014[101]	1	1	2	1	5
Tanigava, N. Y. et al, 2022[102]	0	0	0	1	1
Hersh, A. O. et al, 2009[103]	0	1	2	1	4
Sousa S et al, 2016[104]	2	1	2	1	6
Koutsonikoli A et al, 2015[105]	1	1	2	1	5
Gamal SM et al, 2022[106]	1	0	2	1	4
Groot N et al, 2019[4]	2	1	2	1	6
Sinicato, N. A. et al, 2017[94]	1	1	2	1	5
**JDM adulthood**					
Boros, C. et al, 2022[117]	0	1	0	0	1
Mathiesen, P. R. et al, 2014[118]	2	1	2	1	6
Tsaltskan V et al, 2020[5]	2	1	2	0	5
Mathiesen P et al , 2012[119]	2	1	1	1	5
Sanner, H. et al, 2010[120]	2	1	2	1	6

Supplementary Table S5: Articles on childhood and adulthood JIA, jSLE and JDM and corresponding NOS scores

NOS scores	JIA childhood	JIA adulthood	jSLE childhood	jSLE adulthood	JDM childhood	JDM adulthood
1	2	2	2	1	1	1
2	5	1	2	0	1	0
3	8	2	3	0	2	0
4	8	7	8	2	3	0
5	9	3	13	2	4	2
6	3	2	1	2	1	2
**Total articles (105)**	**35**	**17**	**29**	**7**	**12**	**5**

Supplementary Table S6: Pooled prevalence of comorbidities reported associated with JIA, jSLE and JDM patients presenting in childhood or apparent in adulthood[1]

i) JIA

Childhood					Adulthood				
**Disease**	**Comorbidities**	**Studies**	**PP (%), (95% CI)**	**I^2 (%)**	**Disease**	**Comorbidities**	**Studies**	**PP(%); (95% CI)**	**I^2 (%)**
**JIA**	Amyloidosis	4	0.47; [0.13-1.70]	50.10%	**JIA**	Anxiety	3	6.18; [2.36-15.25]	89.10%
	Anxiety	4	5.02; [2.00-12.06]	98.80%		Asthma	3	8.36; [7.88-8.88]	0.00%
	Asthma	3	8.84; [7.86-9.93]	88.70%		Cataract	3	7.48; [0.87-42.66]	95.70%
	Autoimmune thyroiditis	4	1.34; [0.94-1.89]	0.00%		Osteoporosis	3	1.58; [0.26-9.18]	94.40%
	Band keratopathy	7	2.01; [0.66-5.93]	90.00%		Depression	5	9.11; [4.42-17.87]	85.80%
	Cataract	7	2.32; [1.39-3.85]	63.00%		Diabetes (undefined)	6	3.19; [1.36-7.31]	81.40%
	Depression	9	3.61; [1.80-7.11]	97.30%		Glaucoma	3	7.11; [1.50-27.74]	96.00%
	Diabetes (undefined)	8	0.92; [0.72-1.18]	55.00%		Hypertension	5	3.81; [1.59-8.85]	96.20%
	Glaucoma	5	1.84; [1.33-2.56]	1.90%		Malignancy	3	3.45; [2.14-5.53]	93.20%
	Herpes zoster	3	1.33; [0.64-2.75]	95.70%		Uveitis	7	14.46; [9.76-20.90]	89.10%
	Hypothyroidism	4	1.52; [1.01-2.28]	24.10%					
	Hypertension	5	0.29; [0.04-1.97]	95.80%					
	Inflammatory bowel diseases	5	0.42; [0.28-0.63]	60.20%					
	Malignancy	4	0.40; [0.23-0.68]	85.60%					
	Macrophage activation syndrome	6	3.21; [0.84-11.53]	98.90%					
	Macular oedema	3	1.12; [0.45-2.76]	70.10%					

[1] A comorbidity reported in at least three separate studies, either presenting in childhood or apparent in adulthood, in JIA, jSLE and JDM patients, was considered to estimate the pooled prevalence in meta-analysis. For JIA patients, amyloidosis and malignancy were reported in five articles each presenting in childhood. One article each did not report the exact number of cases. Hence, four articles were considered to estimate the pooled prevalence of each comorbidity. Ulcerative colitis was reported in three separate articles presenting in childhood, of which two did not report the exact number of cases. Therefore, it was not possible to estimate the pooled prevalence for ulcerative colitis.

	Multiple sclerosis	5	0.02; [0.01-0.05]	0.00%
	Osteoporosis	3	0.72; [0.37-1.38]	0.00%
	Psoriasis	3	3.99; [3.16-05.03]	4.90%
	Synechiae	4	3.39; [2.67-4.30]	0.00%
	Type 1 Diabetes	3	0.42; [0.11-1.58]	96.10%
	Tuberculosis/TB	6	0.18; [0.04-0.90]	94.10%
	Uveitis	18	13.95; [11.79-16.43]	91.50%
	Varicella	5	10.31; [1.79-42.04]	99.50%

ii) jSLE

Childhood					Adulthood				
Disease	Comorbidities	Studies	PP (%), (95% CI)	I^2 (%)	Disease	Comorbidities	Studies	PP(%); (95% CI)	I^2 (%)
jSLE	Anxiety	3	11.29; [6.73-18.33]	85.60%	jSLE	Avascular necrosis	4	9.90; [4.82-19.28]	69.60%
	Avascular necrosis	8	2.20; [1.20-4.02]	76.40%		Cataract	3	6.62; [3.95-10.87]	0.00%
	Cataract	6	6.39; [3.49-11.41]	87.80%		Diabetes (undefined)	4	3.82; [1.88-7.58]	39.20%
	Chronic kidney disease	3	48.38; [0.67-99.24]	93.60%		End-stage renal disease	4	7.59; [5.31-10.75]	20.00%
	Cerebrovascular accident	4	3.15; [1.06-8.93]	74.50%		Hypertension	4	18.30; [7.52-38.16]	92.40%
	Depression	3	18.27; [16.47-20.21]	58.40%		Malignancy	3	1.12; [0.39-3.14]	0.00%
	Diabetes (undefined)	8	1.98; [1.07-3.62]	55.50%		Premature gonadal failure	3	2.83; [0.87-8.82]	39.50%
	End-stage renal disease	7	3.62; [2.03-6.39]	78.00%		Pulmonary fibrosis	3	3.14; [1.50-6.44]	0.00%
	Growth failure	4	11.73; [5.99-21.70]	78.10%		Pulmonary infarction	3	1.09; [0.27-4.27]	0.00%
	Obesity	3	18.86; [5.56-47.84]	93.20%		Seizures	3	11.24; [4.22-26.68]	82.50%
	Hypertension	8	19.14; [13.05-27.18]	85.80%		Transverse myelitis	3	1.09; [0.27-4.27]	0.00%

	Malignancy	4	0.33; [0.11-0.93]	0.00%		Venous thrombosis	3	7.35; [4.39-12.04]	43.50%
	Osteoporosis	5	4.05; [2.61-06.24]	56.50%					
	Premature gonadal failure	3	1.92; [1.27-2.90]	0.00%					
	Pulmonary hypertension	3	0.61; [0.16-2.33]	74.50%					
	Transverse myelitis	3	0.73; [0.28-1.93]	52.70%					
	Venous thrombosis	3	1.61; [0.57-4.44]	61.30%					

iii) JDM

Childhood					Adulthood				
**Disease**	**Comorbidities**	**Studies**	**PP (%), (95% CI)**	**I^2 (%)**	**Disease**	**Comorbidities**	**Studies**	**PP (%), (95% CI)**	**I^2 (%)**
**JDM**	Calcinosis	11	29.70; [25.91-33.81]	26.70%	**JDM**	Calcinosis	4	40.37; [19.02-66.11]	92.70%
	Cataract	5	7.75; [2.39-22.39]	88.90%		Lipodystrophy	3	15.67; [6.05-34.91]	85.10%
	Contractures	3	17.98; [14.82-21.64]	51.70%					
	Diabetes (undefined)	5	3.52; [1.36-8.79]	86.60%					
	Growth failure	3	22.85; [7.66-51.39]	96.50%					
	Interstitial lung disease	3	6.85; [1.48-26.48]	81.00%					
	Osteoporosis/ Osteopenia	4	8.51; [3.91-17.56]	72.90%					
	Lipodystrophy	4	8.11; [3.38-18.21]	57.70%					
	Malignancy	4	0.89; [0.18-4.42]	27.00%					
